# Identification and Diversity of Killer Cell Ig-Like Receptors in *Aotus vociferans*, a New World Monkey

**DOI:** 10.1371/journal.pone.0079731

**Published:** 2013-11-06

**Authors:** Diego Garzón-Ospina, Carolina López, Luis F. Cadavid, Manuel E. Patarroyo, Manuel A. Patarroyo

**Affiliations:** 1 Molecular Biology and Immunology Department, Fundación Instituto de Inmunología de Colombia (FIDIC), Bogotá, Colombia; 2 School of Medicine and Health Sciences, Universidad del Rosario, Bogotá, Colombia; 3 Institute of Genetics and Department of Biology, Universidad Nacional de Colombia, Bogotá, Colombia; National HIV and Retrovirology Laboratories, Canada

## Abstract

Previous BAC clone analysis of the Platyrrhini owl monkey KIRs have shown an unusual genetic structure in some loci. Therefore, cDNAs encoding KIR molecules from eleven *Aotus vociferans* monkeys were characterized here; ten putative KIR loci were found, some of which encoded atypical proteins such as KIR4DL and transcripts predicted to encode a D0+D1 configuration (AOTVOKIR2DL1*01v1) which appear to be unique in the *Aotus* genus. Furthermore, alternative splicing was found as a likely mechanism for producing activator receptors in *A. vociferans* species. KIR proteins from New World monkeys may be split into three new lineages according to domain by domain phylogenetic analysis. Although the *A. vociferans* KIR family displayed a high divergence among paralogous genes, individual loci were limited in their genetic polymorphism. Selection analysis showed that both constrained and rapid evolution may operate within the AvKIR family. The frequent alternative splicing (as a likely mechanism generating activator receptors), the presence of KIR4DL and KIR2DL1 (D0+D1) molecules and other data reported here suggest that the KIR family in *Aotus* has had a rapid evolution, independent from its Catarrhini counterparts.

## Introduction

Natural killer (NK) cells are granular lymphocytes which are involved in immune responses against infected or malignant cells [1]. NK cells have two effector responses: the direct cytotoxicity of target cell and cytokine production; the former function is mediated by balancing different receptors’ inhibitory and activating stimulation on NK surface. The large NK receptor repertoire includes *killer cell immunoglobulin (Ig*)*-like receptors* (KIRs), *natural killer cell receptors* (NKRs), leukocyte Ig-like receptors (LIRs), natural cytotoxicity receptors (NCRs), *sialic acid binding Ig-like lectin 7* (Siglec-7) and members of *NK lectin-like* receptor *2* (NKG2) [2].

Multigene KIR family members are localized in the leukocyte receptor complex (LRC) and encode type I membrane proteins which have two or three Ig-like domains (D0, D1 and D2, according to their proximity to the membrane). KIRs having three domains are denoted as KIR3D and proteins with two domains as KIR2D. The cytoplasmic region can be long (L), having one or two immunoreceptor tyrosine-based inhibitory motifs (ITIMs) conducting inhibitory responses or, it can be short (S), interacting with adapter molecules having immunoreceptor tyrosine-based activation motifs (ITAMs) and thereby triggering activating signals [3-5].

The KIR haplotype has been characterizing in several primates: the chimpanzee [6,7], the gorilla [8], the orangutan [9], the rhesus macaque [10], the crab-eating macaque [11], baboons [12], the green monkey [13], the vervet monkey, the olive baboon, the colobus monkey [14] and an *A. nancymae* - *A. azarai* hybrid owl monkey [15]. KIRs evolutionary history has thus been thoroughly assessed in Catarrhini primates while a full understanding of such background has yet to be achieved in Platyrrhini monkeys. Sequence analysis of these Catarrhini species has shown a fast evolution and many of them have diverged in a species-specific manner [7,16]. Phylogenetic analysis has shown that domain shuffling has allowed the emergence of new primate receptors [8]. According to their phylogenetic relationships, five lineages can be observed, lineage I being represented by human KIR2DL4 and KIR2DL5 (KIR2DL4 being the only true ortholog KIR among Catarrhini primates) [7,14]. Lineage II includes human KIR3DL1 and KIR3DL2 which interact with class I molecules (MHC-A and -B). Lineage III is represented by KIRs having a D1+D2 and D0+D1+D2 configuration interacting with the MHC-C protein. *Rhesus macaque* KIRs form lineage IV and lineage V encompasses human KIR3DL3 [8].

Cadavid and Lun [15] analyzed a bacterial artificial chromosome from a hybrid F1 *Aotus* monkey; they showed that *Aotus* KIR gene models form a monophyletic clade (lineage VI), suggesting that differential diversification of the KIR family occurred after the divergence of hominids, Old World primates and New World monkeys [15]. This report has thus been aimed at characterizing the KIR repertoire from field sampled *A. vociferans* individuals, showing this family’s great diversity and rapid evolution in this species.

## Results

### Species identification

Morphological similarity in several *Aotus* species has led to frequent misidentification [17]; a fragment of cytochrome oxidase subunit II (COII) was thus sequenced before KIR gene amplification. This was then aligned with reported COII from different *Aotus* species and maximum likelihood (ML) phylogeny was then constructed by using the best nucleotide substitution model (i.e. the Hasegawa, Kishino and Yano (HKY) model, assuming a proportion of invariant sites (*+I*)) inferred by Bayesian information criteria (BIC). All COII fragments from the individuals included in this study formed a monophyletic group with the reported *A. vociferans* COII (Figure S1), confirming that the field sampled primates belonged to this species. 

### Characterizing KIR loci, alleles and splice variants

**Figure 1:**
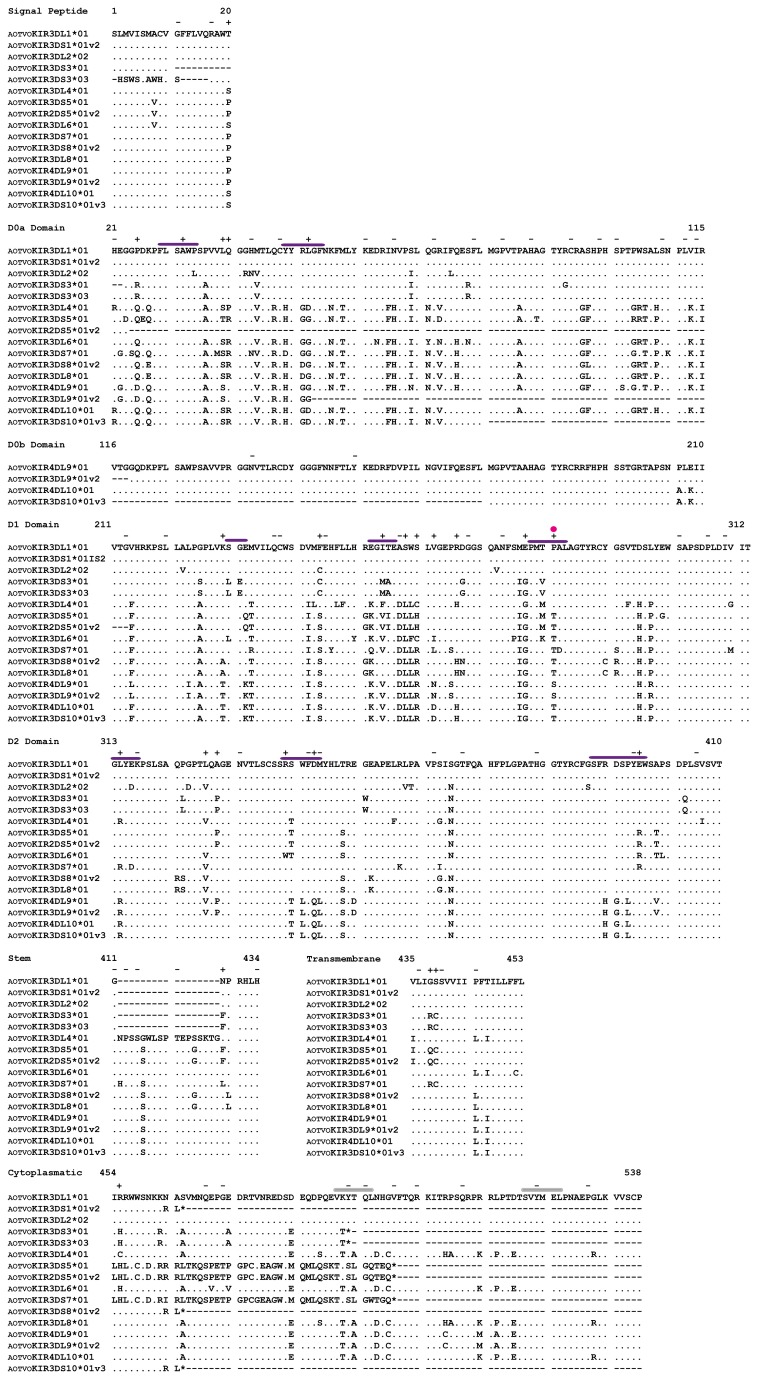
*A. vociferans* predicted amino acid KIR sequence loci alignment. Dots (.) indicate identity among AOTVOKIR sequences, dashes (-) indicate absence of amino acids. ITIMs are indicated by gray bars above the motifs. According to previous reports in humans, putative MHC binding regions of AOTVOKIR are indicated by purple bars above the amino acid sequence and magenta dots indicate site that could be in contact with the peptide being presented in class I. + and – symbols denote sites under positive and negative selection, respectively. Not all characterized sequences are shown.

**Figure 2:**
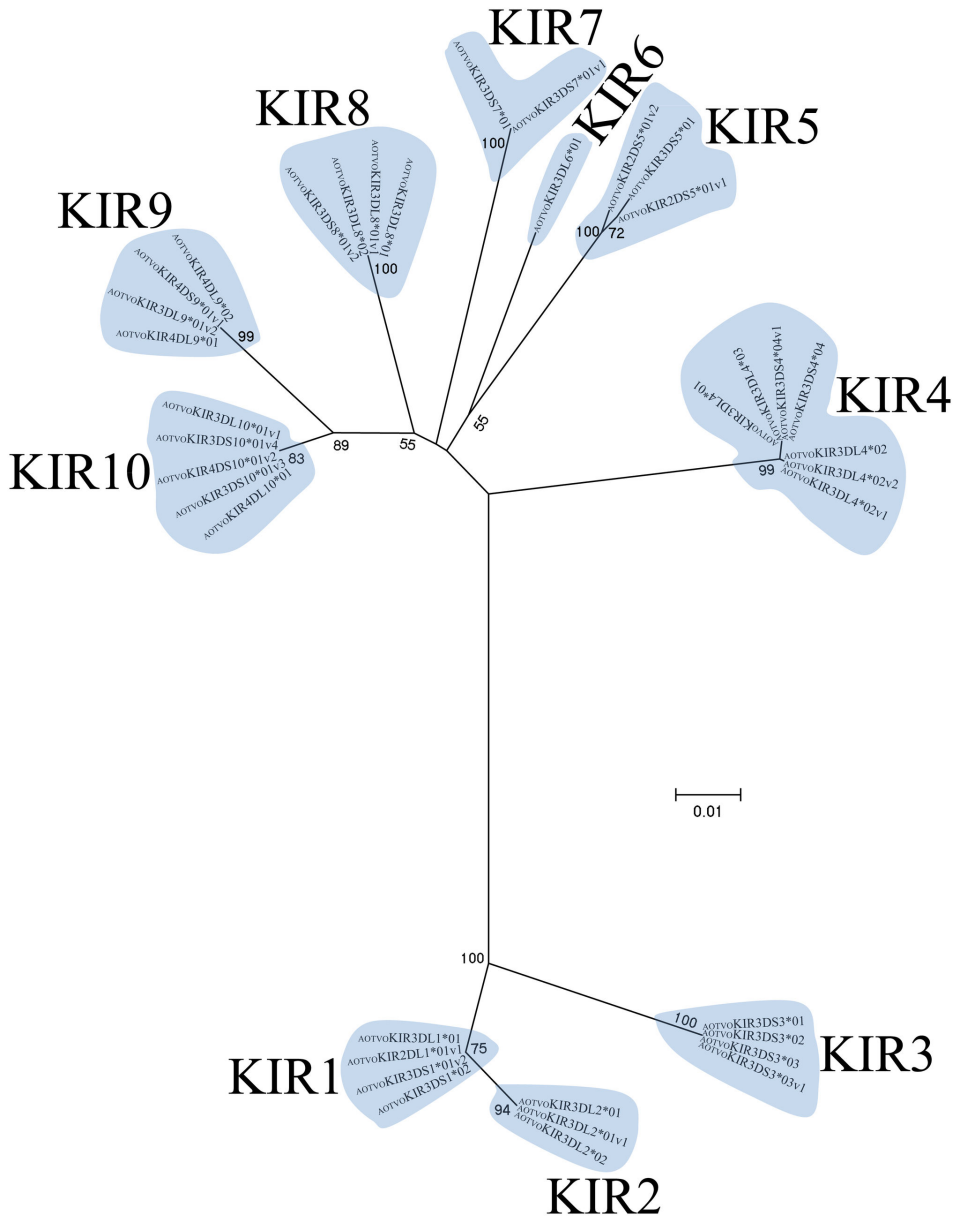
*A. vociferans* allele phylogenetic relationships. The topology was inferred by ML method using a GTR +*G* model. Numbers designate the KIR putative locus and numbers on branches represent bootstrap values (1,000 replicates).

A total of 52 amplicons ranging between 1,000 and 1,600 base pairs were found in 11 animals analyzed. The open reading frames (ORF) encoded proteins having around 308 to 538 amino acids (Figure 1); 1 to 4 genes were identified per individual (Table S1). Each putative *A. vociferans* KIR gene (**AOTVO**KIR) was assigned a number according to the phylogenetic tree shown in Figure 2 (KIR1-KIR10). The allele/splice variants were named in an analogous fashion to the IPD-KIR database [18]. Each KIR transcript was assigned a number following the KIR acronym corresponding to the number of Ig-like domains in the molecule (e.g. AOTVOKIR**3DL** for a long cytoplasmic tail or AOTVOKIR**3DS** for a short cytoplasmic one). Each putative KIR receptor was assigned a number indicating the gene encoding it (AOTVOKIR3DL**1**… AOTVOKIR4DL**10**) and the splicing variants from an appointed gene were assigned the same number (e.g. AOTVOKIR4DL10*01 and AOTVOKIR3DS10*01v3 were encoded by the same gene). An asterisk was used as a separator before a numerical allele designation. Two digits were used to indicate alleles whose encoded protein sequence differed by a non-synonymous substitution. The variants generated by alternative splicing were designed by the letters “v” and a number (e.g. AOTVOKIR3DS3*03**v1**). *A. vociferans* KIR sequences found at least twice after sequencing several clones or present in two or more individuals are available in GenBank: accession numbers KF014088-KF014122.

*A. vociferans* KIRs having 2, 3 and 4 Ig-like domains were identified (Figure 1, and Figures S2-S10). These sequences were aligned and a phylogenetic tree was inferred. Ten clades can be observed in Figure 2, each representing a single *A. vociferans* putative locus (AOTVOKIR1- AOTVOKIR10). Twenty alleles (Table S1) were identified from these 10 loci and several splice variants appear to have been generated by alternative splicing (Figures S11 and S12). The AOTVOKIR1 locus encoded receptors having 2 or 3 Ig-like domains with either long or short cytoplasmatic tails (Figure S2). Long cytoplasmatic tail KIRs had two distinguishing ITIMs (VKYTQL and SVYMEL) thus encoding inhibitory receptors. Two alleles were found in this locus. Allele 1 (AOTVOKIR3DL1*01) had 2 variants apparently generated by alternative splicing (Figure S11). Deletion of the D2 domain in allele 1 generated a KIR protein having 2 Ig-like domains (AOTVOKIR-2DL1*01v1). In addition to this variant, a new one encoded a short cytoplasmatic tail KIR lacking the transmembrane charged residue (Figure S2).

The AOTVOKIR2 locus encoded proteins having 3 Ig-like domains with long tails with the same two ITIM motifs as AOTVOKIR1 (Figure S3). AOTVOKIR2 also had 2 alleles. The AOTVOKIR3DL2*01 allele had one splice variant; such variant (AOTVOKIR3DL2* 01v1) had a 12-residue deletion at the beginning of the D1 domain (Figure S3) due to the absence of 36nt in exon 4 that might have been generated by the use of an alternative acceptor site (Figure S11).

The AOTVOKIR3 locus had three alleles encoding activator receptors. Exon 2 was absent in AOTVOKIR3DS3*01 and *02 alleles, while the AOTVOKIR3DS3*03 allele had 19nt from this exon (Figure S11) causing a shift in the reading frame. Additionally, this allele had one splice variant which lacks exon 7, generating a 35-residue loss where stem and transmembrane domains are absent (Figure S4). On the other hand, AOTVOKIR3DS3*02 allele differed in sequence at the C-terminal region of the cytoplasmatic tail (Figure S4). However, the three alleles shared the charged amino acid position (residue 4 of the transmembrane domain).

The fourth locus (AOTVOKIR4) encoded inhibitory receptors having 3 Ig-like domains, as well as others having uncommon C-terminal regions (Figure S5). The former had the ITIM motif (VTYAQL and SVYMEL) that differed from the aforementioned KIRs. The AOTVOKIR3DL4*02 allele had two splice variants. The first (AOTVOKIR3DL4*02v1) had an INDEL covering the signal peptide; variant 2 (AOTVOKIR3DL4*02v2) had an insertion at the beginning of the stem domain (Figure S5). Similarly to inhibitory receptors, the “short-tailed” ones seem to be generated by alternative splicing (Figure S12). Despite having short cytoplasmatic tails, the positively charged amino acid was absent in these receptors. The AOTVOKIR3DS4*04v1 splice variant seems to have an in-frame intron fragment included, which led to an uncommon C-terminal sequence. No transmembrane region was found when Phobius [19] and TMHMM [20] predictors were used. 

Two alleles encoding short-tailed receptors were found in the AOTVOKIR5 locus. Due to two deletions of 1nt in exon 7, donor splicing site changed (Figure S12), joining exon 8 in a different frame and generating a premature stop codon. Likewise, alternative splicing (Figure S12), generated 2 variants having a complete (AOTVOKIR2DS5*02v2) or incomplete (AOTVOKIR2DS5*02v1) D0a domain deletion and KIRs having 2 Ig-like domains were thus encoded (Figure S6). The positively charged amino acid was absent in these receptors. Only one allele was identified in the AOTVOKIR6 locus which encoded an inhibitory protein with 3 Ig-like domains having the same ITIMs as the AOTVOKIR4 inhibitor. 

The two AOTVOKIR7 sequences had 100% identity in the three Ig-like domains, but contained rare C-terminal portions, which were only found once (Figure S7). The AOTVOKIR3DS7*01 allele had typical stem and transmembrane domains and also a rare cytoplasmatic region due to a two deletions of 1nt each at the end of exon 7 that changed the reading frame and the donor splicing site (Figure S12); however, this allele had an arginine in position 4 of the transmembrane domain (Figure S7). No transmembrane domain was found using Phobius and TMHMM predictors with the AOTVOKIR3DS7*01v1 allele. 

The AOTVOKIR8 locus encoded short- and long-tailed proteins with 3 Ig-like domains. The short tail KIR (AOTVOKIR3DS8*01v2) had no charged amino acid in the transmembrane domain and seemed to be a splice variant considering that exon 5 joined to exon 7 (Figure S12) in a different frame generating a premature stop codon. The long-tailed protein had the two typical VTYAQL and VYMEL ITIMs; 2 alleles were found in this locus and in addition to the variant mentioned above, another splicing variant (AOTVOKIR3DL8*01v1) produced by the absence of 17-residues in the stem domain was found in the AOTVOKIR3DL8*01 allele (Figure S8 and Figure S12). 

The AOTVOKIR9 and AOTVOKIR10 loci encoded proteins having 4 Ig-like domains (Figure S9 and Figure S10). The former encoded inhibitory receptors and one possible secreted KIR which did not have a stem, transmembrane or cytoplasmatic domain (Figure S9). This locus had two alleles; a KIR protein having 3 Ig-like domains where most of the D0a domain was absent was probably generated by an alternative splicing in the AOTVOKIR4DL9*01 allele (Figure S9 and Figure S12). The AvKIR10 locus encoded short-, long-tailed receptors and secreted proteins. The AOTVOKIR10 inhibitor and secreted (splice variant) receptor proteins had a similar genetic structure to that of AOTVOKIR9 (Figure S10). One allele and four splice variants were found at this locus. Joints between exon 7 and exon 9 were found in the former splice variant (AOTVOKIR4DS10*01v2 and AOTVOKIR3DS10*01v3) thus encoding short-tailed receptors that lacked the charged amino acid in the transmembrane domain. Part of the D0a and D0b domains were absent in the AOTVOKIR3DS10*01v3 and AOTVOKIR3DS10*01v4 variants (possibly generated by alternative splicing [Figure S12]) and the latter variant (AOTVOKIR3DL10*01v1) lacked most of the D0a domain (Figure S10), thus generating KIRs having 3 Ig-like domains.

### Phylogenetic and recombination analysis

*A. vociferans* KIR nucleotide sequences (AOTVOKIR) were aligned together with previously reported human (KIR), chimpanzee (*Pan troglodytes* - PANTRKIR), gorilla (*Gorilla gorilla* - GORGOKIR), orangutan (*Pongo pygmaeus* - PONPYKIR), rhesus monkey (*Macaca mulatta* - MACMUKIR), and owl monkey (*Aotus* sp - OmKIR) KIR sequences. Gene trees were then generated using maximum likelihood (ML) with different evolutionary models. Full-length DNA trees constructed with generalized time reversible‎ (GTR) and a discrete Gamma distribution (*+G*) model revealed four main clades (Figure S13). The *Cercopithecidae* KIR (MACMUKIR) genes clustered in a paraphyletic clade, the *Hominidea* species (PONPYKIR, GORGOKIR, PANTRKIR and KIR) came together in another clade and the Platyrrhini *A. vociferans* and *Aotus* sp clustered in a monophyletic clade. The ancestral KIRs clustered together, lying outside the clades mentioned above (Figure S13). 

**Figure 3:**
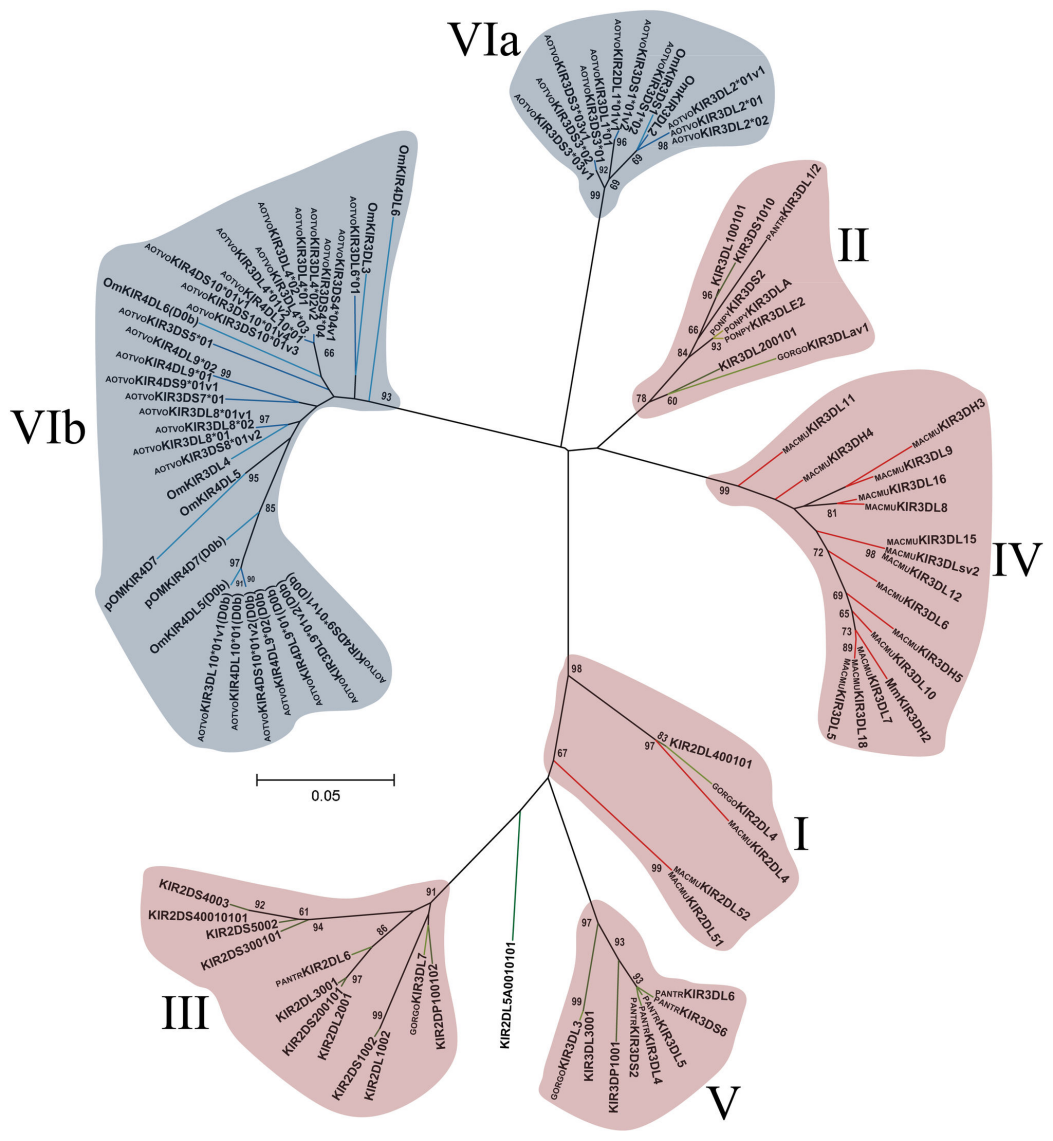
Primate D0 Ig-like domain phylogenetic tree. The tree was constructed from DNA sequences encoding the D0 Ig-like domain using the ML method based on the K2 [39] *+G* model. Primate KIR lineages are indicated by roman numerals. The *Aotus* KIR lineages (VI) are shown with a blue background and the other primate lineages (I, II, III, IV and V) are depicted with a red background. Numbers on branches represent bootstrap percentages after 1,000 replicates. Human allele sequences have no prefix; GORGO, *Gorilla* *gorilla*; PANTR, *Pan* *troglodytes*; PONPY, *Pongo* *pygmaeus*; MACMU, *Macaca* *mulatta*; Om, *Aotus* sp and AOTVO, *A*. *vociferans*. Branches in red represent *Cercopithecidae* KIR, green *Hominidea* KIR and blue branches combine Platyrrhini KIR.

**Figure 4:**
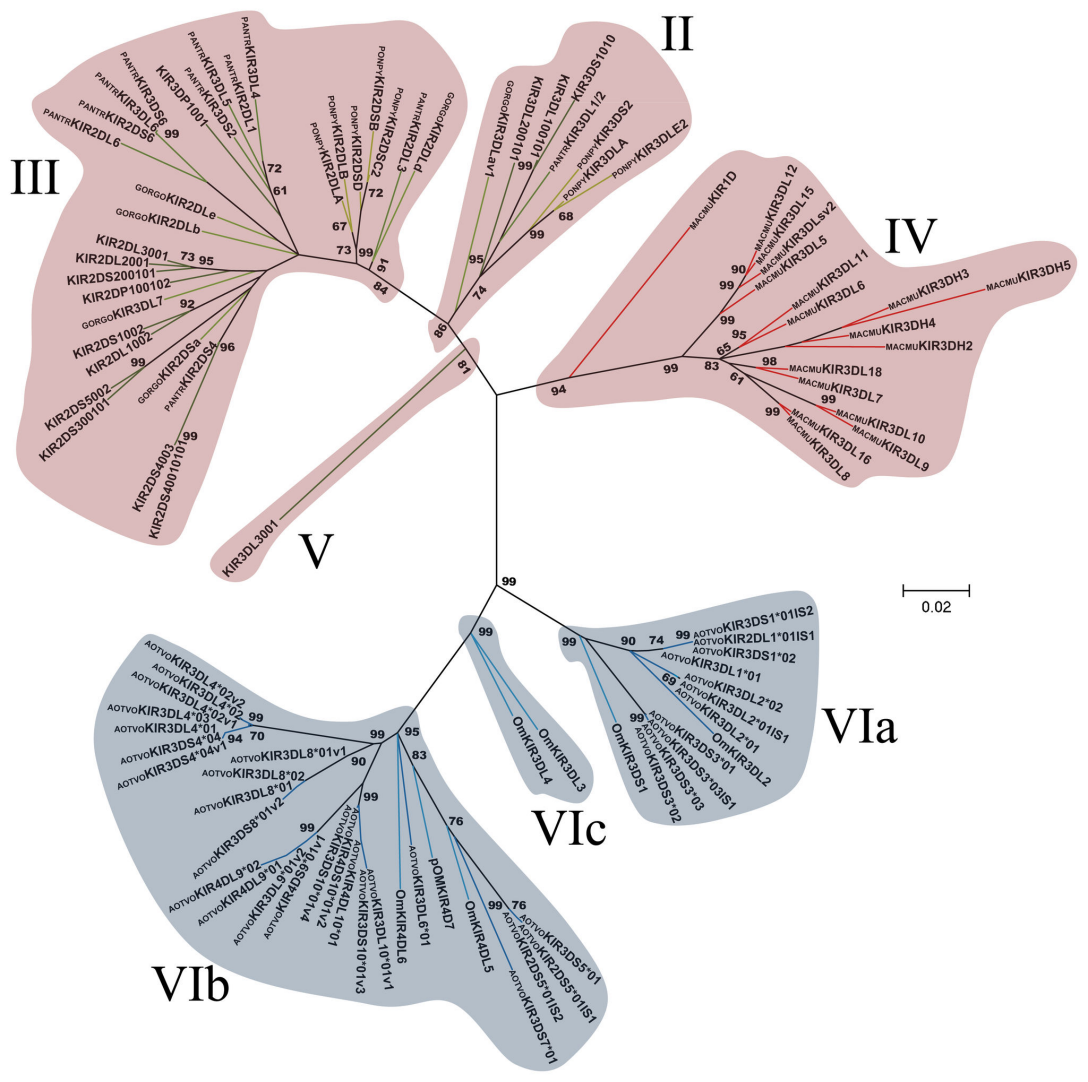
Primate D1 Ig-like domain phylogenetic tree. The tree was constructed from DNA sequences encoding the D1 Ig-like domain, using the ML method based on the K2 [39] *+G* model. Primate KIR lineages are indicated by roman numerals. The *Aotus* KIR lineages (VI) are shown with a blue background and the other primate lineages (I, II, III, IV and V) are depicted with a red background. Numbers on branches represent bootstrap percentages after 1,000 replicates. Human allele sequences have no prefix; GORGO, *Gorilla* *gorilla*; PANTR, *Pan* *troglodytes*; PONPY, *Pongo* *pygmaeus*; MACMU, *Macaca* *mulatta*; Om, *Aotus* sp and AOTVO, *A*. *vociferans*. Branches in red represent *Cercopithecidae* KIR, green *Hominidea* KIR and the blue branches combines Platyrrhini KIR.

**Figure 5:**
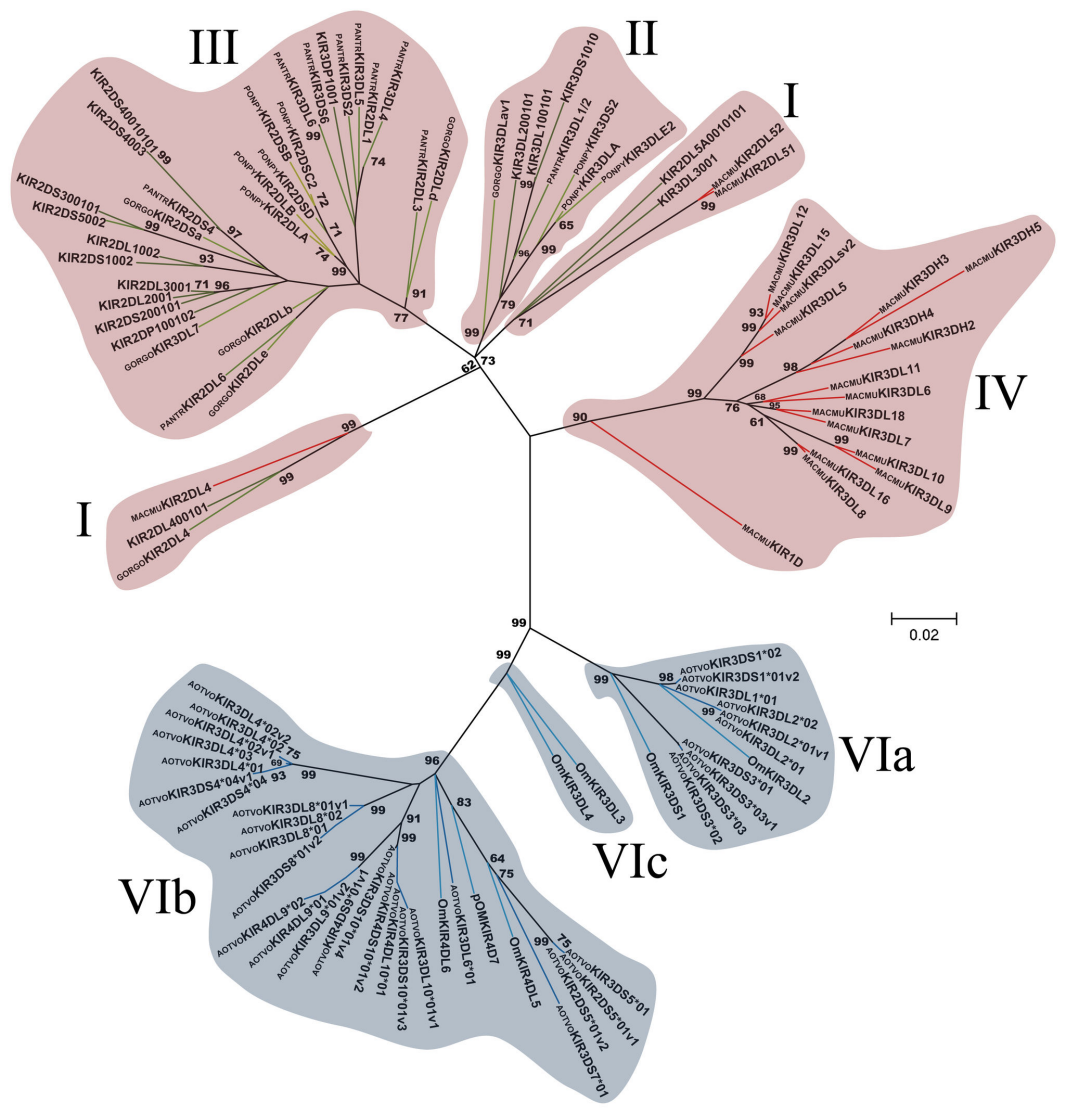
Primate D2 Ig-like domain phylogenetic tree. The tree was constructed from DNA sequences encoding the D2 Ig-like domain using the ML method based on the K2 [39] *+G* model. Primate KIR lineages are indicated by roman numerals. The *Aotus* KIR lineages (VI) are shown with a blue background and the other primate lineages (I, II, III, IV and V) are depicted with a red background. Numbers on branches represent bootstrap percentages after 1,000 replicates. Human alleles sequences have no prefix; GORGO, *Gorilla* *gorilla*; PANTR, *Pan* *troglodytes*; PONPY, *Pongo* *pygmaeus*; MACMU, *Macaca* *mulatta*; Om, *Aotus* sp and AOTVO, *A*. *vociferans*. Branches in red represent *Cercopithecidae* KIR, green *Hominidea* KIR and blue branches combine Platyrrhini KIR.

**Figure 6:**
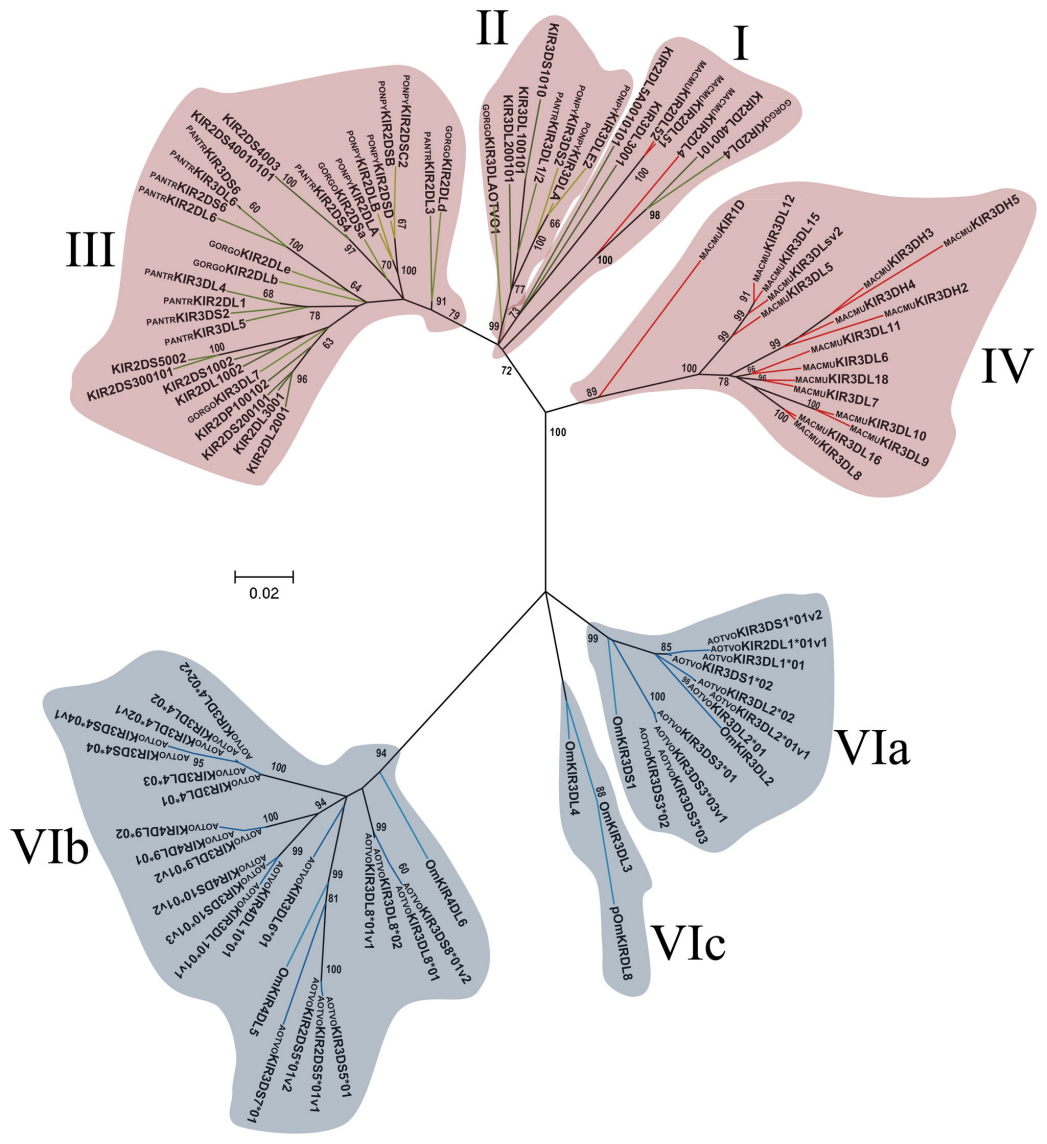
Phylogenetic tree for higher primates’ stem, transmembrane and cytoplasmatic domains. The tree was constructed from stem, transmembrane and cytoplasmatic domains using the ML method based on the Tamura-Nei [47] (TN93) *+G* model. Primate KIR lineages are indicated by roman numerals. The *Aotus* KIR lineages (VI) are shown with a blue background and the other primate lineages (I, II, III, IV and V) are depicted with a red background. Numbers on branches represent bootstrap percentages after 1,000 replicates. Human alleles sequences have no prefix; GORGO, *Gorilla* *gorilla*; PANTR, *Pan* *troglodytes*; PONPY, *Pongo* *pygmaeus*; MACMU, *Macaca* *mulatta*; Om, *Aotus* sp and AOTVO, *A*. *vociferans*. Branches in red represent *Cercopithecidae* KIR, green *Hominidea* KIR and blue branches combine Platyrrhini KIR.

**Figure 7:**
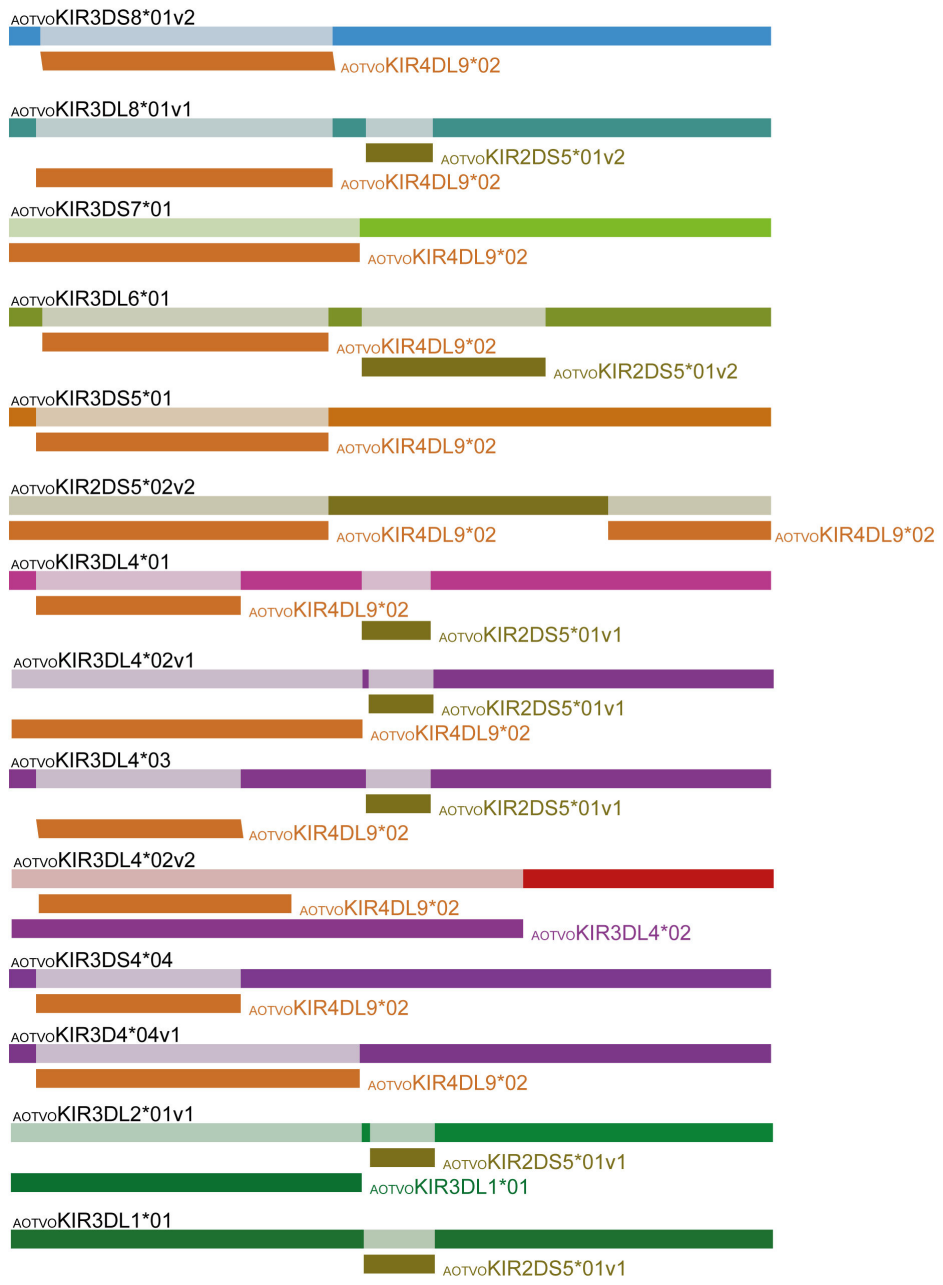
Schematic representation of recombination fragments among AOTVOKIR sequences. The sequence names in black above the rectangles indicate the recombinant sequence name. The rectangle with a name to the right or below (name of the close relative minor parent) shown in different colors is a graphical representation of a sequence fragment that has potentially been derived through recombination. Only recombination events having ≤ 0.03 p-value were taken into account.

Domain by domain phylogenetic analysis of primate KIR genes showed the characteristic lineages (I to VI) (Figures 3 - 6). According to inferred phylogenies, lineage VI reported by Cadavid et al. [15], might be split into three new lineages. The topology of D0 (Figure 3) showed two clades in lineage VI. The first clade (VIa) clustered the AOTVOKIR1, AOTVOKIR2 and AOTVOKIR3 loci, as well as the OmKIR3DS1 and OmKIR3DL2 genes. The second clade (VIb) brought together the remaining AOTVOKIR loci (AOTVOKIR4, AOTVOKIR5, AOTVOKIR6, AOTVOKIR7, AOTVOKIR8, AOTVOKIR9 and AOTVOKIR10) along with OmKIR3DL3, OmKIR3DL4, OmKIR4DL5, OmKIR4DL6 and pOmKIR4DL7 genes. Moreover, this tree showed that the D0b domain formed a paraphyletic group in lineage VIb where the OmKIR4DL6-D0b domain appeared to be more phylogenetically related to D0a from the AOTVOKIR4 and AOTVOKIR10 loci. The estimated evolutionary genetic distances between D0 domains showed that the OmKIR4DL6-D0b domain had low values when compared to the AOTVOKIR4-D0a domains, whereas the remaining D0b domains had the lowest values and appeared to be closest to the AOTVOKIR8-D0a domain. Phylogenies from the D1, D2 and STC (stem, transmembrane and cytoplasmatic) domains showed similar topologies to those in Figure 3, clustering the AOTVOKIR1 to AOTVOKIR3 loci into clade VIa and AOTVOKIR4 to AOTVOKIR10 loci into the VIb clade (Figures 4-6). However, the OmKIR3DL3, OmKIR3DL4 and pOmKIRDL8 genes clustered independently to VIa and VIb in these trees, forming a third new lineage (VIc) (Figures 4-6). The topologies did not provide broad evidence of exon shuffling; however, recombination analysis showed recombinant fragments between loci (Figure 7). Most recombinant fragments were located on immunoglobulin domains and were large, indicating complete domain shuffling, although small recombinant fragments were found too. Nevertheless, domain shuffling does not seem to be the main mechanism responsible for creating new *Aotus* KIR receptors.

### Sequence divergence in paralogs, polymorphism and selection in *AOTVOKIR*

Nucleotide diversity (π) and Watterson estimator (θw) values were high when *A. vociferans* KIR loci were compared, showing a large divergence in paralogs (Table 1). The highest values were found in the D0 and D1 domains, whereas the D0b domain showed low paralogous sequence divergence (π = 0.008). Despite high KIR family paralogous divergence, individual loci showed low genetic polymorphism (Table 2). The most diverse locus was AOTVOKIR9 (π = 0.008) followed by AOTVOKIR1 and AOTVOKIR4, while the most conserved loci were AOTVOKIR2, AOTVOKIR3, AOTVOKIR8, and AOTVOKIR10 (π lower than 0.0001). 

**Table 1:** Domain by domain genetic sequence divergence in paralogs in the *A. vociferans* KIR family.

**N**	**Domain**	**Sites**	**Ss**	**θw (SD)**	**π (SD)**
31	D0a	279	83	0.074 (0.023)	0.097 (0.010)
8	D0b	282	4	0.005 (0.003)	0.008 (0.001)
33	D1	306	78	0.062 (0.020)	0.079 (0.004)
34	D2	294	63	0.052 (0.017)	0.051 (0.003)
5	STC-A	103	12	0.056 (0.031)	0.062 (0.018)
22	STC-I	106	26	0.067 (0.025)	0.042 (0.012)

n: Number of sequences, Sites: Total sites analyzed, Ss: Number of segregating sites, θ_W_: Watterson estimator, π: Nucleotide diversity. (SD): Standard deviation. The C-terminal region including stem, transmembrane and cytoplasmatic domain (STC) and split according to their function: STC-A = activator tail, STC-I = inhibitor tail

**Table 2:** Genetic polymorphism of *A. vociferans* KIR loci.

**n**	**Gene**	**Sites**	**Ss**	**NP**	**θw (SD)**	**π (SD)**
4	KIR1	1056	7	4(2^a^,2^b^)	0.004 (0.002)	0.004 (0.001)
2	KIR2	1278	1	3(2^a^,1^b^)	0.001 (0.001)	0.001 (0.000)
3	KIR3	908	1	4(3^a^,1^b^)	0.001 (0.001)	0.001 (0.000)
10	KIR4	1131	8	7(4^a^,3^b^)	0.002 (0.001)	0.002 (0.001)
1	KIR5	-	-	4(2^a^,2^b^)	-	-
1	KIR6	-	-	1(1^a^)	-	-
1	KIR7	-	-	2(1^a^,1^b^)	-	-
3	KIR8	1329	2	4(2^a^,2^b^)	0.001 (0.000)	0.001 (0.000)
3	KIR9	1233	14	4(2^a^,2^b^)	0.008 (0.005)	0.008 (0.003)
4	KIR10	948	1	5(1^a^,4^b^)	0.001 (0.001)	0.000 (0.000)

n: Number of sequences, Sites: Total of sites analyzed, Ss: Number of segregating sites, NP: Number of different encoded protein products (including ^a^ alleles and ^b^ splice variants) per locus, θ_W_: Watterson estimator, π: Nucleotide diversity. (SD): Standard deviation

The rate of non-synonymous (d_N_) and synonymous (d_S_) substitutions per site per domain (Table 3) was calculated to evaluate how natural selection was acting in the *A. vociferans* KIR family. A higher d_S_ rate than d_N_ was found; however, no statistical difference was found, except for the C-terminal region of inhibitor KIRs (Table 3). The slide window for ω (d_N_/d_S_) showed that codons could be differentially affected by natural selection (Figure S14). Different codon-based methods were used to estimate the ω ratio at every codon in the alignment. These methods found evidence of positive selection acting in different codons in the D0a, D1 and D2 domains as well as in the STC domain (Figure 1 and Table 3). Residues involved in the interaction with the MHC are also shown in Figure 1. Likewise, several codons were found under negative selection throughout these receptors (Figure 1). 

**Table 3:** Average number of synonymous substitutions per synonymous site (d_S_) and non-synonymous substitutions per non-synonymous site (d_N_) at each domain and individual codons affected by positive selection.

					**Positive selected sites**		
**Domain**	**d_N_ (SE)**	**d_S_ (SE)**	**FEL**	**REL**		**MEME**	**FUBAR**
D0a	0.103 (0.023)	0.113 (0.035)	39, 40, 52	39, 40, 52		32, 40, 52	25, 40
D0b	0.005 (0.004)	0.016 (0.012)	-	-		-	-
D1	0.081 (0.013)	0.102 (0.024)	244, 262. 266, 281	244, 262. 266,		229, 258, 262, 266, 277	254, 260, 262, 266
D2	0.050 (0.009)	0.066 (0.018)	314, 328, 330, 341	314, 328, 330, 341		314, 328, 330, 341, 345, 397	314, 328, 330
STC-A	0.047 (0.018)	0.158 (0.069)	429, 438, 439, 455	455		455	429
STC-I	0.033 (0.008)	0.069 (0.020)*					

No statistical differences were found between d_N_ and d_S_ substitution in Ig-like domains and STC-A domain or by SLAC method. Codon positions are according to the alignment shown in Figure 1. The C-terminal region including stem, transmembrane and cytoplasmatic domains (STC) was split according to their function: STC-A = activator tail and STC-I = inhibitor tail. (SE): Standard error. * p < 0.04.

## Discussion

Eight KIR gene models have been identified in previous analysis of a working draft sequence of the BAC clone from a hybrid *Aotus* monkey [15]. We have identified ten putative loci when characterizing *A. vociferans* KIR molecules from field samples, some of them having great similarity with KIR gene models. OmKIR3DS1 and OmKIR3DL2 were phylogenetically related to the AOTVOKIR1, AOTVOKIR2 and AOTVOKIR3 loci. However, other gene models were more phylogenetically distant, clustering outside the AOTVOKIR loci clades identified in this study (Figure S13), thereby suggesting rapid evolution in *Aotus*. Likewise, receptors having four Ig-like domains were characterized in this BAC clone analysis, such unusual KIR proteins being found in our cDNA analysis thus confirming the presence of these atypical receptors in *Aotus* species. Nevertheless, orthologs could not be found, since OmKIR4D gene models appeared phylogenetically distant to the AOTVOKIR-4D loci (Figure S13). Furthermore, the dual function KIR molecule (OmKIR4DL5) [15] was not found here. These atypical receptors are characterized by an extra D0 domain. This domain has been suggested to act as an ‘innate HLA sensor’ in humans since it extends towards the β2-microglobulin and a class I HLA region with limited polymorphism [21]. D0b might thus be an ‘innate MHC sensor’ for a species-specific MHC in *Aotus* monkeys. AOTVOKIR2DL1*01v2 and AOTVOKIR3DS3*03 were other atypical KIRs found in *A. vociferans*. The former have a rare Ig-like domain configuration. All reported KIR2D receptors have D0+D2 or D1+D2 configurations [7], but AOTVOKIR2DL1*01v2 had a D0+D1 configuration. Meanwhile, in AOTVOKIR3DS3*03 alleles, the exon 1 joined to nucleotide 18 of exon 2 changing the reading frame; taking into account that the start codon was not sequenced, this result could be due to either an alternative translation start site upstream or to a transcribed pseudogene. These features differed from other KIRs identified in primates; therefore, the KIR4DL loci and AOTVOKIR2DL1*01v2 receptor having a D0+D1 configuration could be exclusive of the *Aotus* genus. 

The KIR family has been reported to be highly diverse in all primates studied to date; our research has shown that this feature also seems to hold true in the *A. vociferans* KIR repertoire due the large sequence divergence in paralogs (Figure 1 and Table 1). Moreover, we detected multiple variants (1 to 4 in each locus) of AOTVOKIR molecules (as in humans and rhesus monkeys [10,22]) which appear to have generated by using alternative splice sites (Figures S11 and S12). This is a remarkable result since using alternative splice sites, *A. vociferans* could: (1) created new encoded receptors by deleting complete/incomplete Ig-like domains or just portions of the stem, transmembrane or cytoplasmatic domains thus increasing *A. vociferans* KIR diversity (although the biological significance of the alternative transcripts remains obscure) and (2) generate putative activating receptors in an unconventional way, seldom found in other primates. 

Soluble KIR receptors have also been reported in humans and rhesus monkeys [23,24]. A rhesus monkey allele lacking the stem and transmembrane domains, like the AOTVOKIR3DS3*03v1 allele, has been reported previously as a soluble KIR [22]. The AOTVOKIR4DS9*01v1 and AOTVOKIR3DS10*01v4 alleles had an intron insertion (Figure S12) generating a premature stop codon which produced a complete deletion of stem, transmembrane and cytoplasmatic domains, suggesting that these molecules are also secreted AOTVOKIRs. Even though soluble KIR receptors have been reported previously [23,24], their biological function is not fully understood. Other uncommon receptors generated by using alternative splice sites were found (AOTVOKIR3DS4*04v1 and AOTVOKIR3DS7*01v1) which could have been soluble as they had an unusual C-terminal region without a transmembrane helix, according to Phobius and TMHMM predictors. Given that AOTVOKIR3DS7 sequences were found only once, further analysis is necessary to determine whether they really are AOTVOKIRs. 

Similar to the human KIR2DS2*003 allele and the NKp80 stimulatory receptor, several short cytoplasmatic tail receptors (AOTVOKIR3DS1*01, AOTVOKIR3DS1*01v2, AOTVOKIR3/2DS5, AOTVOKIR3DS8*01v2 and AOTVOKIR4DS10*01v2 and AOTVOKIR3DS10*01v3) were found lacking a positively charged amino acid. Comparable behavior has been observed previously in cDNA sequences analysis (including only the exon 7 to exon 9) from a F1 hybrid *Aotus* monkey [15]. The deduced amino acid sequences from clone Om12A [15] and AOTVOKIR3/2DS5 had high similarity. The most important difference between them was that Om12A had a positively charged amino acid in the transmembrane domain while AOTVOKIR3/2DS5 did not. The Om32 clone, similar to AOTVOKIR3DS1*01, AOTVOKIR3DS1*01v2, AOTVOKIR3DS8*01v2, AOTVOKIR4DS10*01v2 and AOTVOKIR3DS10*01v3 did not have a positively charged amino acid, thus hampering their interaction with adapter molecules. However, other residues might contribute towards protein interactions within the plasma membrane [25]. In humans, proline 11 and threonine 13 are uniquely located and conserved within the transmembrane domains of activating KIRs [25]. Modifications at these residues have been shown to diminish association with DAP12 [25,26]. Since *A. vociferans* short cytoplasmatic tail receptors had these amino acids conserved at the same position (Figure 1), association between these AOTVOKIRs and adapter molecules might be facilitated by proline and theronine residues. However, short-tailed AOTVOKIR10 receptors do not have proline or theronine residues, so other amino acids should be involved in protein interactions. Alternatively, an unknown adapter molecule could have been coupling with these receptors. Functional studies are thus necessary to elucidate whether short-tailed AOTVOKIR5, AOTVOKIR8 and AOTVOKIR10 splice variants really are activating receptors. 

The *Aotus* KIR sequences were analyzed with Catarrhini receptors for evaluating their phylogenetic relationship within primates. A tree constructed with full-length nucleotide sequences showed that *Cercopithecidae*, *Hominidea* and Platyrrhini KIR genes clustered into separate clades (like species phylogeny), as previously reported [14,15]. Orthologous Catarrhini KIR2DL4 receptors were clustered outside these main clades lacking a Platyrrhini ortholog. Since KIR2DL4 loci might be the ancestral KIR, its absence in Platyrrhini suggested loss of ancestral locus in this parvorder. This clustering pattern also suggested these receptors’ independent divergence among higher primates. 

Two main clades were observed within the *Aotus* genus (Figure S13); similar behavior was found in domain by domain phylogenetic analysis (Figure 3 - Figure 6). Two monophyletic groups were established for the D0, D1, D2 and STC (stem, transmembrane and cytoplasmatic) domains. The former formed lineage VIa, clustering the AOTVOKIR1 to AOTVOKIR3, as well as OmKIR3DS1 and OmKIR3DL2 loci, while the second (lineage VIb) brought together the AOTVOKIR4 to AOTVOKIR10 with OmKIR4DL5, OmKIR4DL6 and pOmKIR4DL7 loci, whereas the OmKIR3DL3, OmKIR3DL4 and pOmKIRDL8 gene models was outside these clades, forming the VIc lineage. In addition to phylogenetic analysis, recombination analysis using RDP software showed recombination within and between lineages. These suggested that *Aotus* KIR family diversification, starting from at least two lineages and recombination between *A. vociferans* paralogs, may have produced new killer cell Ig-like receptors within them like other primates. Nevertheless, the main mechanism responsible for creating new *Aotus* KIR receptors seems to be alternative splicing and not domain shuffling.

According to Figure 3, the *Aotus* D0 duplicated domain (D0b) formed a paraphyletic group within lineage VIb; this extra domain therefore emerged from a KIR inside it. Furthermore, the OmKIR4DL6-D0b domain was phylogenetically closest to AOTVOKIR4 showing a relatively low genetic distance between them (Table S2). The remaining D0b domains formed a sister clade with AOTVOKIR8 and the genetic distance between these D0b domains and AOTVOKIR8 was among the lowest (Table S2). This suggested that KIR4D receptors emerged from two independent duplication events. The origin of the D0b domain still remains not fully clear. 

It is thought that KIR family evolution can be explained by a birth-and-death model [27,28] where loci evolve independently. Individual KIR loci sequences remain remarkably conserved, while KIR haplotypes have evolved to produce a highly diverse family of receptors. The π and θ_W_ values found when compared AOTVOKIR paralogous domains (Table 1) supports this idea and, like human KIRs, individual AOTVOKIR loci show low intragenic polymorphism. Although the small number of sequences could bias the low intragenic polymorphism, the most polymorphic locus here found was AOTVOKIR9 (π = 0.008, n = 3) while the AOTVOKIR4 had a low π value (π = 0.002, n = 10), suggesting that the low polymorphism might be a feature of AOTVOKIRs loci. Table S1 shows that each individual has a different haplotype, however, this behavior could be due to the limitations of our approach that does not allow identifying the full number of genes in each individual.

The mechanics by which KIR loci evolved towards a greatly diverse (paralogous divergence) family is natural selection [16,29,30]. No differences were found when the d_N_ and d_S_ rates were calculated within *A. vociferans* Ig-like KIR domains. However, the domain by domain ω sliding window (Figure S12) showed evidence of positive and negative selection in particular regions; therefore, natural selection might have varied across codons. This was confirmed by using codon-based methods which led to estimating the d_N_/d_S_ (ω) ratio at every codon in the alignment. This analysis showed several sites under negative selection throughout the KIR alignment, whereas positive selection acted on individual sites in D0a, D1 and D2 domains as well as on stem and cytoplasmatic domains (Figure 1). This data suggested that both constraint and rapid evolution may operate within the KIR family. Thus, negative selection constricts functionally or structurally important regions (as the inhibitory cytoplasmatic domain, which displayed 9 negatively selected codons and a d_S_ > d_N_ with a p < 0.04), while positive selection could diversify the residues within domains that are involved in inter-domain interaction [16,29] or are involved in pathogen recognition [30]. Sites in AvKIR D1 and D2 domains, homologous to those involved in KIR-HLA interactions [21,31,32] or sites close to them, were under positive selection and thus, natural selection could be favoring changes that allow the recognition of different MHC-I molecules. Likewise, leucine 166 of the human KIR3DL1*001, involved in direct contact with a self-peptide presented on the HLA-B*5701 [21], and the homologous AvKIR residue (localized in position 281), were under positive selection; natural positive selection could thus allow diversification of amino acids interacting with the peptide being presented on class I. However, it is not clear why positive selection is acting in the stem and cytoplasmatic domain. 

According the data reported here, *A. vociferans* KIR family evolved from at least two lineages which gave rise to 10 putative loci; the high AvKIR diversity would therefore be mainly due: (1) extensive sequence divergence in paralogs generated by natural selection and (2) alternative splicing producing multiple receptor variants. Furthermore, this mechanism allows loci to encode both short and long cytoplasmatic receptors.

## Materials and Methods

### Ethics statement, animal capture and study area

The present study was approved by the Fundación Instituto de Inmunología's ethics committee. The capture of eleven *Aotus vociferans* primates (International Union for Conservation of Nature and Natural Resources (IUCN) status: least concern) was authorized by the official Colombian environmental authority, CORPOAMAZONIA, for this primate's capture, study, and scientific research in the Amazonian region (CORPOAMAZONIA, resolutions 0066/Sep/2006, 0028/May/2010 and 0632/Jun/2010 and previous authorizations beginning in 1982). All animal-handling procedures were carried out according to the Guide for the Care and Use of Laboratory Animals, USA [33]; such recommendations comply with Colombian regulations for biomedical research (resolution 8430/1993 and law 84/1989). Captured individuals were numbered, sexed, weighed, given a physical-clinical examination and temporally housed in individual cages measuring 50 x 50 x 60 centimeters, prior to any experimental procedure. The monkeys were then kept in cages measuring 1 x 1.5 x 2 meters. In both cases, monkeys were kept in similar temperature (25 to 30 degrees centigrade) and relative humidity (83%) conditions to those found in their natural environment. The monkeys’ diet was based on a supply of typical fruits from the amazon region (based on such primates’ natural diet), vegetables and a nutritional supplement including vitamins, minerals and proteins. Their environment was enriched by including visual barriers (to avoid social conflicts), feeding devices, some branches and vegetation, perches and a nest box. All procedures requiring the animals to be handled were carried out by trained veterinary personnel; when necessary the animals were sedated and given analgesia to reduce stress. CORPOAMAZONIA made a weekly visit to evaluate housing conditions, feeding regimens and the environmental enrichment of the monkeys captured. Blood samples were taken from individuals with no kinship, collected within the authorized Amazonian territory in different indigenous communities, as follows: La Libertad (4 individuals), Los Lagos (2 individuals), Naranjales (1 individual), Macedonia (1 individual), San Francisco (1 individual), Siete de Agosto (1 individual) and El Vergel (1 individual). The monkeys were supervised by veterinarians and biologists; all individuals were released back into the Amazon jungle after the experimental procedures in optimal health conditions in the presence of a CORPOAMAZONIA representative.

### DNA, RNA extraction and cDNA synthesis

We collected 3 mL peripheral blood samples from eleven monkeys phenotypically consistent with *Aotus vociferans*. DNA was obtained from 300 µL total blood using a Wizard Genomic DNA purification kit (Promega), following the manufacturer’s instructions. White blood cells were obtained from density gradient separation. Total cellular RNA was isolated from peripheral blood mononuclear cells using the TRIzol one-step procedure (Invitrogen Life Technologies, CA, USA). A SuperScript III reverse transcriptase kit (Invitrogen Life Technologies, CA, USA) was used for cDNA synthesis, according to the manufacturer’s instructions.

### COII gDNA and KIR cDNA amplification

A fragment from the cytochrome oxidase subunit II (COII) gene was amplified using COII-Asp 5’-ACCATTCATAACTTTGTCAA-3’ and COII-Lys 5’-CTCTTAATCTTTAACTTAAAAG-3’ primers to determinate whether field sampled monkeys were actually *A. vociferans*. The reaction mixture contained 2.5 µL enzyme buffer, 1 µL MgCl_2_ [50 mM], 0.5 µL dNTPs [25 mM], 2.5 µL of each primer [5µM], 0.5 µL ACCUZYME DNA polymerase (BIOLINE) and 10–40 ng DNA template in a final 25 µL volume. Thermal conditions were set as follows: one cycle at 98°C for 5 min, 35 cycles at 55°C for 1 min, 1.3 min at 72°C, 1 min at 95°C and a final 1 min cycle at 55°C, followed by a 5 min final extension step at 72°C. Two independent PCR were conducted and amplification fragments were purified with Wizard SV gel and PCR clean-up system. *A. vociferans* cDNA KIR was amplified using KAPA HiFi HotStart Readymix containing 0.3 mM of each primer (KIRF: 5’-GTCACTCATGGTCATCAGC-3’ and KIRR: 5’-TCATGGACAGGAGACAAC-3’) in a final 25 µL volume in an approach aimed at reducing PCR artifacts [34-36]. Thermal conditions were set as follows: one cycle of 5 min at 95°C, 28 cycles of 20 sec at 98°C, 15 sec at 60°C, and 30 sec at 72°C, followed by a 5 min final extension at 72°C. The amplicons were purified just like the COII PCR fragments and an A-tailing protocol with GoTaq DNA polymerase (Promega) was used for ligating them, using a pGEM T-Easy Vector System (Promega), and they were then cloned into *Escherichia coli* JM109 cells. Positive clones were selected using ampicillin-positive selection and α-complementation of the lacZ gene. Sixteen plasmid DNAs were extracted using a Wizard plus Minipreps kit (Promega) per individual. The BigDye capillary electrophoresis method, using ABI-3730 XL (Macrogen, Seoul, South Korea) in both directions with amplification primers and T7 and SP6 primers, was used for DNA sequencing of COII PCR fragments and plasmids, respectively. *A. vociferans* KIR sequences found at least twice after sequencing several clones or present in two or more individuals are available in GenBank: accession numbers KF014088-KF014122.

### Phylogenetic analysis for species identification and KIR sequences

Electropherograms were assembled using CLC main workbench software (CLC bio, Cambridge, MA, USA). The amplified *Aotus* primate COII fragments (Aovos) were compared with reported COII sequences for the *Aotus* genus (GenBank access numbers: HQ005483.1, AF352260.1, DQ321665.1, DQ321666.1, DQ321669.1, DQ321660.1, DQ321659.1, JN161054.1, JN161050.1, AF352255.1, HQ005486.1, HQ005485.1, DQ321670.1, HQ005478.1, AF352257.1, DQ321663.1, DQ321667.1, HQ005474.1, HQ005473.1, HQ005472.1, JF735191.1 and U36848.1). Clustal W [37] was used for aligning all COII sequences. MEGA software [38] was used for selecting the best nucleotide substitution model using Bayesian information criteria (BIC), screening 24 different nucleotide substitution models and a phylogenetic tree was then generated with the maximum likelihood (ML) method. 

*A. vociferans* KIR sequences were manually aligned and a phylogenetic tree was then inferred using the ML method with Kimura’s two parameter (K2) [39] and a discrete Gamma distribution (*+G*) model selected by ML and BIC criteria. Domain by domain phylogenetic analyses were performed with sequences from Catarrhini (*Homo sapiens*, *Pan troglodytes*, *Gorilla gorilla*, *Pongo pygmaeus*, and *Macaca mulatta*) and Platyrrhini (*Aotus* *sp* and *A. vociferans*) primates, selecting the model that best fit the data. Maximum likelihood phylogenetic analyses were performed with MEGA software [38]. Bootstrap analysis (with 1,000 replicates each) was used for assigning confidence levels to branch nodes in all topologies. All positions having less than 95% site coverage were eliminated.

### Genetic diversity and natural selection analyses

DnaSP software [40] was used to evaluate the genetic diversity of the *A. vociferans* KIR family (sequence divergence in paralogs) as well as each locus (polymorphism), positions containing gaps being removed. Natural selection at individual domain was assessed with MEGA v.5 [38], computing non-synonym substitution per non-synonym site (d_N_) and synonym substitution per synonym site (d_S_) rate using the modified Nei-Gojobori method [41]. Differences between d_N_ and d_S_ were assessed by applying the codon-based Z-test included in MEGA v.5. Positive and negative selection at individual sites was evaluated with SLAC, FEL, REL, MEME and FUBAR methods [42-45], taking recombination into account. A ≤ 0.1 p-value was considered significant for SLAC, FEL and MEME methods; ≥ 0.9 posterior probability was significant for FUBAR and ≥ 50 Bayes factor for REL. RDP3 v.3.4 software [46] was used for detecting recombination among paralogous genes, ignoring positions containing gaps.

## Supporting Information

Figure S1 ***Aotus* genus phylogenetic tree based on cytochrome oxidase subunit II (COII).** The ML topology was inferred from the HKY*+I* evolutionary model. The gray-necked *Aotus* group and the red-necked *Aotus* group were label in gray and red, respectively. AOTVO01 - 11: field sampled *A. vociferans* individuals included in this study. COII sequences from *Cebus capucinus* and *Saimiri sciureus* were used as out-group. Numbers on branches correspond to Bootstrap values.
(TIF)

Figure S2 **Deduced AOTVOKIR1 amino acid sequence alignment.** Dots (.) indicate identity among AOTVOKIR sequences, dashes (-) indicate absence of amino acids.
(TIF)

Figure S3 **Deduced AOTVOKIR2 amino acid sequence alignment.** Dots (.) indicate identity among AOTVOKIR sequences, dashes (-) indicate absence of amino acids.
(TIF)

Figure S4 **Deduced AOTVOKIR3 amino acid sequence alignment.** Dots (.) indicate identity among AOTVOKIR sequences, dashes (-) indicate absence of amino acids.
(TIF)

Figure S5 **Deduced AOTVOKIR4 amino acid sequence alignment.** Dots (.) indicate identity among AOTVOKIR sequences, dashes (-) indicate absence of amino acids.
(TIF)

Figure S6 **Deduced AOTVOKIR5 amino acid sequence alignment.** Dots (.) indicate identity among AOTVOKIR sequences, dashes (-) indicate absence of amino acids.
(TIF)

Figure S7 **Deduced AOTVOKIR7 amino acid sequence alignment.** Dots (.) indicate identity among AOTVOKIR sequences, dashes (-) indicate absence of amino acids.
(TIF)

Figure S8 **Deduced AOTVOKIR8 amino acid sequence alignment.** Dots (.) indicate identity among AOTVOKIR sequences, dashes (-) indicate absence of amino acids.
(TIF)

Figure S9 **Deduced AOTVOKIR9 amino acid sequence alignment.** Dots (.) indicate identity among AOTVOKIR sequences, dashes (-) indicate absence of amino acids.
(TIF)

Figure S10 **Deduced AOTVOKIR10 amino acid sequence alignment.** Dots (.) indicate identity among AOTVOKIR sequences, dashes (-) indicate absence of amino acids.
(TIF)

Figure S11 **Alternative splicing on lineage VIa.** Alignment of a putative locus from an *A. nancymaae* - *A. azarai* hybrid owl monkey (obtained from a BAC clone) and the putative *A. vociferans* exons in loci belonging to lineage VIa. Arrows indicate the putative acceptor and donor sites.
(PDF)

Figure S12 **Alternative splicing on lineage VIb.** Alignment of a putative locus from an *A. nancymaae* - *A. azarai* hybrid owl monkey (obtained from a BAC clone) and the putative *A. vociferans* exons in loci belonging to lineage VIb. Arrows indicate the putative acceptor and donor sites.
(PDF)

Figure S13 **Full-length maximum likelihood phylogenetic tree from nucleotide sequences of higher primate KIR genes.** Branches in red represent *Cercopithecidae* KIR, green *Hominidea* KIR and blue branches combine Platyrrhini KIR. Ancestral KIRs are surrounded by a black curved line. Numbers on branches represent bootstrap values after 1,000 replicates. Human allele sequences have no prefix; GORGO, *Gorilla gorilla*; PANTR, *Pan troglodytes*; PONPY, *Pongo pygmaeus*; MACMU, *Macaca mulatta*; Om, *Aotus* sp and AOTVO, *A. vociferans*.
(TIF)

Figure S14 **Domain by domain sliding window analysis for ω rates (d_N_/d_S_).** Values above 1 indicate positive selection and below 1 indicate negative selection.
(TIF)

Table S1 **Alleles and splice variants identified in 10 putative AOTVOKIR loci.** Alleles were named analogously to the IPD-KIR database. Each putative locus, allele and splice variant carries the number of Ig-like domains and the letter L for a long cytoplasmic tail or S for a short cytoplasmic tail. A number (after the letter representing tail length) was added (according to the phylogenetic tree shown in Figure 2) to differentiate sequences from each putative locus. Two digits are used to indicate alleles that differ in their encoded protein sequences. The variants generated by alternative splicing are designed by the letters “v” and a number.
(XLS)

Table S2 **Estimates of evolutionary genetic distance between D0 domain sequences.** The number of base pair substitutions per site between sequences are shown. Standard errors are shown in every other column and were obtained by a bootstrap procedure (1,000 replicates). Analyses were conducted using the K2 *+G* model.
(XLS)
