# Supplementary figures and images for: Identification and Diversity of Killer Cell Ig-Like Receptors in Aotus vociferans, a New World Monkey
Source: PLoS One. 2013 Nov 6;8(11):e79731. doi: 10.1371/journal.pone.0079731 (PMC3819253; doi:10.1371/journal.pone.0079731)

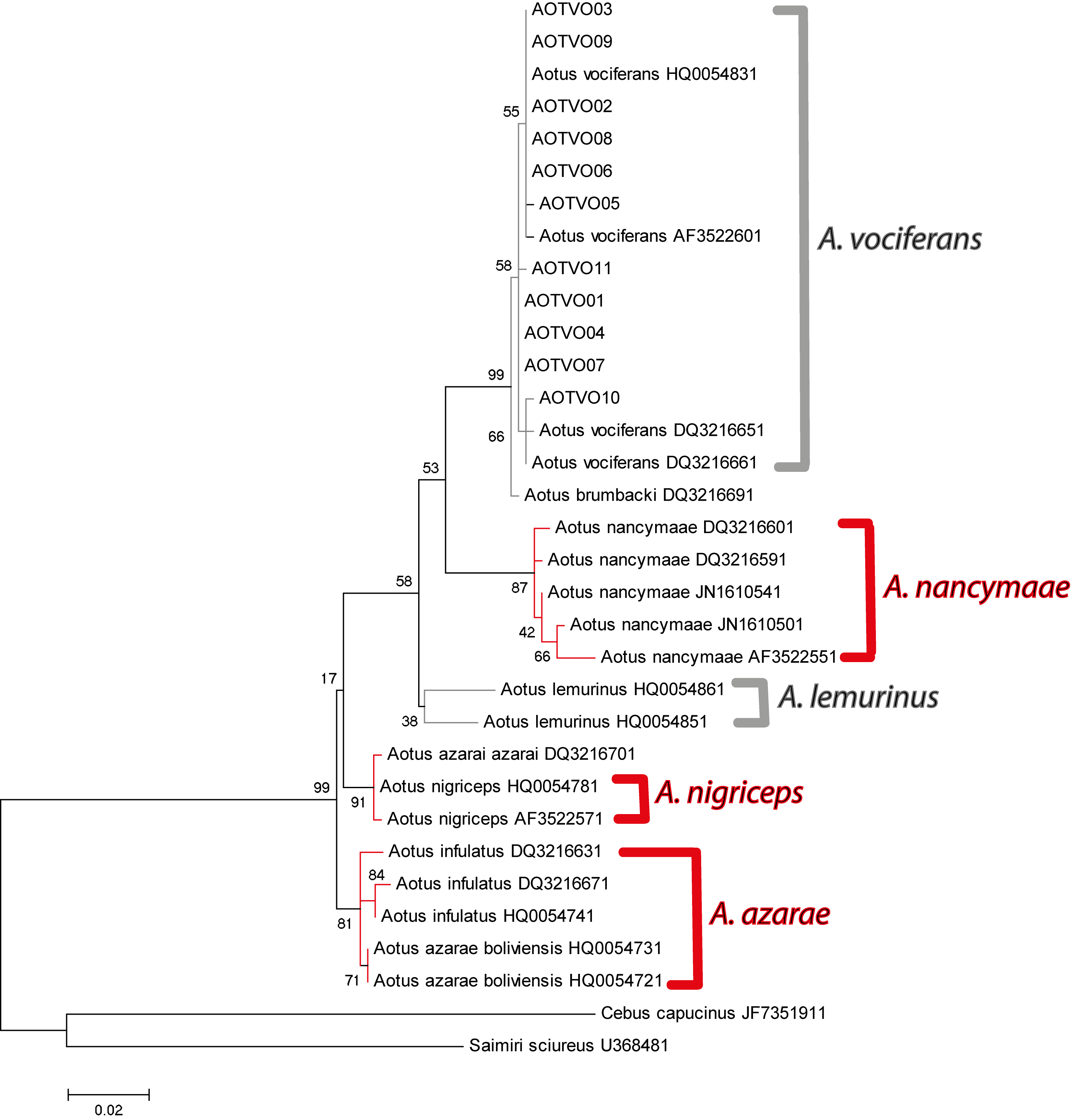

Supplement: Figure S1 — Aotus genus phylogenetic tree based on cytochrome oxidase subunit II (COII). The ML topology was inferred from the HKY+I evolutionary model. The gray-necked Aotus group and the red-necked Aotus group were label in gray and red, respectively. AOTVO01 - 11: field sampled A. vociferans individuals included in this study. COII sequences from Cebus capucinus and Saimiri sciureus were used as out-group. Numbers on branches correspond to Bootstrap values. (TIF) [file pone.0079731.s001.tif]

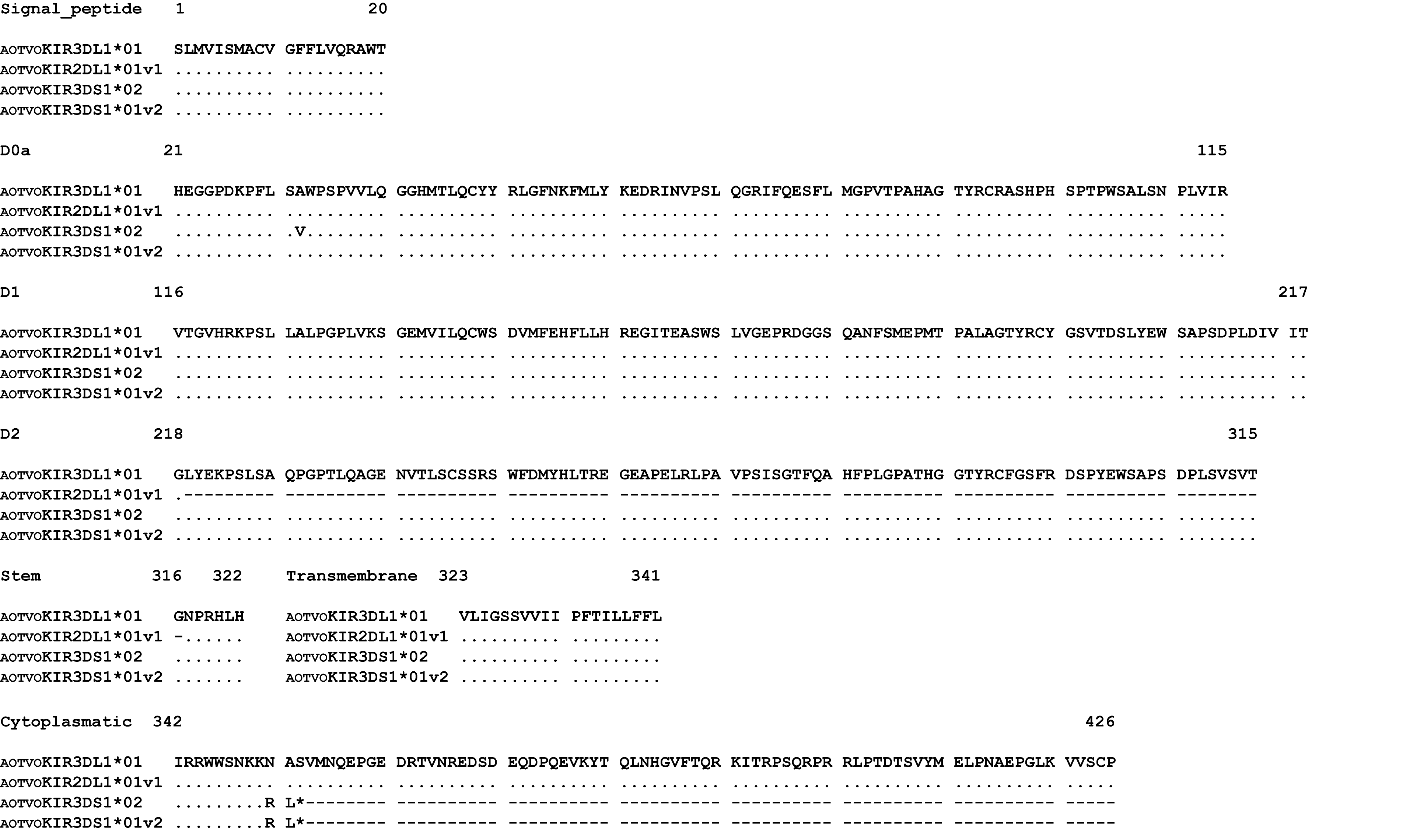

Supplement: Figure S2 — Deduced AOTVOKIR1 amino acid sequence alignment. Dots (.) indicate identity among AOTVOKIR sequences, dashes (-) indicate absence of amino acids. (TIF) [file pone.0079731.s002.tif]

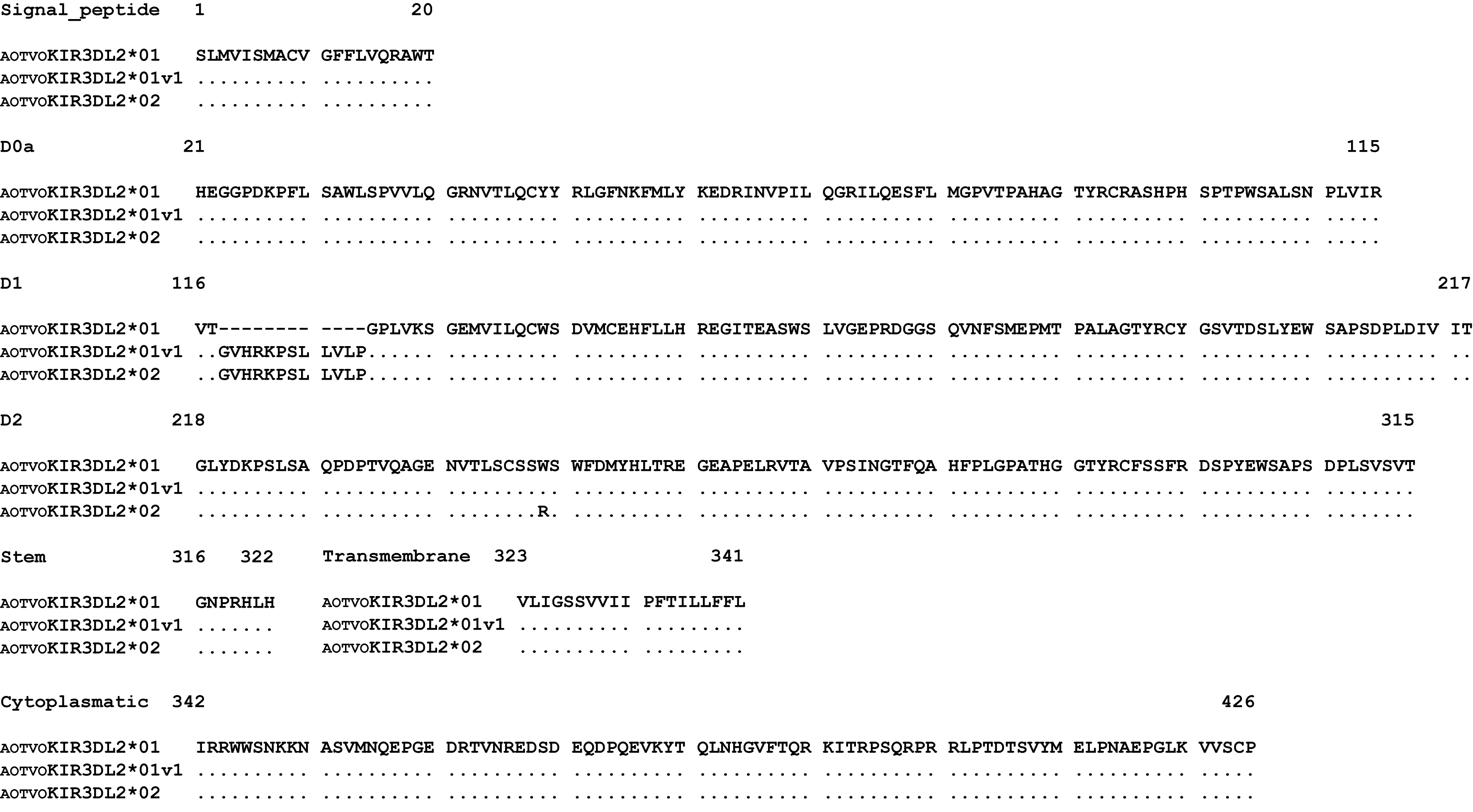

Supplement: Figure S3 — Deduced AOTVOKIR2 amino acid sequence alignment. Dots (.) indicate identity among AOTVOKIR sequences, dashes (-) indicate absence of amino acids. (TIF) [file pone.0079731.s003.tif]

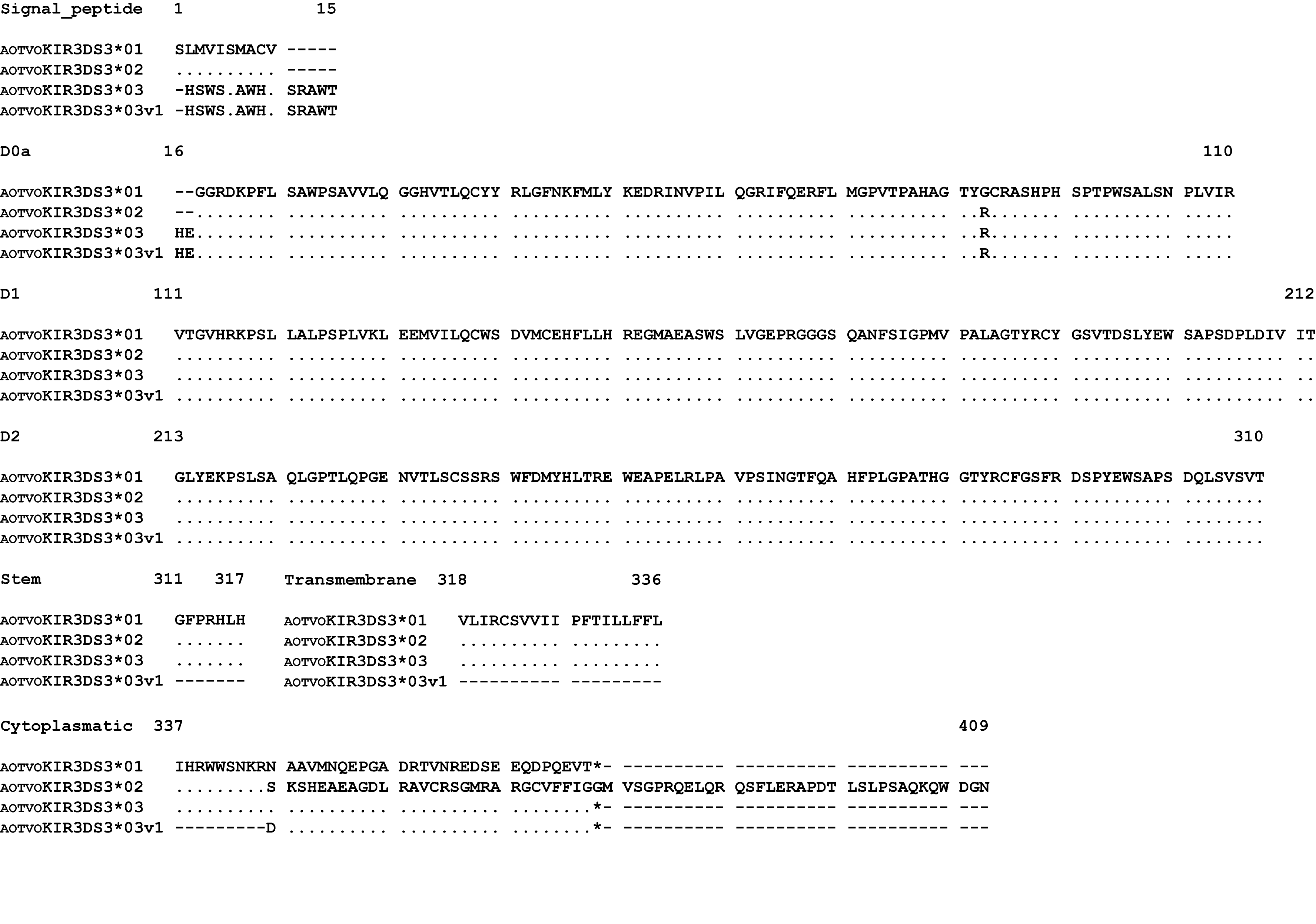

Supplement: Figure S4 — Deduced AOTVOKIR3 amino acid sequence alignment. Dots (.) indicate identity among AOTVOKIR sequences, dashes (-) indicate absence of amino acids. (TIF) [file pone.0079731.s004.tif]

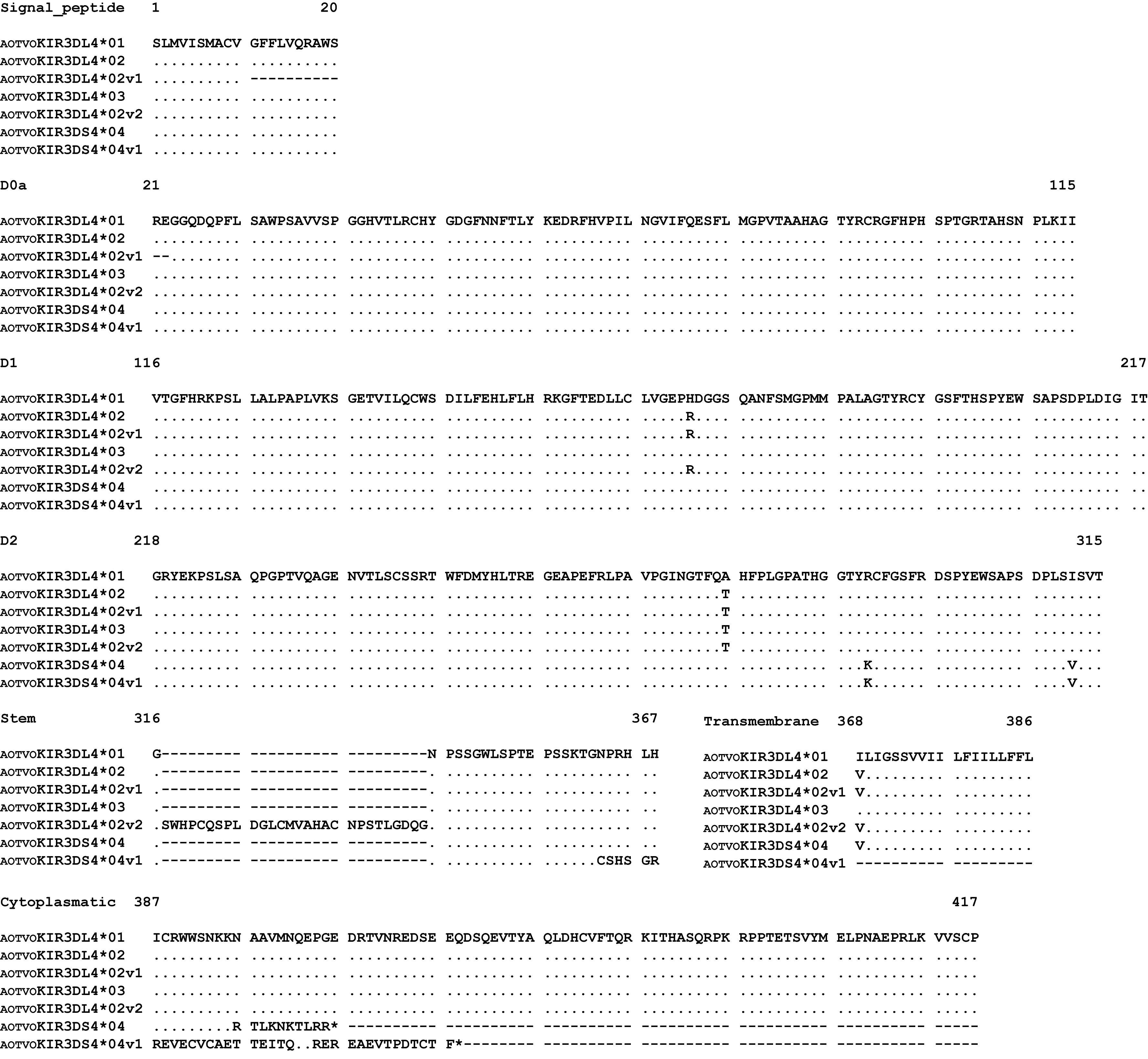

Supplement: Figure S5 — Deduced AOTVOKIR4 amino acid sequence alignment. Dots (.) indicate identity among AOTVOKIR sequences, dashes (-) indicate absence of amino acids. (TIF) [file pone.0079731.s005.tif]

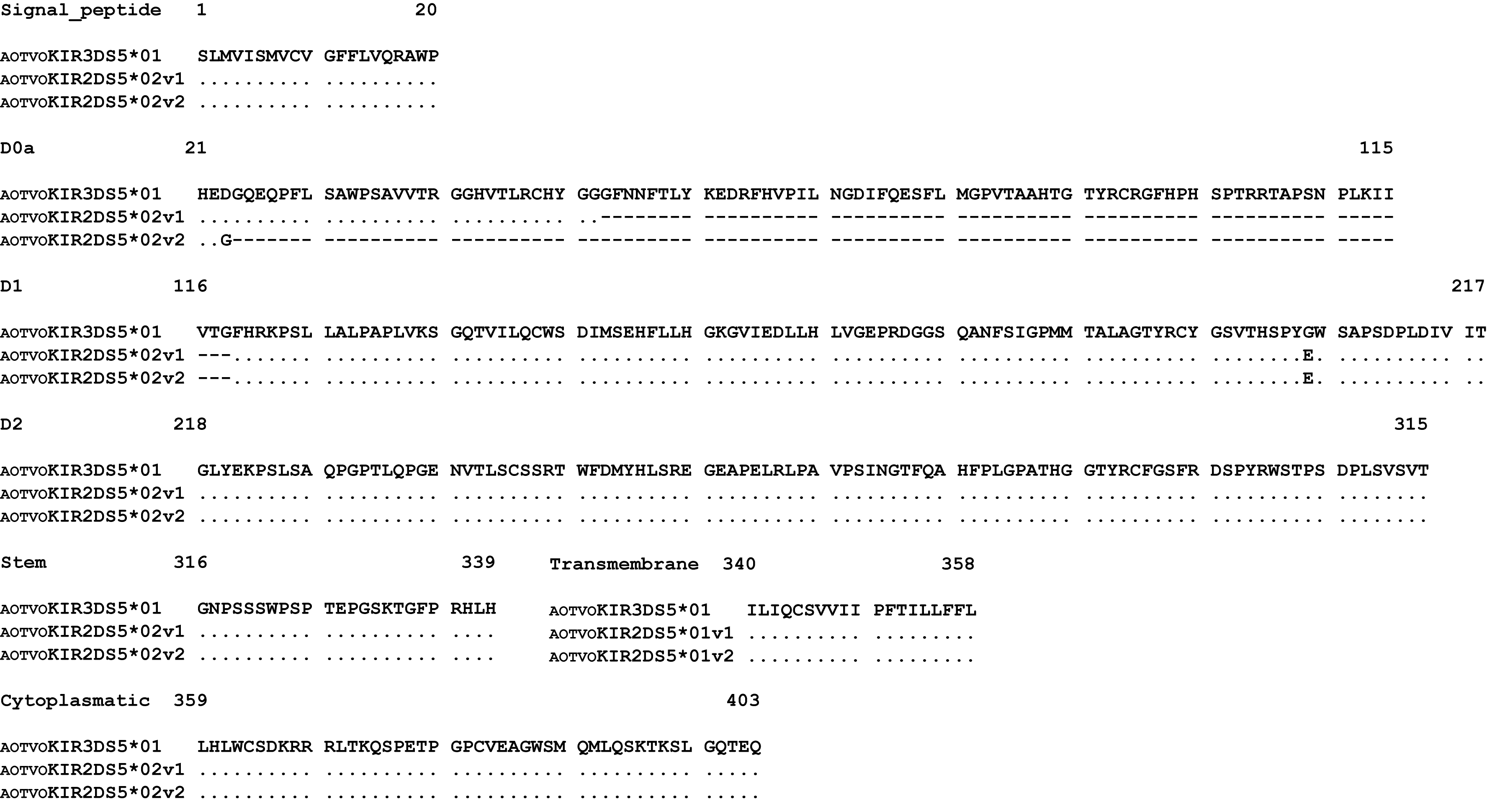

Supplement: Figure S6 — Deduced AOTVOKIR5 amino acid sequence alignment. Dots (.) indicate identity among AOTVOKIR sequences, dashes (-) indicate absence of amino acids. (TIF) [file pone.0079731.s006.tif]

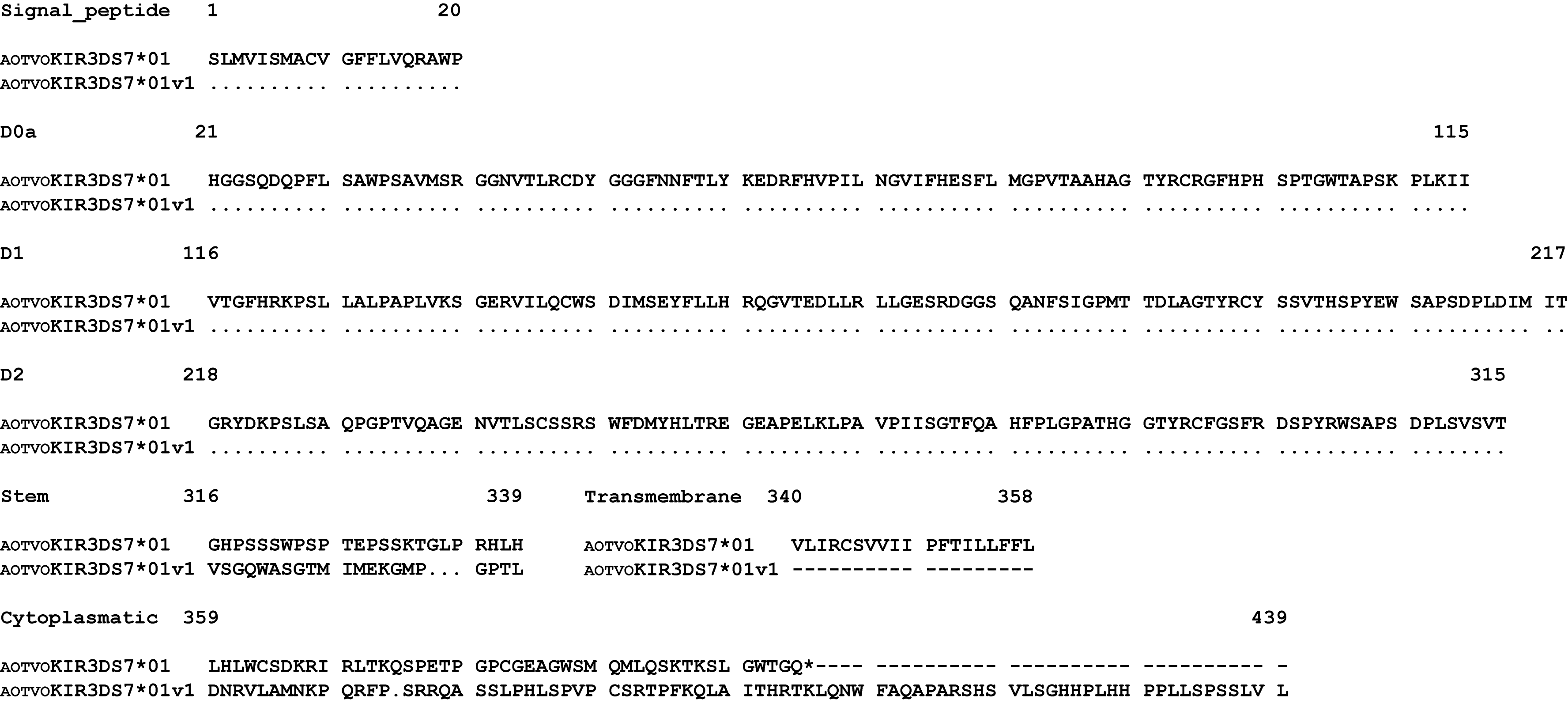

Supplement: Figure S7 — Deduced AOTVOKIR7 amino acid sequence alignment. Dots (.) indicate identity among AOTVOKIR sequences, dashes (-) indicate absence of amino acids. (TIF) [file pone.0079731.s007.tif]

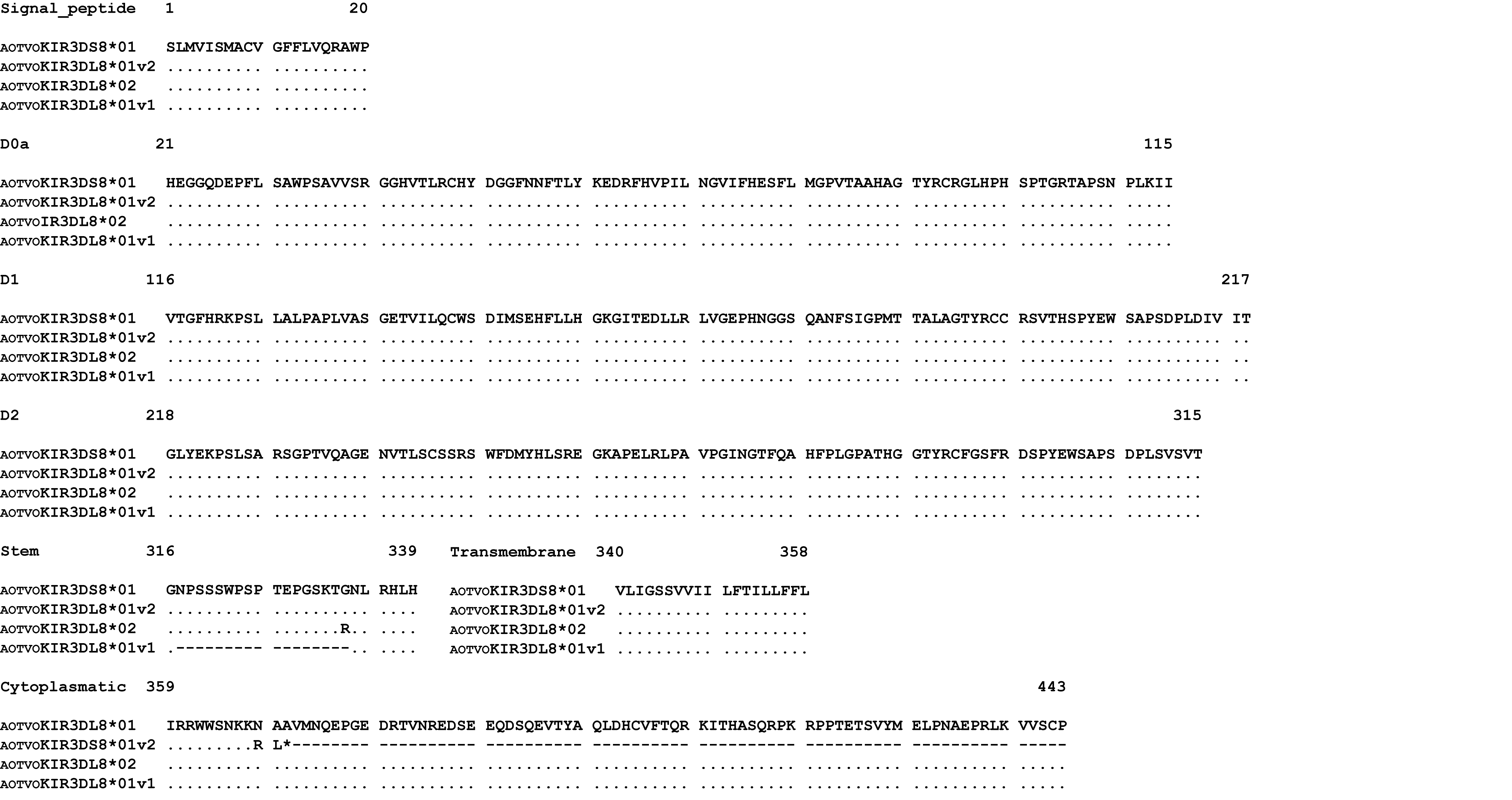

Supplement: Figure S8 — Deduced AOTVOKIR8 amino acid sequence alignment. Dots (.) indicate identity among AOTVOKIR sequences, dashes (-) indicate absence of amino acids. (TIF) [file pone.0079731.s008.tif]

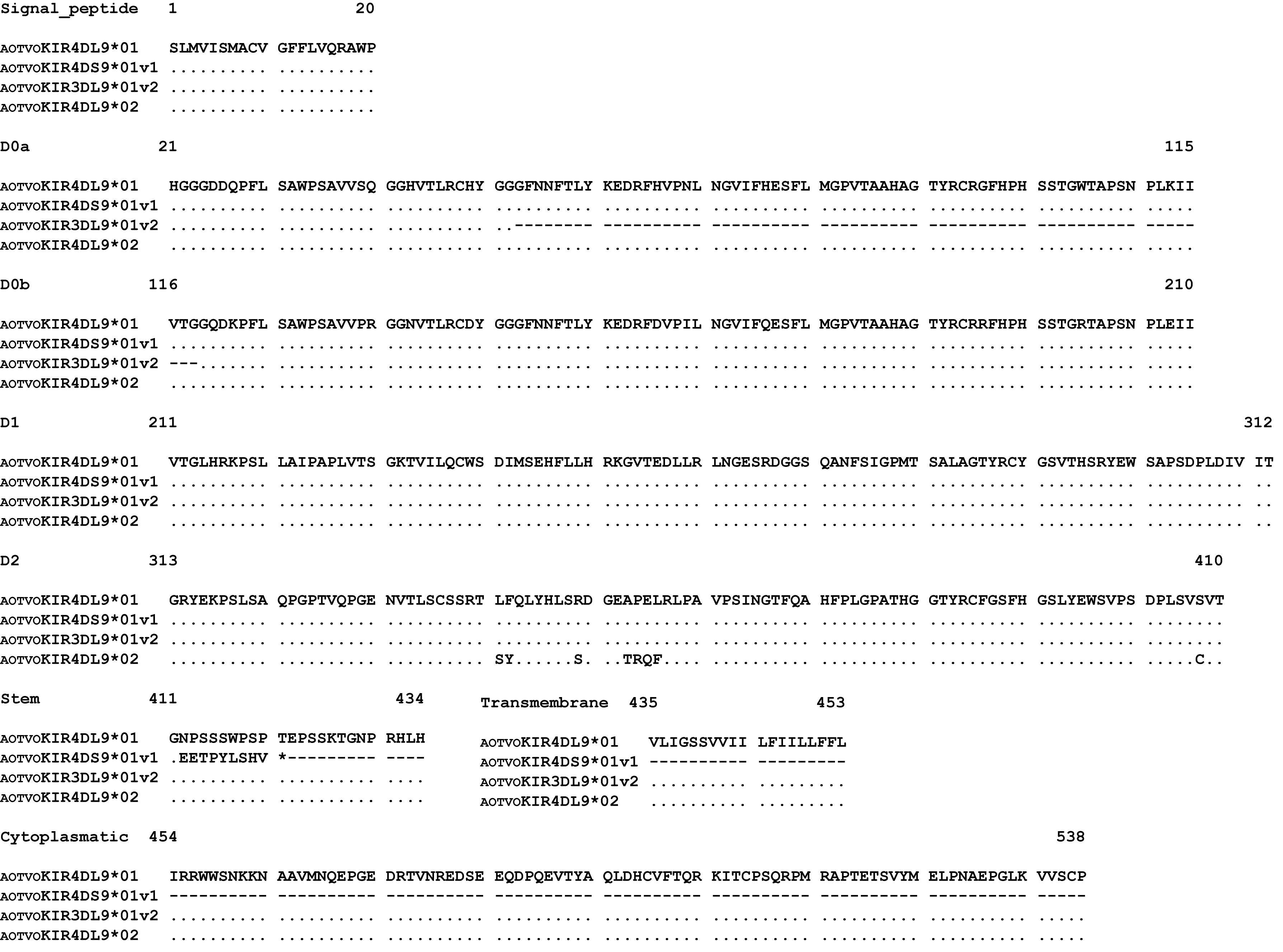

Supplement: Figure S9 — Deduced AOTVOKIR9 amino acid sequence alignment. Dots (.) indicate identity among AOTVOKIR sequences, dashes (-) indicate absence of amino acids. (TIF) [file pone.0079731.s009.tif]

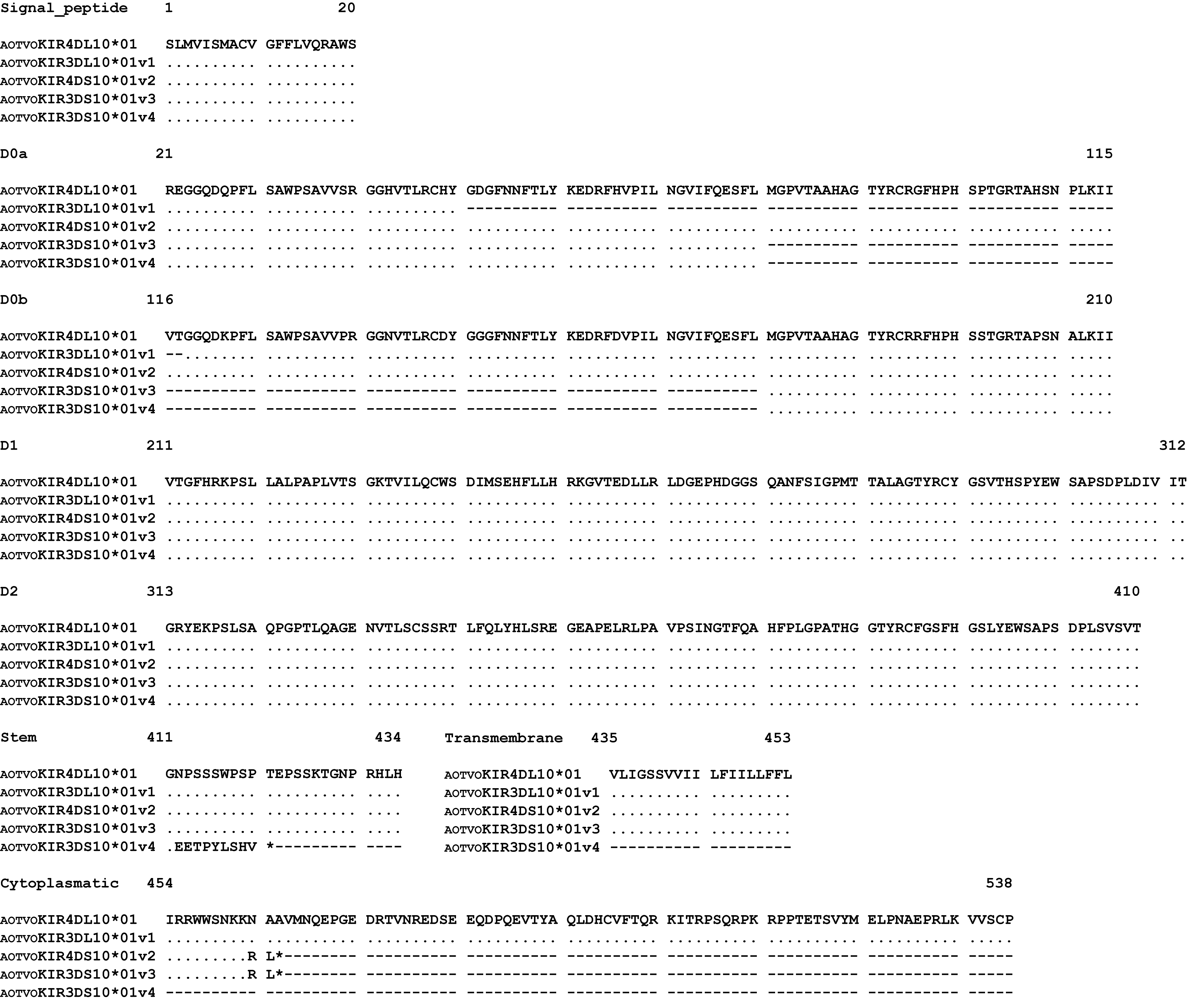

Supplement: Figure S10 — Deduced AOTVOKIR10 amino acid sequence alignment. Dots (.) indicate identity among AOTVOKIR sequences, dashes (-) indicate absence of amino acids. (TIF) [file pone.0079731.s010.tif]

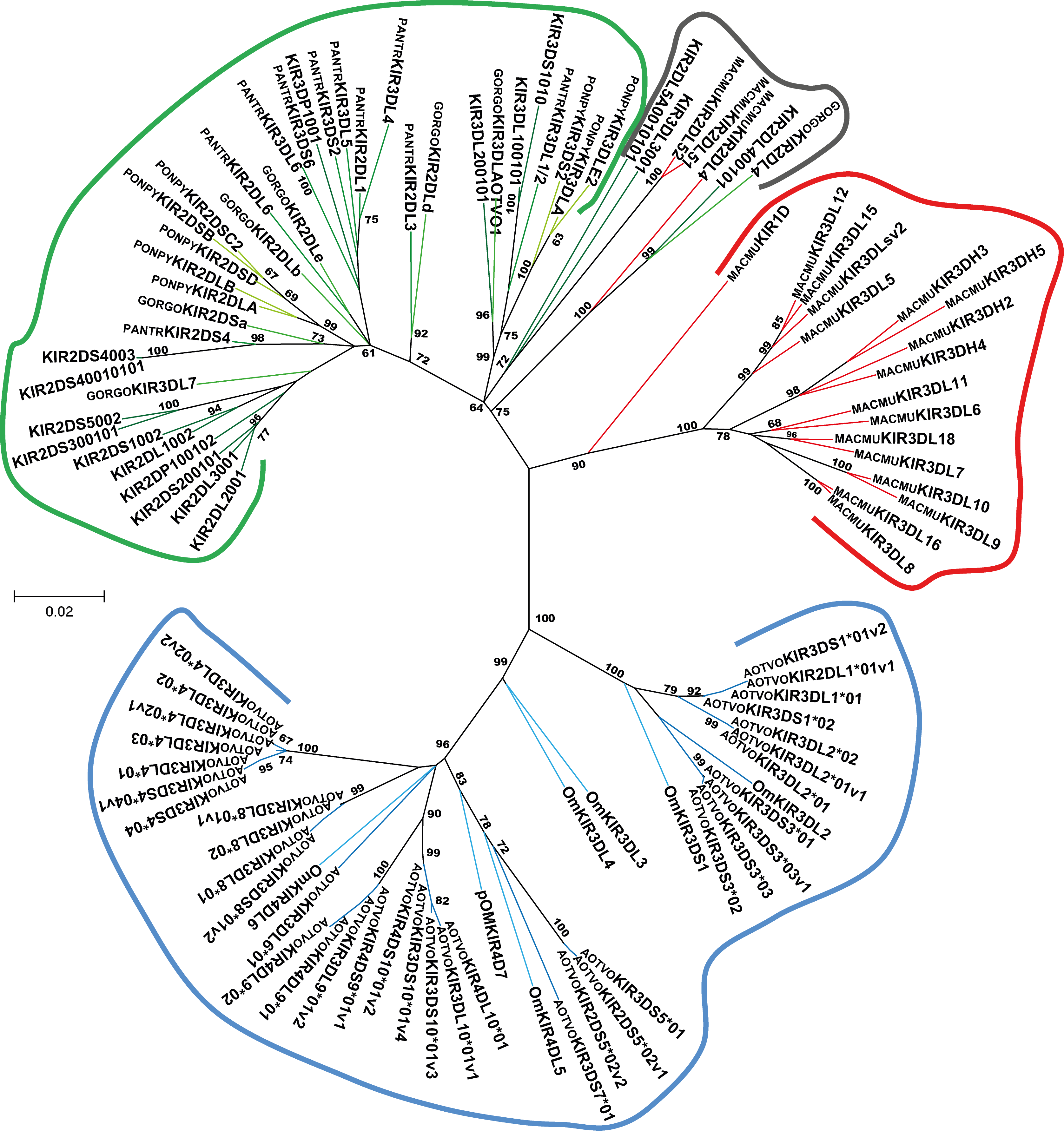

Supplement: Figure S13 — Full-length maximum likelihood phylogenetic tree from nucleotide sequences of higher primate KIR genes. Branches in red represent Cercopithecidae KIR, green Hominidea KIR and blue branches combine Platyrrhini KIR. Ancestral KIRs are surrounded by a black curved line. Numbers on branches represent bootstrap values after 1,000 replicates. Human allele sequences have no prefix; GORGO, Gorilla gorilla; PANTR, Pan troglodytes; PONPY, Pongo pygmaeus; MACMU, Macaca mulatta; Om, Aotus sp and AOTVO, A. vociferans. (TIF) [file pone.0079731.s013.tif]

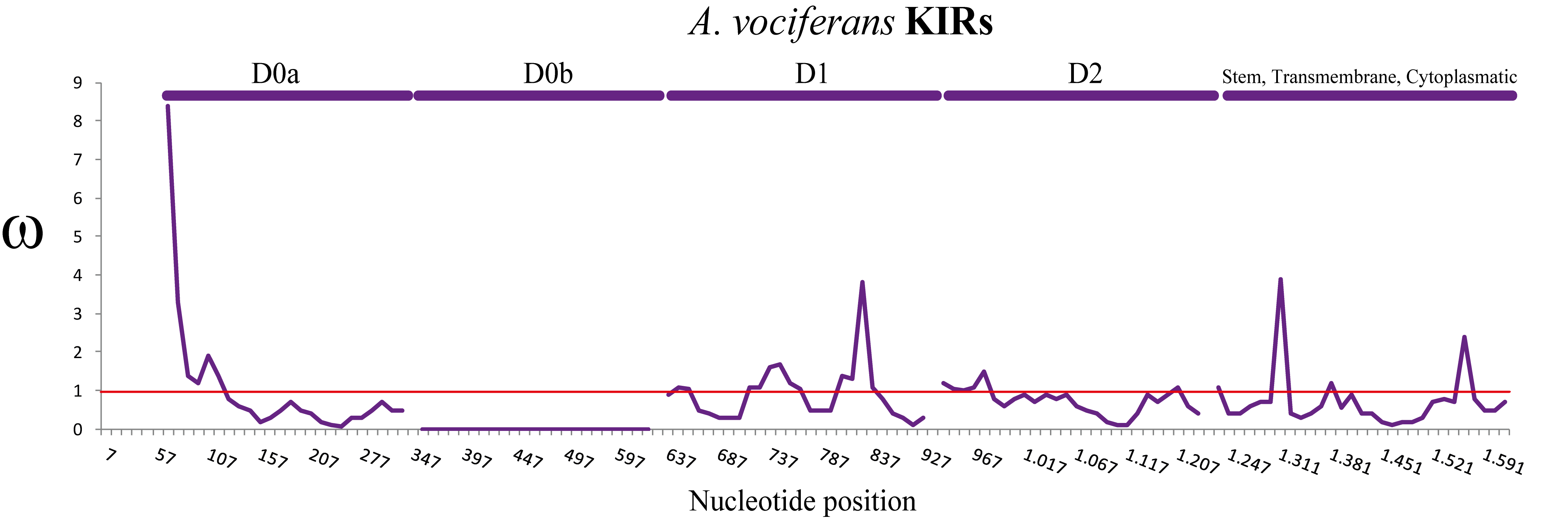

Supplement: Figure S14 — Domain by domain sliding window analysis for ω rates (dN/dS). Values above 1 indicate positive selection and below 1 indicate negative selection. (TIF) [file pone.0079731.s014.tif]
