# Supplementary material for: Identification and Diversity of Killer Cell Ig-Like Receptors in Aotus vociferans, a New World Monkey
Source: PLoS One. 2013 Nov 6;8(11):e79731. doi: 10.1371/journal.pone.0079731 (PMC3819253; doi:10.1371/journal.pone.0079731)
Supplement: Figure S11 — Alternative splicing on lineage VIa. Alignment of a putative locus from an A. nancymaae - A. azarai hybrid owl monkey (obtained from a BAC clone) and the putative A. vociferans exons in loci belonging to lineage VIa. Arrows indicate the putative acceptor and donor sites. (PDF) [file pone.0079731.s011.pdf]

EXON 1

```
                                   *        20         *        40         *        60         *        80         *
BAC_clone_Om        : ATGTCACTCATGGTCATCAGCATGGCATGTGTCGGTGAGTCCTGGAGGGAATCCAGGGGGGAGCGATGGGGTGGGGATCCCAGCCTGGAGCGGAGA :  96
AOTVOKIR3DL1*01     : ---TCACTCATGGTCATCAGCATGGCATGTGTCG-------------------------------------------------------------- :  31
AOTVOKIR2DL1*01v1   : ---TCACTCATGGTCATCAGCATGGCATGTGTCG-------------------------------------------------------------- :  31
AOTVOKIR3DS1*01v2   : ---TCACTCATGGTCATCAGCATGGCATGTGTCG-------------------------------------------------------------- :  31
AOTVOKIR3DS1*02     : ---TCACTCATGGTCATCAGCATGGCATGTGTCG-------------------------------------------------------------- :  31
AOTVOKIR3DL2*01     : ---TCACTCATGGTCATCAGCATGGCATGTGTCG-------------------------------------------------------------- :  31
AOTVOKIR3DL2*01v1   : ---TCACTCATGGTCATCAGCATGGCATGTGTCG-------------------------------------------------------------- :  31
AOTVOKIR3DL2*02     : ---TCACTCATGGTCATCAGCATGGCATGTGTCG-------------------------------------------------------------- :  31
AOTVOKIR3DS3*01     : ---TCACTCATGGTCATCAGCATGGCATGTGTCG-------------------------------------------------------------- :  31
AOTVOKIR3DS3*02     : ---TCACTCATGGTCATCAGCATGGCATGTGTCG-------------------------------------------------------------- :  31
AOTVOKIR3DS3*03     : ----CACTCATGGTCATCAGCATGGCATGTGTCG-------------------------------------------------------------- :  30
AOTVOKIR3DS3*03v1   : ----CACTCATGGTCATCAGCATGGCATGTGTCG-------------------------------------------------------------- :  30
                         tCACTCATGGTCATCAGCATGGCATGTGTCG

                       100         *       120         *       140         *       160         *       180         *
BAC_clone_Om        : TATGAGCCTGGAGTGGAGATACGGGCCTGGAAGTGTAGACCTGGGCCTGGAGCCTGGGTCTCTGCACAGCCAAGAGCCCTGTTCCCTGCCGTCAGG : 192
AOTVOKIR3DL1*01     : ------------------------------------------------------------------------------------------------ :   -
AOTVOKIR2DL1*01v1   : ------------------------------------------------------------------------------------------------ :   -
AOTVOKIR3DS1*01v2   : ------------------------------------------------------------------------------------------------ :   -
AOTVOKIR3DS1*02     : ------------------------------------------------------------------------------------------------ :   -
AOTVOKIR3DL2*01     : ------------------------------------------------------------------------------------------------ :   -
AOTVOKIR3DL2*01v1   : ------------------------------------------------------------------------------------------------ :   -
AOTVOKIR3DL2*02     : ------------------------------------------------------------------------------------------------ :   -
AOTVOKIR3DS3*01     : ------------------------------------------------------------------------------------------------ :   -
AOTVOKIR3DS3*02     : ------------------------------------------------------------------------------------------------ :   -
AOTVOKIR3DS3*03     : ------------------------------------------------------------------------------------------------ :   -
AOTVOKIR3DS3*03v1   : ------------------------------------------------------------------------------------------------ :   -

                           200         *       220         *       240         *       260         *       280
BAC_clone_Om        : TAGGCAATGAGGCTGAGTTTACCTTTAGCCCATCAAGGGCCTGGCTGCCAATGCGCACAGCCCCAACAGGACAGCAGGGGAGGCCTGGTTTGTGTG : 288
AOTVOKIR3DL1*01     : ------------------------------------------------------------------------------------------------ :   -
AOTVOKIR2DL1*01v1   : ------------------------------------------------------------------------------------------------ :   -
AOTVOKIR3DS1*01v2   : ------------------------------------------------------------------------------------------------ :   -
AOTVOKIR3DS1*02     : ------------------------------------------------------------------------------------------------ :   -
AOTVOKIR3DL2*01     : ------------------------------------------------------------------------------------------------ :   -
AOTVOKIR3DL2*01v1   : ------------------------------------------------------------------------------------------------ :   -
AOTVOKIR3DL2*02     : ------------------------------------------------------------------------------------------------ :   -
AOTVOKIR3DS3*01     : ------------------------------------------------------------------------------------------------ :   -
AOTVOKIR3DS3*02     : ------------------------------------------------------------------------------------------------ :   -
AOTVOKIR3DS3*03     : ------------------------------------------------------------------------------------------------ :   -
AOTVOKIR3DS3*03v1   : ------------------------------------------------------------------------------------------------ :   -
```

EXON 2

```
                          *        300         *        320         *        340         *        360         *        380
BAC_clone_Om       : CAGATGAATCATCCATCATGATCTCTCTTTCCAGGGTACTTCTTGGTCCAGAGGGCCTGGACACATGAGGGTAAGTCCTTCCCTAAACTTTAGGGT :  384
AOTVOKIR3DL1*01    : ----------------------------------GGTTCTTCTTGGTCCAGAGGGCCTGGACACATGAGG-------------------------- :   67
AOTVOKIR2DL1*01v1  : ----------------------------------GGTTCTTCTTGGTCCAGAGGGCCTGGACACATGAGG-------------------------- :   67
AOTVOKIR3DS1*01v2  : ----------------------------------GGTTCTTCTTGGTCCAGAGGGCCTGGACACATGAGG-------------------------- :   67
AOTVOKIR3DS1*02    : ----------------------------------GGTTCTTCTTGGTCCAGAGGGCCTGGACACATGAGG-------------------------- :   67
AOTVOKIR3DL2*01    : ----------------------------------GGTTCTTCTTGGTCCAGAGGGCCTGGACACATGAGG-------------------------- :   67
AOTVOKIR3DL2*01v1  : ----------------------------------GGTTCTTCTTGGTCCAGAGGGCCTGGACACATGAGG-------------------------- :   67
AOTVOKIR3DL2*02    : ----------------------------------GGTTCTTCTTGGTCCAGAGGGCCTGGACACATGAGG-------------------------- :   67
AOTVOKIR3DS3*01    : ------------------------------------------------------------------------------------------------ :    -
AOTVOKIR3DS3*02    : ------------------------------------------------------------------------------------------------ :    -
AOTVOKIR3DS3*03    : ---------------------------------------------------AGGGCCTGGACACATGAGG-------------------------- :   49
AOTVOKIR3DS3*03v1  : ---------------------------------------------------AGGGCCTGGACACATGAGG-------------------------- :   49
                                                                        agggcctggacacatgagg

                              *        400         *        420         *        440         *        460         *        480
BAC_clone_Om       : GTCATCTCCCCACATAAGAGGATTTTCCTGAAACAGGAGGGAAGCCCTGTGGGGGAGTCTCTCATGAACTAGGAAGAGGGGACCCTGGGATGCTCA :  480
AOTVOKIR3DL1*01    : ------------------------------------------------------------------------------------------------ :    -
AOTVOKIR2DL1*01v1  : ------------------------------------------------------------------------------------------------ :    -
AOTVOKIR3DS1*01v2  : ------------------------------------------------------------------------------------------------ :    -
AOTVOKIR3DS1*02    : ------------------------------------------------------------------------------------------------ :    -
AOTVOKIR3DL2*01    : ------------------------------------------------------------------------------------------------ :    -
AOTVOKIR3DL2*01v1  : ------------------------------------------------------------------------------------------------ :    -
AOTVOKIR3DL2*02    : ------------------------------------------------------------------------------------------------ :    -
AOTVOKIR3DS3*01    : ------------------------------------------------------------------------------------------------ :    -
AOTVOKIR3DS3*02    : ------------------------------------------------------------------------------------------------ :    -
AOTVOKIR3DS3*03    : ------------------------------------------------------------------------------------------------ :    -
AOTVOKIR3DS3*03v1  : ------------------------------------------------------------------------------------------------ :    -

                                  *        500         *        520         *        540         *        560         *
BAC_clone_Om       : GCCCAGTTCTGACCTAGCCCTTCCCAGCCTTTCCTTCCCTTGGCTGAGTCAAGCTCTGTGGGGACCGGGGTGAGACTGGGGTGCTCCAAGCTGGGG :  576
AOTVOKIR3DL1*01    : ------------------------------------------------------------------------------------------------ :    -
AOTVOKIR2DL1*01v1  : ------------------------------------------------------------------------------------------------ :    -
AOTVOKIR3DS1*01v2  : ------------------------------------------------------------------------------------------------ :    -
AOTVOKIR3DS1*02    : ------------------------------------------------------------------------------------------------ :    -
AOTVOKIR3DL2*01    : ------------------------------------------------------------------------------------------------ :    -
AOTVOKIR3DL2*01v1  : ------------------------------------------------------------------------------------------------ :    -
AOTVOKIR3DL2*02    : ------------------------------------------------------------------------------------------------ :    -
AOTVOKIR3DS3*01    : ------------------------------------------------------------------------------------------------ :    -
AOTVOKIR3DS3*02    : ------------------------------------------------------------------------------------------------ :    -
AOTVOKIR3DS3*03    : ------------------------------------------------------------------------------------------------ :    -
AOTVOKIR3DS3*03v1  : ------------------------------------------------------------------------------------------------ :    -

                       580         *        600         *        620         *        640         *        660         *
```

```
BAC_clone_Om      : TGTGCAGGGAGGAAGTCATGTCACCGGCAGAGGAAGGGAGGGAAGCAGTGCTAGGAAAGGCCGACCCTCTGAGGACAAAGGTGTAACTCACACACT :  672
AOTVOKIR3DL1*01   : ------------------------------------------------------------------------------------------------ :    -
AOTVOKIR2DL1*01v1 : ------------------------------------------------------------------------------------------------ :    -
AOTVOKIR3DS1*01v2 : ------------------------------------------------------------------------------------------------ :    -
AOTVOKIR3DS1*02   : ------------------------------------------------------------------------------------------------ :    -
AOTVOKIR3DL2*01   : ------------------------------------------------------------------------------------------------ :    -
AOTVOKIR3DL2*01v1 : ------------------------------------------------------------------------------------------------ :    -
AOTVOKIR3DL2*02   : ------------------------------------------------------------------------------------------------ :    -
AOTVOKIR3DS3*01   : ------------------------------------------------------------------------------------------------ :    -
AOTVOKIR3DS3*02   : ------------------------------------------------------------------------------------------------ :    -
AOTVOKIR3DS3*03   : ------------------------------------------------------------------------------------------------ :    -
AOTVOKIR3DS3*03v1 : ------------------------------------------------------------------------------------------------ :    -

                           680       *       700       *       720       *       740       *       760
BAC_clone_Om      : CCAGCGTTTCCATGACGGTAGGGGCTGCTGTGTGGCTGCTGTCATTCTACCAGGAGAGGTGGGGGAACCGCAGCCGTGAGCTTCACATTCTAAATC :  768
AOTVOKIR3DL1*01   : ------------------------------------------------------------------------------------------------ :    -
AOTVOKIR2DL1*01v1 : ------------------------------------------------------------------------------------------------ :    -
AOTVOKIR3DS1*01v2 : ------------------------------------------------------------------------------------------------ :    -
AOTVOKIR3DS1*02   : ------------------------------------------------------------------------------------------------ :    -
AOTVOKIR3DL2*01   : ------------------------------------------------------------------------------------------------ :    -
AOTVOKIR3DL2*01v1 : ------------------------------------------------------------------------------------------------ :    -
AOTVOKIR3DL2*02   : ------------------------------------------------------------------------------------------------ :    -
AOTVOKIR3DS3*01   : ------------------------------------------------------------------------------------------------ :    -
AOTVOKIR3DS3*02   : ------------------------------------------------------------------------------------------------ :    -
AOTVOKIR3DS3*03   : ------------------------------------------------------------------------------------------------ :    -
AOTVOKIR3DS3*03v1 : ------------------------------------------------------------------------------------------------ :    -

                       *       780       *       800       *       820       *       840       *       860
BAC_clone_Om      : CTCTGATGGGGGCTCAGTTGTTTATTGTGGTTCACGCAGTGGCTGATACTCCATTCACAAAGGACCTGCCCCCACCCCGTGTCTACCTTGTGTTGT :  864
AOTVOKIR3DL1*01   : ------------------------------------------------------------------------------------------------ :    -
AOTVOKIR2DL1*01v1 : ------------------------------------------------------------------------------------------------ :    -
AOTVOKIR3DS1*01v2 : ------------------------------------------------------------------------------------------------ :    -
AOTVOKIR3DS1*02   : ------------------------------------------------------------------------------------------------ :    -
AOTVOKIR3DL2*01   : ------------------------------------------------------------------------------------------------ :    -
AOTVOKIR3DL2*01v1 : ------------------------------------------------------------------------------------------------ :    -
AOTVOKIR3DL2*02   : ------------------------------------------------------------------------------------------------ :    -
AOTVOKIR3DS3*01   : ------------------------------------------------------------------------------------------------ :    -
AOTVOKIR3DS3*02   : ------------------------------------------------------------------------------------------------ :    -
AOTVOKIR3DS3*03   : ------------------------------------------------------------------------------------------------ :    -
AOTVOKIR3DS3*03v1 : ------------------------------------------------------------------------------------------------ :    -

                           *       880       *       900       *       920       *       940       *       960
BAC_clone_Om      : TTTATGTAAGCAATTTTGCTGTATTCAAATCTAGTAGGAGTCCCTCATTCAGCACTTACGCAAAGTTCTCAGCTGACACGTTTGTAGTAGGAAGAC :  960
```

```
AOTVOKIR3DL1*01    : ------------------------------------------------------------------------------------------------ :    -
AOTVOKIR2DL1*01v1  : ------------------------------------------------------------------------------------------------ :    -
AOTVOKIR3DS1*01v2  : ------------------------------------------------------------------------------------------------ :    -
AOTVOKIR3DS1*02    : ------------------------------------------------------------------------------------------------ :    -
AOTVOKIR3DL2*01    : ------------------------------------------------------------------------------------------------ :    -
AOTVOKIR3DL2*01v1  : ------------------------------------------------------------------------------------------------ :    -
AOTVOKIR3DL2*02    : ------------------------------------------------------------------------------------------------ :    -
AOTVOKIR3DS3*01    : ------------------------------------------------------------------------------------------------ :    -
AOTVOKIR3DS3*02    : ------------------------------------------------------------------------------------------------ :    -
AOTVOKIR3DS3*03    : ------------------------------------------------------------------------------------------------ :    -
AOTVOKIR3DS3*03v1  : ------------------------------------------------------------------------------------------------ :    -

                                 *       980         *        1000         *        1020         *        1040         *
BAC_clone_Om       : GCCATGTCTATTCAGGATGGGTCCTTCCTGTATCTCTGGGCACCCAGGTGTCGTAGGAGCCTTAGAAATGTGGAAAAAGGGAGAATCTTCTGAGCA : 1056
AOTVOKIR3DL1*01    : ------------------------------------------------------------------------------------------------ :    -
AOTVOKIR2DL1*01v1  : ------------------------------------------------------------------------------------------------ :    -
AOTVOKIR3DS1*01v2  : ------------------------------------------------------------------------------------------------ :    -
AOTVOKIR3DS1*02    : ------------------------------------------------------------------------------------------------ :    -
AOTVOKIR3DL2*01    : ------------------------------------------------------------------------------------------------ :    -
AOTVOKIR3DL2*01v1  : ------------------------------------------------------------------------------------------------ :    -
AOTVOKIR3DL2*02    : ------------------------------------------------------------------------------------------------ :    -
AOTVOKIR3DS3*01    : ------------------------------------------------------------------------------------------------ :    -
AOTVOKIR3DS3*02    : ------------------------------------------------------------------------------------------------ :    -
AOTVOKIR3DS3*03    : ------------------------------------------------------------------------------------------------ :    -
AOTVOKIR3DS3*03v1  : ------------------------------------------------------------------------------------------------ :    -

                                                                                                   EXON 3
                     1060         *       1080         *        1100         *     1120         *       1140        *
BAC_clone_Om       : CAGGGAGAGAGGGGCCGCTCCACATCTTCCTCTCTAAGGCAATGCCTCCTGCTCCCCCAGGTGGTCGGGACAAGCCCTTCCTGTCTGCCTGGCCCA : 1152
AOTVOKIR3DL1*01    : ------------------------------------------------------------GTGGTCCGGACAAGCCCTTCCTGTCTGCCTGGCCCA :  103
AOTVOKIR2DL1*01v1  : ------------------------------------------------------------GTGGTCCGGACAAGCCCTTCCTGTCTGCCTGGCCCA :  103
AOTVOKIR3DS1*01v2  : ------------------------------------------------------------GTGGTCCGGACAAGCCCTTCCTGTCTGCCTGGCCCA :  103
AOTVOKIR3DS1*02    : ------------------------------------------------------------GTGGTCCGGACAAGCCCTTCCTGTCTGTCTGGCCCA :  103
AOTVOKIR3DL2*01    : ------------------------------------------------------------GTGGTCCGGACAAGCCCTTCCTGTCTGCCTGGCTCA :  103
AOTVOKIR3DL2*01v1  : ------------------------------------------------------------GTGGTCCGGACAAGCCCTTCCTGTCTGCCTGGCTCA :  103
AOTVOKIR3DL2*02    : ------------------------------------------------------------GTGGTCCGGACAAGCCCTTCCTGTCTGCCTGGCTCA :  103
AOTVOKIR3DS3*01    : ------------------------------------------------------------GTGGTCGGGACAAGCCCTTCCTGTCTGCCTGGCCCA :   67
AOTVOKIR3DS3*02    : ------------------------------------------------------------GTGGTCGGGACAAGCCCTTCCTGTCTGCCTGGCCCA :   67
AOTVOKIR3DS3*03    : ------------------------------------------------------------GTGGTCGGGACAAGCCCTTCCTGTCTGCCTGGCCCA :   85
AOTVOKIR3DS3*03v1  : ------------------------------------------------------------GTGGTCGGGACAAGCCCTTCCTGTCTGCCTGGCCCA :   85
                                                                                 GTGGTC GGACAAGCCCTTCCTGTCTGcCTGGC CA

                         1160         *       1180         *        1200         *       1220         *       1240
BAC_clone_Om       : GCGCTGTGGTGCTTCAAGGCAGAAATGTGACTCTTCAGTGCTACTATCGTCTTGGGTTTAACAAGTTCATGTTGTACAAGGAAGACCGAATCAACG : 1248
AOTVOKIR3DL1*01    : GCCCTGTGGTGCTTCAAGGAGGACACATGACTCTTCAGTGCTACTATCGTCTTGGGTTTAACAAGTTCATGTTGTACAAGGAAGACCGAATCAACG :  199
```

```
AOTVOKIR2DL1*01v1 : GCCCTGTGGTGCTTCAAGGAGGACACATGACTCTTCAGTGCTACTATCGTCTTGGGTTTAACAAGTTCATGTTGTACAAGGAAGACCGAATCAACG :  199
AOTVOKIR3DS1*01v2 : GCCCTGTGGTGCTTCAAGGAGGACACATGACTCTTCAGTGCTACTATCGTCTTGGGTTTAACAAGTTCATGTTGTACAAGGAAGACCGAATCAACG :  199
AOTVOKIR3DS1*02   : GCCCTGTGGTGCTTCAAGGAGGACACATGACTCTTCAGTGCTACTATCGTCTTGGGTTTAACAAGTTCATGTTGTACAAGGAAGACCGAATCAACG :  199
AOTVOKIR3DL2*01   : GCCCTGTGGTGCTTCAAGGCAGAAATGTGACTCTTCAGTGCTACTATCGTCTTGGGTTTAACAAGTTCATGTTGTACAAGGAAGACCGAATCAACG :  199
AOTVOKIR3DL2*01v1 : GCCCTGTGGTGCTTCAAGGCAGAAATGTGACTCTTCAGTGCTACTATCGTCTTGGGTTTAACAAGTTCATGTTGTACAAGGAAGACCGAATCAACG :  199
AOTVOKIR3DL2*02   : GCCCTGTGGTGCTTCAAGGCAGAAATGTGACTCTTCAGTGCTACTATCGTCTTGGGTTTAACAAGTTCATGTTGTACAAGGAAGACCGAATCAACG :  199
AOTVOKIR3DS3*01   : GCGCTGTGGTGCTTCAAGGAGGACACGTGACTCTTCAGTGTTACTATCGTCTTGGGTTTAACAAGTTCATGTTGTACAAGGAAGACCGAATCAATG :  163
AOTVOKIR3DS3*02   : GCGCTGTGGTGCTTCAAGGAGGACACGTGACTCTTCAGTGTTACTATCGTCTTGGGTTTAACAAGTTCATGTTGTACAAGGAAGACCGAATCAATG :  163
AOTVOKIR3DS3*03   : GCGCTGTGGTGCTTCAAGGAGGACACGTGACTCTTCAGTGTTACTATCGTCTTGGGTTTAACAAGTTCATGTTGTACAAGGAAGACCGAATCAATG :  181
AOTVOKIR3DS3*03v1 : GCGCTGTGGTGCTTCAAGGAGGACACGTGACTCTTCAGTGTTACTATCGTCTTGGGTTTAACAAGTTCATGTTGTACAAGGAAGACCGAATCAATG :  181
                    GC CTGTGGTGCTTCAAGG  GA A  TGACTCTTCAGTG TACTATCGTCTTGGGTTTAACAAGTTCATGTTGTACAAGGAAGACCGAATCAA G

                     *      1260       *      1280       *      1300       *      1320       *      1340
BAC_clone_Om      : TTCCCATCCTCCAGGGCAGAATATTCCAGGAGAGCTTTCTCATGGGCCCTGTGACCCCAGCACACGCAGGGACCTACAGATGCCGGGCTTCTCATC : 1344
AOTVOKIR3DL1*01   : TTCCCAGCCTCCAGGGCAGAATATTCCAGGAGAGCTTTCTCATGGGCCCTGTGACCCCAGCACACGCAGGGACCTACAGATGCCGGGCTTCTCACC :  295
AOTVOKIR2DL1*01v1 : TTCCCAGCCTCCAGGGCAGAATATTCCAGGAGAGCTTTCTCATGGGCCCTGTGACCCCAGCACACGCAGGGACCTACAGATGCCGGGCTTCTCACC :  295
AOTVOKIR3DS1*01v2 : TTCCCAGCCTCCAGGGCAGAATATTCCAGGAGAGCTTTCTCATGGGCCCTGTGACCCCAGCACACGCAGGGACCTACAGATGCCGGGCTTCTCACC :  295
AOTVOKIR3DS1*02   : TTCCCAGCCTCCAGGGCAGAATATTCCAGGAGAGCTTTCTCATGGGCCCTGTGACCCCAGCACACGCAGGGACCTACAGATGCCGGGCTTCTCACC :  295
AOTVOKIR3DL2*01   : TTCCCATCCTCCAGGGCAGAATACTCCAGGAGAGCTTTCTCATGGGCCCTGTGACCCCAGCACACGCAGGGACCTACAGATGCCGGGCTTCTCACC :  295
AOTVOKIR3DL2*01v1 : TTCCCATCCTCCAGGGCAGAATACTCCAGGAGAGCTTTCTCATGGGCCCTGTGACCCCAGCACACGCAGGGACCTACAGATGCCGGGCTTCTCACC :  295
AOTVOKIR3DL2*02   : TTCCCATCCTCCAGGGCAGAATACTCCAGGAGAGCTTTCTCATGGGCCCTGTGACCCCAGCACACGCAGGGACCTACAGATGCCGGGCTTCTCACC :  295
AOTVOKIR3DS3*01   : TTCCCATCCTCCAGGGCAGAATATTCCAGGAGCGCTTTCTCATGGGCCCTGTGACCCCAGCACACGCAGGGACCTACGGATGCCGGGCTTCTCACC :  259
AOTVOKIR3DS3*02   : TTCCCATCCTCCAGGGCAGAATATTCCAGGAGCGCTTTCTCATGGGCCCTGTGACCCCAGCACACGCAGGGACCTACAGATGCCGGGCTTCTCACC :  259
AOTVOKIR3DS3*03   : TTCCCATCCTCCAGGGCAGAATATTCCAGGAGCGCTTTCTCATGGGCCCTGTGACCCCAGCACACGCAGGGACCTACAGATGCCGGGCTTCTCACC :  277
AOTVOKIR3DS3*03v1 : TTCCCATCCTCCAGGGCAGAATATTCCAGGAGCGCTTTCTCATGGGCCCTGTGACCCCAGCACACGCAGGGACCTACAGATGCCGGGCTTCTCACC :  277
                    TTCCCA CCTCCAGGGCAGAATA TCCAGGAG GCTTTCTCATGGGCCCTGTGACCCCAGCACACGCAGGGACCTACaGATGCCGGGCTTCTCAcC

                         *      1360       *      1380       *      1400       *      1420       *      1440
BAC_clone_Om      : CGCACTCACCTACTCCATGGTCGGCACTCAGTAACCCCCTGGTGATCAGGGTCACAGGTCAGAGGGCTCCTGCCTGGGCTTCTCACTGTCCCACCT : 1440
AOTVOKIR3DL1*01   : CCCACTCGCCTACTCCGTGGTCGGCACTCAGTAACCCCCTGGTGATCAGGGTCACAG--------------------------------------- :  352
AOTVOKIR2DL1*01v1 : CCCACTCGCCTACTCCGTGGTCGGCACTCAGTAACCCCCTGGTGATCAGGGTCACAG--------------------------------------- :  352
AOTVOKIR3DS1*01v2 : CCCACTCGCCTACTCCGTGGTCGGCACTCAGTAACCCCCTGGTGATCAGGGTCACAG--------------------------------------- :  352
AOTVOKIR3DS1*02   : CCCACTCGCCTACTCCGTGGTCGGCACTCAGTAACCCCCTGGTGATCAGGGTCACAG--------------------------------------- :  352
AOTVOKIR3DL2*01   : CCCACTCGCCTACTCCGTGGTCGGCACTCAGTAACCCCCTGGTGATCAGGGTCACAG--------------------------------------- :  352
AOTVOKIR3DL2*01v1 : CCCACTCGCCTACTCCGTGGTCGGCACTCAGTAACCCCCTGGTGATCAGGGTCACAG--------------------------------------- :  352
AOTVOKIR3DL2*02   : CCCACTCGCCTACTCCGTGGTCGGCACTCAGTAACCCCCTGGTGATCAGGGTCACAG--------------------------------------- :  352
AOTVOKIR3DS3*01   : CCCACTCGCCTACTCCGTGGTCGGCACTCAGTAACCCCCTGGTGATCAGGGTCACAG--------------------------------------- :  316
AOTVOKIR3DS3*02   : CCCACTCGCCTACTCCGTGGTCGGCACTCAGTAACCCCCTGGTGATCAGGGTCACAG--------------------------------------- :  316
AOTVOKIR3DS3*03   : CCCACTCGCCTACTCCGTGGTCGGCACTCAGTAACCCCCTGGTGATCAGGGTCACAG--------------------------------------- :  334
AOTVOKIR3DS3*03v1 : CCCACTCGCCTACTCCGTGGTCGGCACTCAGTAACCCCCTGGTGATCAGGGTCACAG--------------------------------------- :  334
                    CcCACTCgCCTACTCCgTGGTCGGCACTCAGTAACCCCCTGGTGATCAGGGTCACAG

                             *      1460       *      1480       *      1500       *      1520       *
BAC_clone_Om      : CCTGAATCACAGAGCTTCCA-GGCCCTGACTGTATTTGAGGTTAAGGGGATTGAATACAGGGAAGTGGGTGCTGTGGTGGGAAGAATAACTCTCCC : 1535
AOTVOKIR3DL1*01   : ------------------------------------------------------------------------------------------------ :    -
AOTVOKIR2DL1*01v1 : ------------------------------------------------------------------------------------------------ :    -
```

```
AOTVOKIR3DS1*01v2 : ------------------------------------------------------------------------------------------------ :     -
AOTVOKIR3DS1*02   : ------------------------------------------------------------------------------------------------ :     -
AOTVOKIR3DL2*01   : ------------------------------------------------------------------------------------------------ :     -
AOTVOKIR3DL2*01v1 : ------------------------------------------------------------------------------------------------ :     -
AOTVOKIR3DL2*02   : ------------------------------------------------------------------------------------------------ :     -
AOTVOKIR3DS3*01   : ------------------------------------------------------------------------------------------------ :     -
AOTVOKIR3DS3*02   : ------------------------------------------------------------------------------------------------ :     -
AOTVOKIR3DS3*03   : ------------------------------------------------------------------------------------------------ :     -
AOTVOKIR3DS3*03v1 : ------------------------------------------------------------------------------------------------ :     -

                    1540         *       1560         *       1580         *       1600         *       1620         *
BAC_clone_Om      : CAATGATGGCCACATTCTCATCCCTGGAGCCTGTTACT--ATTTATGTTACAGGGCAGGGCACTGAAGGGGGAAGATGGAGCTCAGGTTGTTGATG :  1629
AOTVOKIR3DL1*01   : ------------------------------------------------------------------------------------------------ :     -
AOTVOKIR2DL1*01v1 : ------------------------------------------------------------------------------------------------ :     -
AOTVOKIR3DS1*01v2 : ------------------------------------------------------------------------------------------------ :     -
AOTVOKIR3DS1*02   : ------------------------------------------------------------------------------------------------ :     -
AOTVOKIR3DL2*01   : ------------------------------------------------------------------------------------------------ :     -
AOTVOKIR3DL2*01v1 : ------------------------------------------------------------------------------------------------ :     -
AOTVOKIR3DL2*02   : ------------------------------------------------------------------------------------------------ :     -
AOTVOKIR3DS3*01   : ------------------------------------------------------------------------------------------------ :     -
AOTVOKIR3DS3*02   : ------------------------------------------------------------------------------------------------ :     -
AOTVOKIR3DS3*03   : ------------------------------------------------------------------------------------------------ :     -
AOTVOKIR3DS3*03v1 : ------------------------------------------------------------------------------------------------ :     -

                        1640         *       1660         *       1680         *       1700         *       1720
BAC_clone_Om      : AGTTGACCTTGAGATGGGGAGGCCACCTGGACTGTCCAGCTGGGCTCAGTGTCATCACAAGTGCCCACATGAGAGGAGGAGGAAGAGGGGAATGGG :  1725
AOTVOKIR3DL1*01   : ------------------------------------------------------------------------------------------------ :     -
AOTVOKIR2DL1*01v1 : ------------------------------------------------------------------------------------------------ :     -
AOTVOKIR3DS1*01v2 : ------------------------------------------------------------------------------------------------ :     -
AOTVOKIR3DS1*02   : ------------------------------------------------------------------------------------------------ :     -
AOTVOKIR3DL2*01   : ------------------------------------------------------------------------------------------------ :     -
AOTVOKIR3DL2*01v1 : ------------------------------------------------------------------------------------------------ :     -
AOTVOKIR3DL2*02   : ------------------------------------------------------------------------------------------------ :     -
AOTVOKIR3DS3*01   : ------------------------------------------------------------------------------------------------ :     -
AOTVOKIR3DS3*02   : ------------------------------------------------------------------------------------------------ :     -
AOTVOKIR3DS3*03   : ------------------------------------------------------------------------------------------------ :     -
AOTVOKIR3DS3*03v1 : ------------------------------------------------------------------------------------------------ :     -

                     *       1740         *       1760         *       1780         *       1800         *       1820
BAC_clone_Om      : GATTAGAGCAGCGTAGTGGGAGGGAGACTCCACCAGCCACTGCGGCCTCTG--CAGGTGGAGGACAGCCAGGAGCCATGAATGCAGGTGGCCTCTA :  1819
AOTVOKIR3DL1*01   : ------------------------------------------------------------------------------------------------ :     -
AOTVOKIR2DL1*01v1 : ------------------------------------------------------------------------------------------------ :     -
AOTVOKIR3DS1*01v2 : ------------------------------------------------------------------------------------------------ :     -
```

```
AOTVOKIR3DS1*02    : ------------------------------------------------------------------------------------------------ :     -
AOTVOKIR3DL2*01    : ------------------------------------------------------------------------------------------------ :     -
AOTVOKIR3DL2*01v1  : ------------------------------------------------------------------------------------------------ :     -
AOTVOKIR3DL2*02    : ------------------------------------------------------------------------------------------------ :     -
AOTVOKIR3DS3*01    : ------------------------------------------------------------------------------------------------ :     -
AOTVOKIR3DS3*02    : ------------------------------------------------------------------------------------------------ :     -
AOTVOKIR3DS3*03    : ------------------------------------------------------------------------------------------------ :     -
AOTVOKIR3DS3*03v1  : ------------------------------------------------------------------------------------------------ :     -

                          *      1840         *      1860         *      1880         *      1900         *      1920
BAC_clone_Om       : AGGGCTGGAGAAGTCAAGGGAACTGATTCTCCCCTGAGTCTCCAGACGGAATTCAGCCCTACAGATGCCCTGATTTTAGCCCAGGGAGAACTGGGC :  1915
AOTVOKIR3DL1*01    : ------------------------------------------------------------------------------------------------ :     -
AOTVOKIR2DL1*01v1  : ------------------------------------------------------------------------------------------------ :     -
AOTVOKIR3DS1*01v2  : ------------------------------------------------------------------------------------------------ :     -
AOTVOKIR3DS1*02    : ------------------------------------------------------------------------------------------------ :     -
AOTVOKIR3DL2*01    : ------------------------------------------------------------------------------------------------ :     -
AOTVOKIR3DL2*01v1  : ------------------------------------------------------------------------------------------------ :     -
AOTVOKIR3DL2*02    : ------------------------------------------------------------------------------------------------ :     -
AOTVOKIR3DS3*01    : ------------------------------------------------------------------------------------------------ :     -
AOTVOKIR3DS3*02    : ------------------------------------------------------------------------------------------------ :     -
AOTVOKIR3DS3*03    : ------------------------------------------------------------------------------------------------ :     -
AOTVOKIR3DS3*03v1  : ------------------------------------------------------------------------------------------------ :     -

                              *      1940         *      1960         *      1980         *      2000         *
BAC_clone_Om       : CCGATTTCTGTCTCCAGAAGTGGAAGGGCTCAGTGTGTTCTCTCCTGCCGTCATGTTTTGACAATTTTCTACAGCAGCAACAGCAAACAACACAGG :  2011
AOTVOKIR3DL1*01    : ------------------------------------------------------------------------------------------------ :     -
AOTVOKIR2DL1*01v1  : ------------------------------------------------------------------------------------------------ :     -
AOTVOKIR3DS1*01v2  : ------------------------------------------------------------------------------------------------ :     -
AOTVOKIR3DS1*02    : ------------------------------------------------------------------------------------------------ :     -
AOTVOKIR3DL2*01    : ------------------------------------------------------------------------------------------------ :     -
AOTVOKIR3DL2*01v1  : ------------------------------------------------------------------------------------------------ :     -
AOTVOKIR3DL2*02    : ------------------------------------------------------------------------------------------------ :     -
AOTVOKIR3DS3*01    : ------------------------------------------------------------------------------------------------ :     -
AOTVOKIR3DS3*02    : ------------------------------------------------------------------------------------------------ :     -
AOTVOKIR3DS3*03    : ------------------------------------------------------------------------------------------------ :     -
AOTVOKIR3DS3*03v1  : ------------------------------------------------------------------------------------------------ :     -

                     2020         *      2040         *      2060         *      2080         *      2100         *
BAC_clone_Om       : AACCCAGGGCAAGGACAAGTCAAGAAACCACACCAGGAGAAGGGTGGCCACCCCGAGATCAGCAAGAGAGGGATGCTGAGGCCACCACCAGGCTGG :  2107
AOTVOKIR3DL1*01    : ------------------------------------------------------------------------------------------------ :     -
AOTVOKIR2DL1*01v1  : ------------------------------------------------------------------------------------------------ :     -
AOTVOKIR3DS1*01v2  : ------------------------------------------------------------------------------------------------ :     -
AOTVOKIR3DS1*02    : ------------------------------------------------------------------------------------------------ :     -
```

```
AOTVOKIR3DL2*01    : ------------------------------------------------------------------------------------------------ :     -
AOTVOKIR3DL2*01v1  : ------------------------------------------------------------------------------------------------ :     -
AOTVOKIR3DL2*02    : ------------------------------------------------------------------------------------------------ :     -
AOTVOKIR3DS3*01    : ------------------------------------------------------------------------------------------------ :     -
AOTVOKIR3DS3*02    : ------------------------------------------------------------------------------------------------ :     -
AOTVOKIR3DS3*03    : ------------------------------------------------------------------------------------------------ :     -
AOTVOKIR3DS3*03v1  : ------------------------------------------------------------------------------------------------ :     -


                         2120         *       2140         *       2160         *       2180         *       2200
BAC_clone_Om       : AGCCACATAGGGAGGGGTCGATGCTCCTGAAACCAGCACCAGGGACCGCCCTATGGAAGCTGGGAC-CATGGAGAAGCACAGACATGGCAGGAGAG :  2202
AOTVOKIR3DL1*01    : ------------------------------------------------------------------------------------------------ :     -
AOTVOKIR2DL1*01v1  : ------------------------------------------------------------------------------------------------ :     -
AOTVOKIR3DS1*01v2  : ------------------------------------------------------------------------------------------------ :     -
AOTVOKIR3DS1*02    : ------------------------------------------------------------------------------------------------ :     -
AOTVOKIR3DL2*01    : ------------------------------------------------------------------------------------------------ :     -
AOTVOKIR3DL2*01v1  : ------------------------------------------------------------------------------------------------ :     -
AOTVOKIR3DL2*02    : ------------------------------------------------------------------------------------------------ :     -
AOTVOKIR3DS3*01    : ------------------------------------------------------------------------------------------------ :     -
AOTVOKIR3DS3*02    : ------------------------------------------------------------------------------------------------ :     -
AOTVOKIR3DS3*03    : ------------------------------------------------------------------------------------------------ :     -
AOTVOKIR3DS3*03v1  : ------------------------------------------------------------------------------------------------ :     -


                      *       2220         *       2240         *       2260         *       2280         *       2300
BAC_clone_Om       : GTTCCCAGTCCCCACCAGGAACAGGGTGTGTGGACACTGCTGCCCGCCTTACTCATCAGTTCATACCTCCTGCCAGGGATTCCAGTTTGTCCTAAA :  2298
AOTVOKIR3DL1*01    : ------------------------------------------------------------------------------------------------ :     -
AOTVOKIR2DL1*01v1  : ------------------------------------------------------------------------------------------------ :     -
AOTVOKIR3DS1*01v2  : ------------------------------------------------------------------------------------------------ :     -
AOTVOKIR3DS1*02    : ------------------------------------------------------------------------------------------------ :     -
AOTVOKIR3DL2*01    : ------------------------------------------------------------------------------------------------ :     -
AOTVOKIR3DL2*01v1  : ------------------------------------------------------------------------------------------------ :     -
AOTVOKIR3DL2*02    : ------------------------------------------------------------------------------------------------ :     -
AOTVOKIR3DS3*01    : ------------------------------------------------------------------------------------------------ :     -
AOTVOKIR3DS3*02    : ------------------------------------------------------------------------------------------------ :     -
AOTVOKIR3DS3*03    : ------------------------------------------------------------------------------------------------ :     -
AOTVOKIR3DS3*03v1  : ------------------------------------------------------------------------------------------------ :     -


                          *       2320         *       2340         *       2360         *       2380         *       2400
BAC_clone_Om       : CACATTGAACCAGGCTGTTCAGATCCTGGACGTGCAGCCTGTCGTGGCTCCTCTTCCACCCTCACATGGACAGGAAGAAACAGATTAGTGGGAAAC :  2394
AOTVOKIR3DL1*01    : ------------------------------------------------------------------------------------------------ :     -
AOTVOKIR2DL1*01v1  : ------------------------------------------------------------------------------------------------ :     -
AOTVOKIR3DS1*01v2  : ------------------------------------------------------------------------------------------------ :     -
AOTVOKIR3DS1*02    : ------------------------------------------------------------------------------------------------ :     -
AOTVOKIR3DL2*01    : ------------------------------------------------------------------------------------------------ :     -
```

```
AOTVOKIR3DL2*01v1  : ------------------------------------------------------------------------------------------------ :      -
AOTVOKIR3DL2*02    : ------------------------------------------------------------------------------------------------ :      -
AOTVOKIR3DS3*01    : ------------------------------------------------------------------------------------------------ :      -
AOTVOKIR3DS3*02    : ------------------------------------------------------------------------------------------------ :      -
AOTVOKIR3DS3*03    : ------------------------------------------------------------------------------------------------ :      -
AOTVOKIR3DS3*03v1  : ------------------------------------------------------------------------------------------------ :      -

                              *      2420         *      2440         *      2460         *      2480         *
BAC_clone_Om       : AGAAACAGCTCAAGGGATGAGGCTGAGCCCAGTGGGAAGGGAGTCAGGGGCTGCTAGAGATGGAGACAGAGAAGAGGGAGGGGGACAGATGGAGGG :   2490
AOTVOKIR3DL1*01    : ------------------------------------------------------------------------------------------------ :      -
AOTVOKIR2DL1*01v1  : ------------------------------------------------------------------------------------------------ :      -
AOTVOKIR3DS1*01v2  : ------------------------------------------------------------------------------------------------ :      -
AOTVOKIR3DS1*02    : ------------------------------------------------------------------------------------------------ :      -
AOTVOKIR3DL2*01    : ------------------------------------------------------------------------------------------------ :      -
AOTVOKIR3DL2*01v1  : ------------------------------------------------------------------------------------------------ :      -
AOTVOKIR3DL2*02    : ------------------------------------------------------------------------------------------------ :      -
AOTVOKIR3DS3*01    : ------------------------------------------------------------------------------------------------ :      -
AOTVOKIR3DS3*02    : ------------------------------------------------------------------------------------------------ :      -
AOTVOKIR3DS3*03    : ------------------------------------------------------------------------------------------------ :      -
AOTVOKIR3DS3*03v1  : ------------------------------------------------------------------------------------------------ :      -

                     2500         *      2520         *      2540         *      2560         *      2580         *
BAC_clone_Om       : ACCTGCACCAGGGGTGACGGGCACAGAAAGGGGCACGGAGACACAGAGAGGGAGGAGAGAGTCAGACACCGGGGAGGGGAAGCCTCACTCATTCCA :   2586
AOTVOKIR3DL1*01    : ------------------------------------------------------------------------------------------------ :      -
AOTVOKIR2DL1*01v1  : ------------------------------------------------------------------------------------------------ :      -
AOTVOKIR3DS1*01v2  : ------------------------------------------------------------------------------------------------ :      -
AOTVOKIR3DS1*02    : ------------------------------------------------------------------------------------------------ :      -
AOTVOKIR3DL2*01    : ------------------------------------------------------------------------------------------------ :      -
AOTVOKIR3DL2*01v1  : ------------------------------------------------------------------------------------------------ :      -
AOTVOKIR3DL2*02    : ------------------------------------------------------------------------------------------------ :      -
AOTVOKIR3DS3*01    : ------------------------------------------------------------------------------------------------ :      -
AOTVOKIR3DS3*02    : ------------------------------------------------------------------------------------------------ :      -
AOTVOKIR3DS3*03    : ------------------------------------------------------------------------------------------------ :      -
AOTVOKIR3DS3*03v1  : ------------------------------------------------------------------------------------------------ :      -

                                                                                            EXON 4
                         2600         *      2620         *      2640         *      2660         *      2680
BAC_clone_Om       : GGTCCCATGGATGGGATGAGAAAGGGAGATGCCTTCTGAACTCACAATCTCGTTTCTTAGGACTCCACAGAAAACCTTCCCTTCTGGCCCTCCCAG :   2682
AOTVOKIR3DL1*01    : ------------------------------------------------------------GAGTGCACAGAAAACCTTCCCTCCTGGCCCTCCCAG :    388
AOTVOKIR2DL1*01v1  : ------------------------------------------------------------GAGTGCACAGAAAACCTTCCCTCCTGGCCCTCCCAG :    388
AOTVOKIR3DS1*01v2  : ------------------------------------------------------------GAGTGCACAGAAAACCTTCCCTCCTGGCCCTCCCAG :    388
AOTVOKIR3DS1*02    : ------------------------------------------------------------GAGTGCACAGAAAACCTTCCCTCCTGGCCCTCCCAG :    388
AOTVOKIR3DL2*01    : ------------------------------------------------------------GAGTGCACAGAAAACCTTCCCTTCTGGTCCTCCCAG :    388
AOTVOKIR3DL2*01v1  : ------------------------------------------------------------------------------------------------ :      -
```

EXON 4

```
AOTVOKIR3DL2*02   : ------------------------------------------------------------GAGTGCACAGAAAACCTTCCCTTCTGGTCCTCCCAG : 388
AOTVOKIR3DS3*01   : ------------------------------------------------------------GAGTGCACAGAAAACCTTCCCTCCTGGCCCTCCCAA : 352
AOTVOKIR3DS3*02   : ------------------------------------------------------------GAGTGCACAGAAAACCTTCCCTCCTGGCCCTCCCAA : 352
AOTVOKIR3DS3*03   : ------------------------------------------------------------GAGTGCACAGAAAACCTTCCCTCCTGGCCCTCCCAA : 370
AOTVOKIR3DS3*03v1 : ------------------------------------------------------------GAGTGCACAGAAAACCTTCCCTCCTGGCCCTCCCAA : 370
                                                                                  gagtgcacagaaaaccttccct ctgg cctccca

                       *        2700         *        2720         *        2740         *        2760         *        2780
BAC_clone_Om      : CTCCCCTGGTGACATCAGGAAAGACGGTCATCCTGCAATGTTGGTCAGATGTCATGTGTGAGCACTTCCTTCTGCACAGAGAGGGGATCGCTGAGG : 2778
AOTVOKIR3DL1*01   : GTCCCCTGGTGAAATCAGGAGAGATGGTCATCCTGCAATGCTGGTCAGATGTCATGTTTGAGCACTTCCTTCTGCACAGAGAGGGGATCACTGAGG : 484
AOTVOKIR2DL1*01v1 : GTCCCCTGGTGAAATCAGGAGAGATGGTCATCCTGCAATGCTGGTCAGATGTCATGTTTGAGCACTTCCTTCTGCACAGAGAGGGGATCACTGAGG : 484
AOTVOKIR3DS1*01v2 : GTCCCCTGGTGAAATCAGGAGAGATGGTCATCCTGCAATGCTGGTCAGATGTCATGTTTGAGCACTTCCTTCTGCACAGAGAGGGGATCACTGAGG : 484
AOTVOKIR3DS1*02   : GTCCCCTGGTGAAATCAGGAGAGATGGTCATCCTGCAATGCTGGTCAGATGTCATGTTTGAGCACTTCCTTCTGCACAGAGAGGGGATCACTGAGG : 484
AOTVOKIR3DL2*01   : GTCCCCTGGTGAAATCAGGAGAGATGGTCATCCTGCAATGCTGGTCAGATGTCATGTGTGAGCACTTCCTTCTGCACAGAGAGGGGATCACTGAGG : 484
AOTVOKIR3DL2*01v1 : GTCCCCTGGTGAAATCAGGAGAGATGGTCATCCTGCAATGCTGGTCAGATGTCATGTGTGAGCACTTCCTTCTGCACAGAGAGGGGATCACTGAGG : 448
AOTVOKIR3DL2*02   : GTCCCCTGGTGAAATCAGGAGAGATGGTCATCCTGCAATGCTGGTCAGATGTCATGTGTGAGCACTTCCTTCTGCACAGAGAGGGGATCACTGAGG : 484
AOTVOKIR3DS3*01   : GTCCCCTGGTGAAATTGGAAGAGATGGTCATCCTGCAATGCTGGTCAGATGTCATGTGTGAGCACTTCCTTCTGCACAGAGAGGGGATGGCTGAGG : 448
AOTVOKIR3DS3*02   : GTCCCCTGGTGAAATTGGAAGAGATGGTCATCCTGCAATGCTGGTCAGATGTCATGTGTGAGCACTTCCTTCTGCACAGAGAGGGGATGGCTGAGG : 448
AOTVOKIR3DS3*03   : GTCCCCTGGTGAAATTGGAAGAGATGGTCATCCTGCAATGCTGGTCAGATGTCATGTGTGAGCACTTCCTTCTGCACAGAGAGGGGATGGCTGAGG : 466
AOTVOKIR3DS3*03v1 : GTCCCCTGGTGAAATTGGAAGAGATGGTCATCCTGCAATGCTGGTCAGATGTCATGTGTGAGCACTTCCTTCTGCACAGAGAGGGGATGGCTGAGG : 466
                    gTCCCCTGGTGAaAT  G AgAGAtGGTCATCCTGCAATGcTGGTCAGATGTCATGT TGAGCACTTCCTTCTGCACAGAGAGGGGAT  CTGAGG

                        *        2800         *        2820         *        2840         *        2860         *        2880
BAC_clone_Om      : CCTCCTGGAGCCTCGTTGGAGAGCCCCATGGTGGCGGCTCCCAGGCCAACTTCTCCATAGGTCCCATGACAACTCACCTTGCAGGGACCTACAGAT : 2874
AOTVOKIR3DL1*01   : CCTCCTGGAGCCTCGTTGGAGAGCCCCGTGATGGTGGCTCCCAGGCCAATTTCTCCATGGAACCCATGACGCCTGCCCTTGCAGGGACCTACAGAT : 580
AOTVOKIR2DL1*01v1 : CCTCCTGGAGCCTCGTTGGAGAGCCCCGTGATGGTGGCTCCCAGGCCAATTTCTCCATGGAACCCATGACGCCTGCCCTTGCAGGGACCTACAGAT : 580
AOTVOKIR3DS1*01v2 : CCTCCTGGAGCCTCGTTGGAGAGCCCCGTGATGGTGGCTCCCAGGCCAATTTCTCCATGGAACCCATGACGCCTGCCCTTGCAGGGACCTACAGAT : 580
AOTVOKIR3DS1*02   : CCTCCTGGAGCCTCGTTGGAGAGCCCCGTGATGGTGGCTCCCAGGCCAATTTCTCCATGGAACCCATGACGCCTGCCCTTGCAGGGACCTACAGAT : 580
AOTVOKIR3DL2*01   : CCTCCTGGAGCCTCGTTGGAGAGCCCCGTGATGGTGGCTCCCAGGTCAATTTCTCCATGGAACCCATGACACCTGCCCTTGCAGGGACCTACAGAT : 580
AOTVOKIR3DL2*01v1 : CCTCCTGGAGCCTCGTTGGAGAGCCCCGTGATGGTGGCTCCCAGGTCAATTTCTCCATGGAACCCATGACACCTGCCCTTGCAGGGACCTACAGAT : 544
AOTVOKIR3DL2*02   : CCTCCTGGAGCCTCGTTGGAGAGCCCCGTGATGGTGGCTCCCAGGTCAATTTCTCCATGGAACCCATGACACCTGCCCTTGCAGGGACCTACAGAT : 580
AOTVOKIR3DS3*01   : CCTCCTGGAGCCTCGTTGGAGAGCCCCGTGGTGGCGGCTCCCAGGCCAACTTCTCCATAGGTCCCATGGTGCCTGCCCTTGCAGGGACCTACAGAT : 544
AOTVOKIR3DS3*02   : CCTCCTGGAGCCTCGTTGGAGAGCCCCGTGGTGGCGGCTCCCAGGCCAACTTCTCCATAGGTCCCATGGTGCCTGCCCTTGCAGGGACCTACAGAT : 544
AOTVOKIR3DS3*03   : CCTCCTGGAGCCTCGTTGGAGAGCCCCGTGGTGGCGGCTCCCAGGCCAACTTCTCCATAGGTCCCATGGTGCCTGCCCTTGCAGGGACCTACAGAT : 562
AOTVOKIR3DS3*03v1 : CCTCCTGGAGCCTCGTTGGAGAGCCCCGTGGTGGCGGCTCCCAGGCCAACTTCTCCATAGGTCCCATGGTGCCTGCCCTTGCAGGGACCTACAGAT : 562
                    CCTCCTGGAGCCTCGTTGGAGAGCCCCgTG TGG GGCTCCCAGG CAA TTCTCCAT G  CCCATG   cCTgcCCTTGCAGGGACCTACAGAT

                            *        2900         *        2920         *        2940         *        2960         *
BAC_clone_Om      : GCTACGGTTCTGTTACTGACTCTCTCTATGAGTGGTCGGCTCCCAGTGACCCCCTGGACATCGTGATCACAGGTGACAGTATCCAGATATTCTTCT : 2970
AOTVOKIR3DL1*01   : GCTACGGTTCTGTTACTGACTCTCTCTATGAGTGGTCGGCTCCCAGTGACCCCCTGGACATCGTGATCACAG------------------------ : 652
AOTVOKIR2DL1*01v1 : GCTACGGTTCTGTTACTGACTCTCTCTATGAGTGGTCGGCTCCCAGTGACCCCCTGGACATCGTGATCACAG------------------------ : 652
AOTVOKIR3DS1*01v2 : GCTACGGTTCTGTTACTGACTCTCTCTATGAGTGGTCGGCTCCCAGTGACCCCCTGGACATCGTGATCACAG------------------------ : 652
AOTVOKIR3DS1*02   : GCTACGGTTCTGTTACTGACTCTCTCTATGAGTGGTCGGCTCCCAGTGACCCCCTGGACATCGTGATCACAG------------------------ : 652
AOTVOKIR3DL2*01   : GCTACGGTTCTGTTACTGACTCTCTCTATGAGTGGTCGGCTCCCAGTGACCCCCTGGACATCGTGATCACAG------------------------ : 652
AOTVOKIR3DL2*01v1 : GCTACGGTTCTGTTACTGACTCTCTCTATGAGTGGTCGGCTCCCAGTGACCCCCTGGACATCGTGATCACAG------------------------ : 616
AOTVOKIR3DL2*02   : GCTACGGTTCTGTTACTGACTCTCTCTATGAGTGGTCGGCTCCCAGTGACCCCCTGGACATCGTGATCACAG------------------------ : 652
```

```
AOTVOKIR3DS3*01   : GCTACGGTTCTGTTACTGACTCTCTCTATGAGTGGTCGGCTCCCAGTGACCCCCTGGACATCGTGATCACAG------------------------ :  616
AOTVOKIR3DS3*02   : GCTACGGTTCTGTTACTGACTCTCTCTATGAGTGGTCGGCTCCCAGTGACCCCCTGGACATCGTGATCACAG------------------------ :  616
AOTVOKIR3DS3*03   : GCTACGGTTCTGTTACTGACTCTCTCTATGAGTGGTCGGCTCCCAGTGACCCCCTGGACATCGTGATCACAG------------------------ :  634
AOTVOKIR3DS3*03v1 : GCTACGGTTCTGTTACTGACTCTCTCTATGAGTGGTCGGCTCCCAGTGACCCCCTGGACATCGTGATCACAG------------------------ :  634
                    GCTACGGTTCTGTTACTGACTCTCTCTATGAGTGGTCGGCTCCCAGTGACCCCCTGGACATCGTGATCACAG

                    2980         *        3000         *        3020         *        3040         *        3060         *
BAC_clone_Om      : CATTGTCAATAGGACACAGAGTGAATGATCCAGGACTTGGAAGCCCCAGGTGCTCATGAGGAAGATGAGCGTGGGGTTCTTATGGAGAGAGACTGA : 3066
AOTVOKIR3DL1*01   : ------------------------------------------------------------------------------------------------ :    -
AOTVOKIR2DL1*01v1 : ------------------------------------------------------------------------------------------------ :    -
AOTVOKIR3DS1*01v2 : ------------------------------------------------------------------------------------------------ :    -
AOTVOKIR3DS1*02   : ------------------------------------------------------------------------------------------------ :    -
AOTVOKIR3DL2*01   : ------------------------------------------------------------------------------------------------ :    -
AOTVOKIR3DL2*01v1 : ------------------------------------------------------------------------------------------------ :    -
AOTVOKIR3DL2*02   : ------------------------------------------------------------------------------------------------ :    -
AOTVOKIR3DS3*01   : ------------------------------------------------------------------------------------------------ :    -
AOTVOKIR3DS3*02   : ------------------------------------------------------------------------------------------------ :    -
AOTVOKIR3DS3*03   : ------------------------------------------------------------------------------------------------ :    -
AOTVOKIR3DS3*03v1 : ------------------------------------------------------------------------------------------------ :    -

                        3080         *        3100         *        3120         *        3140         *        3160
BAC_clone_Om      : CTTGGTGAGGTCTGTACCCGCAGACACAGAGAAACAGGAGACACAAGTCCAGACCACACGTCATAACAGAGGACAGACACAGGGGCCATACAGGGA : 3162
AOTVOKIR3DL1*01   : ------------------------------------------------------------------------------------------------ :    -
AOTVOKIR2DL1*01v1 : ------------------------------------------------------------------------------------------------ :    -
AOTVOKIR3DS1*01v2 : ------------------------------------------------------------------------------------------------ :    -
AOTVOKIR3DS1*02   : ------------------------------------------------------------------------------------------------ :    -
AOTVOKIR3DL2*01   : ------------------------------------------------------------------------------------------------ :    -
AOTVOKIR3DL2*01v1 : ------------------------------------------------------------------------------------------------ :    -
AOTVOKIR3DL2*02   : ------------------------------------------------------------------------------------------------ :    -
AOTVOKIR3DS3*01   : ------------------------------------------------------------------------------------------------ :    -
AOTVOKIR3DS3*02   : ------------------------------------------------------------------------------------------------ :    -
AOTVOKIR3DS3*03   : ------------------------------------------------------------------------------------------------ :    -
AOTVOKIR3DS3*03v1 : ------------------------------------------------------------------------------------------------ :    -

                     *        3180         *        3200         *        3220         *        3240         *        3260
BAC_clone_Om      : GTGAGAAAAGAGGGAAGGAGGTAGAGGGGACACACAGACAGACAGACATGTCCCAGAGAGAGGTGTCCTTCCATGCTGGCTTTGTTCAGACACCTG : 3258
AOTVOKIR3DL1*01   : ------------------------------------------------------------------------------------------------ :    -
AOTVOKIR2DL1*01v1 : ------------------------------------------------------------------------------------------------ :    -
AOTVOKIR3DS1*01v2 : ------------------------------------------------------------------------------------------------ :    -
AOTVOKIR3DS1*02   : ------------------------------------------------------------------------------------------------ :    -
AOTVOKIR3DL2*01   : ------------------------------------------------------------------------------------------------ :    -
AOTVOKIR3DL2*01v1 : ------------------------------------------------------------------------------------------------ :    -
AOTVOKIR3DL2*02   : ------------------------------------------------------------------------------------------------ :    -
AOTVOKIR3DS3*01   : ------------------------------------------------------------------------------------------------ :    -
```

```
AOTVOKIR3DS3*02   : ------------------------------------------------------------------------------------------------ :     -
AOTVOKIR3DS3*03   : ------------------------------------------------------------------------------------------------ :     -
AOTVOKIR3DS3*03v1 : ------------------------------------------------------------------------------------------------ :     -

                         *      3280         *      3300         *      3320         *      3340         *      3360
BAC_clone_Om      : GCACAGGGTAGAAGTTTTATTTCTGCATTACCTCCACAAAGTGTTCTGTACCAGGAGAATCCAAAGAAACACACATTTCTGGCCTGAGTTGGACCC :  3354
AOTVOKIR3DL1*01   : ------------------------------------------------------------------------------------------------ :     -
AOTVOKIR2DL1*01v1 : ------------------------------------------------------------------------------------------------ :     -
AOTVOKIR3DS1*01v2 : ------------------------------------------------------------------------------------------------ :     -
AOTVOKIR3DS1*02   : ------------------------------------------------------------------------------------------------ :     -
AOTVOKIR3DL2*01   : ------------------------------------------------------------------------------------------------ :     -
AOTVOKIR3DL2*01v1 : ------------------------------------------------------------------------------------------------ :     -
AOTVOKIR3DL2*02   : ------------------------------------------------------------------------------------------------ :     -
AOTVOKIR3DS3*01   : ------------------------------------------------------------------------------------------------ :     -
AOTVOKIR3DS3*02   : ------------------------------------------------------------------------------------------------ :     -
AOTVOKIR3DS3*03   : ------------------------------------------------------------------------------------------------ :     -
AOTVOKIR3DS3*03v1 : ------------------------------------------------------------------------------------------------ :     -

                             *      3380         *      3400         *      3420         *      3440         *
BAC_clone_Om      : TGTGGCCTCAGGCCTTGTGGCACCTACAGATGCCGTGTTCATTATGACACCTCTGCCTTCCGTGCAGTGGAGCCTTAATCGTCCCAGGATATCATG :  3450
AOTVOKIR3DL1*01   : ------------------------------------------------------------------------------------------------ :     -
AOTVOKIR2DL1*01v1 : ------------------------------------------------------------------------------------------------ :     -
AOTVOKIR3DS1*01v2 : ------------------------------------------------------------------------------------------------ :     -
AOTVOKIR3DS1*02   : ------------------------------------------------------------------------------------------------ :     -
AOTVOKIR3DL2*01   : ------------------------------------------------------------------------------------------------ :     -
AOTVOKIR3DL2*01v1 : ------------------------------------------------------------------------------------------------ :     -
AOTVOKIR3DL2*02   : ------------------------------------------------------------------------------------------------ :     -
AOTVOKIR3DS3*01   : ------------------------------------------------------------------------------------------------ :     -
AOTVOKIR3DS3*02   : ------------------------------------------------------------------------------------------------ :     -
AOTVOKIR3DS3*03   : ------------------------------------------------------------------------------------------------ :     -
AOTVOKIR3DS3*03v1 : ------------------------------------------------------------------------------------------------ :     -

                    3460         *      3480         *      3500         *      3520         *      3540         *
BAC_clone_Om      : GACCCAGAACACCAACCCCTGTGTGCTGTGTGTATTTGGGGTCACCAGATTGGATTCTGAGGCTCATATTCCAACTAATCTCACATATTGTGGCAT :  3546
AOTVOKIR3DL1*01   : ------------------------------------------------------------------------------------------------ :     -
AOTVOKIR2DL1*01v1 : ------------------------------------------------------------------------------------------------ :     -
AOTVOKIR3DS1*01v2 : ------------------------------------------------------------------------------------------------ :     -
AOTVOKIR3DS1*02   : ------------------------------------------------------------------------------------------------ :     -
AOTVOKIR3DL2*01   : ------------------------------------------------------------------------------------------------ :     -
AOTVOKIR3DL2*01v1 : ------------------------------------------------------------------------------------------------ :     -
AOTVOKIR3DL2*02   : ------------------------------------------------------------------------------------------------ :     -
AOTVOKIR3DS3*01   : ------------------------------------------------------------------------------------------------ :     -
AOTVOKIR3DS3*02   : ------------------------------------------------------------------------------------------------ :     -
```

```
AOTVOKIR3DS3*03    : ------------------------------------------------------------------------------------------------ :    -
AOTVOKIR3DS3*03v1  : ------------------------------------------------------------------------------------------------ :    -

                         3560         *       3580         *       3600         *       3620         *       3640
BAC_clone_Om       : TACTGAGAGACACAGAGAGAAATCAGGGACATCAAAATGCAAAGACATAAACACACACAGAATGAGTCAGGGGAAGGGGATTGAGGGACTCACAGA :  3642
AOTVOKIR3DL1*01    : ------------------------------------------------------------------------------------------------ :    -
AOTVOKIR2DL1*01v1  : ------------------------------------------------------------------------------------------------ :    -
AOTVOKIR3DS1*01v2  : ------------------------------------------------------------------------------------------------ :    -
AOTVOKIR3DS1*02    : ------------------------------------------------------------------------------------------------ :    -
AOTVOKIR3DL2*01    : ------------------------------------------------------------------------------------------------ :    -
AOTVOKIR3DL2*01v1  : ------------------------------------------------------------------------------------------------ :    -
AOTVOKIR3DL2*02    : ------------------------------------------------------------------------------------------------ :    -
AOTVOKIR3DS3*01    : ------------------------------------------------------------------------------------------------ :    -
AOTVOKIR3DS3*02    : ------------------------------------------------------------------------------------------------ :    -
AOTVOKIR3DS3*03    : ------------------------------------------------------------------------------------------------ :    -
AOTVOKIR3DS3*03v1  : ------------------------------------------------------------------------------------------------ :    -

                     *       3660         *       3680         *       3700         *       3720         *       3740
BAC_clone_Om       : CACATAAAGAGAGAGAAAAGAGGGCAGAGAAGTGCAGGGAATGATGGACAGGAGCAGAGAAAAGCACTAAAATCAGGGCCCTGAGGGAGGGGCACA :  3738
AOTVOKIR3DL1*01    : ------------------------------------------------------------------------------------------------ :    -
AOTVOKIR2DL1*01v1  : ------------------------------------------------------------------------------------------------ :    -
AOTVOKIR3DS1*01v2  : ------------------------------------------------------------------------------------------------ :    -
AOTVOKIR3DS1*02    : ------------------------------------------------------------------------------------------------ :    -
AOTVOKIR3DL2*01    : ------------------------------------------------------------------------------------------------ :    -
AOTVOKIR3DL2*01v1  : ------------------------------------------------------------------------------------------------ :    -
AOTVOKIR3DL2*02    : ------------------------------------------------------------------------------------------------ :    -
AOTVOKIR3DS3*01    : ------------------------------------------------------------------------------------------------ :    -
AOTVOKIR3DS3*02    : ------------------------------------------------------------------------------------------------ :    -
AOTVOKIR3DS3*03    : ------------------------------------------------------------------------------------------------ :    -
AOTVOKIR3DS3*03v1  : ------------------------------------------------------------------------------------------------ :    -

                         *       3760         *       3780         *       3800         *       3820         *       3840
BAC_clone_Om       : GGGAGAGAGAAAGATGGGGATGTGGGATGGATTGCAGAGATTCCAAAGAGAACTAGAGAGACGAGAGGCAGAGAAAGACAAGGAGGTGGAGACACA :  3834
AOTVOKIR3DL1*01    : ------------------------------------------------------------------------------------------------ :    -
AOTVOKIR2DL1*01v1  : ------------------------------------------------------------------------------------------------ :    -
AOTVOKIR3DS1*01v2  : ------------------------------------------------------------------------------------------------ :    -
AOTVOKIR3DS1*02    : ------------------------------------------------------------------------------------------------ :    -
AOTVOKIR3DL2*01    : ------------------------------------------------------------------------------------------------ :    -
AOTVOKIR3DL2*01v1  : ------------------------------------------------------------------------------------------------ :    -
AOTVOKIR3DL2*02    : ------------------------------------------------------------------------------------------------ :    -
AOTVOKIR3DS3*01    : ------------------------------------------------------------------------------------------------ :    -
AOTVOKIR3DS3*02    : ------------------------------------------------------------------------------------------------ :    -
AOTVOKIR3DS3*03    : ------------------------------------------------------------------------------------------------ :    -
```

```
AOTVOKIR3DS3*03v1 : ------------------------------------------------------------------------------------------------ :     -

                             *      3860         *      3880         *      3900         *      3920         *
BAC_clone_Om      : GATGACAGATGGAGAGATACAGATAGATAGATGATAAATAGGTAGATGGTAGATCATAGAGAGGTTATAGATACATGATGATTGACAGATGATACA :  3930
AOTVOKIR3DL1*01   : ------------------------------------------------------------------------------------------------ :     -
AOTVOKIR2DL1*01v1 : ------------------------------------------------------------------------------------------------ :     -
AOTVOKIR3DS1*01v2 : ------------------------------------------------------------------------------------------------ :     -
AOTVOKIR3DS1*02   : ------------------------------------------------------------------------------------------------ :     -
AOTVOKIR3DL2*01   : ------------------------------------------------------------------------------------------------ :     -
AOTVOKIR3DL2*01v1 : ------------------------------------------------------------------------------------------------ :     -
AOTVOKIR3DL2*02   : ------------------------------------------------------------------------------------------------ :     -
AOTVOKIR3DS3*01   : ------------------------------------------------------------------------------------------------ :     -
AOTVOKIR3DS3*02   : ------------------------------------------------------------------------------------------------ :     -
AOTVOKIR3DS3*03   : ------------------------------------------------------------------------------------------------ :     -
AOTVOKIR3DS3*03v1 : ------------------------------------------------------------------------------------------------ :     -

                    3940         *      3960         *      3980         *      4000         *      4020         *
BAC_clone_Om      : CAGGGATGATGATGATTATGATGATAGATGATACAAAGGTAGAGAGGTAGACAGAGAGATAATAGAGTGATAGATAATGCACCGATATAGATAATA :  4026
AOTVOKIR3DL1*01   : ------------------------------------------------------------------------------------------------ :     -
AOTVOKIR2DL1*01v1 : ------------------------------------------------------------------------------------------------ :     -
AOTVOKIR3DS1*01v2 : ------------------------------------------------------------------------------------------------ :     -
AOTVOKIR3DS1*02   : ------------------------------------------------------------------------------------------------ :     -
AOTVOKIR3DL2*01   : ------------------------------------------------------------------------------------------------ :     -
AOTVOKIR3DL2*01v1 : ------------------------------------------------------------------------------------------------ :     -
AOTVOKIR3DL2*02   : ------------------------------------------------------------------------------------------------ :     -
AOTVOKIR3DS3*01   : ------------------------------------------------------------------------------------------------ :     -
AOTVOKIR3DS3*02   : ------------------------------------------------------------------------------------------------ :     -
AOTVOKIR3DS3*03   : ------------------------------------------------------------------------------------------------ :     -
AOTVOKIR3DS3*03v1 : ------------------------------------------------------------------------------------------------ :     -

                        4040         *      4060         *      4080         *      4100         *      4120
BAC_clone_Om      : GATAGACAACCTGTGGATGGAACCAAATAGATAGATAGATCAATAGATAATAGATAGAAATATGCAGTTATGAACTGGACAGTGAGAGACTGAGAA :  4122
AOTVOKIR3DL1*01   : ------------------------------------------------------------------------------------------------ :     -
AOTVOKIR2DL1*01v1 : ------------------------------------------------------------------------------------------------ :     -
AOTVOKIR3DS1*01v2 : ------------------------------------------------------------------------------------------------ :     -
AOTVOKIR3DS1*02   : ------------------------------------------------------------------------------------------------ :     -
AOTVOKIR3DL2*01   : ------------------------------------------------------------------------------------------------ :     -
AOTVOKIR3DL2*01v1 : ------------------------------------------------------------------------------------------------ :     -
AOTVOKIR3DL2*02   : ------------------------------------------------------------------------------------------------ :     -
AOTVOKIR3DS3*01   : ------------------------------------------------------------------------------------------------ :     -
AOTVOKIR3DS3*02   : ------------------------------------------------------------------------------------------------ :     -
AOTVOKIR3DS3*03   : ------------------------------------------------------------------------------------------------ :     -
AOTVOKIR3DS3*03v1 : ------------------------------------------------------------------------------------------------ :     -
```

```
                              *        4140          *        4160          *        4180          *        4200          *        4220
BAC_clone_Om         : TTAAAGAAAAAGGAAGATCAAGTCAACCAATTCAAGGAGGGTCAGAGAGAATAAAACAAAAATGGAAAACATACCCGGGGTGGGGAAGTGAGTGGA :  4218
AOTVOKIR3DL1*01      : ------------------------------------------------------------------------------------------------ :     -
AOTVOKIR2DL1*01v1    : ------------------------------------------------------------------------------------------------ :     -
AOTVOKIR3DS1*01v2    : ------------------------------------------------------------------------------------------------ :     -
AOTVOKIR3DS1*02      : ------------------------------------------------------------------------------------------------ :     -
AOTVOKIR3DL2*01      : ------------------------------------------------------------------------------------------------ :     -
AOTVOKIR3DL2*01v1    : ------------------------------------------------------------------------------------------------ :     -
AOTVOKIR3DL2*02      : ------------------------------------------------------------------------------------------------ :     -
AOTVOKIR3DS3*01      : ------------------------------------------------------------------------------------------------ :     -
AOTVOKIR3DS3*02      : ------------------------------------------------------------------------------------------------ :     -
AOTVOKIR3DS3*03      : ------------------------------------------------------------------------------------------------ :     -
AOTVOKIR3DS3*03v1    : ------------------------------------------------------------------------------------------------ :     -

                                  *        4240          *        4260          *        4280          *        4300          *        4320
BAC_clone_Om         : GAGACAGGGAAGGTAGAAGGAGAAAACAGACAGAAGAGAGACGGGGTGGAGGGTAAGAGAGAGCATCAGCTCATGGAGCAGGGGAGTGAGTTCTCA :  4314
AOTVOKIR3DL1*01      : ------------------------------------------------------------------------------------------------ :     -
AOTVOKIR2DL1*01v1    : ------------------------------------------------------------------------------------------------ :     -
AOTVOKIR3DS1*01v2    : ------------------------------------------------------------------------------------------------ :     -
AOTVOKIR3DS1*02      : ------------------------------------------------------------------------------------------------ :     -
AOTVOKIR3DL2*01      : ------------------------------------------------------------------------------------------------ :     -
AOTVOKIR3DL2*01v1    : ------------------------------------------------------------------------------------------------ :     -
AOTVOKIR3DL2*02      : ------------------------------------------------------------------------------------------------ :     -
AOTVOKIR3DS3*01      : ------------------------------------------------------------------------------------------------ :     -
AOTVOKIR3DS3*02      : ------------------------------------------------------------------------------------------------ :     -
AOTVOKIR3DS3*03      : ------------------------------------------------------------------------------------------------ :     -
AOTVOKIR3DS3*03v1    : ------------------------------------------------------------------------------------------------ :     -

                                                                                                              EXON 5
                                      *        4340          *        4360          *        4380          *        4400          *
BAC_clone_Om         : GCTCAGCTGTGAAGGGAGCTGTGACAAGGAAGAGCCTCCTGAGCAAACTGCCTCTTCTCCTTCCAGGTCTATATGAGAAACCTTCTCTCTCAGCCC :  4410
AOTVOKIR3DL1*01      : ------------------------------------------------------------------GTCTATATGAGAAGCCTTCTCTCTCAGCCC :   682
AOTVOKIR2DL1*01v1    : ------------------------------------------------------------------------------------------------ :     -
AOTVOKIR3DS1*01v2    : ------------------------------------------------------------------GTCTATATGAGAAGCCTTCTCTCTCAGCCC :   682
AOTVOKIR3DS1*02      : ------------------------------------------------------------------GTCTATATGAGAAGCCTTCTCTCTCAGCCC :   682
AOTVOKIR3DL2*01      : ------------------------------------------------------------------GTCTATATGACAAACCTTCGCTCTCAGCCC :   682
AOTVOKIR3DL2*01v1    : ------------------------------------------------------------------GTCTATATGACAAACCTTCGCTCTCAGCCC :   646
AOTVOKIR3DL2*02      : ------------------------------------------------------------------GTCTATATGACAAACCTTCGCTCTCAGCCC :   682
AOTVOKIR3DS3*01      : ------------------------------------------------------------------GTCTATATGAGAAACCTTCTCTCTCAGCCC :   646
AOTVOKIR3DS3*02      : ------------------------------------------------------------------GTCTATATGAGAAACCTTCTCTCTCAGCCC :   646
AOTVOKIR3DS3*03      : ------------------------------------------------------------------GTCTATATGAGAAACCTTCTCTCTCAGCCC :   664
AOTVOKIR3DS3*03v1    : ------------------------------------------------------------------GTCTATATGAGAAACCTTCTCTCTCAGCCC :   664
                                                                                         gtctatatga aa ccttc ctctcagccc
```

EXON 5

```
                       4420        *       4440        *       4460        *       4480        *       4500        *
BAC_clone_Om       : AGCCGGGCCCTACGGTTCAGGCAGGAGAGAATGTGACCTTGTCCTGCAGCTCCTGGAGCTGGTTCGACATGTACCATCTAACTAGGGAGGGGGAGG :  4506
AOTVOKIR3DL1*01    : AGCCGGGCCCCACGCTTCAGGCAGGAGAGAATGTGACCTTGTCCTGCAGCTCCCGGAGCTGGTTCGACATGTACCATCTAACCAGGGAGGGGGAGG :   778
AOTVOKIR2DL1*01v1  : ------------------------------------------------------------------------------------------------ :     -
AOTVOKIR3DS1*01v2  : AGCCGGGCCCCACGCTTCAGGCAGGAGAGAATGTGACCTTGTCCTGCAGCTCCCGGAGCTGGTTCGACATGTACCATCTAACCAGGGAGGGGGAGG :   778
AOTVOKIR3DS1*02    : AGCCGGGCCCCACGCTTCAGGCAGGAGAGAATGTGACCTTGTCCTGCAGCTCCCGGAGCTGGTTCGACATGTACCATCTAACCAGGGAGGGGGAGG :   778
AOTVOKIR3DL2*01    : AGCCGGACCCCACGGTTCAGGCAGGAGAGAACGTGACCTTGTCCTGCAGCTCCTGGAGCTGGTTCGACATGTACCATCTAACTAGGGAGGGGGAGG :   778
AOTVOKIR3DL2*01v1  : AGCCGGACCCCACGGTTCAGGCAGGAGAGAACGTGACCTTGTCCTGCAGCTCCTGGAGCTGGTTCGACATGTACCATCTAACTAGGGAGGGGGAGG :   742
AOTVOKIR3DL2*02    : AGCCGGACCCCACGGTTCAGGCAGGAGAGAACGTGACCTTGTCCTGCAGCTCCCGGAGCTGGTTCGACATGTACCATCTAACTAGGGAGGGGGAGG :   778
AOTVOKIR3DS3*01    : AGCTGGGCCCCACGCTTCAGCCAGGAGAGAACGTGACCTTGTCCTGCAGCTCCCGGAGCTGGTTCGACATGTACCATCTAACCAGGGAGTGGGAGG :   742
AOTVOKIR3DS3*02    : AGCTGGGCCCCACGCTTCAGCCAGGAGAGAACGTGACCTTGTCCTGCAGCTCCCGGAGCTGGTTCGACATGTACCATCTAACCAGGGAGTGGGAGG :   742
AOTVOKIR3DS3*03    : AGCTGGGCCCCACGCTTCAGCCAGGAGAGAACGTGACCTTGTCCTGCAGCTCCCGGAGCTGGTTCGACATGTACCATCTAACCAGGGAGTGGGAGG :   760
AOTVOKIR3DS3*03v1  : AGCTGGGCCCCACGCTTCAGCCAGGAGAGAACGTGACCTTGTCCTGCAGCTCCCGGAGCTGGTTCGACATGTACCATCTAACCAGGGAGTGGGAGG :   760
                     agc gg ccccacg ttcag caggagagaa gtgaccttgtcctgcagctcc ggagctggttcgacatgtaccatctaac agggag gggagg

                           4520        *       4540        *       4560        *       4580        *       4600
BAC_clone_Om       : CCCCTGAACTCAGGCTCCCTGCAGTGCCCAGCATCAATGGAACATTCCAGGCCCACTTCCCTCTGGGCCCTGCCACCCACGGAGGGTCCTACAGAT :  4602
AOTVOKIR3DL1*01    : CCCCTGAACTCAGGCTCCCTGCAGTGCCCAGCATCAGTGGAACATTCCAGGCCCACTTCCCTCTGGGCCCTGCCACCCACGGAGGGACCTACAGAT :   874
AOTVOKIR2DL1*01v1  : ------------------------------------------------------------------------------------------------ :     -
AOTVOKIR3DS1*01v2  : CCCCTGAACTCAGGCTCCCTGCAGTGCCCAGCATCAGTGGAACATTCCAGGCCCACTTCCCTCTGGGCCCTGCCACCCACGGAGGGACCTACAGAT :   874
AOTVOKIR3DS1*02    : CCCCTGAACTCAGGCTCCCTGCAGTGCCCAGCATCAGTGGAACATTCCAGGCCCACTTCCCTCTGGGCCCTGCCACCCACGGAGGGACCTACAGAT :   874
AOTVOKIR3DL2*01    : CCCCTGAACTCAGGGTCACTGCAGTGCCCAGCATCAATGGAACATTCCAGGCCCACTTCCCTCTGGGCCCTGCCACCCACGGAGGGACCTACAGAT :   874
AOTVOKIR3DL2*01v1  : CCCCTGAACTCAGGGTCACTGCAGTGCCCAGCATCAATGGAACATTCCAGGCCCACTTCCCTCTGGGCCCTGCCACCCACGGAGGGACCTACAGAT :   838
AOTVOKIR3DL2*02    : CCCCTGAACTCAGGGTCACTGCAGTGCCCAGCATCAATGGAACATTCCAGGCCCACTTCCCTCTGGGCCCTGCCACCCACGGAGGGACCTACAGAT :   874
AOTVOKIR3DS3*01    : CCCCTGAACTTAGGCTTCCTGCAGTGCCCAGCATCAATGGAACGTTCCAGGCCCACTTCCCTCTGGGCCCTGCCACCCATGGAGGGACCTACAGAT :   838
AOTVOKIR3DS3*02    : CCCCTGAACTTAGGCTTCCTGCAGTGCCCAGCATCAATGGAACGTTCCAGGCCCACTTCCCTCTGGGCCCTGCCACCCATGGAGGGACCTACAGAT :   838
AOTVOKIR3DS3*03    : CCCCTGAACTTAGGCTTCCTGCAGTGCCCAGCATCAATGGAACGTTCCAGGCCCACTTCCCTCTGGGCCCTGCCACCCATGGAGGGACCTACAGAT :   856
AOTVOKIR3DS3*03v1  : CCCCTGAACTTAGGCTTCCTGCAGTGCCCAGCATCAATGGAACGTTCCAGGCCCACTTCCCTCTGGGCCCTGCCACCCATGGAGGGACCTACAGAT :   856
                     cccctgaact agg t  ctgcagtgcccagcatca tggaac ttccaggcccacttccctctgggccctgccaccca ggagggacctacagat

                      *       4620        *       4640        *       4660        *       4680        *       4700
BAC_clone_Om       : GCTTCGGCTCTTTCTGTGACTCTCCCTACGAGTGGTCAGCCCCGAGTGACCCACTGTCCGTTTCTGTCACAGGTGAGGAAACCCCATACCTGTCCC :  4698
AOTVOKIR3DL1*01    : GCTTCGGCTCTTTCCGTGACTCTCCCTACGAGTGGTCAGCCCCGAGTGACCCACTGTCCGTTTCTGTCACAG------------------------ :   946
AOTVOKIR2DL1*01v1  : ------------------------------------------------------------------------------------------------ :     -
AOTVOKIR3DS1*01v2  : GCTTCGGCTCTTTCCGTGACTCTCCCTACGAGTGGTCAGCCCCGAGTGACCCACTGTCCGTTTCTGTCACAG------------------------ :   946
AOTVOKIR3DS1*02    : GCTTCGGCTCTTTCCGTGACTCTCCCTACGAGTGGTCAGCCCCGAGTGACCCACTGTCCGTTTCTGTCACAG------------------------ :   946
AOTVOKIR3DL2*01    : GCTTCAGCTCTTTCCGTGACTCTCCCTACGAGTGGTCAGCCCCGAGTGACCCACTGTCCGTTTCTGTCACAG------------------------ :   946
AOTVOKIR3DL2*01v1  : GCTTCAGCTCTTTCCGTGACTCTCCCTACGAGTGGTCAGCCCCGAGTGACCCACTGTCCGTTTCTGTCACAG------------------------ :   910
AOTVOKIR3DL2*02    : GCTTCAGCTCTTTCCGTGACTCTCCCTACGAGTGGTCAGCCCCGAGTGACCCACTGTCCGTTTCTGTCACAG------------------------ :   946
AOTVOKIR3DS3*01    : GCTTCGGCTCTTTCCGTGACTCTCCCTATGAGTGGTCAGCCCCGAGTGACCAACTGTCTGTTTCTGTCACAG------------------------ :   910
AOTVOKIR3DS3*02    : GCTTCGGCTCTTTCCGTGACTCTCCCTATGAGTGGTCAGCCCCGAGTGACCAACTGTCTGTTTCTGTCACAG------------------------ :   910
AOTVOKIR3DS3*03    : GCTTCGGCTCTTTCCGTGACTCTCCCTATGAGTGGTCAGCCCCGAGTGACCAACTGTCTGTTTCTGTCACAG------------------------ :   928
AOTVOKIR3DS3*03v1  : GCTTCGGCTCTTTCCGTGACTCTCCCTATGAGTGGTCAGCCCCGAGTGACCAACTGTCTGTTTCTGTCACAG------------------------ :   928
                     gcttc gctctttccgtgactctcccta gagtggtcagccccgagtgacc actgtc gtttctgtcacag
```

```
                          *      4720         *      4740         *      4760         *      4780         *      4800
BAC_clone_Om       : ATGTCTGATGATCCCAGAGCCATAGCTGAGGAACTTCCTGCTGATGATGGAGGGAAGCGTGGACAGATGCAGAGAGAAGACGAAGCCTGGGTGTGA :  4794
AOTVOKIR3DL1*01    : ------------------------------------------------------------------------------------------------ :     -
AOTVOKIR2DL1*01v1  : ------------------------------------------------------------------------------------------------ :     -
AOTVOKIR3DS1*01v2  : ------------------------------------------------------------------------------------------------ :     -
AOTVOKIR3DS1*02    : ------------------------------------------------------------------------------------------------ :     -
AOTVOKIR3DL2*01    : ------------------------------------------------------------------------------------------------ :     -
AOTVOKIR3DL2*01v1  : ------------------------------------------------------------------------------------------------ :     -
AOTVOKIR3DL2*02    : ------------------------------------------------------------------------------------------------ :     -
AOTVOKIR3DS3*01    : ------------------------------------------------------------------------------------------------ :     -
AOTVOKIR3DS3*02    : ------------------------------------------------------------------------------------------------ :     -
AOTVOKIR3DS3*03    : ------------------------------------------------------------------------------------------------ :     -
AOTVOKIR3DS3*03v1  : ------------------------------------------------------------------------------------------------ :     -

                              *      4820         *      4840         *      4860         *      4880         *
BAC_clone_Om       : GGGCGGGGTCAGGGCACAGGATGGCAGACAGGGCACCTCCAGGCCCTCCTCCACGGCCTGCATGGAGGCCCGCGTTCAGGGCTCCGGGCACCCAGG :  4890
AOTVOKIR3DL1*01    : ------------------------------------------------------------------------------------------------ :     -
AOTVOKIR2DL1*01v1  : ------------------------------------------------------------------------------------------------ :     -
AOTVOKIR3DS1*01v2  : ------------------------------------------------------------------------------------------------ :     -
AOTVOKIR3DS1*02    : ------------------------------------------------------------------------------------------------ :     -
AOTVOKIR3DL2*01    : ------------------------------------------------------------------------------------------------ :     -
AOTVOKIR3DL2*01v1  : ------------------------------------------------------------------------------------------------ :     -
AOTVOKIR3DL2*02    : ------------------------------------------------------------------------------------------------ :     -
AOTVOKIR3DS3*01    : ------------------------------------------------------------------------------------------------ :     -
AOTVOKIR3DS3*02    : ------------------------------------------------------------------------------------------------ :     -
AOTVOKIR3DS3*03    : ------------------------------------------------------------------------------------------------ :     -
AOTVOKIR3DS3*03v1  : ------------------------------------------------------------------------------------------------ :     -

                     4900         *      4920         *      4940         *      4960         *      4980         *
BAC_clone_Om       : CAGATGGAGAAACGGTCAGGACAGGCCCAGAGGAGGGGAGACGGGGCTCAGTTTGGGGAGATCAGAGGTTACCTCAGCCCCTCAAACTCACCCATT :  4986
AOTVOKIR3DL1*01    : ------------------------------------------------------------------------------------------------ :     -
AOTVOKIR2DL1*01v1  : ------------------------------------------------------------------------------------------------ :     -
AOTVOKIR3DS1*01v2  : ------------------------------------------------------------------------------------------------ :     -
AOTVOKIR3DS1*02    : ------------------------------------------------------------------------------------------------ :     -
AOTVOKIR3DL2*01    : ------------------------------------------------------------------------------------------------ :     -
AOTVOKIR3DL2*01v1  : ------------------------------------------------------------------------------------------------ :     -
AOTVOKIR3DL2*02    : ------------------------------------------------------------------------------------------------ :     -
AOTVOKIR3DS3*01    : ------------------------------------------------------------------------------------------------ :     -
AOTVOKIR3DS3*02    : ------------------------------------------------------------------------------------------------ :     -
AOTVOKIR3DS3*03    : ------------------------------------------------------------------------------------------------ :     -
AOTVOKIR3DS3*03v1  : ------------------------------------------------------------------------------------------------ :     -

                         5000         *      5020         *      5040         *      5060         *      5080
```

```
BAC_clone_Om       : TCCCAGAAGCCCATCCTGGCCTCTCACCCACAGAGAGATGTCATCATCAGCAACCCCCATGTCCTTTTCTTTTCATTTTCAAAAATATTTACTAAG :  5082
AOTVOKIR3DL1*01    : ------------------------------------------------------------------------------------------------ :     -
AOTVOKIR2DL1*01v1  : ------------------------------------------------------------------------------------------------ :     -
AOTVOKIR3DS1*01v2  : ------------------------------------------------------------------------------------------------ :     -
AOTVOKIR3DS1*02    : ------------------------------------------------------------------------------------------------ :     -
AOTVOKIR3DL2*01    : ------------------------------------------------------------------------------------------------ :     -
AOTVOKIR3DL2*01v1  : ------------------------------------------------------------------------------------------------ :     -
AOTVOKIR3DL2*02    : ------------------------------------------------------------------------------------------------ :     -
AOTVOKIR3DS3*01    : ------------------------------------------------------------------------------------------------ :     -
AOTVOKIR3DS3*02    : ------------------------------------------------------------------------------------------------ :     -
AOTVOKIR3DS3*03    : ------------------------------------------------------------------------------------------------ :     -
AOTVOKIR3DS3*03v1  : ------------------------------------------------------------------------------------------------ :     -

                       *      5100          *      5120          *      5140          *      5160          *      5180
BAC_clone_Om       : GTTACATAATTTACCACCTGTACCATCATTTTTAAGTGTAAAGTCCAGTGGTCATAAATACATTGATAAGGTTGGCGCGGTGGCTCACGCCTGTAA :  5178
AOTVOKIR3DL1*01    : ------------------------------------------------------------------------------------------------ :     -
AOTVOKIR2DL1*01v1  : ------------------------------------------------------------------------------------------------ :     -
AOTVOKIR3DS1*01v2  : ------------------------------------------------------------------------------------------------ :     -
AOTVOKIR3DS1*02    : ------------------------------------------------------------------------------------------------ :     -
AOTVOKIR3DL2*01    : ------------------------------------------------------------------------------------------------ :     -
AOTVOKIR3DL2*01v1  : ------------------------------------------------------------------------------------------------ :     -
AOTVOKIR3DL2*02    : ------------------------------------------------------------------------------------------------ :     -
AOTVOKIR3DS3*01    : ------------------------------------------------------------------------------------------------ :     -
AOTVOKIR3DS3*02    : ------------------------------------------------------------------------------------------------ :     -
AOTVOKIR3DS3*03    : ------------------------------------------------------------------------------------------------ :     -
AOTVOKIR3DS3*03v1  : ------------------------------------------------------------------------------------------------ :     -

                           *      5200          *      5220          *      5240          *      5260          *      5280
BAC_clone_Om       : TCCCAGCACTTTGAGAGGCCAAGACAGGTGGATCATTTGACATCCGGGGCTCAAAACCACTCTCGCCAACATGGGGGAAATCACATCTTTACTAAA :  5274
AOTVOKIR3DL1*01    : ------------------------------------------------------------------------------------------------ :     -
AOTVOKIR2DL1*01v1  : ------------------------------------------------------------------------------------------------ :     -
AOTVOKIR3DS1*01v2  : ------------------------------------------------------------------------------------------------ :     -
AOTVOKIR3DS1*02    : ------------------------------------------------------------------------------------------------ :     -
AOTVOKIR3DL2*01    : ------------------------------------------------------------------------------------------------ :     -
AOTVOKIR3DL2*01v1  : ------------------------------------------------------------------------------------------------ :     -
AOTVOKIR3DL2*02    : ------------------------------------------------------------------------------------------------ :     -
AOTVOKIR3DS3*01    : ------------------------------------------------------------------------------------------------ :     -
AOTVOKIR3DS3*02    : ------------------------------------------------------------------------------------------------ :     -
AOTVOKIR3DS3*03    : ------------------------------------------------------------------------------------------------ :     -
AOTVOKIR3DS3*03v1  : ------------------------------------------------------------------------------------------------ :     -

                               *      5300          *      5320          *      5340          *      5360          *
BAC_clone_Om       : AATACGAAAAAAAAATTAACCAGGAATGGTGGCATGTGCCTATAGTCTCAGCTACTTGGGAGGCTGAGGCATGAGAACTGTGTAAGCCCAGGAGAC :  5370
```

```
AOTVOKIR3DL1*01    : ------------------------------------------------------------------------------------------------ :     -
AOTVOKIR2DL1*01v1  : ------------------------------------------------------------------------------------------------ :     -
AOTVOKIR3DS1*01v2  : ------------------------------------------------------------------------------------------------ :     -
AOTVOKIR3DS1*02    : ------------------------------------------------------------------------------------------------ :     -
AOTVOKIR3DL2*01    : ------------------------------------------------------------------------------------------------ :     -
AOTVOKIR3DL2*01v1  : ------------------------------------------------------------------------------------------------ :     -
AOTVOKIR3DL2*02    : ------------------------------------------------------------------------------------------------ :     -
AOTVOKIR3DS3*01    : ------------------------------------------------------------------------------------------------ :     -
AOTVOKIR3DS3*02    : ------------------------------------------------------------------------------------------------ :     -
AOTVOKIR3DS3*03    : ------------------------------------------------------------------------------------------------ :     -
AOTVOKIR3DS3*03v1  : ------------------------------------------------------------------------------------------------ :     -

                     5380         *      5400         *      5420         *      5440         *      5460         *
BAC_clone_Om       : AGAGGTTACAGTGATCCGAAATCATACCACTGTTCTGTAGCCTGGGTGACAGACAGAGACACTGTCTCAGCAAATAAATAAATAAATATTTTTATA :  5466
AOTVOKIR3DL1*01    : ------------------------------------------------------------------------------------------------ :     -
AOTVOKIR2DL1*01v1  : ------------------------------------------------------------------------------------------------ :     -
AOTVOKIR3DS1*01v2  : ------------------------------------------------------------------------------------------------ :     -
AOTVOKIR3DS1*02    : ------------------------------------------------------------------------------------------------ :     -
AOTVOKIR3DL2*01    : ------------------------------------------------------------------------------------------------ :     -
AOTVOKIR3DL2*01v1  : ------------------------------------------------------------------------------------------------ :     -
AOTVOKIR3DL2*02    : ------------------------------------------------------------------------------------------------ :     -
AOTVOKIR3DS3*01    : ------------------------------------------------------------------------------------------------ :     -
AOTVOKIR3DS3*02    : ------------------------------------------------------------------------------------------------ :     -
AOTVOKIR3DS3*03    : ------------------------------------------------------------------------------------------------ :     -
AOTVOKIR3DS3*03v1  : ------------------------------------------------------------------------------------------------ :     -

                         5480         *      5500         *      5520         *      5540         *      5560
BAC_clone_Om       : TTCTTTTTTTCGTTGTTACCCTCCACCATTCCCTTCCTGGCCTCTGAGAGCCACCATTCTCTCTACCTTCATGAGGTCCACCTGTTAGCTCCTGCA :  5562
AOTVOKIR3DL1*01    : ------------------------------------------------------------------------------------------------ :     -
AOTVOKIR2DL1*01v1  : ------------------------------------------------------------------------------------------------ :     -
AOTVOKIR3DS1*01v2  : ------------------------------------------------------------------------------------------------ :     -
AOTVOKIR3DS1*02    : ------------------------------------------------------------------------------------------------ :     -
AOTVOKIR3DL2*01    : ------------------------------------------------------------------------------------------------ :     -
AOTVOKIR3DL2*01v1  : ------------------------------------------------------------------------------------------------ :     -
AOTVOKIR3DL2*02    : ------------------------------------------------------------------------------------------------ :     -
AOTVOKIR3DS3*01    : ------------------------------------------------------------------------------------------------ :     -
AOTVOKIR3DS3*02    : ------------------------------------------------------------------------------------------------ :     -
AOTVOKIR3DS3*03    : ------------------------------------------------------------------------------------------------ :     -
AOTVOKIR3DS3*03v1  : ------------------------------------------------------------------------------------------------ :     -

                       *      5580         *      5600         *      5620         *      5640         *      5660
BAC_clone_Om       : TATGGGTGAGAAATGGCAATCTCTGTAATGCCCTCCAGTTCATCCATGTGTCTGAAAGTGACAGGATGTTATTGCTTTTATGGAGGAGTGGTCTCC :  5658
AOTVOKIR3DL1*01    : ------------------------------------------------------------------------------------------------ :     -
```

```
AOTVOKIR2DL1*01v1 : ------------------------------------------------------------------------------------------------ :    -
AOTVOKIR3DS1*01v2 : ------------------------------------------------------------------------------------------------ :    -
AOTVOKIR3DS1*02   : ------------------------------------------------------------------------------------------------ :    -
AOTVOKIR3DL2*01   : ------------------------------------------------------------------------------------------------ :    -
AOTVOKIR3DL2*01v1 : ------------------------------------------------------------------------------------------------ :    -
AOTVOKIR3DL2*02   : ------------------------------------------------------------------------------------------------ :    -
AOTVOKIR3DS3*01   : ------------------------------------------------------------------------------------------------ :    -
AOTVOKIR3DS3*02   : ------------------------------------------------------------------------------------------------ :    -
AOTVOKIR3DS3*03   : ------------------------------------------------------------------------------------------------ :    -
AOTVOKIR3DS3*03v1 : ------------------------------------------------------------------------------------------------ :    -

                         *      5680         *      5700         *      5720         *      5740         *      5760
BAC_clone_Om      : ATTGTGCGTATGTGCTACATTCTCTCTATCCGCTCATCCACTGATGGGCAGGTAGGTTGATGCCACGCCTTGGCTACTGTGAACAGTGCTGGGACA :  5754
AOTVOKIR3DL1*01   : ------------------------------------------------------------------------------------------------ :    -
AOTVOKIR2DL1*01v1 : ------------------------------------------------------------------------------------------------ :    -
AOTVOKIR3DS1*01v2 : ------------------------------------------------------------------------------------------------ :    -
AOTVOKIR3DS1*02   : ------------------------------------------------------------------------------------------------ :    -
AOTVOKIR3DL2*01   : ------------------------------------------------------------------------------------------------ :    -
AOTVOKIR3DL2*01v1 : ------------------------------------------------------------------------------------------------ :    -
AOTVOKIR3DL2*02   : ------------------------------------------------------------------------------------------------ :    -
AOTVOKIR3DS3*01   : ------------------------------------------------------------------------------------------------ :    -
AOTVOKIR3DS3*02   : ------------------------------------------------------------------------------------------------ :    -
AOTVOKIR3DS3*03   : ------------------------------------------------------------------------------------------------ :    -
AOTVOKIR3DS3*03v1 : ------------------------------------------------------------------------------------------------ :    -

                             *      5780         *      5800         *      5820         *      5840         *
BAC_clone_Om      : GTCGTGGGAGTGCAGATAACGCTTCAATACACTGATGTCCTTTCCTTTGGGTTTACACCAGTAGTGGAATTGCAAGATCCTATGGAAATTCTCTTT :  5850
AOTVOKIR3DL1*01   : ------------------------------------------------------------------------------------------------ :    -
AOTVOKIR2DL1*01v1 : ------------------------------------------------------------------------------------------------ :    -
AOTVOKIR3DS1*01v2 : ------------------------------------------------------------------------------------------------ :    -
AOTVOKIR3DS1*02   : ------------------------------------------------------------------------------------------------ :    -
AOTVOKIR3DL2*01   : ------------------------------------------------------------------------------------------------ :    -
AOTVOKIR3DL2*01v1 : ------------------------------------------------------------------------------------------------ :    -
AOTVOKIR3DL2*02   : ------------------------------------------------------------------------------------------------ :    -
AOTVOKIR3DS3*01   : ------------------------------------------------------------------------------------------------ :    -
AOTVOKIR3DS3*02   : ------------------------------------------------------------------------------------------------ :    -
AOTVOKIR3DS3*03   : ------------------------------------------------------------------------------------------------ :    -
AOTVOKIR3DS3*03v1 : ------------------------------------------------------------------------------------------------ :    -

                    5860         *      5880         *      5900         *      5920         *      5940         *
BAC_clone_Om      : TTAGGTTTTGTTTTGTGTTTCTTGTATTTTTGAGCCAGAGTTTCACTCTTCTTGCCCAGGATGGAGTGCAGTGGCACCATCTGGGCTCACTGCAAC :  5946
AOTVOKIR3DL1*01   : ------------------------------------------------------------------------------------------------ :    -
AOTVOKIR2DL1*01v1 : ------------------------------------------------------------------------------------------------ :    -
```

```
AOTVOKIR3DS1*01v2 : ------------------------------------------------------------------------------------------------ :     -
AOTVOKIR3DS1*02   : ------------------------------------------------------------------------------------------------ :     -
AOTVOKIR3DL2*01   : ------------------------------------------------------------------------------------------------ :     -
AOTVOKIR3DL2*01v1 : ------------------------------------------------------------------------------------------------ :     -
AOTVOKIR3DL2*02   : ------------------------------------------------------------------------------------------------ :     -
AOTVOKIR3DS3*01   : ------------------------------------------------------------------------------------------------ :     -
AOTVOKIR3DS3*02   : ------------------------------------------------------------------------------------------------ :     -
AOTVOKIR3DS3*03   : ------------------------------------------------------------------------------------------------ :     -
AOTVOKIR3DS3*03v1 : ------------------------------------------------------------------------------------------------ :     -

                              5960         *       5980         *       6000         *       6020         *       6040
BAC_clone_Om      : CTCCATCTCCTGGATTCCTGCGATTCTCCTGCCTCAGCCTCCCAAGTAGCTGAGATAACTGGCACCCACCACCATGCGCAGCCAGTTTTATTAGAG :  6042
AOTVOKIR3DL1*01   : ------------------------------------------------------------------------------------------------ :     -
AOTVOKIR2DL1*01v1 : ------------------------------------------------------------------------------------------------ :     -
AOTVOKIR3DS1*01v2 : ------------------------------------------------------------------------------------------------ :     -
AOTVOKIR3DS1*02   : ------------------------------------------------------------------------------------------------ :     -
AOTVOKIR3DL2*01   : ------------------------------------------------------------------------------------------------ :     -
AOTVOKIR3DL2*01v1 : ------------------------------------------------------------------------------------------------ :     -
AOTVOKIR3DL2*02   : ------------------------------------------------------------------------------------------------ :     -
AOTVOKIR3DS3*01   : ------------------------------------------------------------------------------------------------ :     -
AOTVOKIR3DS3*02   : ------------------------------------------------------------------------------------------------ :     -
AOTVOKIR3DS3*03   : ------------------------------------------------------------------------------------------------ :     -
AOTVOKIR3DS3*03v1 : ------------------------------------------------------------------------------------------------ :     -

                       *       6060         *       6080         *       6100         *       6120         *       6140
BAC_clone_Om      : ACTGGGTTTTGCCATGTTGGCCAGGCTGGTCTCAAACTCCTGACCTCAGGTGATCTACCCACCTTGGCCTCCCAAATTGCTGGGATTACAGGGGTG :  6138
AOTVOKIR3DL1*01   : ------------------------------------------------------------------------------------------------ :     -
AOTVOKIR2DL1*01v1 : ------------------------------------------------------------------------------------------------ :     -
AOTVOKIR3DS1*01v2 : ------------------------------------------------------------------------------------------------ :     -
AOTVOKIR3DS1*02   : ------------------------------------------------------------------------------------------------ :     -
AOTVOKIR3DL2*01   : ------------------------------------------------------------------------------------------------ :     -
AOTVOKIR3DL2*01v1 : ------------------------------------------------------------------------------------------------ :     -
AOTVOKIR3DL2*02   : ------------------------------------------------------------------------------------------------ :     -
AOTVOKIR3DS3*01   : ------------------------------------------------------------------------------------------------ :     -
AOTVOKIR3DS3*02   : ------------------------------------------------------------------------------------------------ :     -
AOTVOKIR3DS3*03   : ------------------------------------------------------------------------------------------------ :     -
AOTVOKIR3DS3*03v1 : ------------------------------------------------------------------------------------------------ :     -

                           *       6160         *       6180         *       6200         *       6220         *       6240
BAC_clone_Om      : AGCCACGGCTACCGAACGTTTTATTGTATTTATTTATTTTATTTTTAAATTTTTTTTTCTGTTCTCCAATTGTGGATAATTTTTCTGAGGCTTCAG :  6234
AOTVOKIR3DL1*01   : ------------------------------------------------------------------------------------------------ :     -
AOTVOKIR2DL1*01v1 : ------------------------------------------------------------------------------------------------ :     -
AOTVOKIR3DS1*01v2 : ------------------------------------------------------------------------------------------------ :     -
```

```
AOTVOKIR3DS1*02    : ------------------------------------------------------------------------------------------------ :     -
AOTVOKIR3DL2*01    : ------------------------------------------------------------------------------------------------ :     -
AOTVOKIR3DL2*01v1  : ------------------------------------------------------------------------------------------------ :     -
AOTVOKIR3DL2*02    : ------------------------------------------------------------------------------------------------ :     -
AOTVOKIR3DS3*01    : ------------------------------------------------------------------------------------------------ :     -
AOTVOKIR3DS3*02    : ------------------------------------------------------------------------------------------------ :     -
AOTVOKIR3DS3*03    : ------------------------------------------------------------------------------------------------ :     -
AOTVOKIR3DS3*03v1  : ------------------------------------------------------------------------------------------------ :     -

                                    *       6260         *       6280         *       6300         *       6320         *
BAC_clone_Om       : ATAATTCTGTTTATAATTATGTTTCTCTAACTTTGTGCAGAATTACTACAAAGGATATGAACACATAATGTGTCCACCAGGCTTCATTCTCTTTTT :  6330
AOTVOKIR3DL1*01    : ------------------------------------------------------------------------------------------------ :     -
AOTVOKIR2DL1*01v1  : ------------------------------------------------------------------------------------------------ :     -
AOTVOKIR3DS1*01v2  : ------------------------------------------------------------------------------------------------ :     -
AOTVOKIR3DS1*02    : ------------------------------------------------------------------------------------------------ :     -
AOTVOKIR3DL2*01    : ------------------------------------------------------------------------------------------------ :     -
AOTVOKIR3DL2*01v1  : ------------------------------------------------------------------------------------------------ :     -
AOTVOKIR3DL2*02    : ------------------------------------------------------------------------------------------------ :     -
AOTVOKIR3DS3*01    : ------------------------------------------------------------------------------------------------ :     -
AOTVOKIR3DS3*02    : ------------------------------------------------------------------------------------------------ :     -
AOTVOKIR3DS3*03    : ------------------------------------------------------------------------------------------------ :     -
AOTVOKIR3DS3*03v1  : ------------------------------------------------------------------------------------------------ :     -

                     6340         *       6360         *       6380         *       6400         *       6420         *
BAC_clone_Om       : TAAAATTAGTTTATTTTATTTAATATAACAATTAATAATTATTATTATATCAATATATTAATATTATTAATAATATATTATTTTAGAGACGGCATT :  6426
AOTVOKIR3DL1*01    : ------------------------------------------------------------------------------------------------ :     -
AOTVOKIR2DL1*01v1  : ------------------------------------------------------------------------------------------------ :     -
AOTVOKIR3DS1*01v2  : ------------------------------------------------------------------------------------------------ :     -
AOTVOKIR3DS1*02    : ------------------------------------------------------------------------------------------------ :     -
AOTVOKIR3DL2*01    : ------------------------------------------------------------------------------------------------ :     -
AOTVOKIR3DL2*01v1  : ------------------------------------------------------------------------------------------------ :     -
AOTVOKIR3DL2*02    : ------------------------------------------------------------------------------------------------ :     -
AOTVOKIR3DS3*01    : ------------------------------------------------------------------------------------------------ :     -
AOTVOKIR3DS3*02    : ------------------------------------------------------------------------------------------------ :     -
AOTVOKIR3DS3*03    : ------------------------------------------------------------------------------------------------ :     -
AOTVOKIR3DS3*03v1  : ------------------------------------------------------------------------------------------------ :     -

                         6440         *       6460         *       6480         *       6500         *       6520
BAC_clone_Om       : TCTCCATGTTGGTCAGGCTGGTCTTCAACCCCCGACCGCAGGTGATCCACCCGCCTCTGCCTCCCAAAGTGCTGGGACTACAGGCGTGAGCCACCG :  6522
AOTVOKIR3DL1*01    : ------------------------------------------------------------------------------------------------ :     -
AOTVOKIR2DL1*01v1  : ------------------------------------------------------------------------------------------------ :     -
AOTVOKIR3DS1*01v2  : ------------------------------------------------------------------------------------------------ :     -
AOTVOKIR3DS1*02    : ------------------------------------------------------------------------------------------------ :     -
```

```
AOTVOKIR3DL2*01   : ------------------------------------------------------------------------------------------------ :    -
AOTVOKIR3DL2*01v1 : ------------------------------------------------------------------------------------------------ :    -
AOTVOKIR3DL2*02   : ------------------------------------------------------------------------------------------------ :    -
AOTVOKIR3DS3*01   : ------------------------------------------------------------------------------------------------ :    -
AOTVOKIR3DS3*02   : ------------------------------------------------------------------------------------------------ :    -
AOTVOKIR3DS3*03   : ------------------------------------------------------------------------------------------------ :    -
AOTVOKIR3DS3*03v1 : ------------------------------------------------------------------------------------------------ :    -

                     *      6540         *      6560         *      6580         *      6600         *      6620
BAC_clone_Om      : CGCCCGGCAAACTTTTTGTTTTTTGAGGAACTTACATACTCTTCTCCTTTGTAATGTATGTACTAATTTACATTCGTATCGACAGTGTACCAGGGT :  6618
AOTVOKIR3DL1*01   : ------------------------------------------------------------------------------------------------ :    -
AOTVOKIR2DL1*01v1 : ------------------------------------------------------------------------------------------------ :    -
AOTVOKIR3DS1*01v2 : ------------------------------------------------------------------------------------------------ :    -
AOTVOKIR3DS1*02   : ------------------------------------------------------------------------------------------------ :    -
AOTVOKIR3DL2*01   : ------------------------------------------------------------------------------------------------ :    -
AOTVOKIR3DL2*01v1 : ------------------------------------------------------------------------------------------------ :    -
AOTVOKIR3DL2*02   : ------------------------------------------------------------------------------------------------ :    -
AOTVOKIR3DS3*01   : ------------------------------------------------------------------------------------------------ :    -
AOTVOKIR3DS3*02   : ------------------------------------------------------------------------------------------------ :    -
AOTVOKIR3DS3*03   : ------------------------------------------------------------------------------------------------ :    -
AOTVOKIR3DS3*03v1 : ------------------------------------------------------------------------------------------------ :    -

                         *      6640         *      6660         *      6680         *      6700         *      6720
BAC_clone_Om      : CCTCCCTTCTCTACCACCTCGCCAGCATTCGTTTTGTCTTTCAGATAAAAGCCAGTGTAATGCGGCGAGAAGATGGCTCACTGTGATTTCAGTTCG :  6714
AOTVOKIR3DL1*01   : ------------------------------------------------------------------------------------------------ :    -
AOTVOKIR2DL1*01v1 : ------------------------------------------------------------------------------------------------ :    -
AOTVOKIR3DS1*01v2 : ------------------------------------------------------------------------------------------------ :    -
AOTVOKIR3DS1*02   : ------------------------------------------------------------------------------------------------ :    -
AOTVOKIR3DL2*01   : ------------------------------------------------------------------------------------------------ :    -
AOTVOKIR3DL2*01v1 : ------------------------------------------------------------------------------------------------ :    -
AOTVOKIR3DL2*02   : ------------------------------------------------------------------------------------------------ :    -
AOTVOKIR3DS3*01   : ------------------------------------------------------------------------------------------------ :    -
AOTVOKIR3DS3*02   : ------------------------------------------------------------------------------------------------ :    -
AOTVOKIR3DS3*03   : ------------------------------------------------------------------------------------------------ :    -
AOTVOKIR3DS3*03v1 : ------------------------------------------------------------------------------------------------ :    -

                             *      6740         *      6760         *      6780         *      6800         *
BAC_clone_Om      : CATTTCTCTGATGACTACCGATACTGAGCACTTTTTCATACACGTGTTGGCCATTTGAATGTTTTTTTCGCTAAGTGTCTGTTCAGGTCTTTCACT :  6810
AOTVOKIR3DL1*01   : ------------------------------------------------------------------------------------------------ :    -
AOTVOKIR2DL1*01v1 : ------------------------------------------------------------------------------------------------ :    -
AOTVOKIR3DS1*01v2 : ------------------------------------------------------------------------------------------------ :    -
AOTVOKIR3DS1*02   : ------------------------------------------------------------------------------------------------ :    -
AOTVOKIR3DL2*01   : ------------------------------------------------------------------------------------------------ :    -
```

```
AOTVOKIR3DL2*01v1 : ------------------------------------------------------------------------------------------------ :     -
AOTVOKIR3DL2*02   : ------------------------------------------------------------------------------------------------ :     -
AOTVOKIR3DS3*01   : ------------------------------------------------------------------------------------------------ :     -
AOTVOKIR3DS3*02   : ------------------------------------------------------------------------------------------------ :     -
AOTVOKIR3DS3*03   : ------------------------------------------------------------------------------------------------ :     -
AOTVOKIR3DS3*03v1 : ------------------------------------------------------------------------------------------------ :     -

                    6820         *       6840         *       6860         *       6880         *       6900         *
BAC_clone_Om      : AATTGTTAAAGTAACTGTTTCATTTCATTGAGCTTTATGTGTACTCTAGCTATTAATCCCCTCTCAGTCACACACTCTGCAAATATTTCCTCCCAT :  6906
AOTVOKIR3DL1*01   : ------------------------------------------------------------------------------------------------ :     -
AOTVOKIR2DL1*01v1 : ------------------------------------------------------------------------------------------------ :     -
AOTVOKIR3DS1*01v2 : ------------------------------------------------------------------------------------------------ :     -
AOTVOKIR3DS1*02   : ------------------------------------------------------------------------------------------------ :     -
AOTVOKIR3DL2*01   : ------------------------------------------------------------------------------------------------ :     -
AOTVOKIR3DL2*01v1 : ------------------------------------------------------------------------------------------------ :     -
AOTVOKIR3DL2*02   : ------------------------------------------------------------------------------------------------ :     -
AOTVOKIR3DS3*01   : ------------------------------------------------------------------------------------------------ :     -
AOTVOKIR3DS3*02   : ------------------------------------------------------------------------------------------------ :     -
AOTVOKIR3DS3*03   : ------------------------------------------------------------------------------------------------ :     -
AOTVOKIR3DS3*03v1 : ------------------------------------------------------------------------------------------------ :     -

                        6920         *       6940         *       6960         *       6980         *       7000
BAC_clone_Om      : CGTCTGGGTTGTCTCTTCACTTCTTTGGTTGCTTCATTTGAGGTTCAGAAGCTGCTTAGTTGGATGTAATCCCAATGGTCTATTTTTGTTTTGATT :  7002
AOTVOKIR3DL1*01   : ------------------------------------------------------------------------------------------------ :     -
AOTVOKIR2DL1*01v1 : ------------------------------------------------------------------------------------------------ :     -
AOTVOKIR3DS1*01v2 : ------------------------------------------------------------------------------------------------ :     -
AOTVOKIR3DS1*02   : ------------------------------------------------------------------------------------------------ :     -
AOTVOKIR3DL2*01   : ------------------------------------------------------------------------------------------------ :     -
AOTVOKIR3DL2*01v1 : ------------------------------------------------------------------------------------------------ :     -
AOTVOKIR3DL2*02   : ------------------------------------------------------------------------------------------------ :     -
AOTVOKIR3DS3*01   : ------------------------------------------------------------------------------------------------ :     -
AOTVOKIR3DS3*02   : ------------------------------------------------------------------------------------------------ :     -
AOTVOKIR3DS3*03   : ------------------------------------------------------------------------------------------------ :     -
AOTVOKIR3DS3*03v1 : ------------------------------------------------------------------------------------------------ :     -

                     *       7020         *       7040         *       7060         *       7080         *       7100
BAC_clone_Om      : TCTTGTGTTTTTGATGTTTTAAAAAAATATCTTTTCTGGCCGGGTGAGGTGGCTCACACCTGTAATCCCAGCACCGTGGGAGGCTGAAGCAGGCAG :  7098
AOTVOKIR3DL1*01   : ------------------------------------------------------------------------------------------------ :     -
AOTVOKIR2DL1*01v1 : ------------------------------------------------------------------------------------------------ :     -
AOTVOKIR3DS1*01v2 : ------------------------------------------------------------------------------------------------ :     -
AOTVOKIR3DS1*02   : ------------------------------------------------------------------------------------------------ :     -
AOTVOKIR3DL2*01   : ------------------------------------------------------------------------------------------------ :     -
AOTVOKIR3DL2*01v1 : ------------------------------------------------------------------------------------------------ :     -
```

```
AOTVOKIR3DL2*02   : ------------------------------------------------------------------------------------------------ :     -
AOTVOKIR3DS3*01   : ------------------------------------------------------------------------------------------------ :     -
AOTVOKIR3DS3*02   : ------------------------------------------------------------------------------------------------ :     -
AOTVOKIR3DS3*03   : ------------------------------------------------------------------------------------------------ :     -
AOTVOKIR3DS3*03v1 : ------------------------------------------------------------------------------------------------ :     -

                          *      7120          *      7140          *      7160          *      7180          *      7200
BAC_clone_Om      : ATCACGAGGTCAAGAGATAGAGACCATCCTGGCAAACATGGTGAAGCCCCCTCTCTACTAAAAATGCAAAAAATTAGCTTGGCATGGTGGTGGGCG :  7194
AOTVOKIR3DL1*01   : ------------------------------------------------------------------------------------------------ :     -
AOTVOKIR2DL1*01v1 : ------------------------------------------------------------------------------------------------ :     -
AOTVOKIR3DS1*01v2 : ------------------------------------------------------------------------------------------------ :     -
AOTVOKIR3DS1*02   : ------------------------------------------------------------------------------------------------ :     -
AOTVOKIR3DL2*01   : ------------------------------------------------------------------------------------------------ :     -
AOTVOKIR3DL2*01v1 : ------------------------------------------------------------------------------------------------ :     -
AOTVOKIR3DL2*02   : ------------------------------------------------------------------------------------------------ :     -
AOTVOKIR3DS3*01   : ------------------------------------------------------------------------------------------------ :     -
AOTVOKIR3DS3*02   : ------------------------------------------------------------------------------------------------ :     -
AOTVOKIR3DS3*03   : ------------------------------------------------------------------------------------------------ :     -
AOTVOKIR3DS3*03v1 : ------------------------------------------------------------------------------------------------ :     -

                              *      7220          *      7240          *      7260          *      7280          *
BAC_clone_Om      : CCTGTAGTCCCGGCTACTCGGGAGGCTGACGCAGGAGGATCACTTGAACCCAGGAGGCGCAGGTTGCAGTGAGCTGGGATGGCACCACTGCATTCC :  7290
AOTVOKIR3DL1*01   : ------------------------------------------------------------------------------------------------ :     -
AOTVOKIR2DL1*01v1 : ------------------------------------------------------------------------------------------------ :     -
AOTVOKIR3DS1*01v2 : ------------------------------------------------------------------------------------------------ :     -
AOTVOKIR3DS1*02   : ------------------------------------------------------------------------------------------------ :     -
AOTVOKIR3DL2*01   : ------------------------------------------------------------------------------------------------ :     -
AOTVOKIR3DL2*01v1 : ------------------------------------------------------------------------------------------------ :     -
AOTVOKIR3DL2*02   : ------------------------------------------------------------------------------------------------ :     -
AOTVOKIR3DS3*01   : ------------------------------------------------------------------------------------------------ :     -
AOTVOKIR3DS3*02   : ------------------------------------------------------------------------------------------------ :     -
AOTVOKIR3DS3*03   : ------------------------------------------------------------------------------------------------ :     -
AOTVOKIR3DS3*03v1 : ------------------------------------------------------------------------------------------------ :     -

                    7300          *      7320          *      7340          *      7360          *      7380          *
BAC_clone_Om      : AGCCTGGCAACAGAGCAAGACTCTGTCTCAAAAAAAAATTGTCTTTCCTCAGATAAATGTCCTGGAGCATTTCCCCAGTGTTTCCTTTTAGGCATT :  7386
AOTVOKIR3DL1*01   : ------------------------------------------------------------------------------------------------ :     -
AOTVOKIR2DL1*01v1 : ------------------------------------------------------------------------------------------------ :     -
AOTVOKIR3DS1*01v2 : ------------------------------------------------------------------------------------------------ :     -
AOTVOKIR3DS1*02   : ------------------------------------------------------------------------------------------------ :     -
AOTVOKIR3DL2*01   : ------------------------------------------------------------------------------------------------ :     -
AOTVOKIR3DL2*01v1 : ------------------------------------------------------------------------------------------------ :     -
AOTVOKIR3DL2*02   : ------------------------------------------------------------------------------------------------ :     -
```

```
AOTVOKIR3DS3*01   : ------------------------------------------------------------------------------------------------ :    -
AOTVOKIR3DS3*02   : ------------------------------------------------------------------------------------------------ :    -
AOTVOKIR3DS3*03   : ------------------------------------------------------------------------------------------------ :    -
AOTVOKIR3DS3*03v1 : ------------------------------------------------------------------------------------------------ :    -

                        7400        *       7420        *       7440        *       7460        *       7480
BAC_clone_Om      : GTATGGGTTCAGGCTTCCGTCTTTCACCCGTTTTCATTTGGTTTTTGTCTATGCTGAGAGGCAGAGGTTTCAGTTTCATCTCTCTGCATGTAGATA :  7482
AOTVOKIR3DL1*01   : ------------------------------------------------------------------------------------------------ :    -
AOTVOKIR2DL1*01v1 : ------------------------------------------------------------------------------------------------ :    -
AOTVOKIR3DS1*01v2 : ------------------------------------------------------------------------------------------------ :    -
AOTVOKIR3DS1*02   : ------------------------------------------------------------------------------------------------ :    -
AOTVOKIR3DL2*01   : ------------------------------------------------------------------------------------------------ :    -
AOTVOKIR3DL2*01v1 : ------------------------------------------------------------------------------------------------ :    -
AOTVOKIR3DL2*02   : ------------------------------------------------------------------------------------------------ :    -
AOTVOKIR3DS3*01   : ------------------------------------------------------------------------------------------------ :    -
AOTVOKIR3DS3*02   : ------------------------------------------------------------------------------------------------ :    -
AOTVOKIR3DS3*03   : ------------------------------------------------------------------------------------------------ :    -
AOTVOKIR3DS3*03v1 : ------------------------------------------------------------------------------------------------ :    -

                     *       7500        *       7520        *       7540        *       7560        *       7580
BAC_clone_Om      : TTCAGCTTTCCCTGCTCCATTTATTGAAAATGTTGTCCTTTCCTGATTGTAGGTGCTTTGCCCTTACAATCATCAAAGTTCATTGGATGTAAATGC :  7578
AOTVOKIR3DL1*01   : ------------------------------------------------------------------------------------------------ :    -
AOTVOKIR2DL1*01v1 : ------------------------------------------------------------------------------------------------ :    -
AOTVOKIR3DS1*01v2 : ------------------------------------------------------------------------------------------------ :    -
AOTVOKIR3DS1*02   : ------------------------------------------------------------------------------------------------ :    -
AOTVOKIR3DL2*01   : ------------------------------------------------------------------------------------------------ :    -
AOTVOKIR3DL2*01v1 : ------------------------------------------------------------------------------------------------ :    -
AOTVOKIR3DL2*02   : ------------------------------------------------------------------------------------------------ :    -
AOTVOKIR3DS3*01   : ------------------------------------------------------------------------------------------------ :    -
AOTVOKIR3DS3*02   : ------------------------------------------------------------------------------------------------ :    -
AOTVOKIR3DS3*03   : ------------------------------------------------------------------------------------------------ :    -
AOTVOKIR3DS3*03v1 : ------------------------------------------------------------------------------------------------ :    -

                         *       7600        *       7620        *       7640        *       7660        *       7680
BAC_clone_Om      : AAGGATTACGTCTGTGTTCTTCCCTCTGCTCCATTGTTCTATCAGCCTTTCTTTATGCCAGTATCATGCTGTTTTGCTTACTACAGCTTTGTAACA :  7674
AOTVOKIR3DL1*01   : ------------------------------------------------------------------------------------------------ :    -
AOTVOKIR2DL1*01v1 : ------------------------------------------------------------------------------------------------ :    -
AOTVOKIR3DS1*01v2 : ------------------------------------------------------------------------------------------------ :    -
AOTVOKIR3DS1*02   : ------------------------------------------------------------------------------------------------ :    -
AOTVOKIR3DL2*01   : ------------------------------------------------------------------------------------------------ :    -
AOTVOKIR3DL2*01v1 : ------------------------------------------------------------------------------------------------ :    -
AOTVOKIR3DL2*02   : ------------------------------------------------------------------------------------------------ :    -
AOTVOKIR3DS3*01   : ------------------------------------------------------------------------------------------------ :    -
```

```
AOTVOKIR3DS3*02    : ------------------------------------------------------------------------------------------------ :     -
AOTVOKIR3DS3*03    : ------------------------------------------------------------------------------------------------ :     -
AOTVOKIR3DS3*03v1  : ------------------------------------------------------------------------------------------------ :     -

                              *      7700         *      7720         *      7740         *      7760         *
BAC_clone_Om       : TATTTTTAAGTGGGAGAGTGTCATGCATCCAGCACGTGTTTTGTATTTATACCTCAAAATCTTAGGACATGGGCATTGCTTTTGATGATTGTGGAG :  7770
AOTVOKIR3DL1*01    : ------------------------------------------------------------------------------------------------ :     -
AOTVOKIR2DL1*01v1  : ------------------------------------------------------------------------------------------------ :     -
AOTVOKIR3DS1*01v2  : ------------------------------------------------------------------------------------------------ :     -
AOTVOKIR3DS1*02    : ------------------------------------------------------------------------------------------------ :     -
AOTVOKIR3DL2*01    : ------------------------------------------------------------------------------------------------ :     -
AOTVOKIR3DL2*01v1  : ------------------------------------------------------------------------------------------------ :     -
AOTVOKIR3DL2*02    : ------------------------------------------------------------------------------------------------ :     -
AOTVOKIR3DS3*01    : ------------------------------------------------------------------------------------------------ :     -
AOTVOKIR3DS3*02    : ------------------------------------------------------------------------------------------------ :     -
AOTVOKIR3DS3*03    : ------------------------------------------------------------------------------------------------ :     -
AOTVOKIR3DS3*03v1  : ------------------------------------------------------------------------------------------------ :     -

                     7780         *      7800         *      7820         *      7840         *      7860         *
BAC_clone_Om       : AAGGGGGTGCCAGGGCCCATTAGATAACAGTATTGGCCATTAGTTAACCTCAAAGATTTCCAATGAGTGGAGGATAGGCACCCTCATTTCCACACC :  7866
AOTVOKIR3DL1*01    : ------------------------------------------------------------------------------------------------ :     -
AOTVOKIR2DL1*01v1  : ------------------------------------------------------------------------------------------------ :     -
AOTVOKIR3DS1*01v2  : ------------------------------------------------------------------------------------------------ :     -
AOTVOKIR3DS1*02    : ------------------------------------------------------------------------------------------------ :     -
AOTVOKIR3DL2*01    : ------------------------------------------------------------------------------------------------ :     -
AOTVOKIR3DL2*01v1  : ------------------------------------------------------------------------------------------------ :     -
AOTVOKIR3DL2*02    : ------------------------------------------------------------------------------------------------ :     -
AOTVOKIR3DS3*01    : ------------------------------------------------------------------------------------------------ :     -
AOTVOKIR3DS3*02    : ------------------------------------------------------------------------------------------------ :     -
AOTVOKIR3DS3*03    : ------------------------------------------------------------------------------------------------ :     -
AOTVOKIR3DS3*03v1  : ------------------------------------------------------------------------------------------------ :     -

                         7880         *      7900         *      7920         *      7940         *      7960
BAC_clone_Om       : TCTCTCCTGTCTGTTGTTCTGGAAATCCTTTAAGTAGCTGGCCTTCACCCACAGAACCAAGCTCCAAAAACGGTGAGAAAAGGATTCCTCTTATCT :  7962
AOTVOKIR3DL1*01    : ------------------------------------------------------------------------------------------------ :     -
AOTVOKIR2DL1*01v1  : ------------------------------------------------------------------------------------------------ :     -
AOTVOKIR3DS1*01v2  : ------------------------------------------------------------------------------------------------ :     -
AOTVOKIR3DS1*02    : ------------------------------------------------------------------------------------------------ :     -
AOTVOKIR3DL2*01    : ------------------------------------------------------------------------------------------------ :     -
AOTVOKIR3DL2*01v1  : ------------------------------------------------------------------------------------------------ :     -
AOTVOKIR3DL2*02    : ------------------------------------------------------------------------------------------------ :     -
AOTVOKIR3DS3*01    : ------------------------------------------------------------------------------------------------ :     -
AOTVOKIR3DS3*02    : ------------------------------------------------------------------------------------------------ :     -
```

```
AOTVOKIR3DS3*03    : ------------------------------------------------------------------------------------------------ :     -
AOTVOKIR3DS3*03v1  : ------------------------------------------------------------------------------------------------ :     -

                       *      7980          *       8000          *       8020          *       8040          *       8060
BAC_clone_Om       : CTGCTTTTGGAAACCTGGGGAGGTTGGTACCTTGGATTCAAGTGTTGGCTCAGTGCCTCCTAGCTCAGTGATTGTGGGGCGGTCTTCTAACATCTC :  8058
AOTVOKIR3DL1*01    : ------------------------------------------------------------------------------------------------ :     -
AOTVOKIR2DL1*01v1  : ------------------------------------------------------------------------------------------------ :     -
AOTVOKIR3DS1*01v2  : ------------------------------------------------------------------------------------------------ :     -
AOTVOKIR3DS1*02    : ------------------------------------------------------------------------------------------------ :     -
AOTVOKIR3DL2*01    : ------------------------------------------------------------------------------------------------ :     -
AOTVOKIR3DL2*01v1  : ------------------------------------------------------------------------------------------------ :     -
AOTVOKIR3DL2*02    : ------------------------------------------------------------------------------------------------ :     -
AOTVOKIR3DS3*01    : ------------------------------------------------------------------------------------------------ :     -
AOTVOKIR3DS3*02    : ------------------------------------------------------------------------------------------------ :     -
AOTVOKIR3DS3*03    : ------------------------------------------------------------------------------------------------ :     -
AOTVOKIR3DS3*03v1  : ------------------------------------------------------------------------------------------------ :     -

                           *      8080          *       8100          *       8120          *       8140          *       8160
BAC_clone_Om       : TGACTCTAAGACATTACAACAGTGAAACGTATCCGGGGTTAGCAAAGGGCTCACTGAAGTCTCTTCATTTCAAATTTCCACAGCTGAGACCTCCTT :  8154
AOTVOKIR3DL1*01    : ------------------------------------------------------------------------------------------------ :     -
AOTVOKIR2DL1*01v1  : ------------------------------------------------------------------------------------------------ :     -
AOTVOKIR3DS1*01v2  : ------------------------------------------------------------------------------------------------ :     -
AOTVOKIR3DS1*02    : ------------------------------------------------------------------------------------------------ :     -
AOTVOKIR3DL2*01    : ------------------------------------------------------------------------------------------------ :     -
AOTVOKIR3DL2*01v1  : ------------------------------------------------------------------------------------------------ :     -
AOTVOKIR3DL2*02    : ------------------------------------------------------------------------------------------------ :     -
AOTVOKIR3DS3*01    : ------------------------------------------------------------------------------------------------ :     -
AOTVOKIR3DS3*02    : ------------------------------------------------------------------------------------------------ :     -
AOTVOKIR3DS3*03    : ------------------------------------------------------------------------------------------------ :     -
AOTVOKIR3DS3*03v1  : ------------------------------------------------------------------------------------------------ :     -

                               *      8180          *       8200          *       8220          *       8240          *
BAC_clone_Om       : CAGGCTAAATGGATGAGTACAAATCTGACATCCTTCTCAGGGATAATGTGGTGGTTTTTATGCCTGCATTTCAAACTGGAGGACAAATTCATGGGG :  8250
AOTVOKIR3DL1*01    : ------------------------------------------------------------------------------------------------ :     -
AOTVOKIR2DL1*01v1  : ------------------------------------------------------------------------------------------------ :     -
AOTVOKIR3DS1*01v2  : ------------------------------------------------------------------------------------------------ :     -
AOTVOKIR3DS1*02    : ------------------------------------------------------------------------------------------------ :     -
AOTVOKIR3DL2*01    : ------------------------------------------------------------------------------------------------ :     -
AOTVOKIR3DL2*01v1  : ------------------------------------------------------------------------------------------------ :     -
AOTVOKIR3DL2*02    : ------------------------------------------------------------------------------------------------ :     -
AOTVOKIR3DS3*01    : ------------------------------------------------------------------------------------------------ :     -
AOTVOKIR3DS3*02    : ------------------------------------------------------------------------------------------------ :     -
AOTVOKIR3DS3*03    : ------------------------------------------------------------------------------------------------ :     -
```

```
AOTVOKIR3DS3*03v1 : ------------------------------------------------------------------------------------------------ :     -

                            8260         *        8280         *        8300         *        8320         *        8340         *
BAC_clone_Om      : GCTTGTGAGAGGAAAGGGAAGGGAACATCTGATGAGCGCAGGGTGTTTCTGCAGGATTCCACTTACCATGGAATAAGCTCCTGTTGCTCATGATGC :  8346
AOTVOKIR3DL1*01   : ------------------------------------------------------------------------------------------------ :     -
AOTVOKIR2DL1*01v1 : ------------------------------------------------------------------------------------------------ :     -
AOTVOKIR3DS1*01v2 : ------------------------------------------------------------------------------------------------ :     -
AOTVOKIR3DS1*02   : ------------------------------------------------------------------------------------------------ :     -
AOTVOKIR3DL2*01   : ------------------------------------------------------------------------------------------------ :     -
AOTVOKIR3DL2*01v1 : ------------------------------------------------------------------------------------------------ :     -
AOTVOKIR3DL2*02   : ------------------------------------------------------------------------------------------------ :     -
AOTVOKIR3DS3*01   : ------------------------------------------------------------------------------------------------ :     -
AOTVOKIR3DS3*02   : ------------------------------------------------------------------------------------------------ :     -
AOTVOKIR3DS3*03   : ------------------------------------------------------------------------------------------------ :     -
AOTVOKIR3DS3*03v1 : ------------------------------------------------------------------------------------------------ :     -

                        8360         *        8380         *        8400         *        8420         *        8440
BAC_clone_Om      : AACCCTGGCTGACTCAGCAGAGCAATAGTCTTGCAGTAAGAGAGAACGTAGGTCATCCACAGATATGAGGCTTCCACTCACTCGCTCGTTCAGCCC :  8442
AOTVOKIR3DL1*01   : ------------------------------------------------------------------------------------------------ :     -
AOTVOKIR2DL1*01v1 : ------------------------------------------------------------------------------------------------ :     -
AOTVOKIR3DS1*01v2 : ------------------------------------------------------------------------------------------------ :     -
AOTVOKIR3DS1*02   : ------------------------------------------------------------------------------------------------ :     -
AOTVOKIR3DL2*01   : ------------------------------------------------------------------------------------------------ :     -
AOTVOKIR3DL2*01v1 : ------------------------------------------------------------------------------------------------ :     -
AOTVOKIR3DL2*02   : ------------------------------------------------------------------------------------------------ :     -
AOTVOKIR3DS3*01   : ------------------------------------------------------------------------------------------------ :     -
AOTVOKIR3DS3*02   : ------------------------------------------------------------------------------------------------ :     -
AOTVOKIR3DS3*03   : ------------------------------------------------------------------------------------------------ :     -
AOTVOKIR3DS3*03v1 : ------------------------------------------------------------------------------------------------ :     -

                      *        8460         *        8480         *        8500         *        8520         *        8540
BAC_clone_Om      : CTGCGCCGCGCTCTTACTGGGCAGTGTGGAACCCGTTCCTGTTGTTGCTGTAATAAATTTCCACAAGCTTGATGGATGACAACAACACAGCTTTCA :  8538
AOTVOKIR3DL1*01   : ------------------------------------------------------------------------------------------------ :     -
AOTVOKIR2DL1*01v1 : ------------------------------------------------------------------------------------------------ :     -
AOTVOKIR3DS1*01v2 : ------------------------------------------------------------------------------------------------ :     -
AOTVOKIR3DS1*02   : ------------------------------------------------------------------------------------------------ :     -
AOTVOKIR3DL2*01   : ------------------------------------------------------------------------------------------------ :     -
AOTVOKIR3DL2*01v1 : ------------------------------------------------------------------------------------------------ :     -
AOTVOKIR3DL2*02   : ------------------------------------------------------------------------------------------------ :     -
AOTVOKIR3DS3*01   : ------------------------------------------------------------------------------------------------ :     -
AOTVOKIR3DS3*02   : ------------------------------------------------------------------------------------------------ :     -
AOTVOKIR3DS3*03   : ------------------------------------------------------------------------------------------------ :     -
AOTVOKIR3DS3*03v1 : ------------------------------------------------------------------------------------------------ :     -
```

```
                          *      8560          *      8580          *      8600          *      8620          *      8640
BAC_clone_Om       : AAACTATCTTACAGTGTTATAGCTAGGAAATATGAAATGCATCTCACGGCGCTAAAATCAACGTGACAGCGAGGCTGCCTTCCCTCTGAAGGTTTG :  8634
AOTVOKIR3DL1*01    : ------------------------------------------------------------------------------------------------ :     -
AOTVOKIR2DL1*01v1  : ------------------------------------------------------------------------------------------------ :     -
AOTVOKIR3DS1*01v2  : ------------------------------------------------------------------------------------------------ :     -
AOTVOKIR3DS1*02    : ------------------------------------------------------------------------------------------------ :     -
AOTVOKIR3DL2*01    : ------------------------------------------------------------------------------------------------ :     -
AOTVOKIR3DL2*01v1  : ------------------------------------------------------------------------------------------------ :     -
AOTVOKIR3DL2*02    : ------------------------------------------------------------------------------------------------ :     -
AOTVOKIR3DS3*01    : ------------------------------------------------------------------------------------------------ :     -
AOTVOKIR3DS3*02    : ------------------------------------------------------------------------------------------------ :     -
AOTVOKIR3DS3*03    : ------------------------------------------------------------------------------------------------ :     -
AOTVOKIR3DS3*03v1  : ------------------------------------------------------------------------------------------------ :     -

                              *      8660          *      8680          *      8700          *      8720          *
BAC_clone_Om       : AGGCGAGAATCTGCCTCCCACATTTCCCACCTCCTAGAGGCTCCCACATTCCTCGGCTCCTGGTCCCCTTCCTCCCTCTTAAAAGCACCCAAAGAC :  8730
AOTVOKIR3DL1*01    : ------------------------------------------------------------------------------------------------ :     -
AOTVOKIR2DL1*01v1  : ------------------------------------------------------------------------------------------------ :     -
AOTVOKIR3DS1*01v2  : ------------------------------------------------------------------------------------------------ :     -
AOTVOKIR3DS1*02    : ------------------------------------------------------------------------------------------------ :     -
AOTVOKIR3DL2*01    : ------------------------------------------------------------------------------------------------ :     -
AOTVOKIR3DL2*01v1  : ------------------------------------------------------------------------------------------------ :     -
AOTVOKIR3DL2*02    : ------------------------------------------------------------------------------------------------ :     -
AOTVOKIR3DS3*01    : ------------------------------------------------------------------------------------------------ :     -
AOTVOKIR3DS3*02    : ------------------------------------------------------------------------------------------------ :     -
AOTVOKIR3DS3*03    : ------------------------------------------------------------------------------------------------ :     -
AOTVOKIR3DS3*03v1  : ------------------------------------------------------------------------------------------------ :     -

                     8740          *      8760          *      8780          *      8800          *      8820          *
BAC_clone_Om       : TGGTCACGTCTCTCACACGGCATCACTCAACCCTTCTTCCTTACCACACCTCTTTCTCTGAATGTTACTCGCCCTCTTCCTCATCTTTCAAGGACT :  8826
AOTVOKIR3DL1*01    : ------------------------------------------------------------------------------------------------ :     -
AOTVOKIR2DL1*01v1  : ------------------------------------------------------------------------------------------------ :     -
AOTVOKIR3DS1*01v2  : ------------------------------------------------------------------------------------------------ :     -
AOTVOKIR3DS1*02    : ------------------------------------------------------------------------------------------------ :     -
AOTVOKIR3DL2*01    : ------------------------------------------------------------------------------------------------ :     -
AOTVOKIR3DL2*01v1  : ------------------------------------------------------------------------------------------------ :     -
AOTVOKIR3DL2*02    : ------------------------------------------------------------------------------------------------ :     -
AOTVOKIR3DS3*01    : ------------------------------------------------------------------------------------------------ :     -
AOTVOKIR3DS3*02    : ------------------------------------------------------------------------------------------------ :     -
AOTVOKIR3DS3*03    : ------------------------------------------------------------------------------------------------ :     -
AOTVOKIR3DS3*03v1  : ------------------------------------------------------------------------------------------------ :     -
```

```
                         8840         *        8860         *        8880         *        8900         *        8920
BAC_clone_Om       : TTGGGATTCTATTGAGTATGCCGAGATAATCCATCATAATCTCCCTAAAATCGTCGAGGATAACCTCCTTTTAAATTCAGCTGATCAGCATCTTCA :  8922
AOTVOKIR3DL1*01    : ------------------------------------------------------------------------------------------------ :     -
AOTVOKIR2DL1*01v1  : ------------------------------------------------------------------------------------------------ :     -
AOTVOKIR3DS1*01v2  : ------------------------------------------------------------------------------------------------ :     -
AOTVOKIR3DS1*02    : ------------------------------------------------------------------------------------------------ :     -
AOTVOKIR3DL2*01    : ------------------------------------------------------------------------------------------------ :     -
AOTVOKIR3DL2*01v1  : ------------------------------------------------------------------------------------------------ :     -
AOTVOKIR3DL2*02    : ------------------------------------------------------------------------------------------------ :     -
AOTVOKIR3DS3*01    : ------------------------------------------------------------------------------------------------ :     -
AOTVOKIR3DS3*02    : ------------------------------------------------------------------------------------------------ :     -
AOTVOKIR3DS3*03    : ------------------------------------------------------------------------------------------------ :     -
AOTVOKIR3DS3*03v1  : ------------------------------------------------------------------------------------------------ :     -

                      *        8940         *        8960         *        8980         *        9000         *        9020
BAC_clone_Om       : TTGCTCCTTTTCATGTAAAATAACATGTTCACAAGATATGGGGGCTAGGACACGGACATTTTCAGGGTAGGGCGGCATTTCTCTGCCTTCCACAAA :  9018
AOTVOKIR3DL1*01    : ------------------------------------------------------------------------------------------------ :     -
AOTVOKIR2DL1*01v1  : ------------------------------------------------------------------------------------------------ :     -
AOTVOKIR3DS1*01v2  : ------------------------------------------------------------------------------------------------ :     -
AOTVOKIR3DS1*02    : ------------------------------------------------------------------------------------------------ :     -
AOTVOKIR3DL2*01    : ------------------------------------------------------------------------------------------------ :     -
AOTVOKIR3DL2*01v1  : ------------------------------------------------------------------------------------------------ :     -
AOTVOKIR3DL2*02    : ------------------------------------------------------------------------------------------------ :     -
AOTVOKIR3DS3*01    : ------------------------------------------------------------------------------------------------ :     -
AOTVOKIR3DS3*02    : ------------------------------------------------------------------------------------------------ :     -
AOTVOKIR3DS3*03    : ------------------------------------------------------------------------------------------------ :     -
AOTVOKIR3DS3*03v1  : ------------------------------------------------------------------------------------------------ :     -

                          *        9040         *        9060         *        9080         *        9100         *        9120
BAC_clone_Om       : TGGTAAACAGGATGCATTTGTCCTTTGCTCTTAGGACACTGATATTGCAGGTGGTTAAATGGGAGGGCAGAACATGAACGCACAGGTGGGGCAATA :  9114
AOTVOKIR3DL1*01    : ------------------------------------------------------------------------------------------------ :     -
AOTVOKIR2DL1*01v1  : ------------------------------------------------------------------------------------------------ :     -
AOTVOKIR3DS1*01v2  : ------------------------------------------------------------------------------------------------ :     -
AOTVOKIR3DS1*02    : ------------------------------------------------------------------------------------------------ :     -
AOTVOKIR3DL2*01    : ------------------------------------------------------------------------------------------------ :     -
AOTVOKIR3DL2*01v1  : ------------------------------------------------------------------------------------------------ :     -
AOTVOKIR3DL2*02    : ------------------------------------------------------------------------------------------------ :     -
AOTVOKIR3DS3*01    : ------------------------------------------------------------------------------------------------ :     -
AOTVOKIR3DS3*02    : ------------------------------------------------------------------------------------------------ :     -
AOTVOKIR3DS3*03    : ------------------------------------------------------------------------------------------------ :     -
AOTVOKIR3DS3*03v1  : ------------------------------------------------------------------------------------------------ :     -
```

```
                           *        9140          *        9160          *        9180          *        9200          *
BAC_clone_Om        : ATCAATGATCCATTGTGAAGCAACTGTGCACAAAATGTATTAATTTGTCTATTTATTTACTTATTTGAGGTGAAGTCATGCCCCGTTTCCAGGCTG :  9210
AOTVOKIR3DL1*01     : ------------------------------------------------------------------------------------------------ :     -
AOTVOKIR2DL1*01v1   : ------------------------------------------------------------------------------------------------ :     -
AOTVOKIR3DS1*01v2   : ------------------------------------------------------------------------------------------------ :     -
AOTVOKIR3DS1*02     : ------------------------------------------------------------------------------------------------ :     -
AOTVOKIR3DL2*01     : ------------------------------------------------------------------------------------------------ :     -
AOTVOKIR3DL2*01v1   : ------------------------------------------------------------------------------------------------ :     -
AOTVOKIR3DL2*02     : ------------------------------------------------------------------------------------------------ :     -
AOTVOKIR3DS3*01     : ------------------------------------------------------------------------------------------------ :     -
AOTVOKIR3DS3*02     : ------------------------------------------------------------------------------------------------ :     -
AOTVOKIR3DS3*03     : ------------------------------------------------------------------------------------------------ :     -
AOTVOKIR3DS3*03v1   : ------------------------------------------------------------------------------------------------ :     -

                      9220          *        9240          *        9260          *        9280          *        9300          *
BAC_clone_Om        : TAATTCAGTGACATGATCTCGGCTCACTGCAACCTCCACTTTCCAGGTTCAAGAGATTCTCCTGACTCAGCCTCCCAAGTAACTGGGTTTACAATC :  9306
AOTVOKIR3DL1*01     : ------------------------------------------------------------------------------------------------ :     -
AOTVOKIR2DL1*01v1   : ------------------------------------------------------------------------------------------------ :     -
AOTVOKIR3DS1*01v2   : ------------------------------------------------------------------------------------------------ :     -
AOTVOKIR3DS1*02     : ------------------------------------------------------------------------------------------------ :     -
AOTVOKIR3DL2*01     : ------------------------------------------------------------------------------------------------ :     -
AOTVOKIR3DL2*01v1   : ------------------------------------------------------------------------------------------------ :     -
AOTVOKIR3DL2*02     : ------------------------------------------------------------------------------------------------ :     -
AOTVOKIR3DS3*01     : ------------------------------------------------------------------------------------------------ :     -
AOTVOKIR3DS3*02     : ------------------------------------------------------------------------------------------------ :     -
AOTVOKIR3DS3*03     : ------------------------------------------------------------------------------------------------ :     -
AOTVOKIR3DS3*03v1   : ------------------------------------------------------------------------------------------------ :     -

                          9320          *        9340          *        9360          *        9380          *        9400
BAC_clone_Om        : CCCTCCAGCACACTCAGCTAATTTTCTCTTATATTTTTTAGTAGAGATGGGGTTTCTCCGTGTTTTACAGGCTGTCTTGAACTCCCAACCTTAACT :  9402
AOTVOKIR3DL1*01     : ------------------------------------------------------------------------------------------------ :     -
AOTVOKIR2DL1*01v1   : ------------------------------------------------------------------------------------------------ :     -
AOTVOKIR3DS1*01v2   : ------------------------------------------------------------------------------------------------ :     -
AOTVOKIR3DS1*02     : ------------------------------------------------------------------------------------------------ :     -
AOTVOKIR3DL2*01     : ------------------------------------------------------------------------------------------------ :     -
AOTVOKIR3DL2*01v1   : ------------------------------------------------------------------------------------------------ :     -
AOTVOKIR3DL2*02     : ------------------------------------------------------------------------------------------------ :     -
AOTVOKIR3DS3*01     : ------------------------------------------------------------------------------------------------ :     -
AOTVOKIR3DS3*02     : ------------------------------------------------------------------------------------------------ :     -
AOTVOKIR3DS3*03     : ------------------------------------------------------------------------------------------------ :     -
AOTVOKIR3DS3*03v1   : ------------------------------------------------------------------------------------------------ :     -

                        *        9420          *        9440          *        9460          *        9480          *        9500
```

```
BAC_clone_Om      : GATCTCACTGCCTCAGCCTCCCAAACGGCTGGGACTCCAGGCGTGAGCCACAGCACCCAGCTGAAATTTAAAATAAATAATAGATAATGCTAAGCG :  9498
AOTVOKIR3DL1*01   : ------------------------------------------------------------------------------------------------ :     -
AOTVOKIR2DL1*01v1 : ------------------------------------------------------------------------------------------------ :     -
AOTVOKIR3DS1*01v2 : ------------------------------------------------------------------------------------------------ :     -
AOTVOKIR3DS1*02   : ------------------------------------------------------------------------------------------------ :     -
AOTVOKIR3DL2*01   : ------------------------------------------------------------------------------------------------ :     -
AOTVOKIR3DL2*01v1 : ------------------------------------------------------------------------------------------------ :     -
AOTVOKIR3DL2*02   : ------------------------------------------------------------------------------------------------ :     -
AOTVOKIR3DS3*01   : ------------------------------------------------------------------------------------------------ :     -
AOTVOKIR3DS3*02   : ------------------------------------------------------------------------------------------------ :     -
AOTVOKIR3DS3*03   : ------------------------------------------------------------------------------------------------ :     -
AOTVOKIR3DS3*03v1 : ------------------------------------------------------------------------------------------------ :     -

                         *      9520         *      9540         *      9560         *      9580         *      9600
BAC_clone_Om      : TAAAATGTCAGAAGACAGAGTAGGTTCATTGATCGACAATATTTGTGACCTTAATGAAAAAAATGATTTCAACTCTTGGAAGATAGGTGAAAGGAT :  9594
AOTVOKIR3DL1*01   : ------------------------------------------------------------------------------------------------ :     -
AOTVOKIR2DL1*01v1 : ------------------------------------------------------------------------------------------------ :     -
AOTVOKIR3DS1*01v2 : ------------------------------------------------------------------------------------------------ :     -
AOTVOKIR3DS1*02   : ------------------------------------------------------------------------------------------------ :     -
AOTVOKIR3DL2*01   : ------------------------------------------------------------------------------------------------ :     -
AOTVOKIR3DL2*01v1 : ------------------------------------------------------------------------------------------------ :     -
AOTVOKIR3DL2*02   : ------------------------------------------------------------------------------------------------ :     -
AOTVOKIR3DS3*01   : ------------------------------------------------------------------------------------------------ :     -
AOTVOKIR3DS3*02   : ------------------------------------------------------------------------------------------------ :     -
AOTVOKIR3DS3*03   : ------------------------------------------------------------------------------------------------ :     -
AOTVOKIR3DS3*03v1 : ------------------------------------------------------------------------------------------------ :     -

                             *      9620         *      9640         *      9660         *      9680         *
BAC_clone_Om      : TTTTCATGCAGAGTTGTCAGCTGTGAAGACACAAAGGTGCAAACAATCTTGTGTTGAAGGAAGAGGCTGTGCCTGACATGCTGGGAATGAGATGGG :  9690
AOTVOKIR3DL1*01   : ------------------------------------------------------------------------------------------------ :     -
AOTVOKIR2DL1*01v1 : ------------------------------------------------------------------------------------------------ :     -
AOTVOKIR3DS1*01v2 : ------------------------------------------------------------------------------------------------ :     -
AOTVOKIR3DS1*02   : ------------------------------------------------------------------------------------------------ :     -
AOTVOKIR3DL2*01   : ------------------------------------------------------------------------------------------------ :     -
AOTVOKIR3DL2*01v1 : ------------------------------------------------------------------------------------------------ :     -
AOTVOKIR3DL2*02   : ------------------------------------------------------------------------------------------------ :     -
AOTVOKIR3DS3*01   : ------------------------------------------------------------------------------------------------ :     -
AOTVOKIR3DS3*02   : ------------------------------------------------------------------------------------------------ :     -
AOTVOKIR3DS3*03   : ------------------------------------------------------------------------------------------------ :     -
AOTVOKIR3DS3*03v1 : ------------------------------------------------------------------------------------------------ :     -

                    9700         *      9720         *      9740         *      9760         *      9780         *
BAC_clone_Om      : GAGAATTACAGGATGACTGTAGAGACGGGGAGCACGCTGGGTACACAGGAAAATAAGAGGGAAAAGGAGTGTGTGTTTTCTACTCACAGCCATTGG :  9786
```

```
AOTVOKIR3DL1*01    : ------------------------------------------------------------------------------------------------ :      -
AOTVOKIR2DL1*01v1  : ------------------------------------------------------------------------------------------------ :      -
AOTVOKIR3DS1*01v2  : ------------------------------------------------------------------------------------------------ :      -
AOTVOKIR3DS1*02    : ------------------------------------------------------------------------------------------------ :      -
AOTVOKIR3DL2*01    : ------------------------------------------------------------------------------------------------ :      -
AOTVOKIR3DL2*01v1  : ------------------------------------------------------------------------------------------------ :      -
AOTVOKIR3DL2*02    : ------------------------------------------------------------------------------------------------ :      -
AOTVOKIR3DS3*01    : ------------------------------------------------------------------------------------------------ :      -
AOTVOKIR3DS3*02    : ------------------------------------------------------------------------------------------------ :      -
AOTVOKIR3DS3*03    : ------------------------------------------------------------------------------------------------ :      -
AOTVOKIR3DS3*03v1  : ------------------------------------------------------------------------------------------------ :      -

                         9800         *       9820         *       9840         *       9860         *       9880
BAC_clone_Om       : AATTCACCAGGGGCGAACTAGGAATCCCTACATGATTAACAGTGACACTCATTCCAGGGAGACAGAACACACACACAATGAATTACAGAAAGCAGG :   9882
AOTVOKIR3DL1*01    : ------------------------------------------------------------------------------------------------ :      -
AOTVOKIR2DL1*01v1  : ------------------------------------------------------------------------------------------------ :      -
AOTVOKIR3DS1*01v2  : ------------------------------------------------------------------------------------------------ :      -
AOTVOKIR3DS1*02    : ------------------------------------------------------------------------------------------------ :      -
AOTVOKIR3DL2*01    : ------------------------------------------------------------------------------------------------ :      -
AOTVOKIR3DL2*01v1  : ------------------------------------------------------------------------------------------------ :      -
AOTVOKIR3DL2*02    : ------------------------------------------------------------------------------------------------ :      -
AOTVOKIR3DS3*01    : ------------------------------------------------------------------------------------------------ :      -
AOTVOKIR3DS3*02    : ------------------------------------------------------------------------------------------------ :      -
AOTVOKIR3DS3*03    : ------------------------------------------------------------------------------------------------ :      -
AOTVOKIR3DS3*03v1  : ------------------------------------------------------------------------------------------------ :      -

                       *       9900         *       9920         *       9940         *       9960         *       9980
BAC_clone_Om       : TTCATTACTAACAGACAAGCAGCAAGTGACAACAGGAGCTACGCTTCAGGGTGAGCCAGTCCCCTAAGGCTCAGAAAAGCTGCCCGGGACACACGG :   9978
AOTVOKIR3DL1*01    : ------------------------------------------------------------------------------------------------ :      -
AOTVOKIR2DL1*01v1  : ------------------------------------------------------------------------------------------------ :      -
AOTVOKIR3DS1*01v2  : ------------------------------------------------------------------------------------------------ :      -
AOTVOKIR3DS1*02    : ------------------------------------------------------------------------------------------------ :      -
AOTVOKIR3DL2*01    : ------------------------------------------------------------------------------------------------ :      -
AOTVOKIR3DL2*01v1  : ------------------------------------------------------------------------------------------------ :      -
AOTVOKIR3DL2*02    : ------------------------------------------------------------------------------------------------ :      -
AOTVOKIR3DS3*01    : ------------------------------------------------------------------------------------------------ :      -
AOTVOKIR3DS3*02    : ------------------------------------------------------------------------------------------------ :      -
AOTVOKIR3DS3*03    : ------------------------------------------------------------------------------------------------ :      -
AOTVOKIR3DS3*03v1  : ------------------------------------------------------------------------------------------------ :      -

                           *      10000         *      10020         *      10040         *      10060         *      10080
BAC_clone_Om       : AGTCACCCCATATGCAGTGTAGCTGTGGGAACCGAAAAGCAGCCCCACCTGGGCTTTGCACCCTGGAGCCACAGGAAGCGCTGAGCTAAAGCCCTG :  10074
AOTVOKIR3DL1*01    : ------------------------------------------------------------------------------------------------ :      -
```

```
AOTVOKIR2DL1*01v1 : ------------------------------------------------------------------------------------------------ :     -
AOTVOKIR3DS1*01v2 : ------------------------------------------------------------------------------------------------ :     -
AOTVOKIR3DS1*02   : ------------------------------------------------------------------------------------------------ :     -
AOTVOKIR3DL2*01   : ------------------------------------------------------------------------------------------------ :     -
AOTVOKIR3DL2*01v1 : ------------------------------------------------------------------------------------------------ :     -
AOTVOKIR3DL2*02   : ------------------------------------------------------------------------------------------------ :     -
AOTVOKIR3DS3*01   : ------------------------------------------------------------------------------------------------ :     -
AOTVOKIR3DS3*02   : ------------------------------------------------------------------------------------------------ :     -
AOTVOKIR3DS3*03   : ------------------------------------------------------------------------------------------------ :     -
AOTVOKIR3DS3*03v1 : ------------------------------------------------------------------------------------------------ :     -

                             *     10100         *     10120         *     10140         *     10160         *     1
BAC_clone_Om      : CCTCACACCCTCCTCTAGGAAGAGCAGAAGAGAGCCCAGGCTGTTCTGGGACGTTCCTCCTGATCTCAGGACGTTGCTGTCTTAGTCCGTTTTTGT : 10170
AOTVOKIR3DL1*01   : ------------------------------------------------------------------------------------------------ :     -
AOTVOKIR2DL1*01v1 : ------------------------------------------------------------------------------------------------ :     -
AOTVOKIR3DS1*01v2 : ------------------------------------------------------------------------------------------------ :     -
AOTVOKIR3DS1*02   : ------------------------------------------------------------------------------------------------ :     -
AOTVOKIR3DL2*01   : ------------------------------------------------------------------------------------------------ :     -
AOTVOKIR3DL2*01v1 : ------------------------------------------------------------------------------------------------ :     -
AOTVOKIR3DL2*02   : ------------------------------------------------------------------------------------------------ :     -
AOTVOKIR3DS3*01   : ------------------------------------------------------------------------------------------------ :     -
AOTVOKIR3DS3*02   : ------------------------------------------------------------------------------------------------ :     -
AOTVOKIR3DS3*03   : ------------------------------------------------------------------------------------------------ :     -
AOTVOKIR3DS3*03v1 : ------------------------------------------------------------------------------------------------ :     -

                    0180         *     10200         *     10220         *     10240         *     10260         *
BAC_clone_Om      : TGCTATCAGGGAACACCTGAGCCTGGGTAACTTCTAAAGAAAACAGATGTGTTTGGCTCACAGTTCTGCAGGCTGTACTAGAAGCATGGCACCAGC : 10266
AOTVOKIR3DL1*01   : ------------------------------------------------------------------------------------------------ :     -
AOTVOKIR2DL1*01v1 : ------------------------------------------------------------------------------------------------ :     -
AOTVOKIR3DS1*01v2 : ------------------------------------------------------------------------------------------------ :     -
AOTVOKIR3DS1*02   : ------------------------------------------------------------------------------------------------ :     -
AOTVOKIR3DL2*01   : ------------------------------------------------------------------------------------------------ :     -
AOTVOKIR3DL2*01v1 : ------------------------------------------------------------------------------------------------ :     -
AOTVOKIR3DL2*02   : ------------------------------------------------------------------------------------------------ :     -
AOTVOKIR3DS3*01   : ------------------------------------------------------------------------------------------------ :     -
AOTVOKIR3DS3*02   : ------------------------------------------------------------------------------------------------ :     -
AOTVOKIR3DS3*03   : ------------------------------------------------------------------------------------------------ :     -
AOTVOKIR3DS3*03v1 : ------------------------------------------------------------------------------------------------ :     -

                       10280         *     10300         *     10320         *     10340         *     10360
BAC_clone_Om      : ATCTATTTCCTGTGATGACCTGAGGCCGCTTTCACTCTGGCAGAAGGAAGGGGGCCCTTGTGTGCAGACACCACAGAGATCACAGGGCAAAAGAGC : 10362
AOTVOKIR3DL1*01   : ------------------------------------------------------------------------------------------------ :     -
AOTVOKIR2DL1*01v1 : ------------------------------------------------------------------------------------------------ :     -
```

```
AOTVOKIR3DS1*01v2 : ------------------------------------------------------------------------------------------------ :     -
AOTVOKIR3DS1*02   : ------------------------------------------------------------------------------------------------ :     -
AOTVOKIR3DL2*01   : ------------------------------------------------------------------------------------------------ :     -
AOTVOKIR3DL2*01v1 : ------------------------------------------------------------------------------------------------ :     -
AOTVOKIR3DL2*02   : ------------------------------------------------------------------------------------------------ :     -
AOTVOKIR3DS3*01   : ------------------------------------------------------------------------------------------------ :     -
AOTVOKIR3DS3*02   : ------------------------------------------------------------------------------------------------ :     -
AOTVOKIR3DS3*03   : ------------------------------------------------------------------------------------------------ :     -
AOTVOKIR3DS3*03v1 : ------------------------------------------------------------------------------------------------ :     -

                     *     10380         *     10400         *     10420         *     10440         *     10460
BAC_clone_Om      : GAGTGAGGTCAGGCCATGGAGCCTCCAAGCTCTTTTTAGCAACCAGCTCTCAGGGAACTAACAGAAAACTTGCTAACCCCGTCTCATGGGGGCAGC : 10458
AOTVOKIR3DL1*01   : ------------------------------------------------------------------------------------------------ :     -
AOTVOKIR2DL1*01v1 : ------------------------------------------------------------------------------------------------ :     -
AOTVOKIR3DS1*01v2 : ------------------------------------------------------------------------------------------------ :     -
AOTVOKIR3DS1*02   : ------------------------------------------------------------------------------------------------ :     -
AOTVOKIR3DL2*01   : ------------------------------------------------------------------------------------------------ :     -
AOTVOKIR3DL2*01v1 : ------------------------------------------------------------------------------------------------ :     -
AOTVOKIR3DL2*02   : ------------------------------------------------------------------------------------------------ :     -
AOTVOKIR3DS3*01   : ------------------------------------------------------------------------------------------------ :     -
AOTVOKIR3DS3*02   : ------------------------------------------------------------------------------------------------ :     -
AOTVOKIR3DS3*03   : ------------------------------------------------------------------------------------------------ :     -
AOTVOKIR3DS3*03v1 : ------------------------------------------------------------------------------------------------ :     -

                         *     10480         *     10500         *     10520         *     10540         *     10560
BAC_clone_Om      : ATTAATCTATTCATGATGGATCCACCCCCATGACCGAAACACATCTCAATAGGCGCAAACTCCCACACTGCAAGTTAAATATCTAGGTTTGGAGGA : 10554
AOTVOKIR3DL1*01   : ------------------------------------------------------------------------------------------------ :     -
AOTVOKIR2DL1*01v1 : ------------------------------------------------------------------------------------------------ :     -
AOTVOKIR3DS1*01v2 : ------------------------------------------------------------------------------------------------ :     -
AOTVOKIR3DS1*02   : ------------------------------------------------------------------------------------------------ :     -
AOTVOKIR3DL2*01   : ------------------------------------------------------------------------------------------------ :     -
AOTVOKIR3DL2*01v1 : ------------------------------------------------------------------------------------------------ :     -
AOTVOKIR3DL2*02   : ------------------------------------------------------------------------------------------------ :     -
AOTVOKIR3DS3*01   : ------------------------------------------------------------------------------------------------ :     -
AOTVOKIR3DS3*02   : ------------------------------------------------------------------------------------------------ :     -
AOTVOKIR3DS3*03   : ------------------------------------------------------------------------------------------------ :     -
AOTVOKIR3DS3*03v1 : ------------------------------------------------------------------------------------------------ :     -

                             *     10580         *     10600         *     10620         *     10640         *     1
BAC_clone_Om      : GCCAGACATTCAAACTGTAGCAGTGGTATCTCCAGCACATTCTCTGATTATTGAGAACTATAGCTGAGAAAGCAGGAGAAATCTGGGTCTCCTGCC : 10650
AOTVOKIR3DL1*01   : ------------------------------------------------------------------------------------------------ :     -
AOTVOKIR2DL1*01v1 : ------------------------------------------------------------------------------------------------ :     -
AOTVOKIR3DS1*01v2 : ------------------------------------------------------------------------------------------------ :     -
```

```
AOTVOKIR3DS1*02    : ------------------------------------------------------------------------------------------------ :      -
AOTVOKIR3DL2*01    : ------------------------------------------------------------------------------------------------ :      -
AOTVOKIR3DL2*01v1  : ------------------------------------------------------------------------------------------------ :      -
AOTVOKIR3DL2*02    : ------------------------------------------------------------------------------------------------ :      -
AOTVOKIR3DS3*01    : ------------------------------------------------------------------------------------------------ :      -
AOTVOKIR3DS3*02    : ------------------------------------------------------------------------------------------------ :      -
AOTVOKIR3DS3*03    : ------------------------------------------------------------------------------------------------ :      -
AOTVOKIR3DS3*03v1  : ------------------------------------------------------------------------------------------------ :      -

                     0660         *       10680         *       10700         *       10720         *       10740         *
BAC_clone_Om       : ACTGGGGTGCTGGTTCTAAAGAGACGCTGTGTGTGGTTACCTGGCAGTGAAGAGATGAGAGATGATCCCTGAAGAGGAACTGCTGTGGTCAGCTGC : 10746
AOTVOKIR3DL1*01    : ------------------------------------------------------------------------------------------------ :      -
AOTVOKIR2DL1*01v1  : ------------------------------------------------------------------------------------------------ :      -
AOTVOKIR3DS1*01v2  : ------------------------------------------------------------------------------------------------ :      -
AOTVOKIR3DS1*02    : ------------------------------------------------------------------------------------------------ :      -
AOTVOKIR3DL2*01    : ------------------------------------------------------------------------------------------------ :      -
AOTVOKIR3DL2*01v1  : ------------------------------------------------------------------------------------------------ :      -
AOTVOKIR3DL2*02    : ------------------------------------------------------------------------------------------------ :      -
AOTVOKIR3DS3*01    : ------------------------------------------------------------------------------------------------ :      -
AOTVOKIR3DS3*02    : ------------------------------------------------------------------------------------------------ :      -
AOTVOKIR3DS3*03    : ------------------------------------------------------------------------------------------------ :      -
AOTVOKIR3DS3*03v1  : ------------------------------------------------------------------------------------------------ :      -

                                                                                         EXON 7
                        10760         *       10780         *       10800         *       10820         *       10840
BAC_clone_Om       : TTACTGGGTTCCCATCTTCCTCCAGGTTTCCCCAGACACCTGCATGTTCTCATTCGGTGCTCAGTGGTCATCATCCCTTTCACCATCCTCCTCTTC : 10842
AOTVOKIR3DL1*01    : -------------------------GTAACCCCAGACACCTGCACGTTCTGATTGGGTCCTCAGTGGTCATCATCCCCTTCACCATCCTCCTCTTC :   1017
AOTVOKIR2DL1*01v1  : -------------------------GTAACCCCAGACACCTGCACGTTCTGATTGGGTCCTCAGTGGTCATCATCCCCTTCACCATCCTCCTCTTC :    723
AOTVOKIR3DS1*01v2  : -------------------------GTAACCCCAGACACCTGCACGTTCTGATTGGGTCCTCAGTGGTCATCATCCCCTTCACCATCCTCCTCTTC :   1017
AOTVOKIR3DS1*02    : -------------------------GTAACCCCAGACACCTGCACGTTCTGATTGGGTCCTCGGTGGTCATCATCCCCTTCACCATCCTCCTCTTC :   1017
AOTVOKIR3DL2*01    : -------------------------GTAACCCCAGACACCTGCACGTTCTGATTGGGTCCTCAGTGGTCATCATCCCCTTCACCATCCTCCTCTTC :   1017
AOTVOKIR3DL2*01v1  : -------------------------GTAACCCCAGACACCTGCACGTTCTGATTGGGTCCTCAGTGGTCATCATCCCCTTCACCATCCTCCTCTTC :    981
AOTVOKIR3DL2*02    : -------------------------GTAACCCCAGACACCTGCACGTTCTGATTGGGTCCTCAGTGGTCATCATCCCCTTCACCATCCTCCTCTTC :   1017
AOTVOKIR3DS3*01    : -------------------------GTTTCCCCAGACACCTGCACGTTCTAATTCGGTGCTCAGTGGTCATCATCCCTTTCACCATCCTCCTCTTC :    981
AOTVOKIR3DS3*02    : -------------------------GTTTCCCCAGACACCTGCACGTTCTAATTCGGTGCTCAGTGGTCATCATCCCTTTCACCATCCTCCTCTTC :    981
AOTVOKIR3DS3*03    : -------------------------GTTTCCCCAGACACCTGCACGTTCTAATTCGGTGCTCAGTGGTCATCATCCCTTTCACCATCCTCCTCTTC :    999
AOTVOKIR3DS3*03v1  : ------------------------------------------------------------------------------------------------ :      -
                                              gt  ccccagacacctgcacgttct att ggt ctcagtggtcatcatccc ttcaccatcctcctcttc

                      *       10860         *       10880         *       10900         *       10920         *       10940
BAC_clone_Om       : TTTCTCATTCGTCGCTGGTGGTCCAACAAAAGGAGTAAGTCTCATGAAGCAGAGGCCGGAGACCTCAGGGCCGTGTGCGGAAGCAGGATGGGAGCA : 10938
AOTVOKIR3DL1*01    : TTTCTCATTCGTCGCTGGTGGTCCAACAAAAAGA-------------------------------------------------------------- :   1051
AOTVOKIR2DL1*01v1  : TTTCTCATTCGTCGCTGGTGGTCCAACAAAAAGA-------------------------------------------------------------- :    757
AOTVOKIR3DS1*01v2  : TTTCTCATTCGTCGCTGGTGGTCCAACAAAAAGA-------------------------------------------------------------- :   1051
AOTVOKIR3DS1*02    : TTTCTCATTCGTCGCTGGTGGTCCAACAAAAAGA-------------------------------------------------------------- :   1051
```

```
AOTVOKIR3DL2*01   : TTTCTCATTCGTCGCTGGTGGTCCAACAAAAAGA-------------------------------------------------------------- :  1051
AOTVOKIR3DL2*01v1 : TTTCTCATTCGTCGCTGGTGGTCCAACAAAAAGA-------------------------------------------------------------- :  1015
AOTVOKIR3DL2*02   : TTTCTCATTCGTCGCTGGTGGTCCAACAAAAAGA-------------------------------------------------------------- :  1051
AOTVOKIR3DS3*01   : TTTCTCATTCATCGCTGGTGGTCCAACAAAAGGA-------------------------------------------------------------- :  1015
AOTVOKIR3DS3*02   : TTTCTCATTCATCGCTGGTGGTCCAACAAAAGGAGTAAGTCTCATGAAGCAGAGGCCGGAGACCTCAGGGCCGTGTGCAGAAGCGGGATGAGAGCA :  1077
AOTVOKIR3DS3*03   : TTTCTCATTCATCGCTGGTGGTCCAACAAAAGGA-------------------------------------------------------------- :  1033
AOTVOKIR3DS3*03v1 : ------------------------------------------------------------------------------------------------ :     -
                    tttctcattc tcgctggtggtccaacaaaa ga

                         *     10960         *     10980         *     11000         *     11020         *     11040
BAC_clone_Om      : CGCGGGTGTGTGTTCTTCATCAGCGGGATGGTCTCTGGCCCAAGGCAGGAGCTGCAGAGGCAGGGCTTTCTAGAGAGAGCACCAGACACCCTGCCC : 11034
AOTVOKIR3DL1*01   : ------------------------------------------------------------------------------------------------ :     -
AOTVOKIR2DL1*01v1 : ------------------------------------------------------------------------------------------------ :     -
AOTVOKIR3DS1*01v2 : ------------------------------------------------------------------------------------------------ :     -
AOTVOKIR3DS1*02   : ------------------------------------------------------------------------------------------------ :     -
AOTVOKIR3DL2*01   : ------------------------------------------------------------------------------------------------ :     -
AOTVOKIR3DL2*01v1 : ------------------------------------------------------------------------------------------------ :     -
AOTVOKIR3DL2*02   : ------------------------------------------------------------------------------------------------ :     -
AOTVOKIR3DS3*01   : ------------------------------------------------------------------------------------------------ :     -
AOTVOKIR3DS3*02   : CGCGGGTGTGTGTTCTTCATCGGCGGGATGGTCTCTGGCCCAAGGCAGGAGCTGCAGAGGCAGAGCTTTCTAGAGAGAGCACCAGACACCCTGTCC :  1173
AOTVOKIR3DS3*03   : ------------------------------------------------------------------------------------------------ :     -
AOTVOKIR3DS3*03v1 : ------------------------------------------------------------------------------------------------ :     -


                             *     11060         *     11080         *     11100         *     11120         *     1
BAC_clone_Om      : CTGCCTTCAGCTCAGAATCAATGGGATGGAAATTGAGAGCTCTTCATGGGAGGGGTCCTTGTACCCAGAGAGACAGAATGTGTGAGTCAGGCTGTT : 11130
AOTVOKIR3DL1*01   : ------------------------------------------------------------------------------------------------ :     -
AOTVOKIR2DL1*01v1 : ------------------------------------------------------------------------------------------------ :     -
AOTVOKIR3DS1*01v2 : ------------------------------------------------------------------------------------------------ :     -
AOTVOKIR3DS1*02   : ------------------------------------------------------------------------------------------------ :     -
AOTVOKIR3DL2*01   : ------------------------------------------------------------------------------------------------ :     -
AOTVOKIR3DL2*01v1 : ------------------------------------------------------------------------------------------------ :     -
AOTVOKIR3DL2*02   : ------------------------------------------------------------------------------------------------ :     -
AOTVOKIR3DS3*01   : ------------------------------------------------------------------------------------------------ :     -
AOTVOKIR3DS3*02   : CTGCCTTCAGCTCAGAAGCAATGGGATGGGAATTGA------------------------------------------------------------ :  1209
AOTVOKIR3DS3*03   : ------------------------------------------------------------------------------------------------ :     -
AOTVOKIR3DS3*03v1 : ------------------------------------------------------------------------------------------------ :     -


                    1140         *     11160         *     11180         *     11200         *     11220         *
BAC_clone_Om      : GACAGCTGAGGGGCCTCAGGCACCCATGGCCTCCCCCTGTGTGTTGGTGTCTGCACATGAAATGAGGACACAGAAGGGCCCTCCCAGGTGTTTTGA : 11226
AOTVOKIR3DL1*01   : ------------------------------------------------------------------------------------------------ :     -
AOTVOKIR2DL1*01v1 : ------------------------------------------------------------------------------------------------ :     -
AOTVOKIR3DS1*01v2 : ------------------------------------------------------------------------------------------------ :     -
AOTVOKIR3DS1*02   : ------------------------------------------------------------------------------------------------ :     -
AOTVOKIR3DL2*01   : ------------------------------------------------------------------------------------------------ :     -
```

```
AOTVOKIR3DL2*01v1 : ------------------------------------------------------------------------------------------------ :     -
AOTVOKIR3DL2*02   : ------------------------------------------------------------------------------------------------ :     -
AOTVOKIR3DS3*01   : ------------------------------------------------------------------------------------------------ :     -
AOTVOKIR3DS3*02   : ------------------------------------------------------------------------------------------------ :     -
AOTVOKIR3DS3*03   : ------------------------------------------------------------------------------------------------ :     -
AOTVOKIR3DS3*03v1 : ------------------------------------------------------------------------------------------------ :     -

                                                                   EXON 8
                          11240         *       11260         *       11280         *       11300         *       11320
BAC_clone_Om      : TGACTTCTGTCTCCTACAGATGCTGCTGTAATGAACCAAGAACCTGGGGTGGACAGAACAGTGAACAGGGAGGTAGGTCCTCCTCAGCCCAGCCTT : 11322
AOTVOKIR3DL1*01   : -------------------ATGCTTCTGTAATGAACCAAGAGCCTGGGGAGGACAGAACAGTGAACAGGGAG------------------------ :  1104
AOTVOKIR2DL1*01v1 : -------------------ATGCTTCTGTAATGAACCAAGAGCCTGGGGAGGACAGAACAGTGAACAGGGAG------------------------ :   810
AOTVOKIR3DS1*01v2 : ------------------------------------------------------------------------------------------------ :     -
AOTVOKIR3DS1*02   : ------------------------------------------------------------------------------------------------ :     -
AOTVOKIR3DL2*01   : -------------------ATGCTTCTGTAATGAACCAAGAGCCTGGGGAGGACAGAACAGTGAACAGGGAG------------------------ :  1104
AOTVOKIR3DL2*01v1 : -------------------ATGCTTCTGTAATGAACCAAGAGCCTGGGGAGGACAGAACAGTGAACAGGGAG------------------------ :  1068
AOTVOKIR3DL2*02   : -------------------ATGCTTCTGTAATGAACCAAGAGCCTGGGGAGGACAGAACAGTGAACAGGGAG------------------------ :  1104
AOTVOKIR3DS3*01   : -------------------ATGCTGCTGTAATGAACCAAGAACCTGGGGCAGACAGAACAGTGAACAGGGAG------------------------ :  1068
AOTVOKIR3DS3*02   : ------------------------------------------------------------------------------------------------ :     -
AOTVOKIR3DS3*03   : -------------------ATGCTGCTGTAATGAACCAAGAACCTGGGGCAGACAGAACAGTGAACAGGGAG------------------------ :  1086
AOTVOKIR3DS3*03v1 : -------------------ATGCTGCTGTAATGAACCAAGAACCTGGGGCAGACAGAACAGTGAACAGGGAG------------------------ :   981

                                                                                                           EXON 9
                      *       11340         *       11360         *       11380         *       11400         *       11420
BAC_clone_Om      : GGGGATGGAGTCTTATTCCCTAATAGTCACGTAGAATGTGAGCCCCTCCCTCACTCAACATTTCCCTCTCTCCAGGACTCTGAGGAACAAGACCCT : 11418
AOTVOKIR3DL1*01   : ---------------------------------------------------------------------------GACTCTGATGAACAAGACCCT :  1125
AOTVOKIR2DL1*01v1 : ---------------------------------------------------------------------------GACTCTGATGAACAAGACCCT :   831
AOTVOKIR3DS1*01v2 : ---------------------------------------------------------------------------GACTCTGA------------- :  1059
AOTVOKIR3DS1*02   : ---------------------------------------------------------------------------GACTCTGA------------- :  1059
AOTVOKIR3DL2*01   : ---------------------------------------------------------------------------GACTCTGATGAACAAGACCCT :  1125
AOTVOKIR3DL2*01v1 : ---------------------------------------------------------------------------GACTCTGATGAACAAGACCCT :  1089
AOTVOKIR3DL2*02   : ---------------------------------------------------------------------------GACTCTGATGAACAAGACCCT :  1125
AOTVOKIR3DS3*01   : ---------------------------------------------------------------------------GACTCCGAGGAACAAGACCCT :  1089
AOTVOKIR3DS3*02   : ------------------------------------------------------------------------------------------------ :     -
AOTVOKIR3DS3*03   : ---------------------------------------------------------------------------GACTCTGAGGAACAAGACCCT :  1107
AOTVOKIR3DS3*03v1 : ---------------------------------------------------------------------------GACTCTGAGGAACAAGACCCT :  1002
                                                                                           gactctga

                          *       11440         *       11460         *       11480         *       11500         *       11520
BAC_clone_Om      : CAGGAGGTGACATAGGCACAGATGGATCACTGCGTTTTCACACAGAGAAAAATCACTCAGCCTTCTCAGAGGCCCAAGAGAGCCCCAACAGAAACG : 11514
AOTVOKIR3DL1*01   : CAGGAGGTGAAATACACACAGTTGAATCATGGCGTTTTCACACAGAGAAAAATCACTCGCCCATCTCAGAGGCCCAGGAGACTCCCAACAGATACC :  1221
AOTVOKIR2DL1*01v1 : CAGGAGGTGAAATACACACAGTTGAATCATGGCGTTTTCACACAGAGAAAAATCACTCGCCCATCTCAGAGGCCCAGGAGACTCCCAACAGATACC :   927
AOTVOKIR3DS1*01v2 : ------------------------------------------------------------------------------------------------ :     -
AOTVOKIR3DS1*02   : ------------------------------------------------------------------------------------------------ :     -
AOTVOKIR3DL2*01   : CAGGAGGTGAAATACACACAGTTGAATCATGGCGTTTTCACACAGAGAAAAATCACTCGCCCATCTCAGAGGCCCAGGAGACTCCCAACAGATACC :  1221
AOTVOKIR3DL2*01v1 : CAGGAGGTGAAATACACACAGTTGAATCATGGCGTTTTCACACAGAGAAAAATCACTCGCCCATCTCAGAGGCCCAGGAGACTCCCAACAGATACC :  1185
```

```
AOTVOKIR3DL2*02   : CAGGAGGTGAAATACACACAGTTGAATCATGGCGTTTTCACACAGAGAAAAATCACTCGCCCATCTCAGAGGCCCAGGAGACTCCCAACAGATACC :  1221
AOTVOKIR3DS3*01   : CAGGAGGTGACA----------------------------------------------------------------------------------- :  1101
AOTVOKIR3DS3*02   : ------------------------------------------------------------------------------------------------ :     -
AOTVOKIR3DS3*03   : CAGGAGGTGACA----------------------------------------------------------------------------------- :  1119
AOTVOKIR3DS3*03v1 : CAGGAGGTGACA----------------------------------------------------------------------------------- :  1014

                             *     11540         *     11560         *     11580         *     11600
BAC_clone_Om      : AGTGTGTACATGGAACTTCTAAATGCTGAGCCTGGATTG----CTGTCTCCTGTCTGTGAACATCATGCACAGGCCTTGA : 11590
AOTVOKIR3DL1*01   : AGTGTGTACATGGAACTCCCAAATGCGGAGCCCGGATTGAAAGTTGTCTCCTGTCCATGA-------------------- :  1281
AOTVOKIR2DL1*01v1 : AGTGTGTACATGGAACTCCCAAATGCGGAGCCCGGATTGAAAGTTGTCTCCTGTCCATGA-------------------- :   987
AOTVOKIR3DS1*01v2 : -------------------------------------------------------------------------------- :     -
AOTVOKIR3DS1*02   : -------------------------------------------------------------------------------- :     -
AOTVOKIR3DL2*01   : AGTGTGTACATGGAACTCCCAAATGCGGAGCCCGGATTGAAAGTTGTCTCCTGTCCATGA-------------------- :  1281
AOTVOKIR3DL2*01v1 : AGTGTGTACATGGAACTCCCAAATGCGGAGCCCGGATTGAAAGTTGTCTCCTGTCCATGA-------------------- :  1245
AOTVOKIR3DL2*02   : AGTGTGTACATGGAACTCCCAAATGCGGAGCCCGGATTGAAAGTTGTCTCCTGTCCATGA-------------------- :  1281
AOTVOKIR3DS3*01   : -------------------------------------------------------------------------------- :     -
AOTVOKIR3DS3*02   : -------------------------------------------------------------------------------- :     -
AOTVOKIR3DS3*03   : -------------------------------------------------------------------------------- :     -
AOTVOKIR3DS3*03v1 : -------------------------------------------------------------------------------- :     -
```
