# Supplementary material for: Identification and Diversity of Killer Cell Ig-Like Receptors in Aotus vociferans, a New World Monkey
Source: PLoS One. 2013 Nov 6;8(11):e79731. doi: 10.1371/journal.pone.0079731 (PMC3819253; doi:10.1371/journal.pone.0079731)
Supplement: Figure S12 — Alternative splicing on lineage VIb. Alignment of a putative locus from an A. nancymaae - A. azarai hybrid owl monkey (obtained from a BAC clone) and the putative A. vociferans exons in loci belonging to lineage VIb. Arrows indicate the putative acceptor and donor sites. (PDF) [file pone.0079731.s012.pdf]

EXON 1

```
                              *        20         *        40         *        60         *        80         *
BAC_clone_Om       : ATGGCACTCATGGCTGTCAGCATGGTGTGTGTCGGTGAGTCCTGGAACGAATCCAGGGGGGGAACCATGGGGTGGAGATATGGGACTGGAATGGA :    95
AOTVOKIR3DL4*01    : ---TCACTCATGGTCATCAGCATGGCGTGTGTCG------------------------------------------------------------- :    31
AOTVOKIR3DL4*02    : ---TCACTCATGGTCATCAGCATGGCGTGTGTCG------------------------------------------------------------- :    31
AOTVOKIR3DL4*02v1  : ---TCACTCATGGTCATCAGCATGGCGTGTGTCG------------------------------------------------------------- :    31
AOTVOKIR3DL4*02v2  : ---TCACTCATGGTCATCAGCATGGCGTGTGTCG------------------------------------------------------------- :    31
AOTVOKIR3DL4*03    : ---TCACTCATGGTCATCAGCATGGCGTGTGTCG------------------------------------------------------------- :    31
AOTVOKIR3DS4*04    : ---TCACTCATGGTCATCAGCATGGCGTGTGTCG------------------------------------------------------------- :    31
AOTVOKIR3DS4*04v1  : ---TCACTCATGGTCATCAGCATGGCGTGTGTCG------------------------------------------------------------- :    31
AOTVOKIR3DS5*01    : ---TCACTCATGGTCATCAGCATGGTGTGTGTCG------------------------------------------------------------- :    31
AOTVOKIR2DS5*01v1  : ---TCACTCATGGTCATCAGCATGGTGTGTGTCG------------------------------------------------------------- :    31
AOTVOKIR2DS5*01v2  : ---TCACTCATGGTCATCAGCATGGTGTGTGTCG------------------------------------------------------------- :    31
AOTVOKIR3DS7*01    : ---TCACTCATGGTCATCAGCATGGCGTGTGTTG------------------------------------------------------------- :    31
AOTVOKIR3DS7*01v1  : ---TCACTCATGGTCATCAGCATGGCGTGTGTTG------------------------------------------------------------- :    31
AOTVOKIR3DL8*01    : ---TCACTCATGGTCATCAGCATGGCGTGTGTCG------------------------------------------------------------- :    31
AOTVOKIR3DL8*01v1  : ---TCACTCATGGTCATCAGCATGGCGTGTGTCG------------------------------------------------------------- :    31
AOTVOKIR3DS8*01v2  : ---TCACTCATGGTCATCAGCATGGCGTGTGTCG------------------------------------------------------------- :    31
AOTVOKIR3DL8*02    : ---TCACTCATGGTCATCAGCATGGCGTGTGTCG------------------------------------------------------------- :    31
AOTVOKIR4DL9*01    : ---TCACTCATGGTCATCAGCATGGCGTGTGTCG------------------------------------------------------------- :    31
AOTVOKIR4DS9*01v1  : ---TCACTCATGGTCATCAGCATGGCGTGTGTCG------------------------------------------------------------- :    31
AOTVOKIR3DL9*01v2  : ---TCACTCATGGTCATCAGCATGGCGTGTGTCG------------------------------------------------------------- :    31
AOTVOKIR4DL9*02    : ---TCACTCATGGTCATCAGCATGGCGTGTGTCG------------------------------------------------------------- :    31
AOTVOKIR4DL10*01   : ---TCACTCATGGTCATCAGCATGGCGTGTGTCG------------------------------------------------------------- :    31
AOTVOKIR3DL10*01v1 : ---TCACTCATGGTCATCAGCATGGCGTGTGTCG------------------------------------------------------------- :    31
AOTVOKIR4DS10*01v2 : ---TCACTCATGGTCATCAGCATGGCGTGTGTCG------------------------------------------------------------- :    31
AOTVOKIR3DS10*01v3 : ---TCACTCATGGTCATCAGCATGGCGTGTGTCG------------------------------------------------------------- :    31
AOTVOKIR3DS10*01v4 : ---TCACTCATGGTCATCAGCATGGCGTGTGTCG------------------------------------------------------------- :    31
                        tCACTCATGGtcaTCAGCATGGcGTGTGTcG

                         100         *       120         *       140         *       160         *       180         *
BAC_clone_Om       : GATATGGGCCTGGGGTGGAGATATGGGACTGGAATGGAGGTATGGGGCTGGGGTGGATATATGGGCCTGGGAGATATGGGCCTGGGGTGGAGATA :   190
AOTVOKIR3DL4*01    : ----------------------------------------------------------------------------------------------- :     -
AOTVOKIR3DL4*02    : ----------------------------------------------------------------------------------------------- :     -
AOTVOKIR3DL4*02v1  : ----------------------------------------------------------------------------------------------- :     -
AOTVOKIR3DL4*02v2  : ----------------------------------------------------------------------------------------------- :     -
AOTVOKIR3DL4*03    : ----------------------------------------------------------------------------------------------- :     -
AOTVOKIR3DS4*04    : ----------------------------------------------------------------------------------------------- :     -
AOTVOKIR3DS4*04v1  : ----------------------------------------------------------------------------------------------- :     -
AOTVOKIR3DS5*01    : ----------------------------------------------------------------------------------------------- :     -
AOTVOKIR2DS5*01v1  : ----------------------------------------------------------------------------------------------- :     -
AOTVOKIR2DS5*01v2  : ----------------------------------------------------------------------------------------------- :     -
AOTVOKIR3DS7*01    : ----------------------------------------------------------------------------------------------- :     -
AOTVOKIR3DS7*01v1  : ----------------------------------------------------------------------------------------------- :     -
AOTVOKIR3DL8*01    : ----------------------------------------------------------------------------------------------- :     -
AOTVOKIR3DL8*01v1  : ----------------------------------------------------------------------------------------------- :     -
```

```
AOTVOKIR3DS8*01v2  : ----------------------------------------------------------------------------------------------- :     -
AOTVOKIR3DL8*02    : ----------------------------------------------------------------------------------------------- :     -
AOTVOKIR4DL9*01    : ----------------------------------------------------------------------------------------------- :     -
AOTVOKIR4DS9*01v1  : ----------------------------------------------------------------------------------------------- :     -
AOTVOKIR3DL9*01v2  : ----------------------------------------------------------------------------------------------- :     -
AOTVOKIR4DL9*02    : ----------------------------------------------------------------------------------------------- :     -
AOTVOKIR4DL10*01   : ----------------------------------------------------------------------------------------------- :     -
AOTVOKIR3DL10*01v1 : ----------------------------------------------------------------------------------------------- :     -
AOTVOKIR4DS10*01v2 : ----------------------------------------------------------------------------------------------- :     -
AOTVOKIR3DS10*01v3 : ----------------------------------------------------------------------------------------------- :     -
AOTVOKIR3DS10*01v4 : ----------------------------------------------------------------------------------------------- :     -

                              200         *       220         *       240         *       260         *       280
BAC_clone_Om       : TGGGTCTGGAGTGGGGATATGGGCCTGGGGTGGAGATATGGGCCTAGGGTGGAGATATGGGCCTGGAGTGGAGATATGGGCCTGGGGTGGAGATA :   285
AOTVOKIR3DL4*01    : ----------------------------------------------------------------------------------------------- :     -
AOTVOKIR3DL4*02    : ----------------------------------------------------------------------------------------------- :     -
AOTVOKIR3DL4*02v1  : ----------------------------------------------------------------------------------------------- :     -
AOTVOKIR3DL4*02v2  : ----------------------------------------------------------------------------------------------- :     -
AOTVOKIR3DL4*03    : ----------------------------------------------------------------------------------------------- :     -
AOTVOKIR3DS4*04    : ----------------------------------------------------------------------------------------------- :     -
AOTVOKIR3DS4*04v1  : ----------------------------------------------------------------------------------------------- :     -
AOTVOKIR3DS5*01    : ----------------------------------------------------------------------------------------------- :     -
AOTVOKIR2DS5*01v1  : ----------------------------------------------------------------------------------------------- :     -
AOTVOKIR2DS5*01v2  : ----------------------------------------------------------------------------------------------- :     -
AOTVOKIR3DS7*01    : ----------------------------------------------------------------------------------------------- :     -
AOTVOKIR3DS7*01v1  : ----------------------------------------------------------------------------------------------- :     -
AOTVOKIR3DL8*01    : ----------------------------------------------------------------------------------------------- :     -
AOTVOKIR3DL8*01v1  : ----------------------------------------------------------------------------------------------- :     -
AOTVOKIR3DS8*01v2  : ----------------------------------------------------------------------------------------------- :     -
AOTVOKIR3DL8*02    : ----------------------------------------------------------------------------------------------- :     -
AOTVOKIR4DL9*01    : ----------------------------------------------------------------------------------------------- :     -
AOTVOKIR4DS9*01v1  : ----------------------------------------------------------------------------------------------- :     -
AOTVOKIR3DL9*01v2  : ----------------------------------------------------------------------------------------------- :     -
AOTVOKIR4DL9*02    : ----------------------------------------------------------------------------------------------- :     -
AOTVOKIR4DL10*01   : ----------------------------------------------------------------------------------------------- :     -
AOTVOKIR3DL10*01v1 : ----------------------------------------------------------------------------------------------- :     -
AOTVOKIR4DS10*01v2 : ----------------------------------------------------------------------------------------------- :     -
AOTVOKIR3DS10*01v3 : ----------------------------------------------------------------------------------------------- :     -
AOTVOKIR3DS10*01v4 : ----------------------------------------------------------------------------------------------- :     -

                         *       300         *       320         *       340         *       360         *       380
BAC_clone_Om       : CGGGCCTGGAGTGGAGATATGGGCCTGGGATGGAGAAATGGACCTGGGGTGGAGATATGGACCTGAGTTAGAGATATGGGCCTGAAGTGGAGATA :   380
AOTVOKIR3DL4*01    : ----------------------------------------------------------------------------------------------- :     -
AOTVOKIR3DL4*02    : ----------------------------------------------------------------------------------------------- :     -
```

```
AOTVOKIR3DL4*02v1  : ----------------------------------------------------------------------------------------------- :     -
AOTVOKIR3DL4*02v2  : ----------------------------------------------------------------------------------------------- :     -
AOTVOKIR3DL4*03    : ----------------------------------------------------------------------------------------------- :     -
AOTVOKIR3DS4*04    : ----------------------------------------------------------------------------------------------- :     -
AOTVOKIR3DS4*04v1  : ----------------------------------------------------------------------------------------------- :     -
AOTVOKIR3DS5*01    : ----------------------------------------------------------------------------------------------- :     -
AOTVOKIR2DS5*01v1  : ----------------------------------------------------------------------------------------------- :     -
AOTVOKIR2DS5*01v2  : ----------------------------------------------------------------------------------------------- :     -
AOTVOKIR3DS7*01    : ----------------------------------------------------------------------------------------------- :     -
AOTVOKIR3DS7*01v1  : ----------------------------------------------------------------------------------------------- :     -
AOTVOKIR3DL8*01    : ----------------------------------------------------------------------------------------------- :     -
AOTVOKIR3DL8*01v1  : ----------------------------------------------------------------------------------------------- :     -
AOTVOKIR3DS8*01v2  : ----------------------------------------------------------------------------------------------- :     -
AOTVOKIR3DL8*02    : ----------------------------------------------------------------------------------------------- :     -
AOTVOKIR4DL9*01    : ----------------------------------------------------------------------------------------------- :     -
AOTVOKIR4DS9*01v1  : ----------------------------------------------------------------------------------------------- :     -
AOTVOKIR3DL9*01v2  : ----------------------------------------------------------------------------------------------- :     -
AOTVOKIR4DL9*02    : ----------------------------------------------------------------------------------------------- :     -
AOTVOKIR4DL10*01   : ----------------------------------------------------------------------------------------------- :     -
AOTVOKIR3DL10*01v1 : ----------------------------------------------------------------------------------------------- :     -
AOTVOKIR4DS10*01v2 : ----------------------------------------------------------------------------------------------- :     -
AOTVOKIR3DS10*01v3 : ----------------------------------------------------------------------------------------------- :     -
AOTVOKIR3DS10*01v4 : ----------------------------------------------------------------------------------------------- :     -


                              *       400         *       420         *       440         *       460         *
BAC_clone_Om       : TGGGCCTGGGGTGAGGATATGGGCCTGGGAGATATGGGCCTGGAGTGGAGATATGGGCCTGCAGTGGGAATATGGGACTGGGGTGGGGATATGGG :   475
AOTVOKIR3DL4*01    : ----------------------------------------------------------------------------------------------- :     -
AOTVOKIR3DL4*02    : ----------------------------------------------------------------------------------------------- :     -
AOTVOKIR3DL4*02v1  : ----------------------------------------------------------------------------------------------- :     -
AOTVOKIR3DL4*02v2  : ----------------------------------------------------------------------------------------------- :     -
AOTVOKIR3DL4*03    : ----------------------------------------------------------------------------------------------- :     -
AOTVOKIR3DS4*04    : ----------------------------------------------------------------------------------------------- :     -
AOTVOKIR3DS4*04v1  : ----------------------------------------------------------------------------------------------- :     -
AOTVOKIR3DS5*01    : ----------------------------------------------------------------------------------------------- :     -
AOTVOKIR2DS5*01v1  : ----------------------------------------------------------------------------------------------- :     -
AOTVOKIR2DS5*01v2  : ----------------------------------------------------------------------------------------------- :     -
AOTVOKIR3DS7*01    : ----------------------------------------------------------------------------------------------- :     -
AOTVOKIR3DS7*01v1  : ----------------------------------------------------------------------------------------------- :     -
AOTVOKIR3DL8*01    : ----------------------------------------------------------------------------------------------- :     -
AOTVOKIR3DL8*01v1  : ----------------------------------------------------------------------------------------------- :     -
AOTVOKIR3DS8*01v2  : ----------------------------------------------------------------------------------------------- :     -
AOTVOKIR3DL8*02    : ----------------------------------------------------------------------------------------------- :     -
AOTVOKIR4DL9*01    : ----------------------------------------------------------------------------------------------- :     -
AOTVOKIR4DS9*01v1  : ----------------------------------------------------------------------------------------------- :     -
AOTVOKIR3DL9*01v2  : ----------------------------------------------------------------------------------------------- :     -
```

```
AOTVOKIR4DL9*02    : ----------------------------------------------------------------------------------------------- :      -
AOTVOKIR4DL10*01   : ----------------------------------------------------------------------------------------------- :      -
AOTVOKIR3DL10*01v1 : ----------------------------------------------------------------------------------------------- :      -
AOTVOKIR4DS10*01v2 : ----------------------------------------------------------------------------------------------- :      -
AOTVOKIR3DS10*01v3 : ----------------------------------------------------------------------------------------------- :      -
AOTVOKIR3DS10*01v4 : ----------------------------------------------------------------------------------------------- :      -

                       480         *       500         *       520         *       540         *       560         *
BAC_clone_Om       : CCTGCCGTGGAGATATGGGCCTGCGGTGGGGATATGGGGCTGGGGTGGAGATATGGGCTTGCAGTGGGGATATGGGCCTGGTGTGGAGATATGGG :    570
AOTVOKIR3DL4*01    : ----------------------------------------------------------------------------------------------- :      -
AOTVOKIR3DL4*02    : ----------------------------------------------------------------------------------------------- :      -
AOTVOKIR3DL4*02v1  : ----------------------------------------------------------------------------------------------- :      -
AOTVOKIR3DL4*02v2  : ----------------------------------------------------------------------------------------------- :      -
AOTVOKIR3DL4*03    : ----------------------------------------------------------------------------------------------- :      -
AOTVOKIR3DS4*04    : ----------------------------------------------------------------------------------------------- :      -
AOTVOKIR3DS4*04v1  : ----------------------------------------------------------------------------------------------- :      -
AOTVOKIR3DS5*01    : ----------------------------------------------------------------------------------------------- :      -
AOTVOKIR2DS5*01v1  : ----------------------------------------------------------------------------------------------- :      -
AOTVOKIR2DS5*01v2  : ----------------------------------------------------------------------------------------------- :      -
AOTVOKIR3DS7*01    : ----------------------------------------------------------------------------------------------- :      -
AOTVOKIR3DS7*01v1  : ----------------------------------------------------------------------------------------------- :      -
AOTVOKIR3DL8*01    : ----------------------------------------------------------------------------------------------- :      -
AOTVOKIR3DL8*01v1  : ----------------------------------------------------------------------------------------------- :      -
AOTVOKIR3DS8*01v2  : ----------------------------------------------------------------------------------------------- :      -
AOTVOKIR3DL8*02    : ----------------------------------------------------------------------------------------------- :      -
AOTVOKIR4DL9*01    : ----------------------------------------------------------------------------------------------- :      -
AOTVOKIR4DS9*01v1  : ----------------------------------------------------------------------------------------------- :      -
AOTVOKIR3DL9*01v2  : ----------------------------------------------------------------------------------------------- :      -
AOTVOKIR4DL9*02    : ----------------------------------------------------------------------------------------------- :      -
AOTVOKIR4DL10*01   : ----------------------------------------------------------------------------------------------- :      -
AOTVOKIR3DL10*01v1 : ----------------------------------------------------------------------------------------------- :      -
AOTVOKIR4DS10*01v2 : ----------------------------------------------------------------------------------------------- :      -
AOTVOKIR3DS10*01v3 : ----------------------------------------------------------------------------------------------- :      -
AOTVOKIR3DS10*01v4 : ----------------------------------------------------------------------------------------------- :      -

                            580         *       600         *       620         *       640         *       660
BAC_clone_Om       : CCTGGAAGATATGGGCCTACAGTGGGGATATCGGACTGCGGTGGGGATATGGGCCTGGGGTGGAGATATGGGCCTGTGGTGGGGATATGGGCCTG :    665
AOTVOKIR3DL4*01    : ----------------------------------------------------------------------------------------------- :      -
AOTVOKIR3DL4*02    : ----------------------------------------------------------------------------------------------- :      -
AOTVOKIR3DL4*02v1  : ----------------------------------------------------------------------------------------------- :      -
AOTVOKIR3DL4*02v2  : ----------------------------------------------------------------------------------------------- :      -
AOTVOKIR3DL4*03    : ----------------------------------------------------------------------------------------------- :      -
AOTVOKIR3DS4*04    : ----------------------------------------------------------------------------------------------- :      -
AOTVOKIR3DS4*04v1  : ----------------------------------------------------------------------------------------------- :      -
```

```
AOTVOKIR3DS5*01      : ----------------------------------------------------------------------------------------------- :      -
AOTVOKIR2DS5*01v1    : ----------------------------------------------------------------------------------------------- :      -
AOTVOKIR2DS5*01v2    : ----------------------------------------------------------------------------------------------- :      -
AOTVOKIR3DS7*01      : ----------------------------------------------------------------------------------------------- :      -
AOTVOKIR3DS7*01v1    : ----------------------------------------------------------------------------------------------- :      -
AOTVOKIR3DL8*01      : ----------------------------------------------------------------------------------------------- :      -
AOTVOKIR3DL8*01v1    : ----------------------------------------------------------------------------------------------- :      -
AOTVOKIR3DS8*01v2    : ----------------------------------------------------------------------------------------------- :      -
AOTVOKIR3DL8*02      : ----------------------------------------------------------------------------------------------- :      -
AOTVOKIR4DL9*01      : ----------------------------------------------------------------------------------------------- :      -
AOTVOKIR4DS9*01v1    : ----------------------------------------------------------------------------------------------- :      -
AOTVOKIR3DL9*01v2    : ----------------------------------------------------------------------------------------------- :      -
AOTVOKIR4DL9*02      : ----------------------------------------------------------------------------------------------- :      -
AOTVOKIR4DL10*01     : ----------------------------------------------------------------------------------------------- :      -
AOTVOKIR3DL10*01v1   : ----------------------------------------------------------------------------------------------- :      -
AOTVOKIR4DS10*01v2   : ----------------------------------------------------------------------------------------------- :      -
AOTVOKIR3DS10*01v3   : ----------------------------------------------------------------------------------------------- :      -
AOTVOKIR3DS10*01v4   : ----------------------------------------------------------------------------------------------- :      -

                           *       680         *       700         *       720         *       740         *       760
BAC_clone_Om         : GTGTGGAGATATGGGCCTGGAAGATATGGGCCTACAGTGGGGATATGGGACTGGGGTGGGGATATGGGACTGGGGTGGAGATATGGGCCTGTGGT :    760
AOTVOKIR3DL4*01      : ----------------------------------------------------------------------------------------------- :      -
AOTVOKIR3DL4*02      : ----------------------------------------------------------------------------------------------- :      -
AOTVOKIR3DL4*02v1    : ----------------------------------------------------------------------------------------------- :      -
AOTVOKIR3DL4*02v2    : ----------------------------------------------------------------------------------------------- :      -
AOTVOKIR3DL4*03      : ----------------------------------------------------------------------------------------------- :      -
AOTVOKIR3DS4*04      : ----------------------------------------------------------------------------------------------- :      -
AOTVOKIR3DS4*04v1    : ----------------------------------------------------------------------------------------------- :      -
AOTVOKIR3DS5*01      : ----------------------------------------------------------------------------------------------- :      -
AOTVOKIR2DS5*01v1    : ----------------------------------------------------------------------------------------------- :      -
AOTVOKIR2DS5*01v2    : ----------------------------------------------------------------------------------------------- :      -
AOTVOKIR3DS7*01      : ----------------------------------------------------------------------------------------------- :      -
AOTVOKIR3DS7*01v1    : ----------------------------------------------------------------------------------------------- :      -
AOTVOKIR3DL8*01      : ----------------------------------------------------------------------------------------------- :      -
AOTVOKIR3DL8*01v1    : ----------------------------------------------------------------------------------------------- :      -
AOTVOKIR3DS8*01v2    : ----------------------------------------------------------------------------------------------- :      -
AOTVOKIR3DL8*02      : ----------------------------------------------------------------------------------------------- :      -
AOTVOKIR4DL9*01      : ----------------------------------------------------------------------------------------------- :      -
AOTVOKIR4DS9*01v1    : ----------------------------------------------------------------------------------------------- :      -
AOTVOKIR3DL9*01v2    : ----------------------------------------------------------------------------------------------- :      -
AOTVOKIR4DL9*02      : ----------------------------------------------------------------------------------------------- :      -
AOTVOKIR4DL10*01     : ----------------------------------------------------------------------------------------------- :      -
AOTVOKIR3DL10*01v1   : ----------------------------------------------------------------------------------------------- :      -
AOTVOKIR4DS10*01v2   : ----------------------------------------------------------------------------------------------- :      -
AOTVOKIR3DS10*01v3   : ----------------------------------------------------------------------------------------------- :      -
```

```
AOTVOKIR3DS10*01v4 : ----------------------------------------------------------------------------------------------- :     -

                              *       780         *       800         *       820         *       840         *
BAC_clone_Om       : GGGGATATGGGCCTGGTGTGGAGATATGGGCCTGGAAGATATGGGCCTACAGTGGGGATATGGGACTGGGGTGGAGATATGGGACTGGAGTGGAG :   855
AOTVOKIR3DL4*01    : ----------------------------------------------------------------------------------------------- :     -
AOTVOKIR3DL4*02    : ----------------------------------------------------------------------------------------------- :     -
AOTVOKIR3DL4*02v1  : ----------------------------------------------------------------------------------------------- :     -
AOTVOKIR3DL4*02v2  : ----------------------------------------------------------------------------------------------- :     -
AOTVOKIR3DL4*03    : ----------------------------------------------------------------------------------------------- :     -
AOTVOKIR3DS4*04    : ----------------------------------------------------------------------------------------------- :     -
AOTVOKIR3DS4*04v1  : ----------------------------------------------------------------------------------------------- :     -
AOTVOKIR3DS5*01    : ----------------------------------------------------------------------------------------------- :     -
AOTVOKIR2DS5*01v1  : ----------------------------------------------------------------------------------------------- :     -
AOTVOKIR2DS5*01v2  : ----------------------------------------------------------------------------------------------- :     -
AOTVOKIR3DS7*01    : ----------------------------------------------------------------------------------------------- :     -
AOTVOKIR3DS7*01v1  : ----------------------------------------------------------------------------------------------- :     -
AOTVOKIR3DL8*01    : ----------------------------------------------------------------------------------------------- :     -
AOTVOKIR3DL8*01v1  : ----------------------------------------------------------------------------------------------- :     -
AOTVOKIR3DS8*01v2  : ----------------------------------------------------------------------------------------------- :     -
AOTVOKIR3DL8*02    : ----------------------------------------------------------------------------------------------- :     -
AOTVOKIR4DL9*01    : ----------------------------------------------------------------------------------------------- :     -
AOTVOKIR4DS9*01v1  : ----------------------------------------------------------------------------------------------- :     -
AOTVOKIR3DL9*01v2  : ----------------------------------------------------------------------------------------------- :     -
AOTVOKIR4DL9*02    : ----------------------------------------------------------------------------------------------- :     -
AOTVOKIR4DL10*01   : ----------------------------------------------------------------------------------------------- :     -
AOTVOKIR3DL10*01v1 : ----------------------------------------------------------------------------------------------- :     -
AOTVOKIR4DS10*01v2 : ----------------------------------------------------------------------------------------------- :     -
AOTVOKIR3DS10*01v3 : ----------------------------------------------------------------------------------------------- :     -
AOTVOKIR3DS10*01v4 : ----------------------------------------------------------------------------------------------- :     -

                        860         *       880         *       900         *       920         *       940         *
BAC_clone_Om       : ATATGGTCCTGGGGTGCAGATATGATCCTGGGGTGGAGATATGGTCCTGGGGTGGGGCTATGGGCCTGGGGTGGCAATATGGGCCTGTGGTGGAG :   950
AOTVOKIR3DL4*01    : ----------------------------------------------------------------------------------------------- :     -
AOTVOKIR3DL4*02    : ----------------------------------------------------------------------------------------------- :     -
AOTVOKIR3DL4*02v1  : ----------------------------------------------------------------------------------------------- :     -
AOTVOKIR3DL4*02v2  : ----------------------------------------------------------------------------------------------- :     -
AOTVOKIR3DL4*03    : ----------------------------------------------------------------------------------------------- :     -
AOTVOKIR3DS4*04    : ----------------------------------------------------------------------------------------------- :     -
AOTVOKIR3DS4*04v1  : ----------------------------------------------------------------------------------------------- :     -
AOTVOKIR3DS5*01    : ----------------------------------------------------------------------------------------------- :     -
AOTVOKIR2DS5*01v1  : ----------------------------------------------------------------------------------------------- :     -
AOTVOKIR2DS5*01v2  : ----------------------------------------------------------------------------------------------- :     -
AOTVOKIR3DS7*01    : ----------------------------------------------------------------------------------------------- :     -
AOTVOKIR3DS7*01v1  : ----------------------------------------------------------------------------------------------- :     -
```

```
AOTVOKIR3DL8*01     : ----------------------------------------------------------------------------------------------- :     -
AOTVOKIR3DL8*01v1   : ----------------------------------------------------------------------------------------------- :     -
AOTVOKIR3DS8*01v2   : ----------------------------------------------------------------------------------------------- :     -
AOTVOKIR3DL8*02     : ----------------------------------------------------------------------------------------------- :     -
AOTVOKIR4DL9*01     : ----------------------------------------------------------------------------------------------- :     -
AOTVOKIR4DS9*01v1   : ----------------------------------------------------------------------------------------------- :     -
AOTVOKIR3DL9*01v2   : ----------------------------------------------------------------------------------------------- :     -
AOTVOKIR4DL9*02     : ----------------------------------------------------------------------------------------------- :     -
AOTVOKIR4DL10*01    : ----------------------------------------------------------------------------------------------- :     -
AOTVOKIR3DL10*01v1  : ----------------------------------------------------------------------------------------------- :     -
AOTVOKIR4DS10*01v2  : ----------------------------------------------------------------------------------------------- :     -
AOTVOKIR3DS10*01v3  : ----------------------------------------------------------------------------------------------- :     -
AOTVOKIR3DS10*01v4  : ----------------------------------------------------------------------------------------------- :     -

                              960         *       980         *      1000         *      1020         *      1040
BAC_clone_Om        : ATATGGGACTGGGGTGGAGATATGGGCCTGTGGTGGGGATATGGGACTGGGGTGGGGTTATGGGCTTGGGGTGGAGATATGGGCCTGGGAGATAT :  1045
AOTVOKIR3DL4*01     : ----------------------------------------------------------------------------------------------- :     -
AOTVOKIR3DL4*02     : ----------------------------------------------------------------------------------------------- :     -
AOTVOKIR3DL4*02v1   : ----------------------------------------------------------------------------------------------- :     -
AOTVOKIR3DL4*02v2   : ----------------------------------------------------------------------------------------------- :     -
AOTVOKIR3DL4*03     : ----------------------------------------------------------------------------------------------- :     -
AOTVOKIR3DS4*04     : ----------------------------------------------------------------------------------------------- :     -
AOTVOKIR3DS4*04v1   : ----------------------------------------------------------------------------------------------- :     -
AOTVOKIR3DS5*01     : ----------------------------------------------------------------------------------------------- :     -
AOTVOKIR2DS5*01v1   : ----------------------------------------------------------------------------------------------- :     -
AOTVOKIR2DS5*01v2   : ----------------------------------------------------------------------------------------------- :     -
AOTVOKIR3DS7*01     : ----------------------------------------------------------------------------------------------- :     -
AOTVOKIR3DS7*01v1   : ----------------------------------------------------------------------------------------------- :     -
AOTVOKIR3DL8*01     : ----------------------------------------------------------------------------------------------- :     -
AOTVOKIR3DL8*01v1   : ----------------------------------------------------------------------------------------------- :     -
AOTVOKIR3DS8*01v2   : ----------------------------------------------------------------------------------------------- :     -
AOTVOKIR3DL8*02     : ----------------------------------------------------------------------------------------------- :     -
AOTVOKIR4DL9*01     : ----------------------------------------------------------------------------------------------- :     -
AOTVOKIR4DS9*01v1   : ----------------------------------------------------------------------------------------------- :     -
AOTVOKIR3DL9*01v2   : ----------------------------------------------------------------------------------------------- :     -
AOTVOKIR4DL9*02     : ----------------------------------------------------------------------------------------------- :     -
AOTVOKIR4DL10*01    : ----------------------------------------------------------------------------------------------- :     -
AOTVOKIR3DL10*01v1  : ----------------------------------------------------------------------------------------------- :     -
AOTVOKIR4DS10*01v2  : ----------------------------------------------------------------------------------------------- :     -
AOTVOKIR3DS10*01v3  : ----------------------------------------------------------------------------------------------- :     -
AOTVOKIR3DS10*01v4  : ----------------------------------------------------------------------------------------------- :     -

                          *      1060         *      1080         *      1100         *      1120         *      1140
BAC_clone_Om        : GGGCCTGAGGTGGAGATTTGGGCCTGGAATGGAGATATGGGCCTGAGGTGGAGATACTGGCCTGGGATATATGGTCCTAGGGTGGAGATATGGTC :  1140
```

```
AOTVOKIR3DL4*01    : ----------------------------------------------------------------------------------------------- :     -
AOTVOKIR3DL4*02    : ----------------------------------------------------------------------------------------------- :     -
AOTVOKIR3DL4*02v1  : ----------------------------------------------------------------------------------------------- :     -
AOTVOKIR3DL4*02v2  : ----------------------------------------------------------------------------------------------- :     -
AOTVOKIR3DL4*03    : ----------------------------------------------------------------------------------------------- :     -
AOTVOKIR3DS4*04    : ----------------------------------------------------------------------------------------------- :     -
AOTVOKIR3DS4*04v1  : ----------------------------------------------------------------------------------------------- :     -
AOTVOKIR3DS5*01    : ----------------------------------------------------------------------------------------------- :     -
AOTVOKIR2DS5*01v1  : ----------------------------------------------------------------------------------------------- :     -
AOTVOKIR2DS5*01v2  : ----------------------------------------------------------------------------------------------- :     -
AOTVOKIR3DS7*01    : ----------------------------------------------------------------------------------------------- :     -
AOTVOKIR3DS7*01v1  : ----------------------------------------------------------------------------------------------- :     -
AOTVOKIR3DL8*01    : ----------------------------------------------------------------------------------------------- :     -
AOTVOKIR3DL8*01v1  : ----------------------------------------------------------------------------------------------- :     -
AOTVOKIR3DS8*01v2  : ----------------------------------------------------------------------------------------------- :     -
AOTVOKIR3DL8*02    : ----------------------------------------------------------------------------------------------- :     -
AOTVOKIR4DL9*01    : ----------------------------------------------------------------------------------------------- :     -
AOTVOKIR4DS9*01v1  : ----------------------------------------------------------------------------------------------- :     -
AOTVOKIR3DL9*01v2  : ----------------------------------------------------------------------------------------------- :     -
AOTVOKIR4DL9*02    : ----------------------------------------------------------------------------------------------- :     -
AOTVOKIR4DL10*01   : ----------------------------------------------------------------------------------------------- :     -
AOTVOKIR3DL10*01v1 : ----------------------------------------------------------------------------------------------- :     -
AOTVOKIR4DS10*01v2 : ----------------------------------------------------------------------------------------------- :     -
AOTVOKIR3DS10*01v3 : ----------------------------------------------------------------------------------------------- :     -
AOTVOKIR3DS10*01v4 : ----------------------------------------------------------------------------------------------- :     -


                              *      1160         *      1180         *      1200         *      1220         *
BAC_clone_Om       : CTGGGGTGGAGATATGGGCCTGGGGTGGAGATATGGGCCTGTGGTGGGGATATGGGAATGGGGTGGGGATATGGGACTAGGGTTGGGATATGGGA :  1235
AOTVOKIR3DL4*01    : ----------------------------------------------------------------------------------------------- :     -
AOTVOKIR3DL4*02    : ----------------------------------------------------------------------------------------------- :     -
AOTVOKIR3DL4*02v1  : ----------------------------------------------------------------------------------------------- :     -
AOTVOKIR3DL4*02v2  : ----------------------------------------------------------------------------------------------- :     -
AOTVOKIR3DL4*03    : ----------------------------------------------------------------------------------------------- :     -
AOTVOKIR3DS4*04    : ----------------------------------------------------------------------------------------------- :     -
AOTVOKIR3DS4*04v1  : ----------------------------------------------------------------------------------------------- :     -
AOTVOKIR3DS5*01    : ----------------------------------------------------------------------------------------------- :     -
AOTVOKIR2DS5*01v1  : ----------------------------------------------------------------------------------------------- :     -
AOTVOKIR2DS5*01v2  : ----------------------------------------------------------------------------------------------- :     -
AOTVOKIR3DS7*01    : ----------------------------------------------------------------------------------------------- :     -
AOTVOKIR3DS7*01v1  : ----------------------------------------------------------------------------------------------- :     -
AOTVOKIR3DL8*01    : ----------------------------------------------------------------------------------------------- :     -
AOTVOKIR3DL8*01v1  : ----------------------------------------------------------------------------------------------- :     -
AOTVOKIR3DS8*01v2  : ----------------------------------------------------------------------------------------------- :     -
AOTVOKIR3DL8*02    : ----------------------------------------------------------------------------------------------- :     -
AOTVOKIR4DL9*01    : ----------------------------------------------------------------------------------------------- :     -
```

```
AOTVOKIR4DS9*01v1  : ----------------------------------------------------------------------------------------------- :     -
AOTVOKIR3DL9*01v2  : ----------------------------------------------------------------------------------------------- :     -
AOTVOKIR4DL9*02    : ----------------------------------------------------------------------------------------------- :     -
AOTVOKIR4DL10*01   : ----------------------------------------------------------------------------------------------- :     -
AOTVOKIR3DL10*01v1 : ----------------------------------------------------------------------------------------------- :     -
AOTVOKIR4DS10*01v2 : ----------------------------------------------------------------------------------------------- :     -
AOTVOKIR3DS10*01v3 : ----------------------------------------------------------------------------------------------- :     -
AOTVOKIR3DS10*01v4 : ----------------------------------------------------------------------------------------------- :     -

                         1240        *       1260        *       1280        *       1300        *       1320        *
BAC_clone_Om       : CTGGGGTCTGGTTATGGGCCTGGGGTAGAGATATGGGCCTGGGGTATAGATATGGGCCTGGGGTGGAAATATGGGCCTCTGGTGAGATATGGGCC :  1330
AOTVOKIR3DL4*01    : ----------------------------------------------------------------------------------------------- :     -
AOTVOKIR3DL4*02    : ----------------------------------------------------------------------------------------------- :     -
AOTVOKIR3DL4*02v1  : ----------------------------------------------------------------------------------------------- :     -
AOTVOKIR3DL4*02v2  : ----------------------------------------------------------------------------------------------- :     -
AOTVOKIR3DL4*03    : ----------------------------------------------------------------------------------------------- :     -
AOTVOKIR3DS4*04    : ----------------------------------------------------------------------------------------------- :     -
AOTVOKIR3DS4*04v1  : ----------------------------------------------------------------------------------------------- :     -
AOTVOKIR3DS5*01    : ----------------------------------------------------------------------------------------------- :     -
AOTVOKIR2DS5*01v1  : ----------------------------------------------------------------------------------------------- :     -
AOTVOKIR2DS5*01v2  : ----------------------------------------------------------------------------------------------- :     -
AOTVOKIR3DS7*01    : ----------------------------------------------------------------------------------------------- :     -
AOTVOKIR3DS7*01v1  : ----------------------------------------------------------------------------------------------- :     -
AOTVOKIR3DL8*01    : ----------------------------------------------------------------------------------------------- :     -
AOTVOKIR3DL8*01v1  : ----------------------------------------------------------------------------------------------- :     -
AOTVOKIR3DS8*01v2  : ----------------------------------------------------------------------------------------------- :     -
AOTVOKIR3DL8*02    : ----------------------------------------------------------------------------------------------- :     -
AOTVOKIR4DL9*01    : ----------------------------------------------------------------------------------------------- :     -
AOTVOKIR4DS9*01v1  : ----------------------------------------------------------------------------------------------- :     -
AOTVOKIR3DL9*01v2  : ----------------------------------------------------------------------------------------------- :     -
AOTVOKIR4DL9*02    : ----------------------------------------------------------------------------------------------- :     -
AOTVOKIR4DL10*01   : ----------------------------------------------------------------------------------------------- :     -
AOTVOKIR3DL10*01v1 : ----------------------------------------------------------------------------------------------- :     -
AOTVOKIR4DS10*01v2 : ----------------------------------------------------------------------------------------------- :     -
AOTVOKIR3DS10*01v3 : ----------------------------------------------------------------------------------------------- :     -
AOTVOKIR3DS10*01v4 : ----------------------------------------------------------------------------------------------- :     -

                              1340        *       1360        *       1380        *       1400        *       1420
BAC_clone_Om       : TGGGATGTAGATATGGGCCTGGGGTGGAGATATGGGCCTGTGGTGGGTATATGGGACTTGGGTGGGGATATGGGCCTGGGGTGGAGATATGGGCC :  1425
AOTVOKIR3DL4*01    : ----------------------------------------------------------------------------------------------- :     -
AOTVOKIR3DL4*02    : ----------------------------------------------------------------------------------------------- :     -
AOTVOKIR3DL4*02v1  : ----------------------------------------------------------------------------------------------- :     -
AOTVOKIR3DL4*02v2  : ----------------------------------------------------------------------------------------------- :     -
AOTVOKIR3DL4*03    : ----------------------------------------------------------------------------------------------- :     -
```

```
AOTVOKIR3DS4*04     : ----------------------------------------------------------------------------------------------- :    -
AOTVOKIR3DS4*04v1   : ----------------------------------------------------------------------------------------------- :    -
AOTVOKIR3DS5*01     : ----------------------------------------------------------------------------------------------- :    -
AOTVOKIR2DS5*01v1   : ----------------------------------------------------------------------------------------------- :    -
AOTVOKIR2DS5*01v2   : ----------------------------------------------------------------------------------------------- :    -
AOTVOKIR3DS7*01     : ----------------------------------------------------------------------------------------------- :    -
AOTVOKIR3DS7*01v1   : ----------------------------------------------------------------------------------------------- :    -
AOTVOKIR3DL8*01     : ----------------------------------------------------------------------------------------------- :    -
AOTVOKIR3DL8*01v1   : ----------------------------------------------------------------------------------------------- :    -
AOTVOKIR3DS8*01v2   : ----------------------------------------------------------------------------------------------- :    -
AOTVOKIR3DL8*02     : ----------------------------------------------------------------------------------------------- :    -
AOTVOKIR4DL9*01     : ----------------------------------------------------------------------------------------------- :    -
AOTVOKIR4DS9*01v1   : ----------------------------------------------------------------------------------------------- :    -
AOTVOKIR3DL9*01v2   : ----------------------------------------------------------------------------------------------- :    -
AOTVOKIR4DL9*02     : ----------------------------------------------------------------------------------------------- :    -
AOTVOKIR4DL10*01    : ----------------------------------------------------------------------------------------------- :    -
AOTVOKIR3DL10*01v1  : ----------------------------------------------------------------------------------------------- :    -
AOTVOKIR4DS10*01v2  : ----------------------------------------------------------------------------------------------- :    -
AOTVOKIR3DS10*01v3  : ----------------------------------------------------------------------------------------------- :    -
AOTVOKIR3DS10*01v4  : ----------------------------------------------------------------------------------------------- :    -

                          *       1440         *       1460         *       1480         *       1500         *       1520
BAC_clone_Om        : TGGGGTAGAGATATTGCCTCTAGTGGAGATAAGAGCCTCGAGGTATAGACCTGGGCCTGGAGGCTGGGACTCTGCACAGCCAAGAGCCCTGTTGT : 1520
AOTVOKIR3DL4*01     : ----------------------------------------------------------------------------------------------- :    -
AOTVOKIR3DL4*02     : ----------------------------------------------------------------------------------------------- :    -
AOTVOKIR3DL4*02v1   : ----------------------------------------------------------------------------------------------- :    -
AOTVOKIR3DL4*02v2   : ----------------------------------------------------------------------------------------------- :    -
AOTVOKIR3DL4*03     : ----------------------------------------------------------------------------------------------- :    -
AOTVOKIR3DS4*04     : ----------------------------------------------------------------------------------------------- :    -
AOTVOKIR3DS4*04v1   : ----------------------------------------------------------------------------------------------- :    -
AOTVOKIR3DS5*01     : ----------------------------------------------------------------------------------------------- :    -
AOTVOKIR2DS5*01v1   : ----------------------------------------------------------------------------------------------- :    -
AOTVOKIR2DS5*01v2   : ----------------------------------------------------------------------------------------------- :    -
AOTVOKIR3DS7*01     : ----------------------------------------------------------------------------------------------- :    -
AOTVOKIR3DS7*01v1   : ----------------------------------------------------------------------------------------------- :    -
AOTVOKIR3DL8*01     : ----------------------------------------------------------------------------------------------- :    -
AOTVOKIR3DL8*01v1   : ----------------------------------------------------------------------------------------------- :    -
AOTVOKIR3DS8*01v2   : ----------------------------------------------------------------------------------------------- :    -
AOTVOKIR3DL8*02     : ----------------------------------------------------------------------------------------------- :    -
AOTVOKIR4DL9*01     : ----------------------------------------------------------------------------------------------- :    -
AOTVOKIR4DS9*01v1   : ----------------------------------------------------------------------------------------------- :    -
AOTVOKIR3DL9*01v2   : ----------------------------------------------------------------------------------------------- :    -
AOTVOKIR4DL9*02     : ----------------------------------------------------------------------------------------------- :    -
AOTVOKIR4DL10*01    : ----------------------------------------------------------------------------------------------- :    -
AOTVOKIR3DL10*01v1  : ----------------------------------------------------------------------------------------------- :    -
```

```
AOTVOKIR4DS10*01v2 : ----------------------------------------------------------------------------------------------- :    -
AOTVOKIR3DS10*01v3 : ----------------------------------------------------------------------------------------------- :    -
AOTVOKIR3DS10*01v4 : ----------------------------------------------------------------------------------------------- :    -

                              *        1540                *        1560                *        1580                *        1600                *
BAC_clone_Om       : TGGGGTCAGGTGAGCAACGAGGCTGAGTTTACCTTCAACCTAAAAAGGGCTTGGCTCTCAAGGCACACAGCCCATTTGGGGCAGCAGGGCAGGGC : 1615
AOTVOKIR3DL4*01    : ----------------------------------------------------------------------------------------------- :    -
AOTVOKIR3DL4*02    : ----------------------------------------------------------------------------------------------- :    -
AOTVOKIR3DL4*02v1  : ----------------------------------------------------------------------------------------------- :    -
AOTVOKIR3DL4*02v2  : ----------------------------------------------------------------------------------------------- :    -
AOTVOKIR3DL4*03    : ----------------------------------------------------------------------------------------------- :    -
AOTVOKIR3DS4*04    : ----------------------------------------------------------------------------------------------- :    -
AOTVOKIR3DS4*04v1  : ----------------------------------------------------------------------------------------------- :    -
AOTVOKIR3DS5*01    : ----------------------------------------------------------------------------------------------- :    -
AOTVOKIR2DS5*01v1  : ----------------------------------------------------------------------------------------------- :    -
AOTVOKIR2DS5*01v2  : ----------------------------------------------------------------------------------------------- :    -
AOTVOKIR3DS7*01    : ----------------------------------------------------------------------------------------------- :    -
AOTVOKIR3DS7*01v1  : ----------------------------------------------------------------------------------------------- :    -
AOTVOKIR3DL8*01    : ----------------------------------------------------------------------------------------------- :    -
AOTVOKIR3DL8*01v1  : ----------------------------------------------------------------------------------------------- :    -
AOTVOKIR3DS8*01v2  : ----------------------------------------------------------------------------------------------- :    -
AOTVOKIR3DL8*02    : ----------------------------------------------------------------------------------------------- :    -
AOTVOKIR4DL9*01    : ----------------------------------------------------------------------------------------------- :    -
AOTVOKIR4DS9*01v1  : ----------------------------------------------------------------------------------------------- :    -
AOTVOKIR3DL9*01v2  : ----------------------------------------------------------------------------------------------- :    -
AOTVOKIR4DL9*02    : ----------------------------------------------------------------------------------------------- :    -
AOTVOKIR4DL10*01   : ----------------------------------------------------------------------------------------------- :    -
AOTVOKIR3DL10*01v1 : ----------------------------------------------------------------------------------------------- :    -
AOTVOKIR4DS10*01v2 : ----------------------------------------------------------------------------------------------- :    -
AOTVOKIR3DS10*01v3 : ----------------------------------------------------------------------------------------------- :    -
AOTVOKIR3DS10*01v4 : ----------------------------------------------------------------------------------------------- :    -

                                                                                          EXON 2
                         1620         *        1640         *        1660         *        1680         *        1700         *
BAC_clone_Om       : TGGTTTATGTGCAGCTGGATTGTCCATCATGATCTTTCTTTCCAGGGTTCTTCTTGGCCCAGAGGGCCTGGTCACGTGAGGGTGAGTCCTTCCCC : 1710
AOTVOKIR3DL4*01    : ---------------------------------------------GGTTCTTCTTGGTCCAGAGGGCCTGGTCACGTGAGG-------------- :   67
AOTVOKIR3DL4*02    : ---------------------------------------------GGTTCTTCTTGGTCCAGAGGGCCTGGTCACGTGAGG-------------- :   67
AOTVOKIR3DL4*02v1  : ----------------------------------------------------------------------------------------------- :    -
AOTVOKIR3DL4*02v2  : ---------------------------------------------GGTTCTTCTTGGTCCAGAGGGCCTGGTCACGTGAGG-------------- :   67
AOTVOKIR3DL4*03    : ---------------------------------------------GGTTCTTCTTGGTCCAGAGGGCCTGGTCACGTGAGG-------------- :   67
AOTVOKIR3DS4*04    : ---------------------------------------------GGTTCTTCTTGGTCCAGAGGGCCTGGTCACGTGAGG-------------- :   67
AOTVOKIR3DS4*04v1  : ---------------------------------------------GGTTCTTCTTGGTCCAGAGGGCCTGGTCACGTGAGG-------------- :   67
AOTVOKIR3DS5*01    : ---------------------------------------------GGTTCTTCTTGGTCCAGAGGGCCTGGCCACATGAGG-------------- :   67
AOTVOKIR2DS5*01v1  : ---------------------------------------------GGTTCTTCTTGGTCCAGAGGGCCTGGCCACATGAGG-------------- :   67
AOTVOKIR2DS5*01v2  : ---------------------------------------------GGTTCTTCTTGGTCCAGAGGGCCTGGCCACATGAGG-------------- :   67
```

```
AOTVOKIR3DS7*01    : ---------------------------------------------GGTTCTTCTTGGTCCAGAGAGCCTGGCCACACGGGG-------------- :    67
AOTVOKIR3DS7*01v1  : ---------------------------------------------GGTTCTTCTTGGTCCAGAGAGCCTGGCCACACGGGG-------------- :    67
AOTVOKIR3DL8*01    : ---------------------------------------------GGTTCTTCTTGGTCCAGAGGGCCTGGCCACATGAGG-------------- :    67
AOTVOKIR3DL8*01v1  : ---------------------------------------------GGTTCTTCTTGGTCCAGAGGGCCTGGCCACATGAGG-------------- :    67
AOTVOKIR3DS8*01v2  : ---------------------------------------------GGTTCTTCTTGGTCCAGAGGGCCTGGCCACATGAGG-------------- :    67
AOTVOKIR3DL8*02    : ---------------------------------------------GGTTCTTCTTGGTCCAGAGGGCCTGGCCACATGAGG-------------- :    67
AOTVOKIR4DL9*01    : ---------------------------------------------GGTTCTTCTTGGTCCAGAGGGCCTGGCCACACGGGG-------------- :    67
AOTVOKIR4DS9*01v1  : ---------------------------------------------GGTTCTTCTTGGTCCAGAGGGCCTGGCCACACGGGG-------------- :    67
AOTVOKIR3DL9*01v2  : ---------------------------------------------GGTTCTTCTTGGTCCAGAGGGCCTGGCCACACGGGG-------------- :    67
AOTVOKIR4DL9*02    : ---------------------------------------------GGTTCTTCTTGGTCCAGAGGGCCTGGCCACACGGGG-------------- :    67
AOTVOKIR4DL10*01   : ---------------------------------------------GGTTCTTCTTGGTCCAGAGGGCCTGGTCACGTGAGG-------------- :    67
AOTVOKIR3DL10*01v1 : ---------------------------------------------GGTTCTTCTTGGTCCAGAGGGCCTGGTCACGTGAGG-------------- :    67
AOTVOKIR4DS10*01v2 : ---------------------------------------------GGTTCTTCTTGGTCCAGAGGGCCTGGTCACGTGAGG-------------- :    67
AOTVOKIR3DS10*01v3 : ---------------------------------------------GGTTCTTCTTGGTCCAGAGGGCCTGGTCACGTGAGG-------------- :    67
AOTVOKIR3DS10*01v4 : ---------------------------------------------GGTTCTTCTTGGTCCAGAGGGCCTGGTCACGTGAGG-------------- :    67
                                                                  ggttcttcttggtccagagggcctgg cac  g gg

                           1720         *      1740         *      1760         *      1780         *      1800
BAC_clone_Om       : AAACCTTAGGGTGTCATCTTCCTACATAAGGGGGTTTCCTGAAACAGAAGAAAAATCCTGTGGGGGCGTCTCTCATAAACTAGGAAGAGGGGACC :  1805
AOTVOKIR3DL4*01    : ----------------------------------------------------------------------------------------------- :     -
AOTVOKIR3DL4*02    : ----------------------------------------------------------------------------------------------- :     -
AOTVOKIR3DL4*02v1  : ----------------------------------------------------------------------------------------------- :     -
AOTVOKIR3DL4*02v2  : ----------------------------------------------------------------------------------------------- :     -
AOTVOKIR3DL4*03    : ----------------------------------------------------------------------------------------------- :     -
AOTVOKIR3DS4*04    : ----------------------------------------------------------------------------------------------- :     -
AOTVOKIR3DS4*04v1  : ----------------------------------------------------------------------------------------------- :     -
AOTVOKIR3DS5*01    : ----------------------------------------------------------------------------------------------- :     -
AOTVOKIR2DS5*01v1  : ----------------------------------------------------------------------------------------------- :     -
AOTVOKIR2DS5*01v2  : ----------------------------------------------------------------------------------------------- :     -
AOTVOKIR3DS7*01    : ----------------------------------------------------------------------------------------------- :     -
AOTVOKIR3DS7*01v1  : ----------------------------------------------------------------------------------------------- :     -
AOTVOKIR3DL8*01    : ----------------------------------------------------------------------------------------------- :     -
AOTVOKIR3DL8*01v1  : ----------------------------------------------------------------------------------------------- :     -
AOTVOKIR3DS8*01v2  : ----------------------------------------------------------------------------------------------- :     -
AOTVOKIR3DL8*02    : ----------------------------------------------------------------------------------------------- :     -
AOTVOKIR4DL9*01    : ----------------------------------------------------------------------------------------------- :     -
AOTVOKIR4DS9*01v1  : ----------------------------------------------------------------------------------------------- :     -
AOTVOKIR3DL9*01v2  : ----------------------------------------------------------------------------------------------- :     -
AOTVOKIR4DL9*02    : ----------------------------------------------------------------------------------------------- :     -
AOTVOKIR4DL10*01   : ----------------------------------------------------------------------------------------------- :     -
AOTVOKIR3DL10*01v1 : ----------------------------------------------------------------------------------------------- :     -
AOTVOKIR4DS10*01v2 : ----------------------------------------------------------------------------------------------- :     -
AOTVOKIR3DS10*01v3 : ----------------------------------------------------------------------------------------------- :     -
AOTVOKIR3DS10*01v4 : ----------------------------------------------------------------------------------------------- :     -
```

```
                            *      1820         *      1840         *      1860         *      1880         *      1900
BAC_clone_Om         : CTGGGATGCTCGGCCCACAGTTCTGACCTAGCCCTTCCCAGCCTTCCTTCCCTTGGCTGAGTCAAGCTCTGTGGGGACCGGGGTGAGACTGGGGT :  1900
AOTVOKIR3DL4*01      : ----------------------------------------------------------------------------------------------- :     -
AOTVOKIR3DL4*02      : ----------------------------------------------------------------------------------------------- :     -
AOTVOKIR3DL4*02v1    : ----------------------------------------------------------------------------------------------- :     -
AOTVOKIR3DL4*02v2    : ----------------------------------------------------------------------------------------------- :     -
AOTVOKIR3DL4*03      : ----------------------------------------------------------------------------------------------- :     -
AOTVOKIR3DS4*04      : ----------------------------------------------------------------------------------------------- :     -
AOTVOKIR3DS4*04v1    : ----------------------------------------------------------------------------------------------- :     -
AOTVOKIR3DS5*01      : ----------------------------------------------------------------------------------------------- :     -
AOTVOKIR2DS5*01v1    : ----------------------------------------------------------------------------------------------- :     -
AOTVOKIR2DS5*01v2    : ----------------------------------------------------------------------------------------------- :     -
AOTVOKIR3DS7*01      : ----------------------------------------------------------------------------------------------- :     -
AOTVOKIR3DS7*01v1    : ----------------------------------------------------------------------------------------------- :     -
AOTVOKIR3DL8*01      : ----------------------------------------------------------------------------------------------- :     -
AOTVOKIR3DL8*01v1    : ----------------------------------------------------------------------------------------------- :     -
AOTVOKIR3DS8*01v2    : ----------------------------------------------------------------------------------------------- :     -
AOTVOKIR3DL8*02      : ----------------------------------------------------------------------------------------------- :     -
AOTVOKIR4DL9*01      : ----------------------------------------------------------------------------------------------- :     -
AOTVOKIR4DS9*01v1    : ----------------------------------------------------------------------------------------------- :     -
AOTVOKIR3DL9*01v2    : ----------------------------------------------------------------------------------------------- :     -
AOTVOKIR4DL9*02      : ----------------------------------------------------------------------------------------------- :     -
AOTVOKIR4DL10*01     : ----------------------------------------------------------------------------------------------- :     -
AOTVOKIR3DL10*01v1   : ----------------------------------------------------------------------------------------------- :     -
AOTVOKIR4DS10*01v2   : ----------------------------------------------------------------------------------------------- :     -
AOTVOKIR3DS10*01v3   : ----------------------------------------------------------------------------------------------- :     -
AOTVOKIR3DS10*01v4   : ----------------------------------------------------------------------------------------------- :     -

                                 *      1920         *      1940         *      1960         *      1980         *
BAC_clone_Om         : GCTCCAAGCTGGGGTGTGAGGGGAGGAAGGGGTGTCACCAGCAGAGGAAGGGAGGGAAGCAGTGCTAGGAAAGGCCGACCCTCTGAGGACAAAGG :  1995
AOTVOKIR3DL4*01      : ----------------------------------------------------------------------------------------------- :     -
AOTVOKIR3DL4*02      : ----------------------------------------------------------------------------------------------- :     -
AOTVOKIR3DL4*02v1    : ----------------------------------------------------------------------------------------------- :     -
AOTVOKIR3DL4*02v2    : ----------------------------------------------------------------------------------------------- :     -
AOTVOKIR3DL4*03      : ----------------------------------------------------------------------------------------------- :     -
AOTVOKIR3DS4*04      : ----------------------------------------------------------------------------------------------- :     -
AOTVOKIR3DS4*04v1    : ----------------------------------------------------------------------------------------------- :     -
AOTVOKIR3DS5*01      : ----------------------------------------------------------------------------------------------- :     -
AOTVOKIR2DS5*01v1    : ----------------------------------------------------------------------------------------------- :     -
AOTVOKIR2DS5*01v2    : ----------------------------------------------------------------------------------------------- :     -
AOTVOKIR3DS7*01      : ----------------------------------------------------------------------------------------------- :     -
AOTVOKIR3DS7*01v1    : ----------------------------------------------------------------------------------------------- :     -
AOTVOKIR3DL8*01      : ----------------------------------------------------------------------------------------------- :     -
AOTVOKIR3DL8*01v1    : ----------------------------------------------------------------------------------------------- :     -
AOTVOKIR3DS8*01v2    : ----------------------------------------------------------------------------------------------- :     -
```

```
AOTVOKIR3DL8*02     : ----------------------------------------------------------------------------------------------- :     -
AOTVOKIR4DL9*01     : ----------------------------------------------------------------------------------------------- :     -
AOTVOKIR4DS9*01v1   : ----------------------------------------------------------------------------------------------- :     -
AOTVOKIR3DL9*01v2   : ----------------------------------------------------------------------------------------------- :     -
AOTVOKIR4DL9*02     : ----------------------------------------------------------------------------------------------- :     -
AOTVOKIR4DL10*01    : ----------------------------------------------------------------------------------------------- :     -
AOTVOKIR3DL10*01v1  : ----------------------------------------------------------------------------------------------- :     -
AOTVOKIR4DS10*01v2  : ----------------------------------------------------------------------------------------------- :     -
AOTVOKIR3DS10*01v3  : ----------------------------------------------------------------------------------------------- :     -
AOTVOKIR3DS10*01v4  : ----------------------------------------------------------------------------------------------- :     -

                         2000         *       2020         *       2040         *       2060         *       2080         *
BAC_clone_Om        : TGTAACTCACACCCTCCAGCGTTTCCATGACGGTAGGGGCTGCCGTGTGACTGCTGTCATTCTACCAGGAGAGGTGGGGGGACCACAGCCGTGAG :  2090
AOTVOKIR3DL4*01     : ----------------------------------------------------------------------------------------------- :     -
AOTVOKIR3DL4*02     : ----------------------------------------------------------------------------------------------- :     -
AOTVOKIR3DL4*02v1   : ----------------------------------------------------------------------------------------------- :     -
AOTVOKIR3DL4*02v2   : ----------------------------------------------------------------------------------------------- :     -
AOTVOKIR3DL4*03     : ----------------------------------------------------------------------------------------------- :     -
AOTVOKIR3DS4*04     : ----------------------------------------------------------------------------------------------- :     -
AOTVOKIR3DS4*04v1   : ----------------------------------------------------------------------------------------------- :     -
AOTVOKIR3DS5*01     : ----------------------------------------------------------------------------------------------- :     -
AOTVOKIR2DS5*01v1   : ----------------------------------------------------------------------------------------------- :     -
AOTVOKIR2DS5*01v2   : ----------------------------------------------------------------------------------------------- :     -
AOTVOKIR3DS7*01     : ----------------------------------------------------------------------------------------------- :     -
AOTVOKIR3DS7*01v1   : ----------------------------------------------------------------------------------------------- :     -
AOTVOKIR3DL8*01     : ----------------------------------------------------------------------------------------------- :     -
AOTVOKIR3DL8*01v1   : ----------------------------------------------------------------------------------------------- :     -
AOTVOKIR3DS8*01v2   : ----------------------------------------------------------------------------------------------- :     -
AOTVOKIR3DL8*02     : ----------------------------------------------------------------------------------------------- :     -
AOTVOKIR4DL9*01     : ----------------------------------------------------------------------------------------------- :     -
AOTVOKIR4DS9*01v1   : ----------------------------------------------------------------------------------------------- :     -
AOTVOKIR3DL9*01v2   : ----------------------------------------------------------------------------------------------- :     -
AOTVOKIR4DL9*02     : ----------------------------------------------------------------------------------------------- :     -
AOTVOKIR4DL10*01    : ----------------------------------------------------------------------------------------------- :     -
AOTVOKIR3DL10*01v1  : ----------------------------------------------------------------------------------------------- :     -
AOTVOKIR4DS10*01v2  : ----------------------------------------------------------------------------------------------- :     -
AOTVOKIR3DS10*01v3  : ----------------------------------------------------------------------------------------------- :     -
AOTVOKIR3DS10*01v4  : ----------------------------------------------------------------------------------------------- :     -

                              2100         *       2120         *       2140         *       2160         *       2180
BAC_clone_Om        : CTTCACATTCTAAATCCTCTGATGGGGGCTCAGTTGTTTATTGTGGTTCACGCAGTGGCTGATACTCCATTCACAAAGGACCTGCCCCCACCCCG :  2185
AOTVOKIR3DL4*01     : ----------------------------------------------------------------------------------------------- :     -
AOTVOKIR3DL4*02     : ----------------------------------------------------------------------------------------------- :     -
AOTVOKIR3DL4*02v1   : ----------------------------------------------------------------------------------------------- :     -
```

```
AOTVOKIR3DL4*02v2  : ----------------------------------------------------------------------------------------------- :     -
AOTVOKIR3DL4*03    : ----------------------------------------------------------------------------------------------- :     -
AOTVOKIR3DS4*04    : ----------------------------------------------------------------------------------------------- :     -
AOTVOKIR3DS4*04v1  : ----------------------------------------------------------------------------------------------- :     -
AOTVOKIR3DS5*01    : ----------------------------------------------------------------------------------------------- :     -
AOTVOKIR2DS5*01v1  : ----------------------------------------------------------------------------------------------- :     -
AOTVOKIR2DS5*01v2  : ----------------------------------------------------------------------------------------------- :     -
AOTVOKIR3DS7*01    : ----------------------------------------------------------------------------------------------- :     -
AOTVOKIR3DS7*01v1  : ----------------------------------------------------------------------------------------------- :     -
AOTVOKIR3DL8*01    : ----------------------------------------------------------------------------------------------- :     -
AOTVOKIR3DL8*01v1  : ----------------------------------------------------------------------------------------------- :     -
AOTVOKIR3DS8*01v2  : ----------------------------------------------------------------------------------------------- :     -
AOTVOKIR3DL8*02    : ----------------------------------------------------------------------------------------------- :     -
AOTVOKIR4DL9*01    : ----------------------------------------------------------------------------------------------- :     -
AOTVOKIR4DS9*01v1  : ----------------------------------------------------------------------------------------------- :     -
AOTVOKIR3DL9*01v2  : ----------------------------------------------------------------------------------------------- :     -
AOTVOKIR4DL9*02    : ----------------------------------------------------------------------------------------------- :     -
AOTVOKIR4DL10*01   : ----------------------------------------------------------------------------------------------- :     -
AOTVOKIR3DL10*01v1 : ----------------------------------------------------------------------------------------------- :     -
AOTVOKIR4DS10*01v2 : ----------------------------------------------------------------------------------------------- :     -
AOTVOKIR3DS10*01v3 : ----------------------------------------------------------------------------------------------- :     -
AOTVOKIR3DS10*01v4 : ----------------------------------------------------------------------------------------------- :     -

                          *        2200          *        2220          *        2240          *        2260          *        2280
BAC_clone_Om       : TGTCTACCTCGTGTTGTTTTATGTAAGCAATTTTGCCGTATTAAAATCTAGTAAGAGTCCCTCATTCAGCACTTACTAAAAGTTCTCAGCTGACA :  2280
AOTVOKIR3DL4*01    : ----------------------------------------------------------------------------------------------- :     -
AOTVOKIR3DL4*02    : ----------------------------------------------------------------------------------------------- :     -
AOTVOKIR3DL4*02v1  : ----------------------------------------------------------------------------------------------- :     -
AOTVOKIR3DL4*02v2  : ----------------------------------------------------------------------------------------------- :     -
AOTVOKIR3DL4*03    : ----------------------------------------------------------------------------------------------- :     -
AOTVOKIR3DS4*04    : ----------------------------------------------------------------------------------------------- :     -
AOTVOKIR3DS4*04v1  : ----------------------------------------------------------------------------------------------- :     -
AOTVOKIR3DS5*01    : ----------------------------------------------------------------------------------------------- :     -
AOTVOKIR2DS5*01v1  : ----------------------------------------------------------------------------------------------- :     -
AOTVOKIR2DS5*01v2  : ----------------------------------------------------------------------------------------------- :     -
AOTVOKIR3DS7*01    : ----------------------------------------------------------------------------------------------- :     -
AOTVOKIR3DS7*01v1  : ----------------------------------------------------------------------------------------------- :     -
AOTVOKIR3DL8*01    : ----------------------------------------------------------------------------------------------- :     -
AOTVOKIR3DL8*01v1  : ----------------------------------------------------------------------------------------------- :     -
AOTVOKIR3DS8*01v2  : ----------------------------------------------------------------------------------------------- :     -
AOTVOKIR3DL8*02    : ----------------------------------------------------------------------------------------------- :     -
AOTVOKIR4DL9*01    : ----------------------------------------------------------------------------------------------- :     -
AOTVOKIR4DS9*01v1  : ----------------------------------------------------------------------------------------------- :     -
AOTVOKIR3DL9*01v2  : ----------------------------------------------------------------------------------------------- :     -
AOTVOKIR4DL9*02    : ----------------------------------------------------------------------------------------------- :     -
```

```
AOTVOKIR4DL10*01    : ----------------------------------------------------------------------------------------------- :     -
AOTVOKIR3DL10*01v1  : ----------------------------------------------------------------------------------------------- :     -
AOTVOKIR4DS10*01v2  : ----------------------------------------------------------------------------------------------- :     -
AOTVOKIR3DS10*01v3  : ----------------------------------------------------------------------------------------------- :     -
AOTVOKIR3DS10*01v4  : ----------------------------------------------------------------------------------------------- :     -

                                *       2300         *       2320         *       2340         *       2360         *
BAC_clone_Om        : CGTTTGTTGTAGGGAGACGCCATGTCTATGCAGGATGGGACTTTCCTGTAGCTCTGGGCACCCAGGTGTGGTAGGAGCCTTAGAAACGTGGGAAA :  2375
AOTVOKIR3DL4*01     : ----------------------------------------------------------------------------------------------- :     -
AOTVOKIR3DL4*02     : ----------------------------------------------------------------------------------------------- :     -
AOTVOKIR3DL4*02v1   : ----------------------------------------------------------------------------------------------- :     -
AOTVOKIR3DL4*02v2   : ----------------------------------------------------------------------------------------------- :     -
AOTVOKIR3DL4*03     : ----------------------------------------------------------------------------------------------- :     -
AOTVOKIR3DS4*04     : ----------------------------------------------------------------------------------------------- :     -
AOTVOKIR3DS4*04v1   : ----------------------------------------------------------------------------------------------- :     -
AOTVOKIR3DS5*01     : ----------------------------------------------------------------------------------------------- :     -
AOTVOKIR2DS5*01v1   : ----------------------------------------------------------------------------------------------- :     -
AOTVOKIR2DS5*01v2   : ----------------------------------------------------------------------------------------------- :     -
AOTVOKIR3DS7*01     : ----------------------------------------------------------------------------------------------- :     -
AOTVOKIR3DS7*01v1   : ----------------------------------------------------------------------------------------------- :     -
AOTVOKIR3DL8*01     : ----------------------------------------------------------------------------------------------- :     -
AOTVOKIR3DL8*01v1   : ----------------------------------------------------------------------------------------------- :     -
AOTVOKIR3DS8*01v2   : ----------------------------------------------------------------------------------------------- :     -
AOTVOKIR3DL8*02     : ----------------------------------------------------------------------------------------------- :     -
AOTVOKIR4DL9*01     : ----------------------------------------------------------------------------------------------- :     -
AOTVOKIR4DS9*01v1   : ----------------------------------------------------------------------------------------------- :     -
AOTVOKIR3DL9*01v2   : ----------------------------------------------------------------------------------------------- :     -
AOTVOKIR4DL9*02     : ----------------------------------------------------------------------------------------------- :     -
AOTVOKIR4DL10*01    : ----------------------------------------------------------------------------------------------- :     -
AOTVOKIR3DL10*01v1  : ----------------------------------------------------------------------------------------------- :     -
AOTVOKIR4DS10*01v2  : ----------------------------------------------------------------------------------------------- :     -
AOTVOKIR3DS10*01v3  : ----------------------------------------------------------------------------------------------- :     -
AOTVOKIR3DS10*01v4  : ----------------------------------------------------------------------------------------------- :     -

                        2380         *       2400         *       2420         *       2440         *      2460         *
BAC_clone_Om        : AGGGAGAATCTTCTGAGCACAGGGAGGGAGGGGCGGCTCCACATCTTCCTCTCTAAGGCGACACCTCCTCCTCCCCCAGGTGATCAGGACAAGCC :  2470
AOTVOKIR3DL4*01     : -------------------------------------------------------------------------------GTGGTCAGGACCAGCC :    83
AOTVOKIR3DL4*02     : -------------------------------------------------------------------------------GTGGTCAGGACCAGCC :    83
AOTVOKIR3DL4*02v1   : -------------------------------------------------------------------------------GTGGTCAGGACCAGCC :    47
AOTVOKIR3DL4*02v2   : -------------------------------------------------------------------------------GTGGTCAGGACCAGCC :    83
AOTVOKIR3DL4*03     : -------------------------------------------------------------------------------GTGGTCAGGACCAGCC :    83
AOTVOKIR3DS4*04     : -------------------------------------------------------------------------------GTGGTCAGGACCAGCC :    83
AOTVOKIR3DS4*04v1   : -------------------------------------------------------------------------------GTGGTCAGGACCAGCC :    83
AOTVOKIR3DS5*01     : -------------------------------------------------------------------------------ATGGTCAGGAGCAGCC :    83
```

```
AOTVOKIR2DS5*01v1  : --------------------------------------------------------------------------------ATGGTCAGGAGCAGCC :   83
AOTVOKIR2DS5*01v2  : ------------------------------------------------------------------------------------------------ :    -
AOTVOKIR3DS7*01    : --------------------------------------------------------------------------------GTAGTCAGGACCAGCC :   83
AOTVOKIR3DS7*01v1  : --------------------------------------------------------------------------------GTAGTCAGGACCAGCC :   83
AOTVOKIR3DL8*01    : --------------------------------------------------------------------------------GTGGTCAGGACGAGCC :   83
AOTVOKIR3DL8*01v1  : --------------------------------------------------------------------------------GTGGTCAGGACGAGCC :   83
AOTVOKIR3DS8*01v2  : --------------------------------------------------------------------------------GTGGTCAGGACGAGCC :   83
AOTVOKIR3DL8*02    : --------------------------------------------------------------------------------GTGGTCAGGACGAGCC :   83
AOTVOKIR4DL9*01    : --------------------------------------------------------------------------------GTGGTGATGACCAGCC :   83
AOTVOKIR4DS9*01v1  : --------------------------------------------------------------------------------GTGGTGATGACCAGCC :   83
AOTVOKIR3DL9*01v2  : --------------------------------------------------------------------------------GTGGTGATGACCAGCC :   83
AOTVOKIR4DL9*02    : --------------------------------------------------------------------------------GTGGTGATGACCAGCC :   83
AOTVOKIR4DL10*01   : --------------------------------------------------------------------------------GTGGTCAGGACCAGCC :   83
AOTVOKIR3DL10*01v1 : --------------------------------------------------------------------------------GTGGTCAGGACCAGCC :   83
AOTVOKIR4DS10*01v2 : --------------------------------------------------------------------------------GTGGTCAGGACCAGCC :   83
AOTVOKIR3DS10*01v3 : --------------------------------------------------------------------------------GTGGTCAGGACCAGCC :   83
AOTVOKIR3DS10*01v4 : --------------------------------------------------------------------------------GTGGTCAGGACCAGCC :   83
                                               EXON 3a                                          gtggtcaggac agcc

                           2480                *        2500                *        2520                *        2540       *        2560
BAC_clone_Om       : CTTCCTGTCTGCCTGGCCCAGCGCTGTGGTGTCTCTAGGAGGACATGTGACTCTTCGGTGTCACTATGGTCGTGGGTTTAACAACTTCACCCTGT : 2565
AOTVOKIR3DL4*01    : CTTCCTGTCTGCCTGGCCCAGCGCTGTGGTGTCTCCAGGAGGACACGTGACTCTTCGGTGTCACTATGGTGATGGGTTTAACAACTTCACCCTGT :  178
AOTVOKIR3DL4*02    : CTTCCTGTCTGCCTGGCCCAGCGCTGTGGTGTCTCCAGGAGGACACGTGACTCTTCGGTGTCACTATGGTGATGGGTTTAACAACTTCACCCTGT :  178
AOTVOKIR3DL4*02v1  : CTTCCTGTCTGCCTGGCCCAGCGCTGTGGTGTCTCCAGGAGGACACGTGACTCTTCGGTGTCACTATGGTGATGGGTTTAACAACTTCACCCTGT :  142
AOTVOKIR3DL4*02v2  : CTTCCTGTCTGCCTGGCCCAGCGCTGTGGTGTCTCCAGGAGGACACGTGACTCTTCGGTGTCACTATGGTGATGGGTTTAACAACTTCACCCTGT :  178
AOTVOKIR3DL4*03    : CTTCCTGTCTGCCTGGCCCAGCGCTGTGGTGTCTCCAGGAGGACACGTGACTCTTCGGTGTCACTATGGTGATGGGTTTAACAACTTCACCCTGT :  178
AOTVOKIR3DS4*04    : CTTCCTGTCTGCCTGGCCCAGCGCTGTGGTGTCTCCAGGAGGACACGTGACTCTTCGGTGTCACTATGGTGATGGGTTTAACAACTTCACCCTGT :  178
AOTVOKIR3DS4*04v1  : CTTCCTGTCTGCCTGGCCCAGCGCTGTGGTGTCTCCAGGAGGACACGTGACTCTTCGGTGTCACTATGGTGATGGGTTTAACAACTTCACCCTGT :  178
AOTVOKIR3DS5*01    : CTTCCTGTCTGCCTGGCCTAGCGCTGTGGTGACTCGAGGAGGACACGTGACTCTTCGGTGTCACTATGGTGGTGGGTTTAACAACTTCACCCTGT :  178
AOTVOKIR2DS5*01v1  : CTTCCTGTCTGCCTGGCCTAGCGCTGTGGTGACTCGAGGAGGACACGTGACTCTTCGGTGTCACTATGGTG------------------------ :  154
AOTVOKIR2DS5*01v2  : ----------------------------------------------------------------------------------------------- :    -
AOTVOKIR3DS7*01    : CTTCCTGTCTGCCTGGCCCAGCGCTGTGATGTCTCGAGGAGGAAATGTAACTCTTCGGTGTGACTATGGTGGTGGGTTTAACAACTTCACCCTGT :  178
AOTVOKIR3DS7*01v1  : CTTCCTGTCTGCCTGGCCCAGCGCTGTGATGTCTCGAGGAGGAAATGTAACTCTTCGGTGTGACTATGGTGGTGGGTTTAACAACTTCACCCTGT :  178
AOTVOKIR3DL8*01    : CTTCCTGTCTGCCTGGCCCAGCGCTGTGGTGTCTCGAGGAGGACATGTGACTCTTCGGTGTCACTATGATGGTGGGTTTAACAACTTCACCCTGT :  178
AOTVOKIR3DL8*01v1  : CTTCCTGTCTGCCTGGCCCAGCGCTGTGGTGTCTCGAGGAGGACATGTGACTCTTCGGTGTCACTATGATGGTGGGTTTAACAACTTCACCCTGT :  178
AOTVOKIR3DS8*01v2  : CTTCCTGTCTGCCTGGCCCAGCGCTGTGGTGTCTCGAGGAGGACATGTGACTCTTCGGTGTCACTATGATGGTGGGTTTAACAACTTCACCCTGT :  178
AOTVOKIR3DL8*02    : CTTCCTGTCTGCCTGGCCCAGCGCTGTGGTGTCTCGAGGAGGACATGTGACTCTTCGGTGTCACTATGATGGTGGGTTTAACAACTTCACCCTGT :  178
AOTVOKIR4DL9*01    : CTTCCTGTCTGCCTGGCCCAGCGCTGTGGTGTCTCAAGGAGGACATGTGACTCTTCGGTGTCACTATGGTGGTGGGTTTAACAACTTCACCCTGT :  178
AOTVOKIR4DS9*01v1  : CTTCCTGTCTGCCTGGCCCAGCGCTGTGGTGTCTCAAGGAGGACATGTGACTCTTCGGTGTCACTATGGTGGTGGGTTTAACAACTTCACCCTGT :  178
AOTVOKIR3DL9*01v2  : CTTCCTGTCTGCCTGGCCCAGCGCTGTGGTGTCTCAAGGAGGACATGTGACTCTTCGGTGTCACTATGGTG------------------------ :  154
AOTVOKIR4DL9*02    : CTTCCTGTCTGCCTGGCCCAGCGCTGTGGTGTCTCAAGGAGGACATGTGACTCTTCGGTGTCACTATGGTGGTGGGTTTAACAACTTCACCCTGT :  178
AOTVOKIR4DL10*01   : CTTCCTGTCTGCCTGGCCCAGCGCTGTGGTGTCTCGAGGAGGACACGTGACTCTTCGGTGTCACTATGGTGATGGGTTTAACAACTTCACCCTGT :  178
AOTVOKIR3DL10*01v1 : CTTCCTGTCTGCCTGGCCCAGCGCTGTGGTGTCTCGAGGAGGACACGTGACTCTTCGGTGTCACTATG--------------------------- :  151
AOTVOKIR4DS10*01v2 : CTTCCTGTCTGCCTGGCCCAGCGCTGTGGTGTCTCGAGGAGGACACGTGACTCTTCGGTGTCACTATGGTGATGGGTTTAACAACTTCACCCTGT :  178
AOTVOKIR3DS10*01v3 : CTTCCTGTCTGCCTGGCCCAGCGCTGTGGTGTCTCGAGGAGGACACGTGACTCTTCGGTGTCACTATGGTGATGGGTTTAACAACTTCACCCTGT :  178
AOTVOKIR3DS10*01v4 : CTTCCTGTCTGCCTGGCCCAGCGCTGTGGTGTCTCGAGGAGGACACGTGACTCTTCGGTGTCACTATGGTGATGGGTTTAACAACTTCACCCTGT :  178
```

```
                       cttcctgtctgcctggcccagcgctgtggtgtctc aggaggaca gtgactcttcggtgtcactatg tg tgggtttaacaacttcaccctgt

                           *       2580         *       2600         *       2620         *       2640         *       2660
BAC_clone_Om          : ACAAAGAAGACAGATTCCACGTTCCCATCCTCAATGGCATTATATTCCAGGAGAGCTTCGTCATAGGCCCCGTGACTGCAGCAGACGCAGGGACC :  2660
AOTVOKIR3DL4*01       : ACAAAGAAGACAGATTCCACGTTCCCATCCTCAACGGCGTTATATTCCAGGAGAGCTTCCTCATGGGCCCCGTGACTGCAGCACATGCAGGGACC :   273
AOTVOKIR3DL4*02       : ACAAAGAAGACAGATTCCACGTTCCCATCCTCAACGGCGTTATATTCCAGGAGAGCTTCCTCATGGGCCCCGTGACTGCAGCACATGCAGGGACC :   273
AOTVOKIR3DL4*02v1     : ACAAAGAAGACAGATTCCACGTTCCCATCCTCAACGGCGTTATATTCCAGGAGAGCTTCCTCATGGGCCCCGTGACTGCAGCACATGCAGGGACC :   237
AOTVOKIR3DL4*02v2     : ACAAAGAAGACAGATTCCACGTTCCCATCCTCAACGGCGTTATATTCCAGGAGAGCTTCCTCATGGGCCCCGTGACTGCAGCACATGCAGGGACC :   273
AOTVOKIR3DL4*03       : ACAAAGAAGACAGATTCCACGTTCCCATCCTCAACGGCGTTATATTCCAGGAGAGCTTCCTCATGGGCCCCGTGACTGCAGCACATGCAGGGACC :   273
AOTVOKIR3DS4*04       : ACAAAGAAGACAGATTCCACGTTCCCATCCTCAACGGCGTTATATTCCAGGAGAGCTTCCTCATGGGCCCCGTGACTGCAGCACATGCAGGGACC :   273
AOTVOKIR3DS4*04v1     : ACAAAGAAGACAGATTCCACGTTCCCATCCTCAACGGCGTTATATTCCAGGAGAGCTTCCTCATGGGCCCCGTGACTGCAGCACATGCAGGGACC :   273
AOTVOKIR3DS5*01       : ACAAAGAAGACAGATTCCATGTTCCCATCCTGAACGGTGATATATTCCAGGAGAGCTTCCTCATGGGCCCCGTGACTGCAGCACACACAGGGACC :   273
AOTVOKIR2DS5*01v1     : ----------------------------------------------------------------------------------------------- :     -
AOTVOKIR2DS5*01v2     : ----------------------------------------------------------------------------------------------- :     -
AOTVOKIR3DS7*01       : ACAAAGAAGACAGATTCCACGTTCCCATCCTCAACGGCGTAATATTCCATGAGAGCTTCCTCATGGGCCCCGTGACTGCAGCACACGCAGGGACC :   273
AOTVOKIR3DS7*01v1     : ACAAAGAAGACAGATTCCACGTTCCCATCCTCAACGGCGTAATATTCCATGAGAGCTTCCTCATGGGCCCCGTGACTGCAGCACACGCAGGGACC :   273
AOTVOKIR3DL8*01       : ACAAAGAAGACAGATTCCACGTACCCATCCTCAACGGCGTTATATTCCATGAGAGCTTCCTCATGGGCCCGGTGACTGCAGCACACGCAGGGACC :   273
AOTVOKIR3DL8*01v1     : ACAAAGAAGACAGATTCCACGTACCCATCCTCAACGGCGTTATATTCCATGAGAGCTTCCTCATGGGCCCGGTGACTGCAGCACACGCAGGGACC :   273
AOTVOKIR3DS8*01v2     : ACAAAGAAGACAGATTCCACGTACCCATCCTCAACGGCGTTATATTCCATGAGAGCTTCCTCATGGGCCCGGTGACTGCAGCACACGCAGGGACC :   273
AOTVOKIR3DL8*02       : ACAAAGAAGACAGATTCCACGTACCCATCCTCAACGGCGTTATATTCCATGAGAGCTTCCTCATGGGCCCGGTGACTGCAGCACACGCAGGGACC :   273
AOTVOKIR4DL9*01       : ACAAAGAAGACAGATTCCATGTTCCCAACCTCAACGGCGTTATATTCCATGAGAGCTTCCTCATGGGCCCGGTGACTGCAGCACACGCAGGGACC :   273
AOTVOKIR4DS9*01v1     : ACAAAGAAGACAGATTCCATGTTCCCAACCTCAACGGCGTTATATTCCATGAGAGCTTCCTCATGGGCCCGGTGACTGCAGCACACGCAGGGACC :   273
AOTVOKIR3DL9*01v2     : ----------------------------------------------------------------------------------------------- :     -
AOTVOKIR4DL9*02       : ACAAAGAAGACAGATTCCATGTTCCCAACCTCAACGGCGTTATATTCCATGAGAGCTTCCTCATGGGCCCGGTGACTGCAGCACACGCAGGGACC :   273
AOTVOKIR4DL10*01      : ACAAAGAAGACAGATTCCACGTTCCCATCCTCAACGGTGTTATATTCCAGGAGAGCTTCCTCATGGGCCCCGTGACTGCAGCACATGCAGGGACC :   273
AOTVOKIR3DL10*01v1    : ----------------------------------------------------------------------------------------------- :     -
AOTVOKIR4DS10*01v2    : ACAAAGAAGACAGATTCCACGTTCCCATCCTCAACGGTGTTATATTCCAGGAGAGCTTCCTCATGGGCCCCGTGACTGCAGCACATGCAGGGACC :   273
AOTVOKIR3DS10*01v3    : ACAAAGAAGACAGATTCCACGTTCCCATCCTCAACGGTGTTATATTCCAGGAGAGCTTCCTCATGGGCCCCGTGACTGCAGCACACGCAGGGACC :   273
AOTVOKIR3DS10*01v4    : ACAAAGAAGACAGATTCCACGTTCCCATCCTCAACGGTGTTATATTCCAGGAGAGCTTCCTCATGGGCCCCGTGACTGCAGCACACGCAGGGACC :   273
                        acaaagaagacagattcca gt ccca cctcaacgg gt atattcca gagagcttcctcatgggccc gtgactgcagcaca gcagggacc

                                 *       2680         *       2700         *       2720         *       2740         *
BAC_clone_Om          : TACAGATGTCGGGGTTTTCAGCCGCACTCCCACATTCAATGGACAGCTCCCAGCAACCCCCTGAAGATCATAGTTACAGGTCAGAGGGCTCCTGT :  2755
AOTVOKIR3DL4*01       : TACAGATGTCGGGGTTTTCACCCGCACTCCCCCACTGGGCGGACGGCACACAGCAACCCCCTAAAGATCATAGTCACAG---------------- :   352
AOTVOKIR3DL4*02       : TACAGATGTCGGGGTTTTCACCCGCACTCCCCCACTGGGCGGACGGCACACAGCAACCCCCTAAAGATCATAGTCACAG---------------- :   352
AOTVOKIR3DL4*02v1     : TACAGATGTCGGGGTTTTCACCCGCACTCCCCCACTGGGCGGACGGCACACAGCAACCCCCTAAAGATCATAGTCACAG---------------- :   316
AOTVOKIR3DL4*02v2     : TACAGATGTCGGGGTTTTCACCCGCACTCCCCCACTGGGCGGACGGCACACAGCAACCCCCTAAAGATCATAGTCACAG---------------- :   352
AOTVOKIR3DL4*03       : TACAGATGTCGGGGTTTTCACCCGCACTCCCCCACTGGGCGGACGGCACACAGCAACCCCCTAAAGATCATAGTCACAG---------------- :   352
AOTVOKIR3DS4*04       : TACAGATGTCGGGGTTTTCACCCGCACTCCCCCACTGGGCGGACGGCACACAGCAACCCCCTAAAGATCATAGTCACAG---------------- :   352
AOTVOKIR3DS4*04v1     : TACAGATGTCGGGGTTTTCACCCGCACTCCCCCACTGGGCGGACGGCACACAGCAACCCCCTAAAGATCATAGTCACAG---------------- :   352
AOTVOKIR3DS5*01       : TACAGATGTCGGGGATTTCACCCGCACTCCCCCACTAGGCGGACAGCACCCAGCAACCCCCTGAAGATCATAGTCACAG---------------- :   352
AOTVOKIR2DS5*01v1     : ----------------------------------------------------------------------------------------------- :     -
AOTVOKIR2DS5*01v2     : ----------------------------------------------------------------------------------------------- :     -
AOTVOKIR3DS7*01       : TACAGATGTCGGGGTTTTCACCCGCACTCCCCCACTGGGTGGACGGCACCCAGCAAACCCCTGAAGATCATAGTCACAG---------------- :   352
AOTVOKIR3DS7*01v1     : TACAGATGTCGGGGTTTTCACCCGCACTCCCCCACTGGGTGGACGGCACCCAGCAAACCCCTGAAGATCATAGTCACAG---------------- :   352
AOTVOKIR3DL8*01       : TACAGATGTCGGGGTCTTCACCCGCACTCCCCCACTGGGCGGACGGCACCCAGCAACCCCCTGAAGATCATAGTCACAG---------------- :   352
```

```
AOTVOKIR3DL8*01v1  : TACAGATGTCGGGGTCTTCACCCGCACTCCCCCACTGGGCGGACGGCACCCAGCAACCCCCTGAAGATCATAGTCACAG---------------- :   352
AOTVOKIR3DS8*01v2  : TACAGATGTCGGGGTCTTCACCCGCACTCCCCCACTGGGCGGACGGCACCCAGCAACCCCCTGAAGATCATAGTCACAG---------------- :   352
AOTVOKIR3DL8*02    : TACAGATGTCGGGGTCTTCACCCGCACTCCCCCACTGGGCGGACGGCACCCAGCAACCCCCTGAAGATCATAGTCACAG---------------- :   352
AOTVOKIR4DL9*01    : TACAGATGTCGGGGTTTTCACCCGCACTCCTCCACTGGGTGGACAGCACCCAGCAACCCCCTGAAGATTATAGTCACAG---------------- :   352
AOTVOKIR4DS9*01v1  : TACAGATGTCGGGGTTTTCACCCGCACTCCTCCACTGGGTGGACAGCACCCAGCAACCCCCTGAAGATTATAGTCACAG---------------- :   352
AOTVOKIR3DL9*01v2  : ----------------------------------------------------------------------------------------------- :     -
AOTVOKIR4DL9*02    : TACAGATGTCGGGGTTTTCACCCGCACTCCTCCACTGGGTGGACAGCACCCAGCAACCCCCTGAAGATTATAGTCACAG---------------- :   352
AOTVOKIR4DL10*01   : TACAGATGTCGGGGTTTTCACCCGCACTCCCCCACTGGGCGGACGGCACACAGCAACCCCCTAAAGATCATAGTCACAG---------------- :   352
AOTVOKIR3DL10*01v1 : ----------------------------------------------------------------------------------------------- :     -
AOTVOKIR4DS10*01v2 : TACAGATGTCGGGGTTTTCACCCGCACTCCCCCACTGGGCGGACGGCACACAGCAACCCCCTAAAGATCATAGTCACAG---------------- :   352
AOTVOKIR3DS10*01v3 : TACAGATGTCGGCGTTTTCACCCGCACTCCTCCACTGGGCGGACGGCACCCAGCAACGCCCTGAAGATCATAGTCACAG---------------- :   352
AOTVOKIR3DS10*01v4 : TACAGATGTCGGCGTTTTCACCCGCACTCCTCCACTGGGCGGACGGCACCCAGCAACGCCCTGAAGATCATAGTCACAG---------------- :   352
                     tacagatgtcgg gt ttcacccgcactcc ccact gg ggac gcac cagcaa  ccct aagat atagtcacag

                      2760        *       2780        *       2800        *       2820        *       2840        *
BAC_clone_Om       : CTGGGCTTCTCACTGTCCTAACTCCTGAATCCCAGAGCTTCTGGTGGGTTGTCCATCAGGGTTTGATCACCCAGGCCCTGACTGTATTTGGGGTA :  2850
AOTVOKIR3DL4*01    : ----------------------------------------------------------------------------------------------- :     -
AOTVOKIR3DL4*02    : ----------------------------------------------------------------------------------------------- :     -
AOTVOKIR3DL4*02v1  : ----------------------------------------------------------------------------------------------- :     -
AOTVOKIR3DL4*02v2  : ----------------------------------------------------------------------------------------------- :     -
AOTVOKIR3DL4*03    : ----------------------------------------------------------------------------------------------- :     -
AOTVOKIR3DS4*04    : ----------------------------------------------------------------------------------------------- :     -
AOTVOKIR3DS4*04v1  : ----------------------------------------------------------------------------------------------- :     -
AOTVOKIR3DS5*01    : ----------------------------------------------------------------------------------------------- :     -
AOTVOKIR2DS5*01v1  : ----------------------------------------------------------------------------------------------- :     -
AOTVOKIR2DS5*01v2  : ----------------------------------------------------------------------------------------------- :     -
AOTVOKIR3DS7*01    : ----------------------------------------------------------------------------------------------- :     -
AOTVOKIR3DS7*01v1  : ----------------------------------------------------------------------------------------------- :     -
AOTVOKIR3DL8*01    : ----------------------------------------------------------------------------------------------- :     -
AOTVOKIR3DL8*01v1  : ----------------------------------------------------------------------------------------------- :     -
AOTVOKIR3DS8*01v2  : ----------------------------------------------------------------------------------------------- :     -
AOTVOKIR3DL8*02    : ----------------------------------------------------------------------------------------------- :     -
AOTVOKIR4DL9*01    : ----------------------------------------------------------------------------------------------- :     -
AOTVOKIR4DS9*01v1  : ----------------------------------------------------------------------------------------------- :     -
AOTVOKIR3DL9*01v2  : ----------------------------------------------------------------------------------------------- :     -
AOTVOKIR4DL9*02    : ----------------------------------------------------------------------------------------------- :     -
AOTVOKIR4DL10*01   : ----------------------------------------------------------------------------------------------- :     -
AOTVOKIR3DL10*01v1 : ----------------------------------------------------------------------------------------------- :     -
AOTVOKIR4DS10*01v2 : ----------------------------------------------------------------------------------------------- :     -
AOTVOKIR3DS10*01v3 : ----------------------------------------------------------------------------------------------- :     -
AOTVOKIR3DS10*01v4 : ----------------------------------------------------------------------------------------------- :     -


                           2860        *       2880        *       2900        *       2920        *       2940
BAC_clone_Om       : AAGGGGATTGAATACAGGGAAGTGGGTGCCGTGGTGGGAAGAATAACTGTCCCCACTGATGGCCACATTCTCATCCCTGGAGCCTGTGACTATTT :  2945
AOTVOKIR3DL4*01    : ----------------------------------------------------------------------------------------------- :     -
```

```
AOTVOKIR3DL4*02     : ----------------------------------------------------------------------------------------------- :    -
AOTVOKIR3DL4*02v1   : ----------------------------------------------------------------------------------------------- :    -
AOTVOKIR3DL4*02v2   : ----------------------------------------------------------------------------------------------- :    -
AOTVOKIR3DL4*03     : ----------------------------------------------------------------------------------------------- :    -
AOTVOKIR3DS4*04     : ----------------------------------------------------------------------------------------------- :    -
AOTVOKIR3DS4*04v1   : ----------------------------------------------------------------------------------------------- :    -
AOTVOKIR3DS5*01     : ----------------------------------------------------------------------------------------------- :    -
AOTVOKIR2DS5*01v1   : ----------------------------------------------------------------------------------------------- :    -
AOTVOKIR2DS5*01v2   : ----------------------------------------------------------------------------------------------- :    -
AOTVOKIR3DS7*01     : ----------------------------------------------------------------------------------------------- :    -
AOTVOKIR3DS7*01v1   : ----------------------------------------------------------------------------------------------- :    -
AOTVOKIR3DL8*01     : ----------------------------------------------------------------------------------------------- :    -
AOTVOKIR3DL8*01v1   : ----------------------------------------------------------------------------------------------- :    -
AOTVOKIR3DS8*01v2   : ----------------------------------------------------------------------------------------------- :    -
AOTVOKIR3DL8*02     : ----------------------------------------------------------------------------------------------- :    -
AOTVOKIR4DL9*01     : ----------------------------------------------------------------------------------------------- :    -
AOTVOKIR4DS9*01v1   : ----------------------------------------------------------------------------------------------- :    -
AOTVOKIR3DL9*01v2   : ----------------------------------------------------------------------------------------------- :    -
AOTVOKIR4DL9*02     : ----------------------------------------------------------------------------------------------- :    -
AOTVOKIR4DL10*01    : ----------------------------------------------------------------------------------------------- :    -
AOTVOKIR3DL10*01v1  : ----------------------------------------------------------------------------------------------- :    -
AOTVOKIR4DS10*01v2  : ----------------------------------------------------------------------------------------------- :    -
AOTVOKIR3DS10*01v3  : ----------------------------------------------------------------------------------------------- :    -
AOTVOKIR3DS10*01v4  : ----------------------------------------------------------------------------------------------- :    -

                          *      2960         *      2980         *      3000         *      3020         *      3040
BAC_clone_Om        : ATGTTACAGGGCAGGGGACTGAAGGGGGAAGATGGAGCTCAGGTTGTTGATGAGTTGACGTTGAGATGGGGAGGCTGCCTGGACTGTCCAGCTGT : 3040
AOTVOKIR3DL4*01     : ----------------------------------------------------------------------------------------------- :    -
AOTVOKIR3DL4*02     : ----------------------------------------------------------------------------------------------- :    -
AOTVOKIR3DL4*02v1   : ----------------------------------------------------------------------------------------------- :    -
AOTVOKIR3DL4*02v2   : ----------------------------------------------------------------------------------------------- :    -
AOTVOKIR3DL4*03     : ----------------------------------------------------------------------------------------------- :    -
AOTVOKIR3DS4*04     : ----------------------------------------------------------------------------------------------- :    -
AOTVOKIR3DS4*04v1   : ----------------------------------------------------------------------------------------------- :    -
AOTVOKIR3DS5*01     : ----------------------------------------------------------------------------------------------- :    -
AOTVOKIR2DS5*01v1   : ----------------------------------------------------------------------------------------------- :    -
AOTVOKIR2DS5*01v2   : ----------------------------------------------------------------------------------------------- :    -
AOTVOKIR3DS7*01     : ----------------------------------------------------------------------------------------------- :    -
AOTVOKIR3DS7*01v1   : ----------------------------------------------------------------------------------------------- :    -
AOTVOKIR3DL8*01     : ----------------------------------------------------------------------------------------------- :    -
AOTVOKIR3DL8*01v1   : ----------------------------------------------------------------------------------------------- :    -
AOTVOKIR3DS8*01v2   : ----------------------------------------------------------------------------------------------- :    -
AOTVOKIR3DL8*02     : ----------------------------------------------------------------------------------------------- :    -
AOTVOKIR4DL9*01     : ----------------------------------------------------------------------------------------------- :    -
AOTVOKIR4DS9*01v1   : ----------------------------------------------------------------------------------------------- :    -
```

```
AOTVOKIR3DL9*01v2  : ----------------------------------------------------------------------------------------------- :     -
AOTVOKIR4DL9*02    : ----------------------------------------------------------------------------------------------- :     -
AOTVOKIR4DL10*01   : ----------------------------------------------------------------------------------------------- :     -
AOTVOKIR3DL10*01v1 : ----------------------------------------------------------------------------------------------- :     -
AOTVOKIR4DS10*01v2 : ----------------------------------------------------------------------------------------------- :     -
AOTVOKIR3DS10*01v3 : ----------------------------------------------------------------------------------------------- :     -
AOTVOKIR3DS10*01v4 : ----------------------------------------------------------------------------------------------- :     -

                              *      3060         *      3080         *      3100         *      3120         *
BAC_clone_Om       : GCTCAGTGTCATCACAAGTGCCCACATGAGAGGAGGAGAAAGAGGGGAGTGCAGATTAGAGCAGCGTAGTGGGAGGGAGACTCCACCAGCCACTT :  3135
AOTVOKIR3DL4*01    : ----------------------------------------------------------------------------------------------- :     -
AOTVOKIR3DL4*02    : ----------------------------------------------------------------------------------------------- :     -
AOTVOKIR3DL4*02v1  : ----------------------------------------------------------------------------------------------- :     -
AOTVOKIR3DL4*02v2  : ----------------------------------------------------------------------------------------------- :     -
AOTVOKIR3DL4*03    : ----------------------------------------------------------------------------------------------- :     -
AOTVOKIR3DS4*04    : ----------------------------------------------------------------------------------------------- :     -
AOTVOKIR3DS4*04v1  : ----------------------------------------------------------------------------------------------- :     -
AOTVOKIR3DS5*01    : ----------------------------------------------------------------------------------------------- :     -
AOTVOKIR2DS5*01v1  : ----------------------------------------------------------------------------------------------- :     -
AOTVOKIR2DS5*01v2  : ----------------------------------------------------------------------------------------------- :     -
AOTVOKIR3DS7*01    : ----------------------------------------------------------------------------------------------- :     -
AOTVOKIR3DS7*01v1  : ----------------------------------------------------------------------------------------------- :     -
AOTVOKIR3DL8*01    : ----------------------------------------------------------------------------------------------- :     -
AOTVOKIR3DL8*01v1  : ----------------------------------------------------------------------------------------------- :     -
AOTVOKIR3DS8*01v2  : ----------------------------------------------------------------------------------------------- :     -
AOTVOKIR3DL8*02    : ----------------------------------------------------------------------------------------------- :     -
AOTVOKIR4DL9*01    : ----------------------------------------------------------------------------------------------- :     -
AOTVOKIR4DS9*01v1  : ----------------------------------------------------------------------------------------------- :     -
AOTVOKIR3DL9*01v2  : ----------------------------------------------------------------------------------------------- :     -
AOTVOKIR4DL9*02    : ----------------------------------------------------------------------------------------------- :     -
AOTVOKIR4DL10*01   : ----------------------------------------------------------------------------------------------- :     -
AOTVOKIR3DL10*01v1 : ----------------------------------------------------------------------------------------------- :     -
AOTVOKIR4DS10*01v2 : ----------------------------------------------------------------------------------------------- :     -
AOTVOKIR3DS10*01v3 : ----------------------------------------------------------------------------------------------- :     -
AOTVOKIR3DS10*01v4 : ----------------------------------------------------------------------------------------------- :     -

                      3140         *      3160         *      3180         *      3200         *      3220         *
BAC_clone_Om       : CGGGCTCTGAATGTGGAGGAAGGCCAGGAGCCATGAATGCAGGTGGCCTCTAGGGGCTGGAGAAGTCAAGGGAACTGATTCTCCCCCAAGTGTCC :  3230
AOTVOKIR3DL4*01    : ----------------------------------------------------------------------------------------------- :     -
AOTVOKIR3DL4*02    : ----------------------------------------------------------------------------------------------- :     -
AOTVOKIR3DL4*02v1  : ----------------------------------------------------------------------------------------------- :     -
AOTVOKIR3DL4*02v2  : ----------------------------------------------------------------------------------------------- :     -
AOTVOKIR3DL4*03    : ----------------------------------------------------------------------------------------------- :     -
AOTVOKIR3DS4*04    : ----------------------------------------------------------------------------------------------- :     -
```

```
AOTVOKIR3DS4*04v1  : ----------------------------------------------------------------------------------------------- :      -
AOTVOKIR3DS5*01    : ----------------------------------------------------------------------------------------------- :      -
AOTVOKIR2DS5*01v1  : ----------------------------------------------------------------------------------------------- :      -
AOTVOKIR2DS5*01v2  : ----------------------------------------------------------------------------------------------- :      -
AOTVOKIR3DS7*01    : ----------------------------------------------------------------------------------------------- :      -
AOTVOKIR3DS7*01v1  : ----------------------------------------------------------------------------------------------- :      -
AOTVOKIR3DL8*01    : ----------------------------------------------------------------------------------------------- :      -
AOTVOKIR3DL8*01v1  : ----------------------------------------------------------------------------------------------- :      -
AOTVOKIR3DS8*01v2  : ----------------------------------------------------------------------------------------------- :      -
AOTVOKIR3DL8*02    : ----------------------------------------------------------------------------------------------- :      -
AOTVOKIR4DL9*01    : ----------------------------------------------------------------------------------------------- :      -
AOTVOKIR4DS9*01v1  : ----------------------------------------------------------------------------------------------- :      -
AOTVOKIR3DL9*01v2  : ----------------------------------------------------------------------------------------------- :      -
AOTVOKIR4DL9*02    : ----------------------------------------------------------------------------------------------- :      -
AOTVOKIR4DL10*01   : ----------------------------------------------------------------------------------------------- :      -
AOTVOKIR3DL10*01v1 : ----------------------------------------------------------------------------------------------- :      -
AOTVOKIR4DS10*01v2 : ----------------------------------------------------------------------------------------------- :      -
AOTVOKIR3DS10*01v3 : ----------------------------------------------------------------------------------------------- :      -
AOTVOKIR3DS10*01v4 : ----------------------------------------------------------------------------------------------- :      -

                           3240         *       3260         *       3280         *       3300         *       3320
BAC_clone_Om       : AGAGGGGACGCAGCCCTGCATATGCCCTGATTTTAGCCCAGAGAGAACTGGGTCCGATTTCTGTCTCCAGAAGTGGAAGGGCTCAGTGTGTTCTC :   3325
AOTVOKIR3DL4*01    : ----------------------------------------------------------------------------------------------- :      -
AOTVOKIR3DL4*02    : ----------------------------------------------------------------------------------------------- :      -
AOTVOKIR3DL4*02v1  : ----------------------------------------------------------------------------------------------- :      -
AOTVOKIR3DL4*02v2  : ----------------------------------------------------------------------------------------------- :      -
AOTVOKIR3DL4*03    : ----------------------------------------------------------------------------------------------- :      -
AOTVOKIR3DS4*04    : ----------------------------------------------------------------------------------------------- :      -
AOTVOKIR3DS4*04v1  : ----------------------------------------------------------------------------------------------- :      -
AOTVOKIR3DS5*01    : ----------------------------------------------------------------------------------------------- :      -
AOTVOKIR2DS5*01v1  : ----------------------------------------------------------------------------------------------- :      -
AOTVOKIR2DS5*01v2  : ----------------------------------------------------------------------------------------------- :      -
AOTVOKIR3DS7*01    : ----------------------------------------------------------------------------------------------- :      -
AOTVOKIR3DS7*01v1  : ----------------------------------------------------------------------------------------------- :      -
AOTVOKIR3DL8*01    : ----------------------------------------------------------------------------------------------- :      -
AOTVOKIR3DL8*01v1  : ----------------------------------------------------------------------------------------------- :      -
AOTVOKIR3DS8*01v2  : ----------------------------------------------------------------------------------------------- :      -
AOTVOKIR3DL8*02    : ----------------------------------------------------------------------------------------------- :      -
AOTVOKIR4DL9*01    : ----------------------------------------------------------------------------------------------- :      -
AOTVOKIR4DS9*01v1  : ----------------------------------------------------------------------------------------------- :      -
AOTVOKIR3DL9*01v2  : ----------------------------------------------------------------------------------------------- :      -
AOTVOKIR4DL9*02    : ----------------------------------------------------------------------------------------------- :      -
AOTVOKIR4DL10*01   : ----------------------------------------------------------------------------------------------- :      -
AOTVOKIR3DL10*01v1 : ----------------------------------------------------------------------------------------------- :      -
AOTVOKIR4DS10*01v2 : ----------------------------------------------------------------------------------------------- :      -
```

```
AOTVOKIR3DS10*01v3 : ----------------------------------------------------------------------------------------------- :     -
AOTVOKIR3DS10*01v4 : ----------------------------------------------------------------------------------------------- :     -

                         *        3340          *        3360          *        3380          *        3400          *        3420
BAC_clone_Om       : TCCTGCCACCATATTGTGATAATTTTCTACAGCAGCAACAGGAAACAATACAGGAACCCAGGGCAAGGACAAGTCAAGAAACCACACCAGGCTAA :  3420
AOTVOKIR3DL4*01    : ----------------------------------------------------------------------------------------------- :     -
AOTVOKIR3DL4*02    : ----------------------------------------------------------------------------------------------- :     -
AOTVOKIR3DL4*02v1  : ----------------------------------------------------------------------------------------------- :     -
AOTVOKIR3DL4*02v2  : ----------------------------------------------------------------------------------------------- :     -
AOTVOKIR3DL4*03    : ----------------------------------------------------------------------------------------------- :     -
AOTVOKIR3DS4*04    : ----------------------------------------------------------------------------------------------- :     -
AOTVOKIR3DS4*04v1  : ----------------------------------------------------------------------------------------------- :     -
AOTVOKIR3DS5*01    : ----------------------------------------------------------------------------------------------- :     -
AOTVOKIR2DS5*01v1  : ----------------------------------------------------------------------------------------------- :     -
AOTVOKIR2DS5*01v2  : ----------------------------------------------------------------------------------------------- :     -
AOTVOKIR3DS7*01    : ----------------------------------------------------------------------------------------------- :     -
AOTVOKIR3DS7*01v1  : ----------------------------------------------------------------------------------------------- :     -
AOTVOKIR3DL8*01    : ----------------------------------------------------------------------------------------------- :     -
AOTVOKIR3DL8*01v1  : ----------------------------------------------------------------------------------------------- :     -
AOTVOKIR3DS8*01v2  : ----------------------------------------------------------------------------------------------- :     -
AOTVOKIR3DL8*02    : ----------------------------------------------------------------------------------------------- :     -
AOTVOKIR4DL9*01    : ----------------------------------------------------------------------------------------------- :     -
AOTVOKIR4DS9*01v1  : ----------------------------------------------------------------------------------------------- :     -
AOTVOKIR3DL9*01v2  : ----------------------------------------------------------------------------------------------- :     -
AOTVOKIR4DL9*02    : ----------------------------------------------------------------------------------------------- :     -
AOTVOKIR4DL10*01   : ----------------------------------------------------------------------------------------------- :     -
AOTVOKIR3DL10*01v1 : ----------------------------------------------------------------------------------------------- :     -
AOTVOKIR4DS10*01v2 : ----------------------------------------------------------------------------------------------- :     -
AOTVOKIR3DS10*01v3 : ----------------------------------------------------------------------------------------------- :     -
AOTVOKIR3DS10*01v4 : ----------------------------------------------------------------------------------------------- :     -

                              *        3440          *        3460          *        3480          *        3500          *
BAC_clone_Om       : GGGTGGCCACACTGACATCAGCAAGGGGGGGATGCTGAGGCCACCATCAGGCTCGATCCACAGAGGGAGGGGTTGATGCTCCTGGAACCAGCACC :  3515
AOTVOKIR3DL4*01    : ----------------------------------------------------------------------------------------------- :     -
AOTVOKIR3DL4*02    : ----------------------------------------------------------------------------------------------- :     -
AOTVOKIR3DL4*02v1  : ----------------------------------------------------------------------------------------------- :     -
AOTVOKIR3DL4*02v2  : ----------------------------------------------------------------------------------------------- :     -
AOTVOKIR3DL4*03    : ----------------------------------------------------------------------------------------------- :     -
AOTVOKIR3DS4*04    : ----------------------------------------------------------------------------------------------- :     -
AOTVOKIR3DS4*04v1  : ----------------------------------------------------------------------------------------------- :     -
AOTVOKIR3DS5*01    : ----------------------------------------------------------------------------------------------- :     -
AOTVOKIR2DS5*01v1  : ----------------------------------------------------------------------------------------------- :     -
AOTVOKIR2DS5*01v2  : ----------------------------------------------------------------------------------------------- :     -
AOTVOKIR3DS7*01    : ----------------------------------------------------------------------------------------------- :     -
```

```
AOTVOKIR3DS7*01v1  : ----------------------------------------------------------------------------------------------- :    -
AOTVOKIR3DL8*01    : ----------------------------------------------------------------------------------------------- :    -
AOTVOKIR3DL8*01v1  : ----------------------------------------------------------------------------------------------- :    -
AOTVOKIR3DS8*01v2  : ----------------------------------------------------------------------------------------------- :    -
AOTVOKIR3DL8*02    : ----------------------------------------------------------------------------------------------- :    -
AOTVOKIR4DL9*01    : ----------------------------------------------------------------------------------------------- :    -
AOTVOKIR4DS9*01v1  : ----------------------------------------------------------------------------------------------- :    -
AOTVOKIR3DL9*01v2  : ----------------------------------------------------------------------------------------------- :    -
AOTVOKIR4DL9*02    : ----------------------------------------------------------------------------------------------- :    -
AOTVOKIR4DL10*01   : ----------------------------------------------------------------------------------------------- :    -
AOTVOKIR3DL10*01v1 : ----------------------------------------------------------------------------------------------- :    -
AOTVOKIR4DS10*01v2 : ----------------------------------------------------------------------------------------------- :    -
AOTVOKIR3DS10*01v3 : ----------------------------------------------------------------------------------------------- :    -
AOTVOKIR3DS10*01v4 : ----------------------------------------------------------------------------------------------- :    -


                         3520         *       3540         *       3560         *       3580         *       3600         *
BAC_clone_Om       : AGGGGCTGCCCTATGGAAGCTGGGACCATGGAGAAGCACAGACATGGCAGGAGAGGCTCCCAGTCCCCACCAGGAACAGGGTGTGTGGACACTGC :  3610
AOTVOKIR3DL4*01    : ----------------------------------------------------------------------------------------------- :    -
AOTVOKIR3DL4*02    : ----------------------------------------------------------------------------------------------- :    -
AOTVOKIR3DL4*02v1  : ----------------------------------------------------------------------------------------------- :    -
AOTVOKIR3DL4*02v2  : ----------------------------------------------------------------------------------------------- :    -
AOTVOKIR3DL4*03    : ----------------------------------------------------------------------------------------------- :    -
AOTVOKIR3DS4*04    : ----------------------------------------------------------------------------------------------- :    -
AOTVOKIR3DS4*04v1  : ----------------------------------------------------------------------------------------------- :    -
AOTVOKIR3DS5*01    : ----------------------------------------------------------------------------------------------- :    -
AOTVOKIR2DS5*01v1  : ----------------------------------------------------------------------------------------------- :    -
AOTVOKIR2DS5*01v2  : ----------------------------------------------------------------------------------------------- :    -
AOTVOKIR3DS7*01    : ----------------------------------------------------------------------------------------------- :    -
AOTVOKIR3DS7*01v1  : ----------------------------------------------------------------------------------------------- :    -
AOTVOKIR3DL8*01    : ----------------------------------------------------------------------------------------------- :    -
AOTVOKIR3DL8*01v1  : ----------------------------------------------------------------------------------------------- :    -
AOTVOKIR3DS8*01v2  : ----------------------------------------------------------------------------------------------- :    -
AOTVOKIR3DL8*02    : ----------------------------------------------------------------------------------------------- :    -
AOTVOKIR4DL9*01    : ----------------------------------------------------------------------------------------------- :    -
AOTVOKIR4DS9*01v1  : ----------------------------------------------------------------------------------------------- :    -
AOTVOKIR3DL9*01v2  : ----------------------------------------------------------------------------------------------- :    -
AOTVOKIR4DL9*02    : ----------------------------------------------------------------------------------------------- :    -
AOTVOKIR4DL10*01   : ----------------------------------------------------------------------------------------------- :    -
AOTVOKIR3DL10*01v1 : ----------------------------------------------------------------------------------------------- :    -
AOTVOKIR4DS10*01v2 : ----------------------------------------------------------------------------------------------- :    -
AOTVOKIR3DS10*01v3 : ----------------------------------------------------------------------------------------------- :    -
AOTVOKIR3DS10*01v4 : ----------------------------------------------------------------------------------------------- :    -


                              3620         *       3640         *       3660         *       3680         *       3700
```

```
BAC_clone_Om        : TGCCCGCCTTACTCATCAGTTCATACCTCCTGCCAGGGATTCCAATTTGTCCTAAACAGATTGAACCAGGCTGTTCAGATCCTGGACGTGCAGCC :  3705
AOTVOKIR3DL4*01     : ----------------------------------------------------------------------------------------------- :     -
AOTVOKIR3DL4*02     : ----------------------------------------------------------------------------------------------- :     -
AOTVOKIR3DL4*02v1   : ----------------------------------------------------------------------------------------------- :     -
AOTVOKIR3DL4*02v2   : ----------------------------------------------------------------------------------------------- :     -
AOTVOKIR3DL4*03     : ----------------------------------------------------------------------------------------------- :     -
AOTVOKIR3DS4*04     : ----------------------------------------------------------------------------------------------- :     -
AOTVOKIR3DS4*04v1   : ----------------------------------------------------------------------------------------------- :     -
AOTVOKIR3DS5*01     : ----------------------------------------------------------------------------------------------- :     -
AOTVOKIR2DS5*01v1   : ----------------------------------------------------------------------------------------------- :     -
AOTVOKIR2DS5*01v2   : ----------------------------------------------------------------------------------------------- :     -
AOTVOKIR3DS7*01     : ----------------------------------------------------------------------------------------------- :     -
AOTVOKIR3DS7*01v1   : ----------------------------------------------------------------------------------------------- :     -
AOTVOKIR3DL8*01     : ----------------------------------------------------------------------------------------------- :     -
AOTVOKIR3DL8*01v1   : ----------------------------------------------------------------------------------------------- :     -
AOTVOKIR3DS8*01v2   : ----------------------------------------------------------------------------------------------- :     -
AOTVOKIR3DL8*02     : ----------------------------------------------------------------------------------------------- :     -
AOTVOKIR4DL9*01     : ----------------------------------------------------------------------------------------------- :     -
AOTVOKIR4DS9*01v1   : ----------------------------------------------------------------------------------------------- :     -
AOTVOKIR3DL9*01v2   : ----------------------------------------------------------------------------------------------- :     -
AOTVOKIR4DL9*02     : ----------------------------------------------------------------------------------------------- :     -
AOTVOKIR4DL10*01    : ----------------------------------------------------------------------------------------------- :     -
AOTVOKIR3DL10*01v1  : ----------------------------------------------------------------------------------------------- :     -
AOTVOKIR4DS10*01v2  : ----------------------------------------------------------------------------------------------- :     -
AOTVOKIR3DS10*01v3  : ----------------------------------------------------------------------------------------------- :     -
AOTVOKIR3DS10*01v4  : ----------------------------------------------------------------------------------------------- :     -

                          *      3720          *       3740          *       3760          *       3780          *       3800
BAC_clone_Om        : TGTCGTGGCTCCTCTTCCACCGTCACATGGACAGGAAGAAACAGATTAGTGGGAAACAGAAACAGCTCAAGGGATGAGGCTGAGCCCAGTGGGAA :  3800
AOTVOKIR3DL4*01     : ----------------------------------------------------------------------------------------------- :     -
AOTVOKIR3DL4*02     : ----------------------------------------------------------------------------------------------- :     -
AOTVOKIR3DL4*02v1   : ----------------------------------------------------------------------------------------------- :     -
AOTVOKIR3DL4*02v2   : ----------------------------------------------------------------------------------------------- :     -
AOTVOKIR3DL4*03     : ----------------------------------------------------------------------------------------------- :     -
AOTVOKIR3DS4*04     : ----------------------------------------------------------------------------------------------- :     -
AOTVOKIR3DS4*04v1   : ----------------------------------------------------------------------------------------------- :     -
AOTVOKIR3DS5*01     : ----------------------------------------------------------------------------------------------- :     -
AOTVOKIR2DS5*01v1   : ----------------------------------------------------------------------------------------------- :     -
AOTVOKIR2DS5*01v2   : ----------------------------------------------------------------------------------------------- :     -
AOTVOKIR3DS7*01     : ----------------------------------------------------------------------------------------------- :     -
AOTVOKIR3DS7*01v1   : ----------------------------------------------------------------------------------------------- :     -
AOTVOKIR3DL8*01     : ----------------------------------------------------------------------------------------------- :     -
AOTVOKIR3DL8*01v1   : ----------------------------------------------------------------------------------------------- :     -
AOTVOKIR3DS8*01v2   : ----------------------------------------------------------------------------------------------- :     -
AOTVOKIR3DL8*02     : ----------------------------------------------------------------------------------------------- :     -
```

```
AOTVOKIR4DL9*01     : ----------------------------------------------------------------------------------------------- :     -
AOTVOKIR4DS9*01v1   : ----------------------------------------------------------------------------------------------- :     -
AOTVOKIR3DL9*01v2   : ----------------------------------------------------------------------------------------------- :     -
AOTVOKIR4DL9*02     : ----------------------------------------------------------------------------------------------- :     -
AOTVOKIR4DL10*01    : ----------------------------------------------------------------------------------------------- :     -
AOTVOKIR3DL10*01v1  : ----------------------------------------------------------------------------------------------- :     -
AOTVOKIR4DS10*01v2  : ----------------------------------------------------------------------------------------------- :     -
AOTVOKIR3DS10*01v3  : ----------------------------------------------------------------------------------------------- :     -
AOTVOKIR3DS10*01v4  : ----------------------------------------------------------------------------------------------- :     -

                               *       3820         *       3840         *       3860         *       3880         *
BAC_clone_Om        : GGGAGTCAGGGGCTGCTAGAGACAGAGACAGAGAAGAGGGAGGGGGACAGAGGGAGGGACCTGCACCAGGGGTGACGGGCACAGAAAGGAGCACG :  3895
AOTVOKIR3DL4*01     : ----------------------------------------------------------------------------------------------- :     -
AOTVOKIR3DL4*02     : ----------------------------------------------------------------------------------------------- :     -
AOTVOKIR3DL4*02v1   : ----------------------------------------------------------------------------------------------- :     -
AOTVOKIR3DL4*02v2   : ----------------------------------------------------------------------------------------------- :     -
AOTVOKIR3DL4*03     : ----------------------------------------------------------------------------------------------- :     -
AOTVOKIR3DS4*04     : ----------------------------------------------------------------------------------------------- :     -
AOTVOKIR3DS4*04v1   : ----------------------------------------------------------------------------------------------- :     -
AOTVOKIR3DS5*01     : ----------------------------------------------------------------------------------------------- :     -
AOTVOKIR2DS5*01v1   : ----------------------------------------------------------------------------------------------- :     -
AOTVOKIR2DS5*01v2   : ----------------------------------------------------------------------------------------------- :     -
AOTVOKIR3DS7*01     : ----------------------------------------------------------------------------------------------- :     -
AOTVOKIR3DS7*01v1   : ----------------------------------------------------------------------------------------------- :     -
AOTVOKIR3DL8*01     : ----------------------------------------------------------------------------------------------- :     -
AOTVOKIR3DL8*01v1   : ----------------------------------------------------------------------------------------------- :     -
AOTVOKIR3DS8*01v2   : ----------------------------------------------------------------------------------------------- :     -
AOTVOKIR3DL8*02     : ----------------------------------------------------------------------------------------------- :     -
AOTVOKIR4DL9*01     : ----------------------------------------------------------------------------------------------- :     -
AOTVOKIR4DS9*01v1   : ----------------------------------------------------------------------------------------------- :     -
AOTVOKIR3DL9*01v2   : ----------------------------------------------------------------------------------------------- :     -
AOTVOKIR4DL9*02     : ----------------------------------------------------------------------------------------------- :     -
AOTVOKIR4DL10*01    : ----------------------------------------------------------------------------------------------- :     -
AOTVOKIR3DL10*01v1  : ----------------------------------------------------------------------------------------------- :     -
AOTVOKIR4DS10*01v2  : ----------------------------------------------------------------------------------------------- :     -
AOTVOKIR3DS10*01v3  : ----------------------------------------------------------------------------------------------- :     -
AOTVOKIR3DS10*01v4  : ----------------------------------------------------------------------------------------------- :     -

                         3900         *       3920         *       3940         *       3960         *       3980         *
BAC_clone_Om        : GAGACACAGAGAGGGAGGAGAGAGTCAGACACCGGGGAGGGGAATCCTCACTCATTCCAGGTCCCATGGATGGGATGAGAAAGGGAGATGCCTTC :  3990
AOTVOKIR3DL4*01     : ----------------------------------------------------------------------------------------------- :     -
AOTVOKIR3DL4*02     : ----------------------------------------------------------------------------------------------- :     -
AOTVOKIR3DL4*02v1   : ----------------------------------------------------------------------------------------------- :     -
AOTVOKIR3DL4*02v2   : ----------------------------------------------------------------------------------------------- :     -
```

```
AOTVOKIR3DL4*03     : ----------------------------------------------------------------------------------------------- :     -
AOTVOKIR3DS4*04     : ----------------------------------------------------------------------------------------------- :     -
AOTVOKIR3DS4*04v1   : ----------------------------------------------------------------------------------------------- :     -
AOTVOKIR3DS5*01     : ----------------------------------------------------------------------------------------------- :     -
AOTVOKIR2DS5*01v1   : ----------------------------------------------------------------------------------------------- :     -
AOTVOKIR2DS5*01v2   : ----------------------------------------------------------------------------------------------- :     -
AOTVOKIR3DS7*01     : ----------------------------------------------------------------------------------------------- :     -
AOTVOKIR3DS7*01v1   : ----------------------------------------------------------------------------------------------- :     -
AOTVOKIR3DL8*01     : ----------------------------------------------------------------------------------------------- :     -
AOTVOKIR3DL8*01v1   : ----------------------------------------------------------------------------------------------- :     -
AOTVOKIR3DS8*01v2   : ----------------------------------------------------------------------------------------------- :     -
AOTVOKIR3DL8*02     : ----------------------------------------------------------------------------------------------- :     -
AOTVOKIR4DL9*01     : ----------------------------------------------------------------------------------------------- :     -
AOTVOKIR4DS9*01v1   : ----------------------------------------------------------------------------------------------- :     -
AOTVOKIR3DL9*01v2   : ----------------------------------------------------------------------------------------------- :     -
AOTVOKIR4DL9*02     : ----------------------------------------------------------------------------------------------- :     -
AOTVOKIR4DL10*01    : ----------------------------------------------------------------------------------------------- :     -
AOTVOKIR3DL10*01v1  : ----------------------------------------------------------------------------------------------- :     -
AOTVOKIR4DS10*01v2  : ----------------------------------------------------------------------------------------------- :     -
AOTVOKIR3DS10*01v3  : ----------------------------------------------------------------------------------------------- :     -
AOTVOKIR3DS10*01v4  : ----------------------------------------------------------------------------------------------- :     -

                               4000         *       4020         *       4040         *       4060         *       4080
BAC_clone_Om        : TAAACTCACAATCTCGTTTCTTAGGATTCCACAGAAAACCTTCCCTCCTGGCCCTCCCAGCTCCCTTGGTGAGATCAGGAGAGACGGTCATCCTG :  4085
AOTVOKIR3DL4*01     : ----------------------------------------------------------------------------------------------- :     -
AOTVOKIR3DL4*02     : ----------------------------------------------------------------------------------------------- :     -
AOTVOKIR3DL4*02v1   : ----------------------------------------------------------------------------------------------- :     -
AOTVOKIR3DL4*02v2   : ----------------------------------------------------------------------------------------------- :     -
AOTVOKIR3DL4*03     : ----------------------------------------------------------------------------------------------- :     -
AOTVOKIR3DS4*04     : ----------------------------------------------------------------------------------------------- :     -
AOTVOKIR3DS4*04v1   : ----------------------------------------------------------------------------------------------- :     -
AOTVOKIR3DS5*01     : ----------------------------------------------------------------------------------------------- :     -
AOTVOKIR2DS5*01v1   : ----------------------------------------------------------------------------------------------- :     -
AOTVOKIR2DS5*01v2   : ----------------------------------------------------------------------------------------------- :     -
AOTVOKIR3DS7*01     : ----------------------------------------------------------------------------------------------- :     -
AOTVOKIR3DS7*01v1   : ----------------------------------------------------------------------------------------------- :     -
AOTVOKIR3DL8*01     : ----------------------------------------------------------------------------------------------- :     -
AOTVOKIR3DL8*01v1   : ----------------------------------------------------------------------------------------------- :     -
AOTVOKIR3DS8*01v2   : ----------------------------------------------------------------------------------------------- :     -
AOTVOKIR3DL8*02     : ----------------------------------------------------------------------------------------------- :     -
AOTVOKIR4DL9*01     : ----------------------------------------------------------------------------------------------- :     -
AOTVOKIR4DS9*01v1   : ----------------------------------------------------------------------------------------------- :     -
AOTVOKIR3DL9*01v2   : ----------------------------------------------------------------------------------------------- :     -
AOTVOKIR4DL9*02     : ----------------------------------------------------------------------------------------------- :     -
AOTVOKIR4DL10*01    : ----------------------------------------------------------------------------------------------- :     -
```

```
AOTVOKIR3DL10*01v1 : ----------------------------------------------------------------------------------------------- :     -
AOTVOKIR4DS10*01v2 : ----------------------------------------------------------------------------------------------- :     -
AOTVOKIR3DS10*01v3 : ----------------------------------------------------------------------------------------------- :     -
AOTVOKIR3DS10*01v4 : ----------------------------------------------------------------------------------------------- :     -

                         *      4100         *      4120         *      4140         *      4160         *      4180
BAC_clone_Om       : CAGTGTTGGTCAGATATCATGTCTGAGCACTTCCTTCTGCACAGAAAGGAGCTCACAAGGACATGCCCCCACCCCGTGTCTACCTTGTGTGTTTT :  4180
AOTVOKIR3DL4*01    : ----------------------------------------------------------------------------------------------- :     -
AOTVOKIR3DL4*02    : ----------------------------------------------------------------------------------------------- :     -
AOTVOKIR3DL4*02v1  : ----------------------------------------------------------------------------------------------- :     -
AOTVOKIR3DL4*02v2  : ----------------------------------------------------------------------------------------------- :     -
AOTVOKIR3DL4*03    : ----------------------------------------------------------------------------------------------- :     -
AOTVOKIR3DS4*04    : ----------------------------------------------------------------------------------------------- :     -
AOTVOKIR3DS4*04v1  : ----------------------------------------------------------------------------------------------- :     -
AOTVOKIR3DS5*01    : ----------------------------------------------------------------------------------------------- :     -
AOTVOKIR2DS5*01v1  : ----------------------------------------------------------------------------------------------- :     -
AOTVOKIR2DS5*01v2  : ----------------------------------------------------------------------------------------------- :     -
AOTVOKIR3DS7*01    : ----------------------------------------------------------------------------------------------- :     -
AOTVOKIR3DS7*01v1  : ----------------------------------------------------------------------------------------------- :     -
AOTVOKIR3DL8*01    : ----------------------------------------------------------------------------------------------- :     -
AOTVOKIR3DL8*01v1  : ----------------------------------------------------------------------------------------------- :     -
AOTVOKIR3DS8*01v2  : ----------------------------------------------------------------------------------------------- :     -
AOTVOKIR3DL8*02    : ----------------------------------------------------------------------------------------------- :     -
AOTVOKIR4DL9*01    : ----------------------------------------------------------------------------------------------- :     -
AOTVOKIR4DS9*01v1  : ----------------------------------------------------------------------------------------------- :     -
AOTVOKIR3DL9*01v2  : ----------------------------------------------------------------------------------------------- :     -
AOTVOKIR4DL9*02    : ----------------------------------------------------------------------------------------------- :     -
AOTVOKIR4DL10*01   : ----------------------------------------------------------------------------------------------- :     -
AOTVOKIR3DL10*01v1 : ----------------------------------------------------------------------------------------------- :     -
AOTVOKIR4DS10*01v2 : ----------------------------------------------------------------------------------------------- :     -
AOTVOKIR3DS10*01v3 : ----------------------------------------------------------------------------------------------- :     -
AOTVOKIR3DS10*01v4 : ----------------------------------------------------------------------------------------------- :     -

                              *      4200         *      4220         *      4240         *      4260         *
BAC_clone_Om       : TATGAAAGCAATTTTACCGTATTAAAATCTAGTAGGAGTCGCTCATTCAGCACTTACTCAAAGTTCTCAGCTGGCACGTTTGTTGTAGGGAGATG :  4275
AOTVOKIR3DL4*01    : ----------------------------------------------------------------------------------------------- :     -
AOTVOKIR3DL4*02    : ----------------------------------------------------------------------------------------------- :     -
AOTVOKIR3DL4*02v1  : ----------------------------------------------------------------------------------------------- :     -
AOTVOKIR3DL4*02v2  : ----------------------------------------------------------------------------------------------- :     -
AOTVOKIR3DL4*03    : ----------------------------------------------------------------------------------------------- :     -
AOTVOKIR3DS4*04    : ----------------------------------------------------------------------------------------------- :     -
AOTVOKIR3DS4*04v1  : ----------------------------------------------------------------------------------------------- :     -
AOTVOKIR3DS5*01    : ----------------------------------------------------------------------------------------------- :     -
AOTVOKIR2DS5*01v1  : ----------------------------------------------------------------------------------------------- :     -
```

```
AOTVOKIR2DS5*01v2  : ----------------------------------------------------------------------------------------------- :     -
AOTVOKIR3DS7*01    : ----------------------------------------------------------------------------------------------- :     -
AOTVOKIR3DS7*01v1  : ----------------------------------------------------------------------------------------------- :     -
AOTVOKIR3DL8*01    : ----------------------------------------------------------------------------------------------- :     -
AOTVOKIR3DL8*01v1  : ----------------------------------------------------------------------------------------------- :     -
AOTVOKIR3DS8*01v2  : ----------------------------------------------------------------------------------------------- :     -
AOTVOKIR3DL8*02    : ----------------------------------------------------------------------------------------------- :     -
AOTVOKIR4DL9*01    : ----------------------------------------------------------------------------------------------- :     -
AOTVOKIR4DS9*01v1  : ----------------------------------------------------------------------------------------------- :     -
AOTVOKIR3DL9*01v2  : ----------------------------------------------------------------------------------------------- :     -
AOTVOKIR4DL9*02    : ----------------------------------------------------------------------------------------------- :     -
AOTVOKIR4DL10*01   : ----------------------------------------------------------------------------------------------- :     -
AOTVOKIR3DL10*01v1 : ----------------------------------------------------------------------------------------------- :     -
AOTVOKIR4DS10*01v2 : ----------------------------------------------------------------------------------------------- :     -
AOTVOKIR3DS10*01v3 : ----------------------------------------------------------------------------------------------- :     -
AOTVOKIR3DS10*01v4 : ----------------------------------------------------------------------------------------------- :     -

                         4280         *       4300         *       4320         *       4340         *       4360         *
BAC_clone_Om       : CCATGTCTGTGCAGGATGGGTCCTTCCTGTAGCTCTGGGCACCCAGGTGTGGTAGGAGCCTTAGAAACATGGAAAAAGGGAGAATCTTCTGAGCA :  4370
AOTVOKIR3DL4*01    : ----------------------------------------------------------------------------------------------- :     -
AOTVOKIR3DL4*02    : ----------------------------------------------------------------------------------------------- :     -
AOTVOKIR3DL4*02v1  : ----------------------------------------------------------------------------------------------- :     -
AOTVOKIR3DL4*02v2  : ----------------------------------------------------------------------------------------------- :     -
AOTVOKIR3DL4*03    : ----------------------------------------------------------------------------------------------- :     -
AOTVOKIR3DS4*04    : ----------------------------------------------------------------------------------------------- :     -
AOTVOKIR3DS4*04v1  : ----------------------------------------------------------------------------------------------- :     -
AOTVOKIR3DS5*01    : ----------------------------------------------------------------------------------------------- :     -
AOTVOKIR2DS5*01v1  : ----------------------------------------------------------------------------------------------- :     -
AOTVOKIR2DS5*01v2  : ----------------------------------------------------------------------------------------------- :     -
AOTVOKIR3DS7*01    : ----------------------------------------------------------------------------------------------- :     -
AOTVOKIR3DS7*01v1  : ----------------------------------------------------------------------------------------------- :     -
AOTVOKIR3DL8*01    : ----------------------------------------------------------------------------------------------- :     -
AOTVOKIR3DL8*01v1  : ----------------------------------------------------------------------------------------------- :     -
AOTVOKIR3DS8*01v2  : ----------------------------------------------------------------------------------------------- :     -
AOTVOKIR3DL8*02    : ----------------------------------------------------------------------------------------------- :     -
AOTVOKIR4DL9*01    : ----------------------------------------------------------------------------------------------- :     -
AOTVOKIR4DS9*01v1  : ----------------------------------------------------------------------------------------------- :     -
AOTVOKIR3DL9*01v2  : ----------------------------------------------------------------------------------------------- :     -
AOTVOKIR4DL9*02    : ----------------------------------------------------------------------------------------------- :     -
AOTVOKIR4DL10*01   : ----------------------------------------------------------------------------------------------- :     -
AOTVOKIR3DL10*01v1 : ----------------------------------------------------------------------------------------------- :     -
AOTVOKIR4DS10*01v2 : ----------------------------------------------------------------------------------------------- :     -
AOTVOKIR3DS10*01v3 : ----------------------------------------------------------------------------------------------- :     -
AOTVOKIR3DS10*01v4 : ----------------------------------------------------------------------------------------------- :     -
```

```
                                                                                                EXON 3b
                              4380         *        4400         *        4420         *        4440         *        4460
BAC_clone_Om        : CAGGGAGGGAGGGGCGGCTCCACATCTTCCTCTCTAAGGCAACGCCTCCTCCTCCCCCAGGTGCTCAGGACCAGCCCTTCCTGTCTGCCTGGCCC :  4465
AOTVOKIR3DL4*01     : ----------------------------------------------------------------------------------------------- :     -
AOTVOKIR3DL4*02     : ----------------------------------------------------------------------------------------------- :     -
AOTVOKIR3DL4*02v1   : ----------------------------------------------------------------------------------------------- :     -
AOTVOKIR3DL4*02v2   : ----------------------------------------------------------------------------------------------- :     -
AOTVOKIR3DL4*03     : ----------------------------------------------------------------------------------------------- :     -
AOTVOKIR3DS4*04     : ----------------------------------------------------------------------------------------------- :     -
AOTVOKIR3DS4*04v1   : ----------------------------------------------------------------------------------------------- :     -
AOTVOKIR3DS5*01     : ----------------------------------------------------------------------------------------------- :     -
AOTVOKIR2DS5*01v1   : ----------------------------------------------------------------------------------------------- :     -
AOTVOKIR2DS5*01v2   : ----------------------------------------------------------------------------------------------- :     -
AOTVOKIR3DS7*01     : ----------------------------------------------------------------------------------------------- :     -
AOTVOKIR3DS7*01v1   : ----------------------------------------------------------------------------------------------- :     -
AOTVOKIR3DL8*01     : ----------------------------------------------------------------------------------------------- :     -
AOTVOKIR3DL8*01v1   : ----------------------------------------------------------------------------------------------- :     -
AOTVOKIR3DS8*01v2   : ----------------------------------------------------------------------------------------------- :     -
AOTVOKIR3DL8*02     : ----------------------------------------------------------------------------------------------- :     -
AOTVOKIR4DL9*01     : ------------------------------------------------------------GTGGTCAGGACAAGCCCTTCCTGTCTGCCTGGCCC :   387
AOTVOKIR4DS9*01v1   : ------------------------------------------------------------GTGGTCAGGACAAGCCCTTCCTGTCTGCCTGGCCC :   387
AOTVOKIR3DL9*01v2   : ------------------------------------------------------------GTGGTCAGGACAAGCCCTTCCTGTCTGCCTGGCCC :   189
AOTVOKIR4DL9*02     : ------------------------------------------------------------GTGGTCAGGACAAGCCCTTCCTGTCTGCCTGGCCC :   387
AOTVOKIR4DL10*01    : ------------------------------------------------------------GTGGTCAGGACAAGCCCTTCCTGTCTGCCTGGCCC :   387
AOTVOKIR3DL10*01v1  : ------------------------------------------------------------GTGGTCAGGACAAGCCCTTCCTGTCTGCCTGGCCC :   186
AOTVOKIR4DS10*01v2  : ------------------------------------------------------------GTGGTCAGGACAAGCCCTTCCTGTCTGCCTGGCCC :   387
AOTVOKIR3DS10*01v3  : ----------------------------------------------------------------------------------------------- :     -
AOTVOKIR3DS10*01v4  : ----------------------------------------------------------------------------------------------- :     -

                          *        4480         *        4500         *        4520         *        4540         *        4560
BAC_clone_Om        : AGCGCTGTGGTGTCTCCAGGAGGACACGTGACTCTTCGGTGTCACTATGGTCGTGGGTTTAACAACTTCACCCTGTACAAAGAAGACAGATTCCA :  4560
AOTVOKIR3DL4*01     : ----------------------------------------------------------------------------------------------- :     -
AOTVOKIR3DL4*02     : ----------------------------------------------------------------------------------------------- :     -
AOTVOKIR3DL4*02v1   : ----------------------------------------------------------------------------------------------- :     -
AOTVOKIR3DL4*02v2   : ----------------------------------------------------------------------------------------------- :     -
AOTVOKIR3DL4*03     : ----------------------------------------------------------------------------------------------- :     -
AOTVOKIR3DS4*04     : ----------------------------------------------------------------------------------------------- :     -
AOTVOKIR3DS4*04v1   : ----------------------------------------------------------------------------------------------- :     -
AOTVOKIR3DS5*01     : ----------------------------------------------------------------------------------------------- :     -
AOTVOKIR2DS5*01v1   : ----------------------------------------------------------------------------------------------- :     -
AOTVOKIR2DS5*01v2   : ----------------------------------------------------------------------------------------------- :     -
AOTVOKIR3DS7*01     : ----------------------------------------------------------------------------------------------- :     -
AOTVOKIR3DS7*01v1   : ----------------------------------------------------------------------------------------------- :     -
AOTVOKIR3DL8*01     : ----------------------------------------------------------------------------------------------- :     -
AOTVOKIR3DL8*01v1   : ----------------------------------------------------------------------------------------------- :     -
```

```
AOTVOKIR3DS8*01v2   : ----------------------------------------------------------------------------------------------- :     -
AOTVOKIR3DL8*02     : ----------------------------------------------------------------------------------------------- :     -
AOTVOKIR4DL9*01     : AGCGCTGTGGTGCCTCGAGGTGGAAACGTGACTCTTCGGTGTGACTATGGTGGTGGGTTTAACAACTTCACCCTGTACAAAGAAGACAGATTCGA :   482
AOTVOKIR4DS9*01v1   : AGCGCTGTGGTGCCTCGAGGTGGAAACGTGACTCTTCGGTGTGACTATGGTGGTGGGTTTAACAACTTCACCCTGTACAAAGAAGACAGATTCGA :   482
AOTVOKIR3DL9*01v2   : AGCGCTGTGGTGCCTCGAGGTGGAAACGTGACTCTTCGGTGTGACTATGGTGGTGGGTTTAACAACTTCACCCTGTACAAAGAAGACAGATTCGA :   284
AOTVOKIR4DL9*02     : AGCGCTGTGGTGCCTCGAGGTGGAAACGTGACTCTTCGGTGTGACTATGGTGGTGGGTTTAACAACTTCACCCTGTACAAAGAAGACAGATTCGA :   482
AOTVOKIR4DL10*01    : AGCGCTGTGGTGCCTCGAGGTGGAAATGTGACTCTTCGGTGTGACTATGGTGGTGGGTTTAACAACTTCACCCTGTATAAAGAAGACAGATTCGA :   482
AOTVOKIR3DL10*01v1  : AGCGCTGTGGTGCCTCGAGGTGGAAATGTGACTCTTCGGTGTGACTATGGTGGTGGGTTTAACAACTTCACCCTGTATAAAGAAGACAGATTCGA :   281
AOTVOKIR4DS10*01v2  : AGCGCTGTGGTGCCTCGAGGTGGAAATGTGACTCTTCGGTGTGACTATGGTGGTGGGTTTAACAACTTCACCCTGTATAAAGAAGACAGATTCGA :   482
AOTVOKIR3DS10*01v3  : ----------------------------------------------------------------------------------------------- :     -
AOTVOKIR3DS10*01v4  : ----------------------------------------------------------------------------------------------- :     -

                               *       4580         *       4600         *       4620         *       4640         *
BAC_clone_Om        : CGTTCCTATCCTCAACGGCGACATATTCCAGGAGAGCTTCCTCATGGGCCCCGTGACTGCAGAACACGCAGGGACCTACAGATGTCGGGGTTTTC :  4655
AOTVOKIR3DL4*01     : ----------------------------------------------------------------------------------------------- :     -
AOTVOKIR3DL4*02     : ----------------------------------------------------------------------------------------------- :     -
AOTVOKIR3DL4*02v1   : ----------------------------------------------------------------------------------------------- :     -
AOTVOKIR3DL4*02v2   : ----------------------------------------------------------------------------------------------- :     -
AOTVOKIR3DL4*03     : ----------------------------------------------------------------------------------------------- :     -
AOTVOKIR3DS4*04     : ----------------------------------------------------------------------------------------------- :     -
AOTVOKIR3DS4*04v1   : ----------------------------------------------------------------------------------------------- :     -
AOTVOKIR3DS5*01     : ----------------------------------------------------------------------------------------------- :     -
AOTVOKIR2DS5*01v1   : ----------------------------------------------------------------------------------------------- :     -
AOTVOKIR2DS5*01v2   : ----------------------------------------------------------------------------------------------- :     -
AOTVOKIR3DS7*01     : ----------------------------------------------------------------------------------------------- :     -
AOTVOKIR3DS7*01v1   : ----------------------------------------------------------------------------------------------- :     -
AOTVOKIR3DL8*01     : ----------------------------------------------------------------------------------------------- :     -
AOTVOKIR3DL8*01v1   : ----------------------------------------------------------------------------------------------- :     -
AOTVOKIR3DS8*01v2   : ----------------------------------------------------------------------------------------------- :     -
AOTVOKIR3DL8*02     : ----------------------------------------------------------------------------------------------- :     -
AOTVOKIR4DL9*01     : CGTACCCATCCTCAATGGCGTTATATTCCAGGAGAGCTTCTTGATGGGCCCCGTGACTGCAGCACACGCAGGGACCTACAGATGTCGGCGTTTTC :   577
AOTVOKIR4DS9*01v1   : CGTACCCATCCTCAATGGCGTTATATTCCAGGAGAGCTTCTTGATGGGCCCCGTGACTGCAGCACACGCAGGGACCTACAGATGTCGGCGTTTTC :   577
AOTVOKIR3DL9*01v2   : CGTACCCATCCTCAATGGCGTTATATTCCAGGAGAGCTTCTTGATGGGCCCCGTGACTGCAGCACACGCAGGGACCTACAGATGTCGGCGTTTTC :   379
AOTVOKIR4DL9*02     : CGTACCCATCCTCAATGGCGTTATATTCCAGGAGAGCTTCTTGATGGGCCCCGTGACTGCAGCACACGCAGGGACCTACAGATGTCGGCGTTTTC :   577
AOTVOKIR4DL10*01    : CGTACCCATCCTCAATGGCGTTATATTCCAGGAGAGCTTCTTGATGGGCCCCGTGACTGCAGCACACGCAGGGACCTACAGATGTCGGCGTTTTC :   577
AOTVOKIR3DL10*01v1  : CGTACCCATCCTCAATGGCGTTATATTCCAGGAGAGCTTCTTGATGGGCCCCGTGACTGCAGCACACGCAGGGACCTACAGATGTCGGCGTTTTC :   376
AOTVOKIR4DS10*01v2  : CGTACCCATCCTCAATGGCGTTATATTCCAGGAGAGCTTCTTGATGGGCCCCGTGACTGCAGCACACGCAGGGACCTACAGATGTCGGCGTTTTC :   577
AOTVOKIR3DS10*01v3  : ----------------------------------------------------------------------------------------------- :     -
AOTVOKIR3DS10*01v4  : ----------------------------------------------------------------------------------------------- :     -

                        4660         *       4680         *       4700         *       4720         *       4740         *
BAC_clone_Om        : ACCCGCACTACCCCACTGGGTGGACGGCACACAGCAACCCCCTGAAGATCATAGTCACAGGTCAGAGGGCTCCTGCCTGGGCTTCTCACTGTCCC :  4750
AOTVOKIR3DL4*01     : ----------------------------------------------------------------------------------------------- :     -
AOTVOKIR3DL4*02     : ----------------------------------------------------------------------------------------------- :     -
```

```
AOTVOKIR3DL4*02v1  : ----------------------------------------------------------------------------------------------- :    -
AOTVOKIR3DL4*02v2  : ----------------------------------------------------------------------------------------------- :    -
AOTVOKIR3DL4*03    : ----------------------------------------------------------------------------------------------- :    -
AOTVOKIR3DS4*04    : ----------------------------------------------------------------------------------------------- :    -
AOTVOKIR3DS4*04v1  : ----------------------------------------------------------------------------------------------- :    -
AOTVOKIR3DS5*01    : ----------------------------------------------------------------------------------------------- :    -
AOTVOKIR2DS5*01v1  : ----------------------------------------------------------------------------------------------- :    -
AOTVOKIR2DS5*01v2  : ----------------------------------------------------------------------------------------------- :    -
AOTVOKIR3DS7*01    : ----------------------------------------------------------------------------------------------- :    -
AOTVOKIR3DS7*01v1  : ----------------------------------------------------------------------------------------------- :    -
AOTVOKIR3DL8*01    : ----------------------------------------------------------------------------------------------- :    -
AOTVOKIR3DL8*01v1  : ----------------------------------------------------------------------------------------------- :    -
AOTVOKIR3DS8*01v2  : ----------------------------------------------------------------------------------------------- :    -
AOTVOKIR3DL8*02    : ----------------------------------------------------------------------------------------------- :    -
AOTVOKIR4DL9*01    : ACCCGCACTCCTCCACTGGGCGGACGGCACCCAGCAACCCCCTGGAGATCATAGTCACAG----------------------------------- :  637
AOTVOKIR4DS9*01v1  : ACCCGCACTCCTCCACTGGGCGGACGGCACCCAGCAACCCCCTGGAGATCATAGTCACAG----------------------------------- :  637
AOTVOKIR3DL9*01v2  : ACCCGCACTCCTCCACTGGGCGGACGGCACCCAGCAACCCCCTGGAGATCATAGTCACAG----------------------------------- :  439
AOTVOKIR4DL9*02    : ACCCGCACTCCTCCACTGGGCGGACGGCACCCAGCAACCCCCTGGAGATCATAGTCACAG----------------------------------- :  637
AOTVOKIR4DL10*01   : ACCCGCACTCCTCCACTGGGCGGACGGCACCCAGCAACGCCCTGAAGATCATAGTCACAG----------------------------------- :  637
AOTVOKIR3DL10*01v1 : ACCCGCACTCCTCCACTGGGCGGACGGCACCCAGCAACGCCCTGAAGATCATAGTCACAG----------------------------------- :  436
AOTVOKIR4DS10*01v2 : ACCCGCACTCCTCCACTGGGCGGACGGCACCCAGCAACGCCCTGAAGATCATAGTCACAG----------------------------------- :  637
AOTVOKIR3DS10*01v3 : ----------------------------------------------------------------------------------------------- :    -
AOTVOKIR3DS10*01v4 : ----------------------------------------------------------------------------------------------- :    -

                              4760         *        4780         *        4800         *        4820         *        4840
BAC_clone_Om       : ACTTCCTGAATCCCAGAGCTTTTGGTGGGGTGCCCATCAGGGTCCAATCACCGAGGCCCTGACTGTATTTGGGGTAAAGGGGATTGAATACAGGG : 4845
AOTVOKIR3DL4*01    : ----------------------------------------------------------------------------------------------- :    -
AOTVOKIR3DL4*02    : ----------------------------------------------------------------------------------------------- :    -
AOTVOKIR3DL4*02v1  : ----------------------------------------------------------------------------------------------- :    -
AOTVOKIR3DL4*02v2  : ----------------------------------------------------------------------------------------------- :    -
AOTVOKIR3DL4*03    : ----------------------------------------------------------------------------------------------- :    -
AOTVOKIR3DS4*04    : ----------------------------------------------------------------------------------------------- :    -
AOTVOKIR3DS4*04v1  : ----------------------------------------------------------------------------------------------- :    -
AOTVOKIR3DS5*01    : ----------------------------------------------------------------------------------------------- :    -
AOTVOKIR2DS5*01v1  : ----------------------------------------------------------------------------------------------- :    -
AOTVOKIR2DS5*01v2  : ----------------------------------------------------------------------------------------------- :    -
AOTVOKIR3DS7*01    : ----------------------------------------------------------------------------------------------- :    -
AOTVOKIR3DS7*01v1  : ----------------------------------------------------------------------------------------------- :    -
AOTVOKIR3DL8*01    : ----------------------------------------------------------------------------------------------- :    -
AOTVOKIR3DL8*01v1  : ----------------------------------------------------------------------------------------------- :    -
AOTVOKIR3DS8*01v2  : ----------------------------------------------------------------------------------------------- :    -
AOTVOKIR3DL8*02    : ----------------------------------------------------------------------------------------------- :    -
AOTVOKIR4DL9*01    : ----------------------------------------------------------------------------------------------- :    -
AOTVOKIR4DS9*01v1  : ----------------------------------------------------------------------------------------------- :    -
AOTVOKIR3DL9*01v2  : ----------------------------------------------------------------------------------------------- :    -
```

```
AOTVOKIR4DL9*02     : ----------------------------------------------------------------------------------------------- :     -
AOTVOKIR4DL10*01    : ----------------------------------------------------------------------------------------------- :     -
AOTVOKIR3DL10*01v1  : ----------------------------------------------------------------------------------------------- :     -
AOTVOKIR4DS10*01v2  : ----------------------------------------------------------------------------------------------- :     -
AOTVOKIR3DS10*01v3  : ----------------------------------------------------------------------------------------------- :     -
AOTVOKIR3DS10*01v4  : ----------------------------------------------------------------------------------------------- :     -

                          *      4860         *      4880         *      4900         *      4920         *      4940
BAC_clone_Om        : AAGTGGGTGCCATGGTGGGAAGAATAACTGTCCCCACTGATGGCCACATTCTCATCCCTGGAACCTGTGACTATTTATGTTACAGGGCAGGGGAC :  4940
AOTVOKIR3DL4*01     : ----------------------------------------------------------------------------------------------- :     -
AOTVOKIR3DL4*02     : ----------------------------------------------------------------------------------------------- :     -
AOTVOKIR3DL4*02v1   : ----------------------------------------------------------------------------------------------- :     -
AOTVOKIR3DL4*02v2   : ----------------------------------------------------------------------------------------------- :     -
AOTVOKIR3DL4*03     : ----------------------------------------------------------------------------------------------- :     -
AOTVOKIR3DS4*04     : ----------------------------------------------------------------------------------------------- :     -
AOTVOKIR3DS4*04v1   : ----------------------------------------------------------------------------------------------- :     -
AOTVOKIR3DS5*01     : ----------------------------------------------------------------------------------------------- :     -
AOTVOKIR2DS5*01v1   : ----------------------------------------------------------------------------------------------- :     -
AOTVOKIR2DS5*01v2   : ----------------------------------------------------------------------------------------------- :     -
AOTVOKIR3DS7*01     : ----------------------------------------------------------------------------------------------- :     -
AOTVOKIR3DS7*01v1   : ----------------------------------------------------------------------------------------------- :     -
AOTVOKIR3DL8*01     : ----------------------------------------------------------------------------------------------- :     -
AOTVOKIR3DL8*01v1   : ----------------------------------------------------------------------------------------------- :     -
AOTVOKIR3DS8*01v2   : ----------------------------------------------------------------------------------------------- :     -
AOTVOKIR3DL8*02     : ----------------------------------------------------------------------------------------------- :     -
AOTVOKIR4DL9*01     : ----------------------------------------------------------------------------------------------- :     -
AOTVOKIR4DS9*01v1   : ----------------------------------------------------------------------------------------------- :     -
AOTVOKIR3DL9*01v2   : ----------------------------------------------------------------------------------------------- :     -
AOTVOKIR4DL9*02     : ----------------------------------------------------------------------------------------------- :     -
AOTVOKIR4DL10*01    : ----------------------------------------------------------------------------------------------- :     -
AOTVOKIR3DL10*01v1  : ----------------------------------------------------------------------------------------------- :     -
AOTVOKIR4DS10*01v2  : ----------------------------------------------------------------------------------------------- :     -
AOTVOKIR3DS10*01v3  : ----------------------------------------------------------------------------------------------- :     -
AOTVOKIR3DS10*01v4  : ----------------------------------------------------------------------------------------------- :     -

                               *      4960         *      4980         *      5000         *      5020         *
BAC_clone_Om        : TGAAGGGGGAAGATGGAGCTCAGGTTGTTGATGAGTTGACCTTGAGATGGGGAGGCCGCCTGGACTGTCCAGCTGGGCTCAGTGTCATCACAAGT :  5035
AOTVOKIR3DL4*01     : ----------------------------------------------------------------------------------------------- :     -
AOTVOKIR3DL4*02     : ----------------------------------------------------------------------------------------------- :     -
AOTVOKIR3DL4*02v1   : ----------------------------------------------------------------------------------------------- :     -
AOTVOKIR3DL4*02v2   : ----------------------------------------------------------------------------------------------- :     -
AOTVOKIR3DL4*03     : ----------------------------------------------------------------------------------------------- :     -
AOTVOKIR3DS4*04     : ----------------------------------------------------------------------------------------------- :     -
AOTVOKIR3DS4*04v1   : ----------------------------------------------------------------------------------------------- :     -
```

```
AOTVOKIR3DS5*01    : ----------------------------------------------------------------------------------------------- :    -
AOTVOKIR2DS5*01v1  : ----------------------------------------------------------------------------------------------- :    -
AOTVOKIR2DS5*01v2  : ----------------------------------------------------------------------------------------------- :    -
AOTVOKIR3DS7*01    : ----------------------------------------------------------------------------------------------- :    -
AOTVOKIR3DS7*01v1  : ----------------------------------------------------------------------------------------------- :    -
AOTVOKIR3DL8*01    : ----------------------------------------------------------------------------------------------- :    -
AOTVOKIR3DL8*01v1  : ----------------------------------------------------------------------------------------------- :    -
AOTVOKIR3DS8*01v2  : ----------------------------------------------------------------------------------------------- :    -
AOTVOKIR3DL8*02    : ----------------------------------------------------------------------------------------------- :    -
AOTVOKIR4DL9*01    : ----------------------------------------------------------------------------------------------- :    -
AOTVOKIR4DS9*01v1  : ----------------------------------------------------------------------------------------------- :    -
AOTVOKIR3DL9*01v2  : ----------------------------------------------------------------------------------------------- :    -
AOTVOKIR4DL9*02    : ----------------------------------------------------------------------------------------------- :    -
AOTVOKIR4DL10*01   : ----------------------------------------------------------------------------------------------- :    -
AOTVOKIR3DL10*01v1 : ----------------------------------------------------------------------------------------------- :    -
AOTVOKIR4DS10*01v2 : ----------------------------------------------------------------------------------------------- :    -
AOTVOKIR3DS10*01v3 : ----------------------------------------------------------------------------------------------- :    -
AOTVOKIR3DS10*01v4 : ----------------------------------------------------------------------------------------------- :    -

                         5040         *       5060         *       5080         *       5100         *       5120         *
BAC_clone_Om       : GCCCACATGCGAGGAGGAGGAAGAGGGGAGTGGGGATTAGAGCAGTGTAGTGGGAGGGAGACTCCGCCAGCCACTGCAGGCTCTGAAGGTGGAGG :  5130
AOTVOKIR3DL4*01    : ----------------------------------------------------------------------------------------------- :    -
AOTVOKIR3DL4*02    : ----------------------------------------------------------------------------------------------- :    -
AOTVOKIR3DL4*02v1  : ----------------------------------------------------------------------------------------------- :    -
AOTVOKIR3DL4*02v2  : ----------------------------------------------------------------------------------------------- :    -
AOTVOKIR3DL4*03    : ----------------------------------------------------------------------------------------------- :    -
AOTVOKIR3DS4*04    : ----------------------------------------------------------------------------------------------- :    -
AOTVOKIR3DS4*04v1  : ----------------------------------------------------------------------------------------------- :    -
AOTVOKIR3DS5*01    : ----------------------------------------------------------------------------------------------- :    -
AOTVOKIR2DS5*01v1  : ----------------------------------------------------------------------------------------------- :    -
AOTVOKIR2DS5*01v2  : ----------------------------------------------------------------------------------------------- :    -
AOTVOKIR3DS7*01    : ----------------------------------------------------------------------------------------------- :    -
AOTVOKIR3DS7*01v1  : ----------------------------------------------------------------------------------------------- :    -
AOTVOKIR3DL8*01    : ----------------------------------------------------------------------------------------------- :    -
AOTVOKIR3DL8*01v1  : ----------------------------------------------------------------------------------------------- :    -
AOTVOKIR3DS8*01v2  : ----------------------------------------------------------------------------------------------- :    -
AOTVOKIR3DL8*02    : ----------------------------------------------------------------------------------------------- :    -
AOTVOKIR4DL9*01    : ----------------------------------------------------------------------------------------------- :    -
AOTVOKIR4DS9*01v1  : ----------------------------------------------------------------------------------------------- :    -
AOTVOKIR3DL9*01v2  : ----------------------------------------------------------------------------------------------- :    -
AOTVOKIR4DL9*02    : ----------------------------------------------------------------------------------------------- :    -
AOTVOKIR4DL10*01   : ----------------------------------------------------------------------------------------------- :    -
AOTVOKIR3DL10*01v1 : ----------------------------------------------------------------------------------------------- :    -
AOTVOKIR4DS10*01v2 : ----------------------------------------------------------------------------------------------- :    -
AOTVOKIR3DS10*01v3 : ----------------------------------------------------------------------------------------------- :    -
```

```
AOTVOKIR3DS10*01v4 : ----------------------------------------------------------------------------------------------- :    -

                           5140         *      5160         *      5180         *      5200         *      5220
BAC_clone_Om       : ACGGCCAGGAGCCATGCATGCAGGTGGCCTCTAGGGGCTGGAGAAGTCAAGGGAACTGATTCTCCCCTGAGTCTCCAGAGGGGACGCAGCCCTGC : 5225
AOTVOKIR3DL4*01    : ----------------------------------------------------------------------------------------------- :    -
AOTVOKIR3DL4*02    : ----------------------------------------------------------------------------------------------- :    -
AOTVOKIR3DL4*02v1  : ----------------------------------------------------------------------------------------------- :    -
AOTVOKIR3DL4*02v2  : ----------------------------------------------------------------------------------------------- :    -
AOTVOKIR3DL4*03    : ----------------------------------------------------------------------------------------------- :    -
AOTVOKIR3DS4*04    : ----------------------------------------------------------------------------------------------- :    -
AOTVOKIR3DS4*04v1  : ----------------------------------------------------------------------------------------------- :    -
AOTVOKIR3DS5*01    : ----------------------------------------------------------------------------------------------- :    -
AOTVOKIR2DS5*01v1  : ----------------------------------------------------------------------------------------------- :    -
AOTVOKIR2DS5*01v2  : ----------------------------------------------------------------------------------------------- :    -
AOTVOKIR3DS7*01    : ----------------------------------------------------------------------------------------------- :    -
AOTVOKIR3DS7*01v1  : ----------------------------------------------------------------------------------------------- :    -
AOTVOKIR3DL8*01    : ----------------------------------------------------------------------------------------------- :    -
AOTVOKIR3DL8*01v1  : ----------------------------------------------------------------------------------------------- :    -
AOTVOKIR3DS8*01v2  : ----------------------------------------------------------------------------------------------- :    -
AOTVOKIR3DL8*02    : ----------------------------------------------------------------------------------------------- :    -
AOTVOKIR4DL9*01    : ----------------------------------------------------------------------------------------------- :    -
AOTVOKIR4DS9*01v1  : ----------------------------------------------------------------------------------------------- :    -
AOTVOKIR3DL9*01v2  : ----------------------------------------------------------------------------------------------- :    -
AOTVOKIR4DL9*02    : ----------------------------------------------------------------------------------------------- :    -
AOTVOKIR4DL10*01   : ----------------------------------------------------------------------------------------------- :    -
AOTVOKIR3DL10*01v1 : ----------------------------------------------------------------------------------------------- :    -
AOTVOKIR4DS10*01v2 : ----------------------------------------------------------------------------------------------- :    -
AOTVOKIR3DS10*01v3 : ----------------------------------------------------------------------------------------------- :    -
AOTVOKIR3DS10*01v4 : ----------------------------------------------------------------------------------------------- :    -

                         *      5240         *      5260         *      5280         *      5300         *      5320
BAC_clone_Om       : AGATGCCCTGATTTTAGCCATGGGAGAACTGGGTCCGATTTCTGTCTCCAGAAGTGGAAGGGCTCAGTGTGTTCTCTTCTGCTGCCGTATTGTGA : 5320
AOTVOKIR3DL4*01    : ----------------------------------------------------------------------------------------------- :    -
AOTVOKIR3DL4*02    : ----------------------------------------------------------------------------------------------- :    -
AOTVOKIR3DL4*02v1  : ----------------------------------------------------------------------------------------------- :    -
AOTVOKIR3DL4*02v2  : ----------------------------------------------------------------------------------------------- :    -
AOTVOKIR3DL4*03    : ----------------------------------------------------------------------------------------------- :    -
AOTVOKIR3DS4*04    : ----------------------------------------------------------------------------------------------- :    -
AOTVOKIR3DS4*04v1  : ----------------------------------------------------------------------------------------------- :    -
AOTVOKIR3DS5*01    : ----------------------------------------------------------------------------------------------- :    -
AOTVOKIR2DS5*01v1  : ----------------------------------------------------------------------------------------------- :    -
AOTVOKIR2DS5*01v2  : ----------------------------------------------------------------------------------------------- :    -
AOTVOKIR3DS7*01    : ----------------------------------------------------------------------------------------------- :    -
AOTVOKIR3DS7*01v1  : ----------------------------------------------------------------------------------------------- :    -
```

```
AOTVOKIR3DL8*01     : ----------------------------------------------------------------------------------------------- :     -
AOTVOKIR3DL8*01v1   : ----------------------------------------------------------------------------------------------- :     -
AOTVOKIR3DS8*01v2   : ----------------------------------------------------------------------------------------------- :     -
AOTVOKIR3DL8*02     : ----------------------------------------------------------------------------------------------- :     -
AOTVOKIR4DL9*01     : ----------------------------------------------------------------------------------------------- :     -
AOTVOKIR4DS9*01v1   : ----------------------------------------------------------------------------------------------- :     -
AOTVOKIR3DL9*01v2   : ----------------------------------------------------------------------------------------------- :     -
AOTVOKIR4DL9*02     : ----------------------------------------------------------------------------------------------- :     -
AOTVOKIR4DL10*01    : ----------------------------------------------------------------------------------------------- :     -
AOTVOKIR3DL10*01v1  : ----------------------------------------------------------------------------------------------- :     -
AOTVOKIR4DS10*01v2  : ----------------------------------------------------------------------------------------------- :     -
AOTVOKIR3DS10*01v3  : ----------------------------------------------------------------------------------------------- :     -
AOTVOKIR3DS10*01v4  : ----------------------------------------------------------------------------------------------- :     -

                               *      5340          *      5360          *      5380          *      5400          *
BAC_clone_Om        : TAATTTTCTACAGCAGCAACAGGACACAACACAGGAACCCAGGGAAAGGACAAGTCAAGAAACCACACCAGGAGAAGGGTGGCCACCCCAAGATC :  5415
AOTVOKIR3DL4*01     : ----------------------------------------------------------------------------------------------- :     -
AOTVOKIR3DL4*02     : ----------------------------------------------------------------------------------------------- :     -
AOTVOKIR3DL4*02v1   : ----------------------------------------------------------------------------------------------- :     -
AOTVOKIR3DL4*02v2   : ----------------------------------------------------------------------------------------------- :     -
AOTVOKIR3DL4*03     : ----------------------------------------------------------------------------------------------- :     -
AOTVOKIR3DS4*04     : ----------------------------------------------------------------------------------------------- :     -
AOTVOKIR3DS4*04v1   : ----------------------------------------------------------------------------------------------- :     -
AOTVOKIR3DS5*01     : ----------------------------------------------------------------------------------------------- :     -
AOTVOKIR2DS5*01v1   : ----------------------------------------------------------------------------------------------- :     -
AOTVOKIR2DS5*01v2   : ----------------------------------------------------------------------------------------------- :     -
AOTVOKIR3DS7*01     : ----------------------------------------------------------------------------------------------- :     -
AOTVOKIR3DS7*01v1   : ----------------------------------------------------------------------------------------------- :     -
AOTVOKIR3DL8*01     : ----------------------------------------------------------------------------------------------- :     -
AOTVOKIR3DL8*01v1   : ----------------------------------------------------------------------------------------------- :     -
AOTVOKIR3DS8*01v2   : ----------------------------------------------------------------------------------------------- :     -
AOTVOKIR3DL8*02     : ----------------------------------------------------------------------------------------------- :     -
AOTVOKIR4DL9*01     : ----------------------------------------------------------------------------------------------- :     -
AOTVOKIR4DS9*01v1   : ----------------------------------------------------------------------------------------------- :     -
AOTVOKIR3DL9*01v2   : ----------------------------------------------------------------------------------------------- :     -
AOTVOKIR4DL9*02     : ----------------------------------------------------------------------------------------------- :     -
AOTVOKIR4DL10*01    : ----------------------------------------------------------------------------------------------- :     -
AOTVOKIR3DL10*01v1  : ----------------------------------------------------------------------------------------------- :     -
AOTVOKIR4DS10*01v2  : ----------------------------------------------------------------------------------------------- :     -
AOTVOKIR3DS10*01v3  : ----------------------------------------------------------------------------------------------- :     -
AOTVOKIR3DS10*01v4  : ----------------------------------------------------------------------------------------------- :     -

                        5420          *      5440          *      5460          *      5480          *      5500          *
BAC_clone_Om        : AGCAAGGGGGGGATGCTGAGGCCACCACCAGGCTCGATCCTCATAGGGAGGGGTCGATGCTCCTGGAACCAGCACCAGGGGCCGCCTATGGAAGC :  5510
```

```
AOTVOKIR3DL4*01    : ----------------------------------------------------------------------------------------------- :     -
AOTVOKIR3DL4*02    : ----------------------------------------------------------------------------------------------- :     -
AOTVOKIR3DL4*02v1  : ----------------------------------------------------------------------------------------------- :     -
AOTVOKIR3DL4*02v2  : ----------------------------------------------------------------------------------------------- :     -
AOTVOKIR3DL4*03    : ----------------------------------------------------------------------------------------------- :     -
AOTVOKIR3DS4*04    : ----------------------------------------------------------------------------------------------- :     -
AOTVOKIR3DS4*04v1  : ----------------------------------------------------------------------------------------------- :     -
AOTVOKIR3DS5*01    : ----------------------------------------------------------------------------------------------- :     -
AOTVOKIR2DS5*01v1  : ----------------------------------------------------------------------------------------------- :     -
AOTVOKIR2DS5*01v2  : ----------------------------------------------------------------------------------------------- :     -
AOTVOKIR3DS7*01    : ----------------------------------------------------------------------------------------------- :     -
AOTVOKIR3DS7*01v1  : ----------------------------------------------------------------------------------------------- :     -
AOTVOKIR3DL8*01    : ----------------------------------------------------------------------------------------------- :     -
AOTVOKIR3DL8*01v1  : ----------------------------------------------------------------------------------------------- :     -
AOTVOKIR3DS8*01v2  : ----------------------------------------------------------------------------------------------- :     -
AOTVOKIR3DL8*02    : ----------------------------------------------------------------------------------------------- :     -
AOTVOKIR4DL9*01    : ----------------------------------------------------------------------------------------------- :     -
AOTVOKIR4DS9*01v1  : ----------------------------------------------------------------------------------------------- :     -
AOTVOKIR3DL9*01v2  : ----------------------------------------------------------------------------------------------- :     -
AOTVOKIR4DL9*02    : ----------------------------------------------------------------------------------------------- :     -
AOTVOKIR4DL10*01   : ----------------------------------------------------------------------------------------------- :     -
AOTVOKIR3DL10*01v1 : ----------------------------------------------------------------------------------------------- :     -
AOTVOKIR4DS10*01v2 : ----------------------------------------------------------------------------------------------- :     -
AOTVOKIR3DS10*01v3 : ----------------------------------------------------------------------------------------------- :     -
AOTVOKIR3DS10*01v4 : ----------------------------------------------------------------------------------------------- :     -

                           5520         *      5540         *      5560         *      5580         *      5600
BAC_clone_Om       : CGGGACCATGGAGAAGCACAGACATGGCAGGAGAGGCTCCCAGTCCCCACCAGGAACAGGGTGTGTGGACACTGCTGCCCGCCTTACTCATCAGT :  5605
AOTVOKIR3DL4*01    : ----------------------------------------------------------------------------------------------- :     -
AOTVOKIR3DL4*02    : ----------------------------------------------------------------------------------------------- :     -
AOTVOKIR3DL4*02v1  : ----------------------------------------------------------------------------------------------- :     -
AOTVOKIR3DL4*02v2  : ----------------------------------------------------------------------------------------------- :     -
AOTVOKIR3DL4*03    : ----------------------------------------------------------------------------------------------- :     -
AOTVOKIR3DS4*04    : ----------------------------------------------------------------------------------------------- :     -
AOTVOKIR3DS4*04v1  : ----------------------------------------------------------------------------------------------- :     -
AOTVOKIR3DS5*01    : ----------------------------------------------------------------------------------------------- :     -
AOTVOKIR2DS5*01v1  : ----------------------------------------------------------------------------------------------- :     -
AOTVOKIR2DS5*01v2  : ----------------------------------------------------------------------------------------------- :     -
AOTVOKIR3DS7*01    : ----------------------------------------------------------------------------------------------- :     -
AOTVOKIR3DS7*01v1  : ----------------------------------------------------------------------------------------------- :     -
AOTVOKIR3DL8*01    : ----------------------------------------------------------------------------------------------- :     -
AOTVOKIR3DL8*01v1  : ----------------------------------------------------------------------------------------------- :     -
AOTVOKIR3DS8*01v2  : ----------------------------------------------------------------------------------------------- :     -
AOTVOKIR3DL8*02    : ----------------------------------------------------------------------------------------------- :     -
AOTVOKIR4DL9*01    : ----------------------------------------------------------------------------------------------- :     -
```

```
AOTVOKIR4DS9*01v1  : ----------------------------------------------------------------------------------------------- :     -
AOTVOKIR3DL9*01v2  : ----------------------------------------------------------------------------------------------- :     -
AOTVOKIR4DL9*02    : ----------------------------------------------------------------------------------------------- :     -
AOTVOKIR4DL10*01   : ----------------------------------------------------------------------------------------------- :     -
AOTVOKIR3DL10*01v1 : ----------------------------------------------------------------------------------------------- :     -
AOTVOKIR4DS10*01v2 : ----------------------------------------------------------------------------------------------- :     -
AOTVOKIR3DS10*01v3 : ----------------------------------------------------------------------------------------------- :     -
AOTVOKIR3DS10*01v4 : ----------------------------------------------------------------------------------------------- :     -

                          *      5620          *      5640          *      5660          *      5680          *      5700
BAC_clone_Om       : TCATACCTCCTGCCAGGGATTCCAATTTGTCCAAAACACATTGAACCAGGCTGTTCAGATCCTGGACGTGCAGCCTGTCGTGGCTCCTCTTCCAC :  5700
AOTVOKIR3DL4*01    : ----------------------------------------------------------------------------------------------- :     -
AOTVOKIR3DL4*02    : ----------------------------------------------------------------------------------------------- :     -
AOTVOKIR3DL4*02v1  : ----------------------------------------------------------------------------------------------- :     -
AOTVOKIR3DL4*02v2  : ----------------------------------------------------------------------------------------------- :     -
AOTVOKIR3DL4*03    : ----------------------------------------------------------------------------------------------- :     -
AOTVOKIR3DS4*04    : ----------------------------------------------------------------------------------------------- :     -
AOTVOKIR3DS4*04v1  : ----------------------------------------------------------------------------------------------- :     -
AOTVOKIR3DS5*01    : ----------------------------------------------------------------------------------------------- :     -
AOTVOKIR2DS5*01v1  : ----------------------------------------------------------------------------------------------- :     -
AOTVOKIR2DS5*01v2  : ----------------------------------------------------------------------------------------------- :     -
AOTVOKIR3DS7*01    : ----------------------------------------------------------------------------------------------- :     -
AOTVOKIR3DS7*01v1  : ----------------------------------------------------------------------------------------------- :     -
AOTVOKIR3DL8*01    : ----------------------------------------------------------------------------------------------- :     -
AOTVOKIR3DL8*01v1  : ----------------------------------------------------------------------------------------------- :     -
AOTVOKIR3DS8*01v2  : ----------------------------------------------------------------------------------------------- :     -
AOTVOKIR3DL8*02    : ----------------------------------------------------------------------------------------------- :     -
AOTVOKIR4DL9*01    : ----------------------------------------------------------------------------------------------- :     -
AOTVOKIR4DS9*01v1  : ----------------------------------------------------------------------------------------------- :     -
AOTVOKIR3DL9*01v2  : ----------------------------------------------------------------------------------------------- :     -
AOTVOKIR4DL9*02    : ----------------------------------------------------------------------------------------------- :     -
AOTVOKIR4DL10*01   : ----------------------------------------------------------------------------------------------- :     -
AOTVOKIR3DL10*01v1 : ----------------------------------------------------------------------------------------------- :     -
AOTVOKIR4DS10*01v2 : ----------------------------------------------------------------------------------------------- :     -
AOTVOKIR3DS10*01v3 : ----------------------------------------------------------------------------------------------- :     -
AOTVOKIR3DS10*01v4 : ----------------------------------------------------------------------------------------------- :     -

                               *      5720          *      5740          *      5760          *      5780          *
BAC_clone_Om       : CCTCACATGGACAGGAAGAAACAGATTAGTGGGAAACAGAAACAGCTCAAGGATGAGGCTGAGCCCAGTGGGAAGGGAGTCAGGGGCTGCTAGA :  5795
AOTVOKIR3DL4*01    : ----------------------------------------------------------------------------------------------- :     -
AOTVOKIR3DL4*02    : ----------------------------------------------------------------------------------------------- :     -
AOTVOKIR3DL4*02v1  : ----------------------------------------------------------------------------------------------- :     -
AOTVOKIR3DL4*02v2  : ----------------------------------------------------------------------------------------------- :     -
AOTVOKIR3DL4*03    : ----------------------------------------------------------------------------------------------- :     -
```

```
AOTVOKIR3DS4*04     : ----------------------------------------------------------------------------------------------- :     -
AOTVOKIR3DS4*04v1   : ----------------------------------------------------------------------------------------------- :     -
AOTVOKIR3DS5*01     : ----------------------------------------------------------------------------------------------- :     -
AOTVOKIR2DS5*01v1   : ----------------------------------------------------------------------------------------------- :     -
AOTVOKIR2DS5*01v2   : ----------------------------------------------------------------------------------------------- :     -
AOTVOKIR3DS7*01     : ----------------------------------------------------------------------------------------------- :     -
AOTVOKIR3DS7*01v1   : ----------------------------------------------------------------------------------------------- :     -
AOTVOKIR3DL8*01     : ----------------------------------------------------------------------------------------------- :     -
AOTVOKIR3DL8*01v1   : ----------------------------------------------------------------------------------------------- :     -
AOTVOKIR3DS8*01v2   : ----------------------------------------------------------------------------------------------- :     -
AOTVOKIR3DL8*02     : ----------------------------------------------------------------------------------------------- :     -
AOTVOKIR4DL9*01     : ----------------------------------------------------------------------------------------------- :     -
AOTVOKIR4DS9*01v1   : ----------------------------------------------------------------------------------------------- :     -
AOTVOKIR3DL9*01v2   : ----------------------------------------------------------------------------------------------- :     -
AOTVOKIR4DL9*02     : ----------------------------------------------------------------------------------------------- :     -
AOTVOKIR4DL10*01    : ----------------------------------------------------------------------------------------------- :     -
AOTVOKIR3DL10*01v1  : ----------------------------------------------------------------------------------------------- :     -
AOTVOKIR4DS10*01v2  : ----------------------------------------------------------------------------------------------- :     -
AOTVOKIR3DS10*01v3  : ----------------------------------------------------------------------------------------------- :     -
AOTVOKIR3DS10*01v4  : ----------------------------------------------------------------------------------------------- :     -

                          5800           *       5820           *       5840           *       5860           *       5880           *
BAC_clone_Om        : GACAGAGACAGAGAAGAGGGAGGGAGACAGAGGGAGTGACCTGCACCAGGGGTGACGGGCACAGAAAGGAGCACGGAGACACAGAGAGGGAGGAG :  5890
AOTVOKIR3DL4*01     : ----------------------------------------------------------------------------------------------- :     -
AOTVOKIR3DL4*02     : ----------------------------------------------------------------------------------------------- :     -
AOTVOKIR3DL4*02v1   : ----------------------------------------------------------------------------------------------- :     -
AOTVOKIR3DL4*02v2   : ----------------------------------------------------------------------------------------------- :     -
AOTVOKIR3DL4*03     : ----------------------------------------------------------------------------------------------- :     -
AOTVOKIR3DS4*04     : ----------------------------------------------------------------------------------------------- :     -
AOTVOKIR3DS4*04v1   : ----------------------------------------------------------------------------------------------- :     -
AOTVOKIR3DS5*01     : ----------------------------------------------------------------------------------------------- :     -
AOTVOKIR2DS5*01v1   : ----------------------------------------------------------------------------------------------- :     -
AOTVOKIR2DS5*01v2   : ----------------------------------------------------------------------------------------------- :     -
AOTVOKIR3DS7*01     : ----------------------------------------------------------------------------------------------- :     -
AOTVOKIR3DS7*01v1   : ----------------------------------------------------------------------------------------------- :     -
AOTVOKIR3DL8*01     : ----------------------------------------------------------------------------------------------- :     -
AOTVOKIR3DL8*01v1   : ----------------------------------------------------------------------------------------------- :     -
AOTVOKIR3DS8*01v2   : ----------------------------------------------------------------------------------------------- :     -
AOTVOKIR3DL8*02     : ----------------------------------------------------------------------------------------------- :     -
AOTVOKIR4DL9*01     : ----------------------------------------------------------------------------------------------- :     -
AOTVOKIR4DS9*01v1   : ----------------------------------------------------------------------------------------------- :     -
AOTVOKIR3DL9*01v2   : ----------------------------------------------------------------------------------------------- :     -
AOTVOKIR4DL9*02     : ----------------------------------------------------------------------------------------------- :     -
AOTVOKIR4DL10*01    : ----------------------------------------------------------------------------------------------- :     -
AOTVOKIR3DL10*01v1  : ----------------------------------------------------------------------------------------------- :     -
```

```
AOTVOKIR4DS10*01v2 : ----------------------------------------------------------------------------------------------- :    -
AOTVOKIR3DS10*01v3 : ----------------------------------------------------------------------------------------------- :    -
AOTVOKIR3DS10*01v4 : ----------------------------------------------------------------------------------------------- :    -

                                  5900         *        5920         *        5940         *        5960         *        5980
BAC_clone_Om       : AGAGTCAGACACCGGGGAGGGGAAGCCTCACTCATTCCAGGTCCCATCGATGGGAAGACAAAGGGAGATGCCTTCTAAACTCACAATATTTTTTC : 5985
AOTVOKIR3DL4*01    : ----------------------------------------------------------------------------------------------- :    -
AOTVOKIR3DL4*02    : ----------------------------------------------------------------------------------------------- :    -
AOTVOKIR3DL4*02v1  : ----------------------------------------------------------------------------------------------- :    -
AOTVOKIR3DL4*02v2  : ----------------------------------------------------------------------------------------------- :    -
AOTVOKIR3DL4*03    : ----------------------------------------------------------------------------------------------- :    -
AOTVOKIR3DS4*04    : ----------------------------------------------------------------------------------------------- :    -
AOTVOKIR3DS4*04v1  : ----------------------------------------------------------------------------------------------- :    -
AOTVOKIR3DS5*01    : ----------------------------------------------------------------------------------------------- :    -
AOTVOKIR2DS5*01v1  : ----------------------------------------------------------------------------------------------- :    -
AOTVOKIR2DS5*01v2  : ----------------------------------------------------------------------------------------------- :    -
AOTVOKIR3DS7*01    : ----------------------------------------------------------------------------------------------- :    -
AOTVOKIR3DS7*01v1  : ----------------------------------------------------------------------------------------------- :    -
AOTVOKIR3DL8*01    : ----------------------------------------------------------------------------------------------- :    -
AOTVOKIR3DL8*01v1  : ----------------------------------------------------------------------------------------------- :    -
AOTVOKIR3DS8*01v2  : ----------------------------------------------------------------------------------------------- :    -
AOTVOKIR3DL8*02    : ----------------------------------------------------------------------------------------------- :    -
AOTVOKIR4DL9*01    : ----------------------------------------------------------------------------------------------- :    -
AOTVOKIR4DS9*01v1  : ----------------------------------------------------------------------------------------------- :    -
AOTVOKIR3DL9*01v2  : ----------------------------------------------------------------------------------------------- :    -
AOTVOKIR4DL9*02    : ----------------------------------------------------------------------------------------------- :    -
AOTVOKIR4DL10*01   : ----------------------------------------------------------------------------------------------- :    -
AOTVOKIR3DL10*01v1 : ----------------------------------------------------------------------------------------------- :    -
AOTVOKIR4DS10*01v2 : ----------------------------------------------------------------------------------------------- :    -
AOTVOKIR3DS10*01v3 : ----------------------------------------------------------------------------------------------- :    -
AOTVOKIR3DS10*01v4 : ----------------------------------------------------------------------------------------------- :    -

                                                                                   EXON 4
                         *       6000         *        6020         *        6040         *        6060         *        6080
BAC_clone_Om       : TTAGGATTCCACAGAAAACCTTCCCTCCTGGCCCTCCCAGCTCCCCTGGTGAAATTAGGAGAGACGGTCATCCTGCAGTGTTGGTCAGATGTCAT : 6080
AOTVOKIR3DL4*01    : ----GATTCCACAGAAAACCTTCCCTCCTGGCCCTCCCAGCTCCCCTGGTGAAATCAGGAGAGACCGTCATCCTGCAGTGTTGGTCAGATATCCT :  443
AOTVOKIR3DL4*02    : ----GATTCCACAGAAAACCTTCCCTCCTGGCCCTCCCAGCTCCCCTGGTGAAATCAGGAGAGACCGTCATCCTGCAGTGTTGGTCAGATATCCT :  443
AOTVOKIR3DL4*02v1  : ----GATTCCACAGAAAACCTTCCCTCCTGGCCCTCCCAGCTCCCCTGGTGAAATCAGGAGAGACCGTCATCCTGCAGTGTTGGTCAGATATCCT :  407
AOTVOKIR3DL4*02v2  : ----GATTCCACAGAAAACCTTCCCTCCTGGCCCTCCCAGCTCCCCTGGTGAAATCAGGAGAGACCGTCATCCTGCAGTGTTGGTCAGATATCCT :  443
AOTVOKIR3DL4*03    : ----GATTCCACAGAAAACCTTCCCTCCTGGCCCTCCCAGCTCCCCTGGTGAAATCAGGAGAGACCGTCATCCTGCAGTGTTGGTCAGATATCCT :  443
AOTVOKIR3DS4*04    : ----GATTCCACAGAAAACCTTCCCTCCTGGCCCTCCCAGCTCCCCTGGTGAAATCAGGAGAGACCGTCATCCTGCAGTGTTGGTCAGATATCCT :  443
AOTVOKIR3DS4*04v1  : ----GATTCCACAGAAAACCTTCCCTCCTGGCCCTCCCAGCTCCCCTGGTGAAATCAGGAGAGACCGTCATCCTGCAGTGTTGGTCAGATATCCT :  443
AOTVOKIR3DS5*01    : ----GATTCCACAGAAAACCTTCGCTCCTGGCCCTCCCAGCTCCCCTGGTGAAATCAGGACAGACGGTCATCCTGCAGTGTTGGTCAGATATCAT :  443
AOTVOKIR2DS5*01v1  : ----GATTCCACAGAAAACCTTCGCTCCTGGCCCTCCCAGCTCCCCTGGTGAAATCAGGACAGACGGTCATCCTGCAGTGTTGGTCAGATATCAT :  245
AOTVOKIR2DS5*01v2  : ----GATTCCACAGAAAACCTTCGCTCCTGGCCCTCCCAGCTCCCCTGGTGAAATCAGGACAGACGGTCATCCTGCAGTGTTGGTCAGATATCAT :  158
```

```
AOTVOKIR3DS7*01     : ----GATTCCACAGAAAACCTTCCCTCCTGGCCCTCCCAGCTCCCCTGGTGAAATCAGGAGAGAGGGTCATCCTGCAGTGTTGGTCAGATATCAT :   443
AOTVOKIR3DS7*01v1   : ----GATTCCACAGAAAACCTTCCCTCCTGGCCCTCCCAGCTCCCCTGGTGAAATCAGGAGAGAGGGTCATCCTGCAGTGTTGGTCAGATATCAT :   443
AOTVOKIR3DL8*01     : ----GATTCCACAGAAAACCTTCCCTCCTGGCCCTCCCAGCTCCCCTTGTGGCATCAGGAGAGACGGTCATCCTGCAGTGTTGGTCAGATATCAT :   443
AOTVOKIR3DL8*01v1   : ----GATTCCACAGAAAACCTTCCCTCCTGGCCCTCCCAGCTCCCCTTGTGGCATCAGGAGAGACGGTCATCCTGCAGTGTTGGTCAGATATCAT :   443
AOTVOKIR3DS8*01v2   : ----GATTCCACAGAAAACCTTCCCTCCTGGCCCTCCCAGCTCCCCTTGTGGCATCAGGAGAGACGGTCATCCTGCAGTGTTGGTCAGATATCAT :   443
AOTVOKIR3DL8*02     : ----GATTCCACAGAAAACCTTCCCTCCTGGCCCTCCCAGCTCCCCTTGTGGCATCAGGAGAGACGGTCATCCTGCAGTGTTGGTCAGATATCAT :   443
AOTVOKIR4DL9*01     : ----GACTCCACAGAAAACCTTCCCTTCTGGCCATCCCAGCTCCCCTGGTGACATCAGGCAAGACGGTCATCCTGCAATGTTGGTCAGATATCAT :   728
AOTVOKIR4DS9*01v1   : ----GACTCCACAGAAAACCTTCCCTTCTGGCCATCCCAGCTCCCCTGGTGACATCAGGCAAGACGGTCATCCTGCAATGTTGGTCAGATATCAT :   728
AOTVOKIR3DL9*01v2   : ----GACTCCACAGAAAACCTTCCCTTCTGGCCATCCCAGCTCCCCTGGTGACATCAGGCAAGACGGTCATCCTGCAATGTTGGTCAGATATCAT :   530
AOTVOKIR4DL9*02     : ----GACTCCACAGAAAACCTTCCCTTCTGGCCATCCCAGCTCCCCTGGTGACATCAGGCAAGACGGTCATCCTGCAATGTTGGTCAGATATCAT :   728
AOTVOKIR4DL10*01    : ----GATTCCACAGAAAACCTTCCCTTCTGGCCCTCCCAGCTCCCCTGGTGACATCAGGAAAGACGGTCATCCTGCAGTGTTGGTCAGATATCAT :   728
AOTVOKIR3DL10*01v1  : ----GATTCCACAGAAAACCTTCCCTTCTGGCCCTCCCAGCTCCCCTGGTGACATCAGGAAAGACGGTCATCCTGCAGTGTTGGTCAGATATCAT :   527
AOTVOKIR4DS10*01v2  : ----GATTCCACAGAAAACCTTCCCTTCTGGCCCTCCCAGCTCCCCTGGTGACATCAGGAAAGACGGTCATCCTGCAGTGTTGGTCAGATATCAT :   728
AOTVOKIR3DS10*01v3  : ----GATTCCACAGAAAACCTTCCCTTCTGGCCCTCCCAGCTCCCCTGGTGACATCAGGAAAGACGGTCATCCTGCAGTGTTGGTCAGATATCAT :   443
AOTVOKIR3DS10*01v4  : ----GATTCCACAGAAAACCTTCCCTTCTGGCCCTCCCAGCTCCCCTGGTGACATCAGGAAAGACGGTCATCCTGCAGTGTTGGTCAGATATCAT :   443
                          GAtTCCACAGAAAACCTTCcCT CTGGCCcTCCCAGCTCCCCTgGTGa ATcAGGa AGAc GTCATCCTGCAgTGTTGGTCAGATaTC T

                                  *       6100        *       6120        *       6140        *       6160        *
BAC_clone_Om        : GTCTGAGCACTTCCTTCTGCACAGAGAGGGGATGTCTGAGGACACCTTGTGCCTCGTTGGAGAGCCCCATGATGGGGGCTCCCAGGCCATCTACT :  6175
AOTVOKIR3DL4*01     : GTTTGAACACTTATTTCTGCACAGAAAAGGGTTCACTGAGGACCTCTTGTGCCTCGTTGGAGAGCCCCATGATGGGGGCTCCCAGGCCAACTTCT :   538
AOTVOKIR3DL4*02     : GTTTGAACACTTATTTCTGCACAGAAAAGGGTTCACTGAGGACCTCTTGTGCCTCGTTGGAGAGCCCCGTGATGGGGGCTCCCAGGCCAACTTCT :   538
AOTVOKIR3DL4*02v1   : GTTTGAACACTTATTTCTGCACAGAAAAGGGTTCACTGAGGACCTCTTGTGCCTCGTTGGAGAGCCCCGTGATGGGGGCTCCCAGGCCAACTTCT :   502
AOTVOKIR3DL4*02v2   : GTTTGAACACTTATTTCTGCACAGAAAAGGGTTCACTGAGGACCTCTTGTGCCTCGTTGGAGAGCCCCGTGATGGGGGCTCCCAGGCCAACTTCT :   538
AOTVOKIR3DL4*03     : GTTTGAACACTTATTTCTGCACAGAAAAGGGTTCACTGAGGACCTCTTGTGCCTCGTTGGAGAGCCCCATGATGGGGGCTCCCAGGCCAACTTCT :   538
AOTVOKIR3DS4*04     : GTTTGAACACTTATTTCTGCACAGAAAAGGGTTCACTGAGGACCTCTTGTGCCTCGTTGGAGAGCCCCATGATGGGGGCTCCCAGGCCAACTTCT :   538
AOTVOKIR3DS4*04v1   : GTTTGAACACTTATTTCTGCACAGAAAAGGGTTCACTGAGGACCTCTTGTGCCTCGTTGGAGAGCCCCATGATGGGGGCTCCCAGGCCAACTTCT :   538
AOTVOKIR3DS5*01     : GTCTGAGCACTTCCTTCTGCACGGAAAGGGAGTCATTGAGGACCTCTTACACCTTGTTGGAGAGCCCCGTGATGGGGGCTCCCAGGCCAACTTCT :   538
AOTVOKIR2DS5*01v1   : GTCTGAGCACTTCCTTCTGCACGGAAAGGGAGTCATTGAGGACCTCTTACACCTTGTTGGAGAGCCCCGTGATGGGGGCTCCCAGGCCAACTTCT :   340
AOTVOKIR2DS5*01v2   : GTCTGAGCACTTCCTTCTGCACGGAAAGGGAGTCATTGAGGACCTCTTACACCTTGTTGGAGAGCCCCGTGATGGGGGCTCCCAGGCCAACTTCT :   253
AOTVOKIR3DS7*01     : GTCTGAGTACTTCCTTCTGCACAGGCAAGGGGTCACTGAGGACCTCTTGCGCCTCCTTGGAGAGTCCCGTGATGGAGGCTCCCAGGCCAACTTCT :   538
AOTVOKIR3DS7*01v1   : GTCTGAGTACTTCCTTCTGCACAGGCAAGGGGTCACTGAGGACCTCTTGCGCCTCCTTGGAGAGTCCCGTGATGGAGGCTCCCAGGCCAACTTCT :   538
AOTVOKIR3DL8*01     : GTCTGAGCACTTCCTTCTGCACGGAAAGGGGATCACTGAGGACCTCTTGCGCCTCGTTGGAGAGCCCCATAATGGGGGCTCCCAGGCCAACTTCT :   538
AOTVOKIR3DL8*01v1   : GTCTGAGCACTTCCTTCTGCACGGAAAGGGGATCACTGAGGACCTCTTGCGCCTCGTTGGAGAGCCCCATAATGGGGGCTCCCAGGCCAACTTCT :   538
AOTVOKIR3DS8*01v2   : GTCTGAGCACTTCCTTCTGCACGGAAAGGGGATCACTGAGGACCTCTTGCGCCTCGTTGGAGAGCCCCATAATGGGGGCTCCCAGGCCAACTTCT :   538
AOTVOKIR3DL8*02     : GTCTGAGCACTTCCTTCTGCACGGAAAGGGGATCACTGAGGACCTCTTGCGCCTCGTTGGAGAGCCCCATAATGGGGGCTCCCAGGCCAACTTCT :   538
AOTVOKIR4DL9*01     : GTCTGAGCACTTCCTTCTGCACAGAAAGGGGGTCACTGAGGACCTCTTGCGCCTCAATGGAGAGTCCCGTGACGGAGGCTCCCAGGCCAACTTCT :   823
AOTVOKIR4DS9*01v1   : GTCTGAGCACTTCCTTCTGCACAGAAAGGGGGTCACTGAGGACCTCTTGCGCCTCAATGGAGAGTCCCGTGACGGAGGCTCCCAGGCCAACTTCT :   823
AOTVOKIR3DL9*01v2   : GTCTGAGCACTTCCTTCTGCACAGAAAGGGGGTCACTGAGGACCTCTTGCGCCTCAATGGAGAGTCCCGTGACGGAGGCTCCCAGGCCAACTTCT :   625
AOTVOKIR4DL9*02     : GTCTGAGCACTTCCTTCTGCACAGAAAGGGGGTCACTGAGGACCTCTTGCGCCTCAATGGAGAGTCCCGTGACGGAGGCTCCCAGGCCAACTTCT :   823
AOTVOKIR4DL10*01    : GTCTGAGCACTTCCTTCTGCACAGAAAGGGGGTCACTGAGGATCTCTTGCGCCTCGATGGAGAGCCCCATGATGGAGGCTCCCAGGCCAACTTCT :   823
AOTVOKIR3DL10*01v1  : GTCTGAGCACTTCCTTCTGCACAGAAAGGGGGTCACTGAGGATCTCTTGCGCCTCGATGGAGAGCCCCATGATGGAGGCTCCCAGGCCAACTTCT :   622
AOTVOKIR4DS10*01v2  : GTCTGAGCACTTCCTTCTGCACAGAAAGGGGGTCACTGAGGATCTCTTGCGCCTCGATGGAGAGCCCCATGATGGAGGCTCCCAGGCCAACTTCT :   823
AOTVOKIR3DS10*01v3  : GTCTGAGCACTTCCTTCTGCACAGAAAGGGGGTCACTGAGGATCTCTTGCGCCTCGATGGAGAGCCCCATGATGGAGGCTCCCAGGCCAACTTCT :   538
AOTVOKIR3DS10*01v4  : GTCTGAGCACTTCCTTCTGCACAGAAAGGGGGTCACTGAGGATCTCTTGCGCCTCGATGGAGAGCCCCATGATGGAGGCTCCCAGGCCAACTTCT :   538
                      GT TGA cACTT  TTCTGCAC GaaA GGg TcacTGAGGAcctCTTg gCCTc  TGGAGAG CCC TgAtGG GGCTCCCAGGCCAaCTtCT
```

```
                         6180         *       6200         *       6220         *       6240         *       6260         *
BAC_clone_Om         : CCATAGGTCCCATGACAACTGACCTTGCAGGGACCTACAGATGCTACGGTTCTGTTACTCACTCCCCCTATGAGTGGTCAGCTCCCAGTGACCCC :  6270
AOTVOKIR3DL4*01      : CCATGGGTCCCATGATGCCTGCCCTTGCAGGGACCTACAGATGCTACGGTTCTTTCACTCACTCCCCCTATGAGTGGTCAGCTCCCAGTGACCCC :   633
AOTVOKIR3DL4*02      : CCATGGGTCCCATGATGCCTGCCCTTGCAGGGACCTACAGATGCTACGGTTCTTTCACTCACTCCCCCTATGAGTGGTCAGCTCCCAGTGACCCC :   633
AOTVOKIR3DL4*02v1    : CCATGGGTCCCATGATGCCTGCCCTTGCAGGGACCTACAGATGCTACGGTTCTTTCACTCACTCCCCCTATGAGTGGTCAGCTCCCAGTGACCCC :   597
AOTVOKIR3DL4*02v2    : CCATGGGTCCCATGATGCCTGCCCTTGCAGGGACCTACAGATGCTACGGTTCTTTCACTCACTCCCCCTATGAGTGGTCAGCTCCCAGTGACCCC :   633
AOTVOKIR3DL4*03      : CCATGGGTCCCATGATGCCTGCCCTTGCAGGGACCTACAGATGCTACGGTTCTTTCACTCACTCCCCCTATGAGTGGTCAGCTCCCAGTGACCCC :   633
AOTVOKIR3DS4*04      : CCATGGGTCCCATGATGCCTGCCCTTGCAGGGACCTACAGATGCTACGGTTCTTTCACTCACTCCCCCTATGAGTGGTCAGCTCCCAGTGACCCC :   633
AOTVOKIR3DS4*04v1    : CCATGGGTCCCATGATGCCTGCCCTTGCAGGGACCTACAGATGCTACGGTTCTTTCACTCACTCCCCCTATGAGTGGTCAGCTCCCAGTGACCCC :   633
AOTVOKIR3DS5*01      : CCATAGGTCCCATGATGACTGCCCTTGCAGGGACCTACAGATGCTACGGTTCTGTCACTCACTCCCCCTATGGGTGGTCGGCTCCCAGTGACCCC :   633
AOTVOKIR2DS5*01v1    : CCATAGGTCCCATGATGACTGCCCTTGCAGGGACCTACAGATGCTACGGTTCTGTCACTCACTCCCCCTATGAGTGGTCGGCTCCCAGTGACCCC :   435
AOTVOKIR2DS5*01v2    : CCATAGGTCCCATGATGACTGCCCTTGCAGGGACCTACAGATGCTACGGTTCTGTCACTCACTCCCCCTATGAGTGGTCGGCTCCCAGTGACCCC :   348
AOTVOKIR3DS7*01      : CCATAGGTCCCATGACAACTGACCTTGCAGGGACCTACAGATGCTACAGTTCTGTGACTCACTCCCCCTATGAGTGGTCAGCTCCCAGTGACCCC :   633
AOTVOKIR3DS7*01v1    : CCATAGGTCCCATGACAACTGACCTTGCAGGGACCTACAGATGCTACAGTTCTGTGACTCACTCCCCCTATGAGTGGTCAGCTCCCAGTGACCCC :   633
AOTVOKIR3DL8*01      : CCATAGGTCCCATGACGACTGCCCTTGCAGGGACCTACAGATGCTGCCGTTCTGTCACTCACTCCCCCTATGAGTGGTCAGCTCCCAGTGACCCC :   633
AOTVOKIR3DL8*01v1    : CCATAGGTCCCATGACGACTGCCCTTGCAGGGACCTACAGATGCTGCCGTTCTGTCACTCACTCCCCCTATGAGTGGTCAGCTCCCAGTGACCCC :   633
AOTVOKIR3DS8*01v2    : CCATAGGTCCCATGACGACTGCCCTTGCAGGGACCTACAGATGCTGCCGTTCTGTCACTCACTCCCCCTATGAGTGGTCAGCTCCCAGTGACCCC :   633
AOTVOKIR3DL8*02      : CCATAGGTCCCATGACGACTGCCCTTGCAGGGACCTACAGATGCTGCCGTTCTGTCACTCACTCCCCCTATGAGTGGTCAGCTCCCAGTGACCCC :   633
AOTVOKIR4DL9*01      : CCATAGGTCCCATGACGTCTGCCCTTGCAGGGACCTACAGATGCTACGGTTCTGTCACTCACTCCCGCTATGAGTGGTCAGCTCCCAGTGACCCT :   918
AOTVOKIR4DS9*01v1    : CCATAGGTCCCATGACGTCTGCCCTTGCAGGGACCTACAGATGCTACGGTTCTGTCACTCACTCCCGCTATGAGTGGTCAGCTCCCAGTGACCCT :   918
AOTVOKIR3DL9*01v2    : CCATAGGTCCCATGACGTCTGCCCTTGCAGGGACCTACAGATGCTACGGTTCTGTCACTCACTCCCGCTATGAGTGGTCAGCTCCCAGTGACCCT :   720
AOTVOKIR4DL9*02      : CCATAGGTCCCATGACGTCTGCCCTTGCAGGGACCTACAGATGCTACGGTTCTGTCACTCACTCCCGCTATGAGTGGTCAGCTCCCAGTGACCCT :   918
AOTVOKIR4DL10*01     : CCATAGGTCCCATGACGACTGCCCTTGCAGGGACCTACAGATGCTACGGTTCTGTCACTCACTCCCCCTATGAGTGGTCAGCTCCCAGTGACCCT :   918
AOTVOKIR3DL10*01v1   : CCATAGGTCCCATGACGACTGCCCTTGCAGGGACCTACAGATGCTACGGTTCTGTCACTCACTCCCCCTATGAGTGGTCAGCTCCCAGTGACCCT :   717
AOTVOKIR4DS10*01v2   : CCATAGGTCCCATGACGACTGCCCTTGCAGGGACCTACAGATGCTACGGTTCTGTCACTCACTCCCCCTATGAGTGGTCAGCTCCCAGTGACCCT :   918
AOTVOKIR3DS10*01v3   : CCATAGGTCCCATGACGACTGCCCTTGCAGGGACCTACAGATGCTACGGTTCTGTCACTCACTCCCCCTATGAGTGGTCAGCTCCCAGTGACCCT :   633
AOTVOKIR3DS10*01v4   : CCATAGGTCCCATGACGACTGCCCTTGCAGGGACCTACAGATGCTACGGTTCTGTCACTCACTCCCCCTATGAGTGGTCAGCTCCCAGTGACCCT :   633
                       CCAT GGTCCCATGA g CTGcCCTTGCAGGGACCTACAGATGCTaC GTTCT TcACTCACTCCCcCTATGaGTGGTCaGCTCCCAGTGACCC

                                 6280         *       6300         *       6320         *       6340         *       6360
BAC_clone_Om         : CTGGATATCGTGATCACAGGTGAGAGTGTCCAGACATTCTTCTCATTGTCACTGGGACACAGAGTGAATGATCCAGGACTTGGAAGCCCCAGGTG :  6365
AOTVOKIR3DL4*01      : CTGGACATCGGGATCACAG---------------------------------------------------------------------------- :   652
AOTVOKIR3DL4*02      : CTGGACATCGGGATCACAG---------------------------------------------------------------------------- :   652
AOTVOKIR3DL4*02v1    : CTGGACATCGGGATCACAG---------------------------------------------------------------------------- :   616
AOTVOKIR3DL4*02v2    : CTGGACATCGGGATCACAG---------------------------------------------------------------------------- :   652
AOTVOKIR3DL4*03      : CTGGACATCGGGATCACAG---------------------------------------------------------------------------- :   652
AOTVOKIR3DS4*04      : CTGGACATCGGGATCACAG---------------------------------------------------------------------------- :   652
AOTVOKIR3DS4*04v1    : CTGGACATCGGGATCACAG---------------------------------------------------------------------------- :   652
AOTVOKIR3DS5*01      : CTGGACATTGTGATCACAG---------------------------------------------------------------------------- :   652
AOTVOKIR2DS5*01v1    : CTGGACATTGTGATCACAG---------------------------------------------------------------------------- :   454
AOTVOKIR2DS5*01v2    : CTGGACATTGTGATCACAG---------------------------------------------------------------------------- :   367
AOTVOKIR3DS7*01      : CTGGACATCATGATCACAG---------------------------------------------------------------------------- :   652
AOTVOKIR3DS7*01v1    : CTGGACATCATGATCACAG---------------------------------------------------------------------------- :   652
AOTVOKIR3DL8*01      : CTGGACATCGTGATCACAG---------------------------------------------------------------------------- :   652
AOTVOKIR3DL8*01v1    : CTGGACATCGTGATCACAG---------------------------------------------------------------------------- :   652
AOTVOKIR3DS8*01v2    : CTGGACATCGTGATCACAG---------------------------------------------------------------------------- :   652
```

```
AOTVOKIR3DL8*02    : CTGGACATCGTGATCACAG---------------------------------------------------------------------------- :   652
AOTVOKIR4DL9*01    : CTGGACATCGTGATCACAG---------------------------------------------------------------------------- :   937
AOTVOKIR4DS9*01v1  : CTGGACATCGTGATCACAG---------------------------------------------------------------------------- :   937
AOTVOKIR3DL9*01v2  : CTGGACATCGTGATCACAG---------------------------------------------------------------------------- :   739
AOTVOKIR4DL9*02    : CTGGACATCGTGATCACAG---------------------------------------------------------------------------- :   937
AOTVOKIR4DL10*01   : CTGGACATCGTGATCACAG---------------------------------------------------------------------------- :   937
AOTVOKIR3DL10*01v1 : CTGGACATCGTGATCACAG---------------------------------------------------------------------------- :   736
AOTVOKIR4DS10*01v2 : CTGGACATCGTGATCACAG---------------------------------------------------------------------------- :   937
AOTVOKIR3DS10*01v3 : CTGGACATCGTGATCACAG---------------------------------------------------------------------------- :   652
AOTVOKIR3DS10*01v4 : CTGGACATCGTGATCACAG---------------------------------------------------------------------------- :   652
                     CTGGAcATcg GATCACAG

                         *      6380         *      6400         *      6420         *      6440         *      6460
BAC_clone_Om       : CTCATGAGGAAAATGAGCGTGGGGTTCTTATGGAGAGAGACTGACTTGGTGAGGTCTGTACCAGCAGAGACAGAGAAACAGGAGACACAAGTCCA :  6460
AOTVOKIR3DL4*01    : ----------------------------------------------------------------------------------------------- :     -
AOTVOKIR3DL4*02    : ----------------------------------------------------------------------------------------------- :     -
AOTVOKIR3DL4*02v1  : ----------------------------------------------------------------------------------------------- :     -
AOTVOKIR3DL4*02v2  : ----------------------------------------------------------------------------------------------- :     -
AOTVOKIR3DL4*03    : ----------------------------------------------------------------------------------------------- :     -
AOTVOKIR3DS4*04    : ----------------------------------------------------------------------------------------------- :     -
AOTVOKIR3DS4*04v1  : ----------------------------------------------------------------------------------------------- :     -
AOTVOKIR3DS5*01    : ----------------------------------------------------------------------------------------------- :     -
AOTVOKIR2DS5*01v1  : ----------------------------------------------------------------------------------------------- :     -
AOTVOKIR2DS5*01v2  : ----------------------------------------------------------------------------------------------- :     -
AOTVOKIR3DS7*01    : ----------------------------------------------------------------------------------------------- :     -
AOTVOKIR3DS7*01v1  : ----------------------------------------------------------------------------------------------- :     -
AOTVOKIR3DL8*01    : ----------------------------------------------------------------------------------------------- :     -
AOTVOKIR3DL8*01v1  : ----------------------------------------------------------------------------------------------- :     -
AOTVOKIR3DS8*01v2  : ----------------------------------------------------------------------------------------------- :     -
AOTVOKIR3DL8*02    : ----------------------------------------------------------------------------------------------- :     -
AOTVOKIR4DL9*01    : ----------------------------------------------------------------------------------------------- :     -
AOTVOKIR4DS9*01v1  : ----------------------------------------------------------------------------------------------- :     -
AOTVOKIR3DL9*01v2  : ----------------------------------------------------------------------------------------------- :     -
AOTVOKIR4DL9*02    : ----------------------------------------------------------------------------------------------- :     -
AOTVOKIR4DL10*01   : ----------------------------------------------------------------------------------------------- :     -
AOTVOKIR3DL10*01v1 : ----------------------------------------------------------------------------------------------- :     -
AOTVOKIR4DS10*01v2 : ----------------------------------------------------------------------------------------------- :     -
AOTVOKIR3DS10*01v3 : ----------------------------------------------------------------------------------------------- :     -
AOTVOKIR3DS10*01v4 : ----------------------------------------------------------------------------------------------- :     -

                              *      6480         *      6500         *      6520         *      6540         *
BAC_clone_Om       : GACCACATGTCATAACAGAGGACAGACATGGGGGCCATACAGGGAGTGAGAAAAGAGGGAAAGAGGTAGAGGGGACACACAGACAGACAGACATG :  6555
AOTVOKIR3DL4*01    : ----------------------------------------------------------------------------------------------- :     -
AOTVOKIR3DL4*02    : ----------------------------------------------------------------------------------------------- :     -
AOTVOKIR3DL4*02v1  : ----------------------------------------------------------------------------------------------- :     -
```

```
AOTVOKIR3DL4*02v2   : ----------------------------------------------------------------------------------------------- :     -
AOTVOKIR3DL4*03     : ----------------------------------------------------------------------------------------------- :     -
AOTVOKIR3DS4*04     : ----------------------------------------------------------------------------------------------- :     -
AOTVOKIR3DS4*04v1   : ----------------------------------------------------------------------------------------------- :     -
AOTVOKIR3DS5*01     : ----------------------------------------------------------------------------------------------- :     -
AOTVOKIR2DS5*01v1   : ----------------------------------------------------------------------------------------------- :     -
AOTVOKIR2DS5*01v2   : ----------------------------------------------------------------------------------------------- :     -
AOTVOKIR3DS7*01     : ----------------------------------------------------------------------------------------------- :     -
AOTVOKIR3DS7*01v1   : ----------------------------------------------------------------------------------------------- :     -
AOTVOKIR3DL8*01     : ----------------------------------------------------------------------------------------------- :     -
AOTVOKIR3DL8*01v1   : ----------------------------------------------------------------------------------------------- :     -
AOTVOKIR3DS8*01v2   : ----------------------------------------------------------------------------------------------- :     -
AOTVOKIR3DL8*02     : ----------------------------------------------------------------------------------------------- :     -
AOTVOKIR4DL9*01     : ----------------------------------------------------------------------------------------------- :     -
AOTVOKIR4DS9*01v1   : ----------------------------------------------------------------------------------------------- :     -
AOTVOKIR3DL9*01v2   : ----------------------------------------------------------------------------------------------- :     -
AOTVOKIR4DL9*02     : ----------------------------------------------------------------------------------------------- :     -
AOTVOKIR4DL10*01    : ----------------------------------------------------------------------------------------------- :     -
AOTVOKIR3DL10*01v1  : ----------------------------------------------------------------------------------------------- :     -
AOTVOKIR4DS10*01v2  : ----------------------------------------------------------------------------------------------- :     -
AOTVOKIR3DS10*01v3  : ----------------------------------------------------------------------------------------------- :     -
AOTVOKIR3DS10*01v4  : ----------------------------------------------------------------------------------------------- :     -

                         6560         *       6580         *       6600         *       6620         *       6640         *
BAC_clone_Om        : TCCCAGAGAGAGGTGTCCTTCCATGCTGGCTTTGTTCAGACACCTGGCACAGGGTAGAGGTTTTATTTCTGCATTTCCTCCACAAAGTGTTCTGT :  6650
AOTVOKIR3DL4*01     : ----------------------------------------------------------------------------------------------- :     -
AOTVOKIR3DL4*02     : ----------------------------------------------------------------------------------------------- :     -
AOTVOKIR3DL4*02v1   : ----------------------------------------------------------------------------------------------- :     -
AOTVOKIR3DL4*02v2   : ----------------------------------------------------------------------------------------------- :     -
AOTVOKIR3DL4*03     : ----------------------------------------------------------------------------------------------- :     -
AOTVOKIR3DS4*04     : ----------------------------------------------------------------------------------------------- :     -
AOTVOKIR3DS4*04v1   : ----------------------------------------------------------------------------------------------- :     -
AOTVOKIR3DS5*01     : ----------------------------------------------------------------------------------------------- :     -
AOTVOKIR2DS5*01v1   : ----------------------------------------------------------------------------------------------- :     -
AOTVOKIR2DS5*01v2   : ----------------------------------------------------------------------------------------------- :     -
AOTVOKIR3DS7*01     : ----------------------------------------------------------------------------------------------- :     -
AOTVOKIR3DS7*01v1   : ----------------------------------------------------------------------------------------------- :     -
AOTVOKIR3DL8*01     : ----------------------------------------------------------------------------------------------- :     -
AOTVOKIR3DL8*01v1   : ----------------------------------------------------------------------------------------------- :     -
AOTVOKIR3DS8*01v2   : ----------------------------------------------------------------------------------------------- :     -
AOTVOKIR3DL8*02     : ----------------------------------------------------------------------------------------------- :     -
AOTVOKIR4DL9*01     : ----------------------------------------------------------------------------------------------- :     -
AOTVOKIR4DS9*01v1   : ----------------------------------------------------------------------------------------------- :     -
AOTVOKIR3DL9*01v2   : ----------------------------------------------------------------------------------------------- :     -
AOTVOKIR4DL9*02     : ----------------------------------------------------------------------------------------------- :     -
```

```
AOTVOKIR4DL10*01    : ----------------------------------------------------------------------------------------------- :     -
AOTVOKIR3DL10*01v1  : ----------------------------------------------------------------------------------------------- :     -
AOTVOKIR4DS10*01v2  : ----------------------------------------------------------------------------------------------- :     -
AOTVOKIR3DS10*01v3  : ----------------------------------------------------------------------------------------------- :     -
AOTVOKIR3DS10*01v4  : ----------------------------------------------------------------------------------------------- :     -

                             6660        *       6680        *       6700        *       6720        *       6740
BAC_clone_Om        : ACCAGGAGAACCCAAAGACACCCAGATTTCTGACCTGAGTTGGGCCCTGTGGCCTCAGGCCTTATGGCACCTACAGATGCCGTGTTTATTCTGAC :  6745
AOTVOKIR3DL4*01     : ----------------------------------------------------------------------------------------------- :     -
AOTVOKIR3DL4*02     : ----------------------------------------------------------------------------------------------- :     -
AOTVOKIR3DL4*02v1   : ----------------------------------------------------------------------------------------------- :     -
AOTVOKIR3DL4*02v2   : ----------------------------------------------------------------------------------------------- :     -
AOTVOKIR3DL4*03     : ----------------------------------------------------------------------------------------------- :     -
AOTVOKIR3DS4*04     : ----------------------------------------------------------------------------------------------- :     -
AOTVOKIR3DS4*04v1   : ----------------------------------------------------------------------------------------------- :     -
AOTVOKIR3DS5*01     : ----------------------------------------------------------------------------------------------- :     -
AOTVOKIR2DS5*01v1   : ----------------------------------------------------------------------------------------------- :     -
AOTVOKIR2DS5*01v2   : ----------------------------------------------------------------------------------------------- :     -
AOTVOKIR3DS7*01     : ----------------------------------------------------------------------------------------------- :     -
AOTVOKIR3DS7*01v1   : ----------------------------------------------------------------------------------------------- :     -
AOTVOKIR3DL8*01     : ----------------------------------------------------------------------------------------------- :     -
AOTVOKIR3DL8*01v1   : ----------------------------------------------------------------------------------------------- :     -
AOTVOKIR3DS8*01v2   : ----------------------------------------------------------------------------------------------- :     -
AOTVOKIR3DL8*02     : ----------------------------------------------------------------------------------------------- :     -
AOTVOKIR4DL9*01     : ----------------------------------------------------------------------------------------------- :     -
AOTVOKIR4DS9*01v1   : ----------------------------------------------------------------------------------------------- :     -
AOTVOKIR3DL9*01v2   : ----------------------------------------------------------------------------------------------- :     -
AOTVOKIR4DL9*02     : ----------------------------------------------------------------------------------------------- :     -
AOTVOKIR4DL10*01    : ----------------------------------------------------------------------------------------------- :     -
AOTVOKIR3DL10*01v1  : ----------------------------------------------------------------------------------------------- :     -
AOTVOKIR4DS10*01v2  : ----------------------------------------------------------------------------------------------- :     -
AOTVOKIR3DS10*01v3  : ----------------------------------------------------------------------------------------------- :     -
AOTVOKIR3DS10*01v4  : ----------------------------------------------------------------------------------------------- :     -

                          *       6760        *       6780        *       6800        *       6820        *       6840
BAC_clone_Om        : ACCTCTGCCTTCCGTGCAGTGGAGCCGTAATCGTCCCAGGATATTATGGCCCCGGAACACCAACCCCAGTGTGCTGTGTGTACTTGGGGTCCCCA :  6840
AOTVOKIR3DL4*01     : ----------------------------------------------------------------------------------------------- :     -
AOTVOKIR3DL4*02     : ----------------------------------------------------------------------------------------------- :     -
AOTVOKIR3DL4*02v1   : ----------------------------------------------------------------------------------------------- :     -
AOTVOKIR3DL4*02v2   : ----------------------------------------------------------------------------------------------- :     -
AOTVOKIR3DL4*03     : ----------------------------------------------------------------------------------------------- :     -
AOTVOKIR3DS4*04     : ----------------------------------------------------------------------------------------------- :     -
AOTVOKIR3DS4*04v1   : ----------------------------------------------------------------------------------------------- :     -
AOTVOKIR3DS5*01     : ----------------------------------------------------------------------------------------------- :     -
```

```
AOTVOKIR2DS5*01v1  : ----------------------------------------------------------------------------------------------- :    -
AOTVOKIR2DS5*01v2  : ----------------------------------------------------------------------------------------------- :    -
AOTVOKIR3DS7*01    : ----------------------------------------------------------------------------------------------- :    -
AOTVOKIR3DS7*01v1  : ----------------------------------------------------------------------------------------------- :    -
AOTVOKIR3DL8*01    : ----------------------------------------------------------------------------------------------- :    -
AOTVOKIR3DL8*01v1  : ----------------------------------------------------------------------------------------------- :    -
AOTVOKIR3DS8*01v2  : ----------------------------------------------------------------------------------------------- :    -
AOTVOKIR3DL8*02    : ----------------------------------------------------------------------------------------------- :    -
AOTVOKIR4DL9*01    : ----------------------------------------------------------------------------------------------- :    -
AOTVOKIR4DS9*01v1  : ----------------------------------------------------------------------------------------------- :    -
AOTVOKIR3DL9*01v2  : ----------------------------------------------------------------------------------------------- :    -
AOTVOKIR4DL9*02    : ----------------------------------------------------------------------------------------------- :    -
AOTVOKIR4DL10*01   : ----------------------------------------------------------------------------------------------- :    -
AOTVOKIR3DL10*01v1 : ----------------------------------------------------------------------------------------------- :    -
AOTVOKIR4DS10*01v2 : ----------------------------------------------------------------------------------------------- :    -
AOTVOKIR3DS10*01v3 : ----------------------------------------------------------------------------------------------- :    -
AOTVOKIR3DS10*01v4 : ----------------------------------------------------------------------------------------------- :    -

                              *      6860         *      6880         *      6900         *      6920         *
BAC_clone_Om       : GACTGGATTCTGAGGCTCATATTCCAACTAATCTCACATACTGTAGCATCACTGAGAGACACAGAGAGAAATCAGGGACATCAGAATGCAAAGAT :  6935
AOTVOKIR3DL4*01    : ----------------------------------------------------------------------------------------------- :    -
AOTVOKIR3DL4*02    : ----------------------------------------------------------------------------------------------- :    -
AOTVOKIR3DL4*02v1  : ----------------------------------------------------------------------------------------------- :    -
AOTVOKIR3DL4*02v2  : ----------------------------------------------------------------------------------------------- :    -
AOTVOKIR3DL4*03    : ----------------------------------------------------------------------------------------------- :    -
AOTVOKIR3DS4*04    : ----------------------------------------------------------------------------------------------- :    -
AOTVOKIR3DS4*04v1  : ----------------------------------------------------------------------------------------------- :    -
AOTVOKIR3DS5*01    : ----------------------------------------------------------------------------------------------- :    -
AOTVOKIR2DS5*01v1  : ----------------------------------------------------------------------------------------------- :    -
AOTVOKIR2DS5*01v2  : ----------------------------------------------------------------------------------------------- :    -
AOTVOKIR3DS7*01    : ----------------------------------------------------------------------------------------------- :    -
AOTVOKIR3DS7*01v1  : ----------------------------------------------------------------------------------------------- :    -
AOTVOKIR3DL8*01    : ----------------------------------------------------------------------------------------------- :    -
AOTVOKIR3DL8*01v1  : ----------------------------------------------------------------------------------------------- :    -
AOTVOKIR3DS8*01v2  : ----------------------------------------------------------------------------------------------- :    -
AOTVOKIR3DL8*02    : ----------------------------------------------------------------------------------------------- :    -
AOTVOKIR4DL9*01    : ----------------------------------------------------------------------------------------------- :    -
AOTVOKIR4DS9*01v1  : ----------------------------------------------------------------------------------------------- :    -
AOTVOKIR3DL9*01v2  : ----------------------------------------------------------------------------------------------- :    -
AOTVOKIR4DL9*02    : ----------------------------------------------------------------------------------------------- :    -
AOTVOKIR4DL10*01   : ----------------------------------------------------------------------------------------------- :    -
AOTVOKIR3DL10*01v1 : ----------------------------------------------------------------------------------------------- :    -
AOTVOKIR4DS10*01v2 : ----------------------------------------------------------------------------------------------- :    -
AOTVOKIR3DS10*01v3 : ----------------------------------------------------------------------------------------------- :    -
AOTVOKIR3DS10*01v4 : ----------------------------------------------------------------------------------------------- :    -
```

```
                      6940         *       6960         *       6980         *       7000         *       7020         *
BAC_clone_Om       : ATAAACACACACAGAATGAGTCAGGGGAAGGGGATTGAGAAACTCACAGACACATAAAGAGAGAGAAAAGAGGGCAGAGAAGTGGAGGGAATGAT :  7030
AOTVOKIR3DL4*01    : ----------------------------------------------------------------------------------------------- :     -
AOTVOKIR3DL4*02    : ----------------------------------------------------------------------------------------------- :     -
AOTVOKIR3DL4*02v1  : ----------------------------------------------------------------------------------------------- :     -
AOTVOKIR3DL4*02v2  : ----------------------------------------------------------------------------------------------- :     -
AOTVOKIR3DL4*03    : ----------------------------------------------------------------------------------------------- :     -
AOTVOKIR3DS4*04    : ----------------------------------------------------------------------------------------------- :     -
AOTVOKIR3DS4*04v1  : ----------------------------------------------------------------------------------------------- :     -
AOTVOKIR3DS5*01    : ----------------------------------------------------------------------------------------------- :     -
AOTVOKIR2DS5*01v1  : ----------------------------------------------------------------------------------------------- :     -
AOTVOKIR2DS5*01v2  : ----------------------------------------------------------------------------------------------- :     -
AOTVOKIR3DS7*01    : ----------------------------------------------------------------------------------------------- :     -
AOTVOKIR3DS7*01v1  : ----------------------------------------------------------------------------------------------- :     -
AOTVOKIR3DL8*01    : ----------------------------------------------------------------------------------------------- :     -
AOTVOKIR3DL8*01v1  : ----------------------------------------------------------------------------------------------- :     -
AOTVOKIR3DS8*01v2  : ----------------------------------------------------------------------------------------------- :     -
AOTVOKIR3DL8*02    : ----------------------------------------------------------------------------------------------- :     -
AOTVOKIR4DL9*01    : ----------------------------------------------------------------------------------------------- :     -
AOTVOKIR4DS9*01v1  : ----------------------------------------------------------------------------------------------- :     -
AOTVOKIR3DL9*01v2  : ----------------------------------------------------------------------------------------------- :     -
AOTVOKIR4DL9*02    : ----------------------------------------------------------------------------------------------- :     -
AOTVOKIR4DL10*01   : ----------------------------------------------------------------------------------------------- :     -
AOTVOKIR3DL10*01v1 : ----------------------------------------------------------------------------------------------- :     -
AOTVOKIR4DS10*01v2 : ----------------------------------------------------------------------------------------------- :     -
AOTVOKIR3DS10*01v3 : ----------------------------------------------------------------------------------------------- :     -
AOTVOKIR3DS10*01v4 : ----------------------------------------------------------------------------------------------- :     -

                           7040         *       7060         *       7080         *       7100         *       7120
BAC_clone_Om       : GGAAGCGAGCAGAGAAATGCCCTAAAATCAGGGCCCTGAGGGAGGGACACAAGGGGAGAGAAAGATGGAGATGTGGGACGGATTGCAGAGAATCC :  7125
AOTVOKIR3DL4*01    : ----------------------------------------------------------------------------------------------- :     -
AOTVOKIR3DL4*02    : ----------------------------------------------------------------------------------------------- :     -
AOTVOKIR3DL4*02v1  : ----------------------------------------------------------------------------------------------- :     -
AOTVOKIR3DL4*02v2  : ----------------------------------------------------------------------------------------------- :     -
AOTVOKIR3DL4*03    : ----------------------------------------------------------------------------------------------- :     -
AOTVOKIR3DS4*04    : ----------------------------------------------------------------------------------------------- :     -
AOTVOKIR3DS4*04v1  : ----------------------------------------------------------------------------------------------- :     -
AOTVOKIR3DS5*01    : ----------------------------------------------------------------------------------------------- :     -
AOTVOKIR2DS5*01v1  : ----------------------------------------------------------------------------------------------- :     -
AOTVOKIR2DS5*01v2  : ----------------------------------------------------------------------------------------------- :     -
AOTVOKIR3DS7*01    : ----------------------------------------------------------------------------------------------- :     -
AOTVOKIR3DS7*01v1  : ----------------------------------------------------------------------------------------------- :     -
AOTVOKIR3DL8*01    : ----------------------------------------------------------------------------------------------- :     -
```

```
AOTVOKIR3DL8*01v1  : ----------------------------------------------------------------------------------------------- :    -
AOTVOKIR3DS8*01v2  : ----------------------------------------------------------------------------------------------- :    -
AOTVOKIR3DL8*02    : ----------------------------------------------------------------------------------------------- :    -
AOTVOKIR4DL9*01    : ----------------------------------------------------------------------------------------------- :    -
AOTVOKIR4DS9*01v1  : ----------------------------------------------------------------------------------------------- :    -
AOTVOKIR3DL9*01v2  : ----------------------------------------------------------------------------------------------- :    -
AOTVOKIR4DL9*02    : ----------------------------------------------------------------------------------------------- :    -
AOTVOKIR4DL10*01   : ----------------------------------------------------------------------------------------------- :    -
AOTVOKIR3DL10*01v1 : ----------------------------------------------------------------------------------------------- :    -
AOTVOKIR4DS10*01v2 : ----------------------------------------------------------------------------------------------- :    -
AOTVOKIR3DS10*01v3 : ----------------------------------------------------------------------------------------------- :    -
AOTVOKIR3DS10*01v4 : ----------------------------------------------------------------------------------------------- :    -

                         *       7140         *       7160         *       7180         *       7200         *       7220
BAC_clone_Om       : AAAGAGAACTAGAGAGACCGAGAGGCAGAGAAAGACAAGGAGGCGGAGACACAGATGATAGATGGATGGATAGAGATAGATAGATGATAAATAGG :  7220
AOTVOKIR3DL4*01    : ----------------------------------------------------------------------------------------------- :    -
AOTVOKIR3DL4*02    : ----------------------------------------------------------------------------------------------- :    -
AOTVOKIR3DL4*02v1  : ----------------------------------------------------------------------------------------------- :    -
AOTVOKIR3DL4*02v2  : ----------------------------------------------------------------------------------------------- :    -
AOTVOKIR3DL4*03    : ----------------------------------------------------------------------------------------------- :    -
AOTVOKIR3DS4*04    : ----------------------------------------------------------------------------------------------- :    -
AOTVOKIR3DS4*04v1  : ----------------------------------------------------------------------------------------------- :    -
AOTVOKIR3DS5*01    : ----------------------------------------------------------------------------------------------- :    -
AOTVOKIR2DS5*01v1  : ----------------------------------------------------------------------------------------------- :    -
AOTVOKIR2DS5*01v2  : ----------------------------------------------------------------------------------------------- :    -
AOTVOKIR3DS7*01    : ----------------------------------------------------------------------------------------------- :    -
AOTVOKIR3DS7*01v1  : ----------------------------------------------------------------------------------------------- :    -
AOTVOKIR3DL8*01    : ----------------------------------------------------------------------------------------------- :    -
AOTVOKIR3DL8*01v1  : ----------------------------------------------------------------------------------------------- :    -
AOTVOKIR3DS8*01v2  : ----------------------------------------------------------------------------------------------- :    -
AOTVOKIR3DL8*02    : ----------------------------------------------------------------------------------------------- :    -
AOTVOKIR4DL9*01    : ----------------------------------------------------------------------------------------------- :    -
AOTVOKIR4DS9*01v1  : ----------------------------------------------------------------------------------------------- :    -
AOTVOKIR3DL9*01v2  : ----------------------------------------------------------------------------------------------- :    -
AOTVOKIR4DL9*02    : ----------------------------------------------------------------------------------------------- :    -
AOTVOKIR4DL10*01   : ----------------------------------------------------------------------------------------------- :    -
AOTVOKIR3DL10*01v1 : ----------------------------------------------------------------------------------------------- :    -
AOTVOKIR4DS10*01v2 : ----------------------------------------------------------------------------------------------- :    -
AOTVOKIR3DS10*01v3 : ----------------------------------------------------------------------------------------------- :    -
AOTVOKIR3DS10*01v4 : ----------------------------------------------------------------------------------------------- :    -

                              *       7240         *       7260         *       7280         *       7300         *
BAC_clone_Om       : TAGATGGTAGATCATAGAGAGGTTATAGATACATAGATGATGATTGATTGACAGATGATACATAGAGATGATGATGAAGATAGAGAGAGATAATA :  7315
AOTVOKIR3DL4*01    : ----------------------------------------------------------------------------------------------- :    -
```

```
AOTVOKIR3DL4*02     : ----------------------------------------------------------------------------------------------- :    -
AOTVOKIR3DL4*02v1   : ----------------------------------------------------------------------------------------------- :    -
AOTVOKIR3DL4*02v2   : ----------------------------------------------------------------------------------------------- :    -
AOTVOKIR3DL4*03     : ----------------------------------------------------------------------------------------------- :    -
AOTVOKIR3DS4*04     : ----------------------------------------------------------------------------------------------- :    -
AOTVOKIR3DS4*04v1   : ----------------------------------------------------------------------------------------------- :    -
AOTVOKIR3DS5*01     : ----------------------------------------------------------------------------------------------- :    -
AOTVOKIR2DS5*01v1   : ----------------------------------------------------------------------------------------------- :    -
AOTVOKIR2DS5*01v2   : ----------------------------------------------------------------------------------------------- :    -
AOTVOKIR3DS7*01     : ----------------------------------------------------------------------------------------------- :    -
AOTVOKIR3DS7*01v1   : ----------------------------------------------------------------------------------------------- :    -
AOTVOKIR3DL8*01     : ----------------------------------------------------------------------------------------------- :    -
AOTVOKIR3DL8*01v1   : ----------------------------------------------------------------------------------------------- :    -
AOTVOKIR3DS8*01v2   : ----------------------------------------------------------------------------------------------- :    -
AOTVOKIR3DL8*02     : ----------------------------------------------------------------------------------------------- :    -
AOTVOKIR4DL9*01     : ----------------------------------------------------------------------------------------------- :    -
AOTVOKIR4DS9*01v1   : ----------------------------------------------------------------------------------------------- :    -
AOTVOKIR3DL9*01v2   : ----------------------------------------------------------------------------------------------- :    -
AOTVOKIR4DL9*02     : ----------------------------------------------------------------------------------------------- :    -
AOTVOKIR4DL10*01    : ----------------------------------------------------------------------------------------------- :    -
AOTVOKIR3DL10*01v1  : ----------------------------------------------------------------------------------------------- :    -
AOTVOKIR4DS10*01v2  : ----------------------------------------------------------------------------------------------- :    -
AOTVOKIR3DS10*01v3  : ----------------------------------------------------------------------------------------------- :    -
AOTVOKIR3DS10*01v4  : ----------------------------------------------------------------------------------------------- :    -

                          7320         *       7340         *       7360         *       7380         *       7400         *
BAC_clone_Om        : GAGATGATACATAGATATGAATAATAGATGATTGATGCTCAGAAAGACAGACAGTTGATAGAGGCATAGATAGATGATACATAGCTACAGATGAT :  7410
AOTVOKIR3DL4*01     : ----------------------------------------------------------------------------------------------- :    -
AOTVOKIR3DL4*02     : ----------------------------------------------------------------------------------------------- :    -
AOTVOKIR3DL4*02v1   : ----------------------------------------------------------------------------------------------- :    -
AOTVOKIR3DL4*02v2   : ----------------------------------------------------------------------------------------------- :    -
AOTVOKIR3DL4*03     : ----------------------------------------------------------------------------------------------- :    -
AOTVOKIR3DS4*04     : ----------------------------------------------------------------------------------------------- :    -
AOTVOKIR3DS4*04v1   : ----------------------------------------------------------------------------------------------- :    -
AOTVOKIR3DS5*01     : ----------------------------------------------------------------------------------------------- :    -
AOTVOKIR2DS5*01v1   : ----------------------------------------------------------------------------------------------- :    -
AOTVOKIR2DS5*01v2   : ----------------------------------------------------------------------------------------------- :    -
AOTVOKIR3DS7*01     : ----------------------------------------------------------------------------------------------- :    -
AOTVOKIR3DS7*01v1   : ----------------------------------------------------------------------------------------------- :    -
AOTVOKIR3DL8*01     : ----------------------------------------------------------------------------------------------- :    -
AOTVOKIR3DL8*01v1   : ----------------------------------------------------------------------------------------------- :    -
AOTVOKIR3DS8*01v2   : ----------------------------------------------------------------------------------------------- :    -
AOTVOKIR3DL8*02     : ----------------------------------------------------------------------------------------------- :    -
AOTVOKIR4DL9*01     : ----------------------------------------------------------------------------------------------- :    -
AOTVOKIR4DS9*01v1   : ----------------------------------------------------------------------------------------------- :    -
```

```
AOTVOKIR3DL9*01v2  : ----------------------------------------------------------------------------------------------- :     -
AOTVOKIR4DL9*02    : ----------------------------------------------------------------------------------------------- :     -
AOTVOKIR4DL10*01   : ----------------------------------------------------------------------------------------------- :     -
AOTVOKIR3DL10*01v1 : ----------------------------------------------------------------------------------------------- :     -
AOTVOKIR4DS10*01v2 : ----------------------------------------------------------------------------------------------- :     -
AOTVOKIR3DS10*01v3 : ----------------------------------------------------------------------------------------------- :     -
AOTVOKIR3DS10*01v4 : ----------------------------------------------------------------------------------------------- :     -

                              7420        *       7440        *       7460        *       7480        *       7500
BAC_clone_Om       : AGATCATTTGTAGACACCAAATACAGAAATAGAGACATACATAATGGTTAGAAATAGGCAGAAAGTTATGAACAAGACACAAAGTGAGAGACTCA :  7505
AOTVOKIR3DL4*01    : ----------------------------------------------------------------------------------------------- :     -
AOTVOKIR3DL4*02    : ----------------------------------------------------------------------------------------------- :     -
AOTVOKIR3DL4*02v1  : ----------------------------------------------------------------------------------------------- :     -
AOTVOKIR3DL4*02v2  : ----------------------------------------------------------------------------------------------- :     -
AOTVOKIR3DL4*03    : ----------------------------------------------------------------------------------------------- :     -
AOTVOKIR3DS4*04    : ----------------------------------------------------------------------------------------------- :     -
AOTVOKIR3DS4*04v1  : ----------------------------------------------------------------------------------------------- :     -
AOTVOKIR3DS5*01    : ----------------------------------------------------------------------------------------------- :     -
AOTVOKIR2DS5*01v1  : ----------------------------------------------------------------------------------------------- :     -
AOTVOKIR2DS5*01v2  : ----------------------------------------------------------------------------------------------- :     -
AOTVOKIR3DS7*01    : ----------------------------------------------------------------------------------------------- :     -
AOTVOKIR3DS7*01v1  : ----------------------------------------------------------------------------------------------- :     -
AOTVOKIR3DL8*01    : ----------------------------------------------------------------------------------------------- :     -
AOTVOKIR3DL8*01v1  : ----------------------------------------------------------------------------------------------- :     -
AOTVOKIR3DS8*01v2  : ----------------------------------------------------------------------------------------------- :     -
AOTVOKIR3DL8*02    : ----------------------------------------------------------------------------------------------- :     -
AOTVOKIR4DL9*01    : ----------------------------------------------------------------------------------------------- :     -
AOTVOKIR4DS9*01v1  : ----------------------------------------------------------------------------------------------- :     -
AOTVOKIR3DL9*01v2  : ----------------------------------------------------------------------------------------------- :     -
AOTVOKIR4DL9*02    : ----------------------------------------------------------------------------------------------- :     -
AOTVOKIR4DL10*01   : ----------------------------------------------------------------------------------------------- :     -
AOTVOKIR3DL10*01v1 : ----------------------------------------------------------------------------------------------- :     -
AOTVOKIR4DS10*01v2 : ----------------------------------------------------------------------------------------------- :     -
AOTVOKIR3DS10*01v3 : ----------------------------------------------------------------------------------------------- :     -
AOTVOKIR3DS10*01v4 : ----------------------------------------------------------------------------------------------- :     -

                         *       7520        *       7540        *       7560        *       7580        *       7600
BAC_clone_Om       : GAATTCAAGGAAAAAGATCAACCAATCCAAGTCGAGTCAGAGAGAATAAAACAATAGAAAAAACGGAAAACCTACCCGGGGTGGGGAAGTGAGGG :  7600
AOTVOKIR3DL4*01    : ----------------------------------------------------------------------------------------------- :     -
AOTVOKIR3DL4*02    : ----------------------------------------------------------------------------------------------- :     -
AOTVOKIR3DL4*02v1  : ----------------------------------------------------------------------------------------------- :     -
AOTVOKIR3DL4*02v2  : ----------------------------------------------------------------------------------------------- :     -
AOTVOKIR3DL4*03    : ----------------------------------------------------------------------------------------------- :     -
AOTVOKIR3DS4*04    : ----------------------------------------------------------------------------------------------- :     -
```

```
AOTVOKIR3DS4*04v1  : ----------------------------------------------------------------------------------------------- :     -
AOTVOKIR3DS5*01    : ----------------------------------------------------------------------------------------------- :     -
AOTVOKIR2DS5*01v1  : ----------------------------------------------------------------------------------------------- :     -
AOTVOKIR2DS5*01v2  : ----------------------------------------------------------------------------------------------- :     -
AOTVOKIR3DS7*01    : ----------------------------------------------------------------------------------------------- :     -
AOTVOKIR3DS7*01v1  : ----------------------------------------------------------------------------------------------- :     -
AOTVOKIR3DL8*01    : ----------------------------------------------------------------------------------------------- :     -
AOTVOKIR3DL8*01v1  : ----------------------------------------------------------------------------------------------- :     -
AOTVOKIR3DS8*01v2  : ----------------------------------------------------------------------------------------------- :     -
AOTVOKIR3DL8*02    : ----------------------------------------------------------------------------------------------- :     -
AOTVOKIR4DL9*01    : ----------------------------------------------------------------------------------------------- :     -
AOTVOKIR4DS9*01v1  : ----------------------------------------------------------------------------------------------- :     -
AOTVOKIR3DL9*01v2  : ----------------------------------------------------------------------------------------------- :     -
AOTVOKIR4DL9*02    : ----------------------------------------------------------------------------------------------- :     -
AOTVOKIR4DL10*01   : ----------------------------------------------------------------------------------------------- :     -
AOTVOKIR3DL10*01v1 : ----------------------------------------------------------------------------------------------- :     -
AOTVOKIR4DS10*01v2 : ----------------------------------------------------------------------------------------------- :     -
AOTVOKIR3DS10*01v3 : ----------------------------------------------------------------------------------------------- :     -
AOTVOKIR3DS10*01v4 : ----------------------------------------------------------------------------------------------- :     -

                              *      7620         *      7640         *      7660         *      7680         *
BAC_clone_Om       : GAGAGACAGGGAAGGTAGAAGGAGAAAACAGACAGAAGAGAGACGGGGTGGAGGGTGAGAGAGAGCATCAGCTCATGGAGCAGGGGAGTGAGTTC :  7695
AOTVOKIR3DL4*01    : ----------------------------------------------------------------------------------------------- :     -
AOTVOKIR3DL4*02    : ----------------------------------------------------------------------------------------------- :     -
AOTVOKIR3DL4*02v1  : ----------------------------------------------------------------------------------------------- :     -
AOTVOKIR3DL4*02v2  : ----------------------------------------------------------------------------------------------- :     -
AOTVOKIR3DL4*03    : ----------------------------------------------------------------------------------------------- :     -
AOTVOKIR3DS4*04    : ----------------------------------------------------------------------------------------------- :     -
AOTVOKIR3DS4*04v1  : ----------------------------------------------------------------------------------------------- :     -
AOTVOKIR3DS5*01    : ----------------------------------------------------------------------------------------------- :     -
AOTVOKIR2DS5*01v1  : ----------------------------------------------------------------------------------------------- :     -
AOTVOKIR2DS5*01v2  : ----------------------------------------------------------------------------------------------- :     -
AOTVOKIR3DS7*01    : ----------------------------------------------------------------------------------------------- :     -
AOTVOKIR3DS7*01v1  : ----------------------------------------------------------------------------------------------- :     -
AOTVOKIR3DL8*01    : ----------------------------------------------------------------------------------------------- :     -
AOTVOKIR3DL8*01v1  : ----------------------------------------------------------------------------------------------- :     -
AOTVOKIR3DS8*01v2  : ----------------------------------------------------------------------------------------------- :     -
AOTVOKIR3DL8*02    : ----------------------------------------------------------------------------------------------- :     -
AOTVOKIR4DL9*01    : ----------------------------------------------------------------------------------------------- :     -
AOTVOKIR4DS9*01v1  : ----------------------------------------------------------------------------------------------- :     -
AOTVOKIR3DL9*01v2  : ----------------------------------------------------------------------------------------------- :     -
AOTVOKIR4DL9*02    : ----------------------------------------------------------------------------------------------- :     -
AOTVOKIR4DL10*01   : ----------------------------------------------------------------------------------------------- :     -
AOTVOKIR3DL10*01v1 : ----------------------------------------------------------------------------------------------- :     -
AOTVOKIR4DS10*01v2 : ----------------------------------------------------------------------------------------------- :     -
```

```
AOTVOKIR3DS10*01v3 : ---------------------------------------------------------------------------------------------- :     -
AOTVOKIR3DS10*01v4 : ---------------------------------------------------------------------------------------------- :     -

                                                                                                       EXON 5
                      7700         *       7720         *       7740         *       7760        *       7780         *
BAC_clone_Om       : TCAGCTCAGGTGTGAAGGGAGCTGTGACAAGGAAGAGCCTCCTGAGCAAACTGCCTCTTCTCCTTCCAGGTCTATATGATAAACCTTCTCTCTCA :  7790
AOTVOKIR3DL4*01    : --------------------------------------------------------------------GTCGATATGAGAAACCTTCTCTCTCA :   678
AOTVOKIR3DL4*02    : --------------------------------------------------------------------GTCGATATGAGAAACCTTCTCTCTCA :   678
AOTVOKIR3DL4*02v1  : --------------------------------------------------------------------GTCGATATGAGAAACCTTCTCTCTCA :   642
AOTVOKIR3DL4*02v2  : --------------------------------------------------------------------GTCGATATGAGAAACCTTCTCTCTCA :   678
AOTVOKIR3DL4*03    : --------------------------------------------------------------------GTCGATATGAGAAACCTTCTCTCTCA :   678
AOTVOKIR3DS4*04    : --------------------------------------------------------------------GTCGATATGAGAAACCTTCTCTCTCA :   678
AOTVOKIR3DS4*04v1  : --------------------------------------------------------------------GTCGATATGAGAAACCTTCTCTCTCA :   678
AOTVOKIR3DS5*01    : --------------------------------------------------------------------GTCTATATGAGAAACCTTCTCTCTCA :   678
AOTVOKIR2DS5*01v1  : --------------------------------------------------------------------GTCTATATGAGAAACCTTCTCTCTCA :   480
AOTVOKIR2DS5*01v2  : --------------------------------------------------------------------GTCTATATGAGAAACCTTCTCTCTCA :   393
AOTVOKIR3DS7*01    : --------------------------------------------------------------------GTCGATATGACAAACCTTCTCTCTCA :   678
AOTVOKIR3DS7*01v1  : --------------------------------------------------------------------GTCGATATGACAAACCTTCTCTCTCA :   678
AOTVOKIR3DL8*01    : --------------------------------------------------------------------GTCTATATGAGAAACCTTCTCTCTCA :   678
AOTVOKIR3DL8*01v1  : --------------------------------------------------------------------GTCTATATGAGAAACCTTCTCTCTCA :   678
AOTVOKIR3DS8*01v2  : --------------------------------------------------------------------GTCTATATGAGAAACCTTCTCTCTCA :   678
AOTVOKIR3DL8*02    : --------------------------------------------------------------------GTCTATATGAGAAACCTTCTCTCTCA :   678
AOTVOKIR4DL9*01    : --------------------------------------------------------------------GTCGATATGAGAAGCCTTCTCTCTCA :   963
AOTVOKIR4DS9*01v1  : --------------------------------------------------------------------GTCGATATGAGAAGCCTTCTCTCTCA :   963
AOTVOKIR3DL9*01v2  : --------------------------------------------------------------------GTCGATATGAGAAGCCTTCTCTCTCA :   765
AOTVOKIR4DL9*02    : --------------------------------------------------------------------GTCGATATGAGAAGCCTTCTCTCTCA :   963
AOTVOKIR4DL10*01   : --------------------------------------------------------------------GTCGATATGAGAAACCTTCTCTCTCA :   963
AOTVOKIR3DL10*01v1 : --------------------------------------------------------------------GTCGATATGAGAAACCTTCTCTCTCA :   762
AOTVOKIR4DS10*01v2 : --------------------------------------------------------------------GTCGATATGAGAAACCTTCTCTCTCA :   963
AOTVOKIR3DS10*01v3 : --------------------------------------------------------------------GTCGATATGAGAAACCTTCTCTCTCA :   678
AOTVOKIR3DS10*01v4 : --------------------------------------------------------------------GTCGATATGAGAAACCTTCTCTCTCA :   678
                                                                                         GTC ATATGAgAAaCCTTCTCTCTCA

                          7800         *       7820         *       7840         *       7860         *       7880
BAC_clone_Om       : GCCCGGCCGGGCCCCACGGTTCAGGCAGGAGAGAACGTGACCTTGTCCTGCAGCTCCCGGACCTGGTTCGACATGTACCATCTATCCAGGGAGGG :  7885
AOTVOKIR3DL4*01    : GCCCAGCCGGGCCCCACGGTTCAGGCAGGAGAGAATGTGACCTTGTCCTGCAGCTCCCGGACCTGGTTTGACATGTACCATCTAACCAGGGAGGG :   773
AOTVOKIR3DL4*02    : GCCCAGCCGGGCCCCACGGTTCAGGCAGGAGAGAATGTGACCTTGTCCTGCAGCTCCCGGACCTGGTTTGACATGTACCATCTAACCAGGGAGGG :   773
AOTVOKIR3DL4*02v1  : GCCCAGCCGGGCCCCACGGTTCAGGCAGGAGAGAATGTGACCTTGTCCTGCAGCTCCCGGACCTGGTTTGACATGTACCATCTAACCAGGGAGGG :   737
AOTVOKIR3DL4*02v2  : GCCCAGCCGGGCCCCACGGTTCAGGCAGGAGAGAATGTGACCTTGTCCTGCAGCTCCCGGACCTGGTTTGACATGTACCATCTAACCAGGGAGGG :   773
AOTVOKIR3DL4*03    : GCCCAGCCGGGCCCCACGGTTCAGGCAGGAGAGAATGTGACCTTGTCCTGCAGCTCCCGGACCTGGTTTGACATGTACCATCTAACCAGGGAGGG :   773
AOTVOKIR3DS4*04    : GCCCAGCCGGGCCCCACGGTTCAGGCAGGAGAGAATGTGACCTTGTCCTGCAGCTCCCGGACCTGGTTTGACATGTACCATCTAACCAGGGAGGG :   773
AOTVOKIR3DS4*04v1  : GCCCAGCCGGGCCCCACGGTTCAGGCAGGAGAGAATGTGACCTTGTCCTGCAGCTCCCGGACCTGGTTTGACATGTACCATCTAACCAGGGAGGG :   773
AOTVOKIR3DS5*01    : GCCCAGCCGGGCCCCACGCTTCAGCCAGGAGAGAATGTGACCTTGTCCTGCAGCTCCCGGACCTGGTTCGACATGTACCATCTATCCAGGGAAGG :   773
AOTVOKIR2DS5*01v1  : GCCCAGCCGGGCCCCACGCTTCAGCCAGGAGAGAATGTGACCTTGTCCTGCAGCTCCCGGACCTGGTTCGACATGTACCATCTATCCAGGGAAGG :   575
AOTVOKIR2DS5*01v2  : GCCCAGCCGGGCCCCACGCTTCAGCCAGGAGAGAATGTGACCTTGTCCTGCAGCTCCCGGACCTGGTTCGACATGTACCATCTATCCAGGGAAGG :   488
AOTVOKIR3DS7*01    : GCCCAGCCAGGCCCCACGGTTCAGGCAGGAGAGAATGTGACCTTGTCCTGCAGCTCCCGGAGCTGGTTCGACATGTACCATCTAACTAGGGAGGG :   773
```

```
AOTVOKIR3DS7*01v1  : GCCCAGCCAGGCCCCACGGTTCAGGCAGGAGAGAATGTGACCTTGTCCTGCAGCTCCCGGAGCTGGTTCGACATGTACCATCTAACTAGGGAGGG :  773
AOTVOKIR3DL8*01    : GCCCGGTCGGGCCCCACGGTTCAGGCAGGAGAGAATGTGACCTTGTCCTGCAGCTCCCGGAGCTGGTTCGACATGTACCATCTATCCAGGGAGGG :  773
AOTVOKIR3DL8*01v1  : GCCCGGTCGGGCCCCACGGTTCAGGCAGGAGAGAATGTGACCTTGTCCTGCAGCTCCCGGAGCTGGTTCGACATGTACCATCTATCCAGGGAGGG :  773
AOTVOKIR3DS8*01v2  : GCCCGGTCGGGCCCCACGGTTCAGGCAGGAGAGAATGTGACCTTGTCCTGCAGCTCCCGGAGCTGGTTCGACATGTACCATCTATCCAGGGAGGG :  773
AOTVOKIR3DL8*02    : GCCCGGTCGGGCCCCACGGTTCAGGCAGGAGAGAATGTGACCTTGTCCTGCAGCTCCCGGAGCTGGTTCGACATGTACCATCTATCCAGGGAGGG :  773
AOTVOKIR4DL9*01    : GCCCAGCCGGGCCCCACGGTTCAGCCAGGAGAGAATGTGACCTTGTCCTGCAGCTCCCGGACCTTGTTTCAGCTGTACCATCTATCCAGGGATGG : 1058
AOTVOKIR4DS9*01v1  : GCCCAGCCGGGCCCCACGGTTCAGCCAGGAGAGAATGTGACCTTGTCCTGCAGCTCCCGGACCTTGTTTCAGCTGTACCATCTATCCAGGGATGG : 1058
AOTVOKIR3DL9*01v2  : GCCCAGCCGGGCCCCACGGTTCAGCCAGGAGAGAATGTGACCTTGTCCTGCAGCTCCCGGACCTTGTTTCAGCTGTACCATCTATCCAGGGATGG :  860
AOTVOKIR4DL9*02    : GCCCAGCCGGGCCCCACGGTTCAGCCAGGAGAGAATGTGACCTTGTCATGCAGCTCCCGGACCTCGTATCAGTTGTACCATCTATCCAGTGATGG : 1058
AOTVOKIR4DL10*01   : GCCCAGCCGGGCCCCACGCTTCAGGCAGGAGAGAATGTGACCTTGTCCTGCAGCTCCCGGACCTTGTTTCAGCTGTACCATCTATCCAGGGAGGG : 1058
AOTVOKIR3DL10*01v1 : GCCCAGCCGGGCCCCACGCTTCAGGCAGGAGAGAATGTGACCTTGTCCTGCAGCTCCCGGACCTTGTTTCAGCTGTACCATCTATCCAGGGAGGG :  857
AOTVOKIR4DS10*01v2 : GCCCAGCCGGGCCCCACGCTTCAGGCAGGAGAGAATGTGACCTTGTCCTGCAGCTCCCGGACCTTGTTTCAGCTGTACCATCTATCCAGGGAGGG : 1058
AOTVOKIR3DS10*01v3 : GCCCAGCCGGGCCCCACGCTTCAGGCAGGAGAGAATGTGACCTTGTCCTGCAGCTCCCGGACCTTGTTTCAGCTGTACCATCTATCCAGGGAGGG :  773
AOTVOKIR3DS10*01v4 : GCCCAGCCGGGCCCCACGCTTCAGGCAGGAGAGAATGTGACCTTGTCCTGCAGCTCCCGGACCTTGTTTCAGCTGTACCATCTATCCAGGGAGGG :  773
                     GCCCaGcCgGGCCCCACG TTCAG CAGGAGAGAAtGTGACCTTGTCcTGCAGCTCCCGGA CT GTt  A  TGTACCATCTA CcAGgGA GG

                          *        7900         *        7920         *        7940         *        7960         *        7980
BAC_clone_Om       : GGAGGCCCCTGAACTCAGGCTCCCTGCAGTGCCCAGCATCAATGGAACATTCCAGGCCCACTTCCCTCTGGGCCCTGCCACCCACGGAGGGACCT : 7980
AOTVOKIR3DL4*01    : GGAGGCCCCTGAATTCAGGCTCCCTGCAGTGCCAGGCATCAATGGAACGTTCCAGGCCCACTTCCCTCTGGGCCCTGCCACCCATGGAGGGACCT :  868
AOTVOKIR3DL4*02    : GGAGGCCCCTGAATTCAGGCTCCCTGCAGTGCCGGGCATCAATGGAACGTTCCAGACCCACTTCCCTCTGGGCCCTGCCACCCATGGAGGGACCT :  868
AOTVOKIR3DL4*02v1  : GGAGGCCCCTGAATTCAGGCTCCCTGCAGTGCCGGGCATCAATGGAACGTTCCAGACCCACTTCCCTCTGGGCCCTGCCACCCATGGAGGGACCT :  832
AOTVOKIR3DL4*02v2  : GGAGGCCCCTGAATTCAGGCTCCCTGCAGTGCCGGGCATCAATGGAACGTTCCAGACCCACTTCCCTCTGGGCCCTGCCACCCATGGAGGGACCT :  868
AOTVOKIR3DL4*03    : GGAGGCCCCTGAATTCAGGCTCCCTGCAGTGCCAGGCATCAATGGAACGTTCCAGACCCACTTCCCTCTGGGCCCTGCCACCCATGGAGGGACCT :  868
AOTVOKIR3DS4*04    : AGAGGCCCCTGAATTCAGGCTCCCTGCAGTGCCGGGCATCAATGGAACGTTCCAGGCCCACTTCCCTCTGGGCCCTGCCACCCATGGAGGGACCT :  868
AOTVOKIR3DS4*04v1  : AGAGGCCCCTGAATTCAGGCTCCCTGCAGTGCCGGGCATCAATGGAACGTTCCAGGCCCACTTCCCTCTGGGCCCTGCCACCCATGGAGGGACCT :  868
AOTVOKIR3DS5*01    : GGAGGCCCCTGAACTCAGGCTCCCTGCAGTGCCCAGCATCAATGGAACATTCCAGGCCCACTTCCCTCTGGGCCCTGCCACCCACGGAGGGACCT :  868
AOTVOKIR2DS5*01v1  : GGAGGCCCCTGAACTCAGGCTCCCTGCAGTGCCCAGCATCAATGGAACATTCCAGGCCCACTTCCCTCTGGGCCCTGCCACCCACGGAGGGACCT :  670
AOTVOKIR2DS5*01v2  : GGAGGCCCCTGAACTCAGGCTCCCTGCAGTGCCCAGCATCAATGGAACATTCCAGGCCCACTTCCCTCTGGGCCCTGCCACCCACGGAGGGACCT :  583
AOTVOKIR3DS7*01    : GGAGGCCCCTGAACTCAAGCTCCCTGCAGTGCCCATCATCAGTGGAACATTCCAGGCCCACTTCCCTCTGGGCCCTGCCACCCACGGAGGGACCT :  868
AOTVOKIR3DS7*01v1  : GGAGGCCCCTGAACTCAAGCTCCCTGCAGTGCCCATCATCAGTGGAACATTCCAGGCCCACTTCCCTCTGGGCCCTGCCACCCACGGAGGGACCT :  868
AOTVOKIR3DL8*01    : GAAGGCCCCTGAACTCAGGCTCCCTGCAGTGCCCGGCATCAATGGAACATTCCAGGCCCACTTCCCTCTGGGCCCTGCCACCCACGGAGGGACCT :  868
AOTVOKIR3DL8*01v1  : GAAGGCCCCTGAACTCAGGCTCCCTGCAGTGCCCGGCATCAATGGAACATTCCAGGCCCACTTCCCTCTGGGCCCTGCCACCCACGGAGGGACCT :  868
AOTVOKIR3DS8*01v2  : GAAGGCCCCTGAACTCAGGCTCCCTGCAGTGCCCGGCATCAATGGAACATTCCAGGCCCACTTCCCTCTGGGCCCTGCCACCCACGGAGGGACCT :  868
AOTVOKIR3DL8*02    : GAAGGCCCCTGAACTCAGGCTCCCTGCAGTGCCCGGCATCAATGGAACATTCCAGGCCCACTTCCCTCTGGGCCCTGCCACCCACGGAGGGACCT :  868
AOTVOKIR4DL9*01    : GGAGGCCCCTGAACTCAGGCTCCCTGCAGTGCCCAGCATCAATGGAACATTCCAGGCCCACTTCCCTCTGGGCCCTGCCACCCACGGAGGGACCT : 1153
AOTVOKIR4DS9*01v1  : GGAGGCCCCTGAACTCAGGCTCCCTGCAGTGCCCAGCATCAATGGAACATTCCAGGCCCACTTCCCTCTGGGCCCTGCCACCCACGGAGGGACCT : 1153
AOTVOKIR3DL9*01v2  : GGAGGCCCCTGAACTCAGGCTCCCTGCAGTGCCCAGCATCAATGGAACATTCCAGGCCCACTTCCCTCTGGGCCCTGCCACCCACGGAGGGACCT :  955
AOTVOKIR4DL9*02    : GGAGACCCGTCAATTCAGACTCCCTGCAGTGCCCAGCATCAATGGAACATTCCAGGCCCACTTCCCTCTGGGCCCTGCCACCCACGGAGGGACCT : 1153
AOTVOKIR4DL10*01   : GGAGGCCCCTGAACTCAGGCTCCCTGCAGTGCCCAGCATCAATGGAACATTCCAGGCCCACTTCCCTCTGGGCCCTGCCACCCACGGAGGGACCT : 1153
AOTVOKIR3DL10*01v1 : GGAGGCCCCTGAACTCAGGCTCCCTGCAGTGCCCAGCATCAATGGAACATTCCAGGCCCACTTCCCTCTGGGCCCTGCCACCCACGGAGGGACCT :  952
AOTVOKIR4DS10*01v2 : GGAGGCCCCTGAACTCAGGCTCCCTGCAGTGCCCAGCATCAATGGAACATTCCAGGCCCACTTCCCTCTGGGCCCTGCCACCCACGGAGGGACCT : 1153
AOTVOKIR3DS10*01v3 : GGAGGCCCCTGAACTCAGGCTCCCTGCAGTGCCCAGCATCAATGGAACATTCCAGGCCCACTTCCCTCTGGGCCCTGCCACCCACGGAGGGACCT :  868
AOTVOKIR3DS10*01v4 : GGAGGCCCCTGAACTCAGGCTCCCTGCAGTGCCCAGCATCAATGGAACATTCCAGGCCCACTTCCCTCTGGGCCCTGCCACCCACGGAGGGACCT :  868
                     ggAGgCCCcTgAA TCAggCTCCCTGCAGTGCC  gCATCAaTGGAAC TTCCAGgCCCACTTCCCTCTGGGCCCTGCCACCCA GGAGGGACCT

                              *        8000         *        8020         *        8040         *        8060         *
```

```
BAC_clone_Om          : ACAGATGCTTCGGCTCTTTCCGTGACTCTCCCTATGAGTGGTCAGCCCCGAGTGACCCACTGTCCGTTTCTGTCACAGGTGAGGAAACCCCATAC : 8075
AOTVOKIR3DL4*01       : ACAGATGCTTCGGCTCTTTCCGTGACTCTCCCTATGAGTGGTCAGCCCCGAGTGACCCACTGTCCATTTCTGTCACAG----------------- :  946
AOTVOKIR3DL4*02       : ACAGATGCTTCGGCTCTTTCCGTGACTCTCCCTATGAGTGGTCAGCCCCGAGTGACCCACTGTCCATTTCTGTCACAG----------------- :  946
AOTVOKIR3DL4*02v1     : ACAGATGCTTCGGCTCTTTCCGTGACTCTCCCTATGAGTGGTCAGCCCCGAGTGACCCACTGTCCATTTCTGTCACAG----------------- :  910
AOTVOKIR3DL4*02v2     : ACAGATGCTTCGGCTCTTTCCGTGACTCTCCCTATGAGTGGTCAGCCCCGAGTGACCCACTGTCCATTTCTGTCACAG----------------- :  946
AOTVOKIR3DL4*03       : ACAGATGCTTCGGCTCTTTCCGTGACTCTCCCTATGAGTGGTCAGCCCCGAGTGACCCACTGTCCATTTCTGTCACAG----------------- :  946
AOTVOKIR3DS4*04       : ACAAATGCTTCGGCTCTTTCCGTGACTCTCCCTATGAGTGGTCAGCCCCGAGTGACCCACTGTCCGTTTCTGTCACAG----------------- :  946
AOTVOKIR3DS4*04v1     : ACAAATGCTTCGGCTCTTTCCGTGACTCTCCCTATGAGTGGTCAGCCCCGAGTGACCCACTGTCCGTTTCTGTCACAG----------------- :  946
AOTVOKIR3DS5*01       : ACAGATGCTTCGGCTCTTTCCGTGACTCTCCCTACCGCTGGTCAACCCCGAGTGACCCACTGTCCGTTTCTGTCACAG----------------- :  946
AOTVOKIR2DS5*01v1     : ACAGATGCTTCGGCTCTTTCCGTGACTCTCCCTACCGCTGGTCAACCCCGAGTGACCCACTGTCCGTTTCTGTCACAG----------------- :  748
AOTVOKIR2DS5*01v2     : ACAGATGCTTCGGCTCTTTCCGTGACTCTCCCTACCGCTGGTCAACCCCGAGTGACCCACTGTCCGTTTCTGTCACAG----------------- :  661
AOTVOKIR3DS7*01       : ACAGATGCTTCGGCTCTTTCCGTGACTCTCCCTACCGCTGGTCAGCCCCGAGTGACCCACTGTCCGTTTCTGTCACAG----------------- :  946
AOTVOKIR3DS7*01v1     : ACAGATGCTTCGGCTCTTTCCGTGACTCTCCCTACCGCTGGTCAGCCCCGAGTGACCCACTGTCCGTTTCTGTCACAG----------------- :  946
AOTVOKIR3DL8*01       : ACAGATGCTTCGGCTCTTTCCGTGACTCTCCCTACGAGTGGTCAGCCCCGAGTGACCCACTGTCCGTTTCTGTCACAG----------------- :  946
AOTVOKIR3DL8*01v1     : ACAGATGCTTCGGCTCTTTCCGTGACTCTCCCTACGAGTGGTCAGCCCCGAGTGACCCACTGTCCGTTTCTGTCACAG----------------- :  946
AOTVOKIR3DS8*01v2     : ACAGATGCTTCGGCTCTTTCCGTGACTCTCCCTACGAGTGGTCAGCCCCGAGTGACCCACTGTCCGTTTCTGTCACAG----------------- :  946
AOTVOKIR3DL8*02       : ACAGATGCTTCGGCTCTTTCCGTGACTCTCCCTACGAGTGGTCAGCCCCGAGTGACCCACTGTCCGTTTCTGTCACAG----------------- :  946
AOTVOKIR4DL9*01       : ACAGATGCTTCGGCTCTTTCCATGGCTCTCTCTATGAGTGGTCAGTCCCCGAGTGACCCACTGTCTGTTTCTGTCACAG----------------- : 1231
AOTVOKIR4DS9*01v1     : ACAGATGCTTCGGCTCTTTCCATGGCTCTCTCTATGAGTGGTCAGTCCCCGAGTGACCCACTGTCTGTTTCTGTCACAGGTGAGGAAACCCCATAC : 1248
AOTVOKIR3DL9*01v2     : ACAGATGCTTCGGCTCTTTCCATGGCTCTCTCTATGAGTGGTCAGTCCCCGAGTGACCCACTGTCTGTTTCTGTCACAG----------------- : 1033
AOTVOKIR4DL9*02       : ACAGATGCTTCGGCTCTTTCCATGGCTCTCTGTATGAGTGGTCAGTCCCCGAGTGACCCACTGTCTGTGTGTGTCACAG----------------- : 1231
AOTVOKIR4DL10*01      : ACAGATGCTTCGGCTCTTTCCATGGCTCTCTCTATGAGTGGTCAGCCCCGAGTGACCCACTGTCTGTTTCTGTCACAG----------------- : 1231
AOTVOKIR3DL10*01v1    : ACAGATGCTTCGGCTCTTTCCATGGCTCTCTCTATGAGTGGTCAGCCCCGAGTGACCCACTGTCTGTTTCTGTCACAG----------------- : 1030
AOTVOKIR4DS10*01v2    : ACAGATGCTTCGGCTCTTTCCATGGCTCTCTCTATGAGTGGTCAGCCCCGAGTGACCCACTGTCTGTTTCTGTCACAG----------------- : 1231
AOTVOKIR3DS10*01v3    : ACAGATGCTTCGGCTCTTTCCATGGCTCTCTCTATGAGTGGTCAGCCCCGAGTGACCCACTGTCTGTTTCTGTCACAG----------------- :  946
AOTVOKIR3DS10*01v4    : ACAGATGCTTCGGCTCTTTCCATGGCTCTCTCTATGAGTGGTCAGCCCCGAGTGACCCACTGTCTGTTTCTGTCACAGGTGAGGAAACCCCATAC :  963
                        ACAgATGCTTCGGCTCTTTCC TG CTCTC cTA gagTGGTCAgcCCCGAGTGACCCACTGTC gTtTcTGTCACAG

                            8080         *         8100         *         8120         *         8140         *         8160         *
BAC_clone_Om          : CTGTCCCATGTCTGATGATCCCAGAGCCATAGCTGAGGAACTTCCTGCTGATGATGGAGGGAAGCGTGGACAGATGCAGAGAGAAGACGAAGCCT : 8170
AOTVOKIR3DL4*01       : ----------------------------------------------------------------------------------------------- :    -
AOTVOKIR3DL4*02       : ----------------------------------------------------------------------------------------------- :    -
AOTVOKIR3DL4*02v1     : ----------------------------------------------------------------------------------------------- :    -
AOTVOKIR3DL4*02v2     : ----------------------------------------------------------------------------------------------- :    -
AOTVOKIR3DL4*03       : ----------------------------------------------------------------------------------------------- :    -
AOTVOKIR3DS4*04       : ----------------------------------------------------------------------------------------------- :    -
AOTVOKIR3DS4*04v1     : ----------------------------------------------------------------------------------------------- :    -
AOTVOKIR3DS5*01       : ----------------------------------------------------------------------------------------------- :    -
AOTVOKIR2DS5*01v1     : ----------------------------------------------------------------------------------------------- :    -
AOTVOKIR2DS5*01v2     : ----------------------------------------------------------------------------------------------- :    -
AOTVOKIR3DS7*01       : ----------------------------------------------------------------------------------------------- :    -
AOTVOKIR3DS7*01v1     : ----------------------------------------------------------------------------------------------- :    -
AOTVOKIR3DL8*01       : ----------------------------------------------------------------------------------------------- :    -
AOTVOKIR3DL8*01v1     : ----------------------------------------------------------------------------------------------- :    -
AOTVOKIR3DS8*01v2     : ----------------------------------------------------------------------------------------------- :    -
AOTVOKIR3DL8*02       : ----------------------------------------------------------------------------------------------- :    -
```

```
AOTVOKIR4DL9*01    : ----------------------------------------------------------------------------------------------- :     -
AOTVOKIR4DS9*01v1  : CTGTCCCATGTCTGA-------------------------------------------------------------------------------- :  1260
AOTVOKIR3DL9*01v2  : ----------------------------------------------------------------------------------------------- :     -
AOTVOKIR4DL9*02    : ----------------------------------------------------------------------------------------------- :     -
AOTVOKIR4DL10*01   : ----------------------------------------------------------------------------------------------- :     -
AOTVOKIR3DL10*01v1 : ----------------------------------------------------------------------------------------------- :     -
AOTVOKIR4DS10*01v2 : ----------------------------------------------------------------------------------------------- :     -
AOTVOKIR3DS10*01v3 : ----------------------------------------------------------------------------------------------- :     -
AOTVOKIR3DS10*01v4 : CTGTCCCATGTCTGA-------------------------------------------------------------------------------- :   975

                           8180         *      8200         *      8220         *      8240         *      8260
BAC_clone_Om       : GGGTGTGAGGACAGGGTCAGGGCGCAGGATGCCAGACAGGGCACCTCCAAGCCCTCCTCCACGACCTGCATGGAGGCCCGCGTTCAGGGCTCTGG :  8265
AOTVOKIR3DL4*01    : ----------------------------------------------------------------------------------------------- :     -
AOTVOKIR3DL4*02    : ----------------------------------------------------------------------------------------------- :     -
AOTVOKIR3DL4*02v1  : ----------------------------------------------------------------------------------------------- :     -
AOTVOKIR3DL4*02v2  : ----------------------------------------------------------------------------------------------- :     -
AOTVOKIR3DL4*03    : ----------------------------------------------------------------------------------------------- :     -
AOTVOKIR3DS4*04    : ----------------------------------------------------------------------------------------------- :     -
AOTVOKIR3DS4*04v1  : ----------------------------------------------------------------------------------------------- :     -
AOTVOKIR3DS5*01    : ----------------------------------------------------------------------------------------------- :     -
AOTVOKIR2DS5*01v1  : ----------------------------------------------------------------------------------------------- :     -
AOTVOKIR2DS5*01v2  : ----------------------------------------------------------------------------------------------- :     -
AOTVOKIR3DS7*01    : ----------------------------------------------------------------------------------------------- :     -
AOTVOKIR3DS7*01v1  : ----------------------------------------------------------------------------------------------- :     -
AOTVOKIR3DL8*01    : ----------------------------------------------------------------------------------------------- :     -
AOTVOKIR3DL8*01v1  : ----------------------------------------------------------------------------------------------- :     -
AOTVOKIR3DS8*01v2  : ----------------------------------------------------------------------------------------------- :     -
AOTVOKIR3DL8*02    : ----------------------------------------------------------------------------------------------- :     -
AOTVOKIR4DL9*01    : ----------------------------------------------------------------------------------------------- :     -
AOTVOKIR4DS9*01v1  : ----------------------------------------------------------------------------------------------- :     -
AOTVOKIR3DL9*01v2  : ----------------------------------------------------------------------------------------------- :     -
AOTVOKIR4DL9*02    : ----------------------------------------------------------------------------------------------- :     -
AOTVOKIR4DL10*01   : ----------------------------------------------------------------------------------------------- :     -
AOTVOKIR3DL10*01v1 : ----------------------------------------------------------------------------------------------- :     -
AOTVOKIR4DS10*01v2 : ----------------------------------------------------------------------------------------------- :     -
AOTVOKIR3DS10*01v3 : ----------------------------------------------------------------------------------------------- :     -
AOTVOKIR3DS10*01v4 : ----------------------------------------------------------------------------------------------- :     -

                         *      8280         *      8300         *      8320         *      8340         *      8360
BAC_clone_Om       : GCACCCAGGCAGATGGAGAAAGCGGTCAGCATAGGCCCAGAGGAGGAGAGACGGGGATCAGTTTGGGGAGATCAGAGGTTCCCTCAGCCCCTCAA :  8360
AOTVOKIR3DL4*01    : ----------------------------------------------------------------------------------------------- :     -
AOTVOKIR3DL4*02    : ----------------------------------------------------------------------------------------------- :     -
AOTVOKIR3DL4*02v1  : ----------------------------------------------------------------------------------------------- :     -
AOTVOKIR3DL4*02v2  : ----------------------------------------------------------------------------------------------- :     -
```

```
AOTVOKIR3DL4*03     : ----------------------------------------------------------------------------------------------- :     -
AOTVOKIR3DS4*04     : ----------------------------------------------------------------------------------------------- :     -
AOTVOKIR3DS4*04v1   : ----------------------------------------------------------------------------------------------- :     -
AOTVOKIR3DS5*01     : ----------------------------------------------------------------------------------------------- :     -
AOTVOKIR2DS5*01v1   : ----------------------------------------------------------------------------------------------- :     -
AOTVOKIR2DS5*01v2   : ----------------------------------------------------------------------------------------------- :     -
AOTVOKIR3DS7*01     : ----------------------------------------------------------------------------------------------- :     -
AOTVOKIR3DS7*01v1   : ----------------------------------------------------------------------------------------------- :     -
AOTVOKIR3DL8*01     : ----------------------------------------------------------------------------------------------- :     -
AOTVOKIR3DL8*01v1   : ----------------------------------------------------------------------------------------------- :     -
AOTVOKIR3DS8*01v2   : ----------------------------------------------------------------------------------------------- :     -
AOTVOKIR3DL8*02     : ----------------------------------------------------------------------------------------------- :     -
AOTVOKIR4DL9*01     : ----------------------------------------------------------------------------------------------- :     -
AOTVOKIR4DS9*01v1   : ----------------------------------------------------------------------------------------------- :     -
AOTVOKIR3DL9*01v2   : ----------------------------------------------------------------------------------------------- :     -
AOTVOKIR4DL9*02     : ----------------------------------------------------------------------------------------------- :     -
AOTVOKIR4DL10*01    : ----------------------------------------------------------------------------------------------- :     -
AOTVOKIR3DL10*01v1  : ----------------------------------------------------------------------------------------------- :     -
AOTVOKIR4DS10*01v2  : ----------------------------------------------------------------------------------------------- :     -
AOTVOKIR3DS10*01v3  : ----------------------------------------------------------------------------------------------- :     -
AOTVOKIR3DS10*01v4  : ----------------------------------------------------------------------------------------------- :     -

                               *      8380          *      8400          *      8420          *      8440          *
BAC_clone_Om        : CCTCACCCATTTCCCAGAAGCCCATCCTGGCCTCTCACCCACAGAGACACGTCATCACCAGCAACCCCCATGCCCTTTTCTTTTCATTTGGAAAA :  8455
AOTVOKIR3DL4*01     : ----------------------------------------------------------------------------------------------- :     -
AOTVOKIR3DL4*02     : ----------------------------------------------------------------------------------------------- :     -
AOTVOKIR3DL4*02v1   : ----------------------------------------------------------------------------------------------- :     -
AOTVOKIR3DL4*02v2   : ----------------------------------------------------------------------------------------------- :     -
AOTVOKIR3DL4*03     : ----------------------------------------------------------------------------------------------- :     -
AOTVOKIR3DS4*04     : ----------------------------------------------------------------------------------------------- :     -
AOTVOKIR3DS4*04v1   : ----------------------------------------------------------------------------------------------- :     -
AOTVOKIR3DS5*01     : ----------------------------------------------------------------------------------------------- :     -
AOTVOKIR2DS5*01v1   : ----------------------------------------------------------------------------------------------- :     -
AOTVOKIR2DS5*01v2   : ----------------------------------------------------------------------------------------------- :     -
AOTVOKIR3DS7*01     : ----------------------------------------------------------------------------------------------- :     -
AOTVOKIR3DS7*01v1   : ----------------------------------------------------------------------------------------------- :     -
AOTVOKIR3DL8*01     : ----------------------------------------------------------------------------------------------- :     -
AOTVOKIR3DL8*01v1   : ----------------------------------------------------------------------------------------------- :     -
AOTVOKIR3DS8*01v2   : ----------------------------------------------------------------------------------------------- :     -
AOTVOKIR3DL8*02     : ----------------------------------------------------------------------------------------------- :     -
AOTVOKIR4DL9*01     : ----------------------------------------------------------------------------------------------- :     -
AOTVOKIR4DS9*01v1   : ----------------------------------------------------------------------------------------------- :     -
AOTVOKIR3DL9*01v2   : ----------------------------------------------------------------------------------------------- :     -
AOTVOKIR4DL9*02     : ----------------------------------------------------------------------------------------------- :     -
AOTVOKIR4DL10*01    : ----------------------------------------------------------------------------------------------- :     -
```

```
AOTVOKIR3DL10*01v1 : ----------------------------------------------------------------------------------------------- :    -
AOTVOKIR4DS10*01v2 : ----------------------------------------------------------------------------------------------- :    -
AOTVOKIR3DS10*01v3 : ----------------------------------------------------------------------------------------------- :    -
AOTVOKIR3DS10*01v4 : ----------------------------------------------------------------------------------------------- :    -

                          8460         *        8480         *        8500         *        8520         *        8540         *
BAC_clone_Om       : ACATTTATTGAGGTTAAATATACCCATATAATTTACCACCTTTACCTTTTTATGAGACAGAGTCTTGCTCCATTGCCCAGGCTGGAGCGCAATTG : 8550
AOTVOKIR3DL4*01    : ----------------------------------------------------------------------------------------------- :    -
AOTVOKIR3DL4*02    : ----------------------------------------------------------------------------------------------- :    -
AOTVOKIR3DL4*02v1  : ----------------------------------------------------------------------------------------------- :    -
AOTVOKIR3DL4*02v2  : ----------------------------------------------------------------------------------------------- :    -
AOTVOKIR3DL4*03    : ----------------------------------------------------------------------------------------------- :    -
AOTVOKIR3DS4*04    : ----------------------------------------------------------------------------------------------- :    -
AOTVOKIR3DS4*04v1  : ----------------------------------------------------------------------------------------------- :    -
AOTVOKIR3DS5*01    : ----------------------------------------------------------------------------------------------- :    -
AOTVOKIR2DS5*01v1  : ----------------------------------------------------------------------------------------------- :    -
AOTVOKIR2DS5*01v2  : ----------------------------------------------------------------------------------------------- :    -
AOTVOKIR3DS7*01    : ----------------------------------------------------------------------------------------------- :    -
AOTVOKIR3DS7*01v1  : ----------------------------------------------------------------------------------------------- :    -
AOTVOKIR3DL8*01    : ----------------------------------------------------------------------------------------------- :    -
AOTVOKIR3DL8*01v1  : ----------------------------------------------------------------------------------------------- :    -
AOTVOKIR3DS8*01v2  : ----------------------------------------------------------------------------------------------- :    -
AOTVOKIR3DL8*02    : ----------------------------------------------------------------------------------------------- :    -
AOTVOKIR4DL9*01    : ----------------------------------------------------------------------------------------------- :    -
AOTVOKIR4DS9*01v1  : ----------------------------------------------------------------------------------------------- :    -
AOTVOKIR3DL9*01v2  : ----------------------------------------------------------------------------------------------- :    -
AOTVOKIR4DL9*02    : ----------------------------------------------------------------------------------------------- :    -
AOTVOKIR4DL10*01   : ----------------------------------------------------------------------------------------------- :    -
AOTVOKIR3DL10*01v1 : ----------------------------------------------------------------------------------------------- :    -
AOTVOKIR4DS10*01v2 : ----------------------------------------------------------------------------------------------- :    -
AOTVOKIR3DS10*01v3 : ----------------------------------------------------------------------------------------------- :    -
AOTVOKIR3DS10*01v4 : ----------------------------------------------------------------------------------------------- :    -

                               8560         *        8580         *        8600         *        8620         *        8640
BAC_clone_Om       : CACGATCTCAGCTCACTGCAACCTCTGCCTCCTGGGTTCAAGCAATTGTCCTTCCTCAGCCTCCCAAATAGTTGGGATTACAGGCGCGCGCCACC : 8645
AOTVOKIR3DL4*01    : ----------------------------------------------------------------------------------------------- :    -
AOTVOKIR3DL4*02    : ----------------------------------------------------------------------------------------------- :    -
AOTVOKIR3DL4*02v1  : ----------------------------------------------------------------------------------------------- :    -
AOTVOKIR3DL4*02v2  : ----------------------------------------------------------------------------------------------- :    -
AOTVOKIR3DL4*03    : ----------------------------------------------------------------------------------------------- :    -
AOTVOKIR3DS4*04    : ----------------------------------------------------------------------------------------------- :    -
AOTVOKIR3DS4*04v1  : ----------------------------------------------------------------------------------------------- :    -
AOTVOKIR3DS5*01    : ----------------------------------------------------------------------------------------------- :    -
AOTVOKIR2DS5*01v1  : ----------------------------------------------------------------------------------------------- :    -
```

```
AOTVOKIR2DS5*01v2   : ----------------------------------------------------------------------------------------------- :      -
AOTVOKIR3DS7*01     : ----------------------------------------------------------------------------------------------- :      -
AOTVOKIR3DS7*01v1   : ----------------------------------------------------------------------------------------------- :      -
AOTVOKIR3DL8*01     : ----------------------------------------------------------------------------------------------- :      -
AOTVOKIR3DL8*01v1   : ----------------------------------------------------------------------------------------------- :      -
AOTVOKIR3DS8*01v2   : ----------------------------------------------------------------------------------------------- :      -
AOTVOKIR3DL8*02     : ----------------------------------------------------------------------------------------------- :      -
AOTVOKIR4DL9*01     : ----------------------------------------------------------------------------------------------- :      -
AOTVOKIR4DS9*01v1   : ----------------------------------------------------------------------------------------------- :      -
AOTVOKIR3DL9*01v2   : ----------------------------------------------------------------------------------------------- :      -
AOTVOKIR4DL9*02     : ----------------------------------------------------------------------------------------------- :      -
AOTVOKIR4DL10*01    : ----------------------------------------------------------------------------------------------- :      -
AOTVOKIR3DL10*01v1  : ----------------------------------------------------------------------------------------------- :      -
AOTVOKIR4DS10*01v2  : ----------------------------------------------------------------------------------------------- :      -
AOTVOKIR3DS10*01v3  : ----------------------------------------------------------------------------------------------- :      -
AOTVOKIR3DS10*01v4  : ----------------------------------------------------------------------------------------------- :      -

                          *      8660         *      8680         *      8700         *      8720         *      8740
BAC_clone_Om        : ACGCCTGGCTAATTTTTTTATTTTTAGTAGAGATGAGGTTTCACCATGTTGGCCAGGCTGGTCTTGAACTCCTCACCTTGTGGTCCACCGCCTCG :   8740
AOTVOKIR3DL4*01     : ----------------------------------------------------------------------------------------------- :      -
AOTVOKIR3DL4*02     : ----------------------------------------------------------------------------------------------- :      -
AOTVOKIR3DL4*02v1   : ----------------------------------------------------------------------------------------------- :      -
AOTVOKIR3DL4*02v2   : ----------------------------------------------------------------------------------------------- :      -
AOTVOKIR3DL4*03     : ----------------------------------------------------------------------------------------------- :      -
AOTVOKIR3DS4*04     : ----------------------------------------------------------------------------------------------- :      -
AOTVOKIR3DS4*04v1   : ----------------------------------------------------------------------------------------------- :      -
AOTVOKIR3DS5*01     : ----------------------------------------------------------------------------------------------- :      -
AOTVOKIR2DS5*01v1   : ----------------------------------------------------------------------------------------------- :      -
AOTVOKIR2DS5*01v2   : ----------------------------------------------------------------------------------------------- :      -
AOTVOKIR3DS7*01     : ----------------------------------------------------------------------------------------------- :      -
AOTVOKIR3DS7*01v1   : ----------------------------------------------------------------------------------------------- :      -
AOTVOKIR3DL8*01     : ----------------------------------------------------------------------------------------------- :      -
AOTVOKIR3DL8*01v1   : ----------------------------------------------------------------------------------------------- :      -
AOTVOKIR3DS8*01v2   : ----------------------------------------------------------------------------------------------- :      -
AOTVOKIR3DL8*02     : ----------------------------------------------------------------------------------------------- :      -
AOTVOKIR4DL9*01     : ----------------------------------------------------------------------------------------------- :      -
AOTVOKIR4DS9*01v1   : ----------------------------------------------------------------------------------------------- :      -
AOTVOKIR3DL9*01v2   : ----------------------------------------------------------------------------------------------- :      -
AOTVOKIR4DL9*02     : ----------------------------------------------------------------------------------------------- :      -
AOTVOKIR4DL10*01    : ----------------------------------------------------------------------------------------------- :      -
AOTVOKIR3DL10*01v1  : ----------------------------------------------------------------------------------------------- :      -
AOTVOKIR4DS10*01v2  : ----------------------------------------------------------------------------------------------- :      -
AOTVOKIR3DS10*01v3  : ----------------------------------------------------------------------------------------------- :      -
AOTVOKIR3DS10*01v4  : ----------------------------------------------------------------------------------------------- :      -
```

```
                                   *        8760          *        8780          *        8800          *        8820          *
BAC_clone_Om         : GCCACCCAAAGTGCTGGGATTACAGGCGTGAGCCACTGCATCCGGCCTCACCTTTCCCATTTTTAAGTGTAAAGTCCAGTGGTCAGGGATACATT :  8835
AOTVOKIR3DL4*01      : ----------------------------------------------------------------------------------------------- :     -
AOTVOKIR3DL4*02      : ----------------------------------------------------------------------------------------------- :     -
AOTVOKIR3DL4*02v1    : ----------------------------------------------------------------------------------------------- :     -
AOTVOKIR3DL4*02v2    : ----------------------------------------------------------------------------------------------- :     -
AOTVOKIR3DL4*03      : ----------------------------------------------------------------------------------------------- :     -
AOTVOKIR3DS4*04      : ----------------------------------------------------------------------------------------------- :     -
AOTVOKIR3DS4*04v1    : ----------------------------------------------------------------------------------------------- :     -
AOTVOKIR3DS5*01      : ----------------------------------------------------------------------------------------------- :     -
AOTVOKIR2DS5*01v1    : ----------------------------------------------------------------------------------------------- :     -
AOTVOKIR2DS5*01v2    : ----------------------------------------------------------------------------------------------- :     -
AOTVOKIR3DS7*01      : ----------------------------------------------------------------------------------------------- :     -
AOTVOKIR3DS7*01v1    : ----------------------------------------------------------------------------------------------- :     -
AOTVOKIR3DL8*01      : ----------------------------------------------------------------------------------------------- :     -
AOTVOKIR3DL8*01v1    : ----------------------------------------------------------------------------------------------- :     -
AOTVOKIR3DS8*01v2    : ----------------------------------------------------------------------------------------------- :     -
AOTVOKIR3DL8*02      : ----------------------------------------------------------------------------------------------- :     -
AOTVOKIR4DL9*01      : ----------------------------------------------------------------------------------------------- :     -
AOTVOKIR4DS9*01v1    : ----------------------------------------------------------------------------------------------- :     -
AOTVOKIR3DL9*01v2    : ----------------------------------------------------------------------------------------------- :     -
AOTVOKIR4DL9*02      : ----------------------------------------------------------------------------------------------- :     -
AOTVOKIR4DL10*01     : ----------------------------------------------------------------------------------------------- :     -
AOTVOKIR3DL10*01v1   : ----------------------------------------------------------------------------------------------- :     -
AOTVOKIR4DS10*01v2   : ----------------------------------------------------------------------------------------------- :     -
AOTVOKIR3DS10*01v3   : ----------------------------------------------------------------------------------------------- :     -
AOTVOKIR3DS10*01v4   : ----------------------------------------------------------------------------------------------- :     -

                          8840          *        8860          *        8880          *        8900          *        8920          *
BAC_clone_Om         : TATATTCTTTTTTTCGTTGTTACCCTCCACCATTCCCTTCCCGGCCTCTGGTAGCCACCATTCTCTCTACCTTCCTGAGATCCACCTGTTAGCTC :  8930
AOTVOKIR3DL4*01      : ----------------------------------------------------------------------------------------------- :     -
AOTVOKIR3DL4*02      : ----------------------------------------------------------------------------------------------- :     -
AOTVOKIR3DL4*02v1    : ----------------------------------------------------------------------------------------------- :     -
AOTVOKIR3DL4*02v2    : ----------------------------------------------------------------------------------------------- :     -
AOTVOKIR3DL4*03      : ----------------------------------------------------------------------------------------------- :     -
AOTVOKIR3DS4*04      : ----------------------------------------------------------------------------------------------- :     -
AOTVOKIR3DS4*04v1    : ----------------------------------------------------------------------------------------------- :     -
AOTVOKIR3DS5*01      : ----------------------------------------------------------------------------------------------- :     -
AOTVOKIR2DS5*01v1    : ----------------------------------------------------------------------------------------------- :     -
AOTVOKIR2DS5*01v2    : ----------------------------------------------------------------------------------------------- :     -
AOTVOKIR3DS7*01      : ----------------------------------------------------------------------------------------------- :     -
AOTVOKIR3DS7*01v1    : ----------------------------------------------------------------------------------------------- :     -
AOTVOKIR3DL8*01      : ----------------------------------------------------------------------------------------------- :     -
AOTVOKIR3DL8*01v1    : ----------------------------------------------------------------------------------------------- :     -
```

```
AOTVOKIR3DS8*01v2  : ----------------------------------------------------------------------------------------------- :    -
AOTVOKIR3DL8*02    : ----------------------------------------------------------------------------------------------- :    -
AOTVOKIR4DL9*01    : ----------------------------------------------------------------------------------------------- :    -
AOTVOKIR4DS9*01v1  : ----------------------------------------------------------------------------------------------- :    -
AOTVOKIR3DL9*01v2  : ----------------------------------------------------------------------------------------------- :    -
AOTVOKIR4DL9*02    : ----------------------------------------------------------------------------------------------- :    -
AOTVOKIR4DL10*01   : ----------------------------------------------------------------------------------------------- :    -
AOTVOKIR3DL10*01v1 : ----------------------------------------------------------------------------------------------- :    -
AOTVOKIR4DS10*01v2 : ----------------------------------------------------------------------------------------------- :    -
AOTVOKIR3DS10*01v3 : ----------------------------------------------------------------------------------------------- :    -
AOTVOKIR3DS10*01v4 : ----------------------------------------------------------------------------------------------- :    -

                           8940         *      8960         *      8980         *      9000         *      9020
BAC_clone_Om       : CTGCATATGGGTGAGACATGGCCATCTTTGTAATGCCCTCCAGTTCATCCATGTGTCTGGAAGTGACAGGACGTTGTTGCTTGTATGGAGGAGTG :  9025
AOTVOKIR3DL4*01    : ----------------------------------------------------------------------------------------------- :    -
AOTVOKIR3DL4*02    : ----------------------------------------------------------------------------------------------- :    -
AOTVOKIR3DL4*02v1  : ----------------------------------------------------------------------------------------------- :    -
AOTVOKIR3DL4*02v2  : ----------------------------------------------------------------------------------------------- :    -
AOTVOKIR3DL4*03    : ----------------------------------------------------------------------------------------------- :    -
AOTVOKIR3DS4*04    : ----------------------------------------------------------------------------------------------- :    -
AOTVOKIR3DS4*04v1  : ----------------------------------------------------------------------------------------------- :    -
AOTVOKIR3DS5*01    : ----------------------------------------------------------------------------------------------- :    -
AOTVOKIR2DS5*01v1  : ----------------------------------------------------------------------------------------------- :    -
AOTVOKIR2DS5*01v2  : ----------------------------------------------------------------------------------------------- :    -
AOTVOKIR3DS7*01    : ----------------------------------------------------------------------------------------------- :    -
AOTVOKIR3DS7*01v1  : ----------------------------------------------------------------------------------------------- :    -
AOTVOKIR3DL8*01    : ----------------------------------------------------------------------------------------------- :    -
AOTVOKIR3DL8*01v1  : ----------------------------------------------------------------------------------------------- :    -
AOTVOKIR3DS8*01v2  : ----------------------------------------------------------------------------------------------- :    -
AOTVOKIR3DL8*02    : ----------------------------------------------------------------------------------------------- :    -
AOTVOKIR4DL9*01    : ----------------------------------------------------------------------------------------------- :    -
AOTVOKIR4DS9*01v1  : ----------------------------------------------------------------------------------------------- :    -
AOTVOKIR3DL9*01v2  : ----------------------------------------------------------------------------------------------- :    -
AOTVOKIR4DL9*02    : ----------------------------------------------------------------------------------------------- :    -
AOTVOKIR4DL10*01   : ----------------------------------------------------------------------------------------------- :    -
AOTVOKIR3DL10*01v1 : ----------------------------------------------------------------------------------------------- :    -
AOTVOKIR4DS10*01v2 : ----------------------------------------------------------------------------------------------- :    -
AOTVOKIR3DS10*01v3 : ----------------------------------------------------------------------------------------------- :    -
AOTVOKIR3DS10*01v4 : ----------------------------------------------------------------------------------------------- :    -

                         *      9040         *      9060         *      9080         *      9100         *      9120
BAC_clone_Om       : GTCTCCATTGTGCGTATGTGCTACGTTCTCTCTATCCGCTCACCCGCTGATGGGCAGGTGGGTTCACTGCACGCCTTGGCTACTGTGAATTGTGC :  9120
AOTVOKIR3DL4*01    : ----------------------------------------------------------------------------------------------- :    -
AOTVOKIR3DL4*02    : ----------------------------------------------------------------------------------------------- :    -
```

```
AOTVOKIR3DL4*02v1  : ----------------------------------------------------------------------------------------------- :     -
AOTVOKIR3DL4*02v2  : ----------------------------------------------------------------------------------------------- :     -
AOTVOKIR3DL4*03    : ----------------------------------------------------------------------------------------------- :     -
AOTVOKIR3DS4*04    : ----------------------------------------------------------------------------------------------- :     -
AOTVOKIR3DS4*04v1  : ----------------------------------------------------------------------------------------------- :     -
AOTVOKIR3DS5*01    : ----------------------------------------------------------------------------------------------- :     -
AOTVOKIR2DS5*01v1  : ----------------------------------------------------------------------------------------------- :     -
AOTVOKIR2DS5*01v2  : ----------------------------------------------------------------------------------------------- :     -
AOTVOKIR3DS7*01    : ----------------------------------------------------------------------------------------------- :     -
AOTVOKIR3DS7*01v1  : ----------------------------------------------------------------------------------------------- :     -
AOTVOKIR3DL8*01    : ----------------------------------------------------------------------------------------------- :     -
AOTVOKIR3DL8*01v1  : ----------------------------------------------------------------------------------------------- :     -
AOTVOKIR3DS8*01v2  : ----------------------------------------------------------------------------------------------- :     -
AOTVOKIR3DL8*02    : ----------------------------------------------------------------------------------------------- :     -
AOTVOKIR4DL9*01    : ----------------------------------------------------------------------------------------------- :     -
AOTVOKIR4DS9*01v1  : ----------------------------------------------------------------------------------------------- :     -
AOTVOKIR3DL9*01v2  : ----------------------------------------------------------------------------------------------- :     -
AOTVOKIR4DL9*02    : ----------------------------------------------------------------------------------------------- :     -
AOTVOKIR4DL10*01   : ----------------------------------------------------------------------------------------------- :     -
AOTVOKIR3DL10*01v1 : ----------------------------------------------------------------------------------------------- :     -
AOTVOKIR4DS10*01v2 : ----------------------------------------------------------------------------------------------- :     -
AOTVOKIR3DS10*01v3 : ----------------------------------------------------------------------------------------------- :     -
AOTVOKIR3DS10*01v4 : ----------------------------------------------------------------------------------------------- :     -

                              *      9140          *      9160          *      9180          *      9200          *
BAC_clone_Om       : TGCACCAATCATGGGAGCGCAGATATCACTCCGATACACTGATGTCCTTTCCTTTGGGTGTAAACCCAGTAGTGAAATTGCTGGATACTATGAAA :  9215
AOTVOKIR3DL4*01    : ----------------------------------------------------------------------------------------------- :     -
AOTVOKIR3DL4*02    : ----------------------------------------------------------------------------------------------- :     -
AOTVOKIR3DL4*02v1  : ----------------------------------------------------------------------------------------------- :     -
AOTVOKIR3DL4*02v2  : ----------------------------------------------------------------------------------------------- :     -
AOTVOKIR3DL4*03    : ----------------------------------------------------------------------------------------------- :     -
AOTVOKIR3DS4*04    : ----------------------------------------------------------------------------------------------- :     -
AOTVOKIR3DS4*04v1  : ----------------------------------------------------------------------------------------------- :     -
AOTVOKIR3DS5*01    : ----------------------------------------------------------------------------------------------- :     -
AOTVOKIR2DS5*01v1  : ----------------------------------------------------------------------------------------------- :     -
AOTVOKIR2DS5*01v2  : ----------------------------------------------------------------------------------------------- :     -
AOTVOKIR3DS7*01    : ----------------------------------------------------------------------------------------------- :     -
AOTVOKIR3DS7*01v1  : ----------------------------------------------------------------------------------------------- :     -
AOTVOKIR3DL8*01    : ----------------------------------------------------------------------------------------------- :     -
AOTVOKIR3DL8*01v1  : ----------------------------------------------------------------------------------------------- :     -
AOTVOKIR3DS8*01v2  : ----------------------------------------------------------------------------------------------- :     -
AOTVOKIR3DL8*02    : ----------------------------------------------------------------------------------------------- :     -
AOTVOKIR4DL9*01    : ----------------------------------------------------------------------------------------------- :     -
AOTVOKIR4DS9*01v1  : ----------------------------------------------------------------------------------------------- :     -
AOTVOKIR3DL9*01v2  : ----------------------------------------------------------------------------------------------- :     -
```

```
AOTVOKIR4DL9*02     : ----------------------------------------------------------------------------------------------- :     -
AOTVOKIR4DL10*01    : ----------------------------------------------------------------------------------------------- :     -
AOTVOKIR3DL10*01v1  : ----------------------------------------------------------------------------------------------- :     -
AOTVOKIR4DS10*01v2  : ----------------------------------------------------------------------------------------------- :     -
AOTVOKIR3DS10*01v3  : ----------------------------------------------------------------------------------------------- :     -
AOTVOKIR3DS10*01v4  : ----------------------------------------------------------------------------------------------- :     -

                          9220         *       9240         *       9260         *       9280         *       9300         *
BAC_clone_Om        : GTTCTCTTTTTAGCTTTTTTTTTTTCTTGAGATGGAGTTTCCCTCTGTAGCCCAGGCTGGAGGGCAGTGGCACCATCTTGGCTCATTGCAGCCTC :  9310
AOTVOKIR3DL4*01     : ----------------------------------------------------------------------------------------------- :     -
AOTVOKIR3DL4*02     : ----------------------------------------------------------------------------------------------- :     -
AOTVOKIR3DL4*02v1   : ----------------------------------------------------------------------------------------------- :     -
AOTVOKIR3DL4*02v2   : ----------------------------------------------------------------------------------------------- :     -
AOTVOKIR3DL4*03     : ----------------------------------------------------------------------------------------------- :     -
AOTVOKIR3DS4*04     : ----------------------------------------------------------------------------------------------- :     -
AOTVOKIR3DS4*04v1   : ----------------------------------------------------------------------------------------------- :     -
AOTVOKIR3DS5*01     : ----------------------------------------------------------------------------------------------- :     -
AOTVOKIR2DS5*01v1   : ----------------------------------------------------------------------------------------------- :     -
AOTVOKIR2DS5*01v2   : ----------------------------------------------------------------------------------------------- :     -
AOTVOKIR3DS7*01     : ----------------------------------------------------------------------------------------------- :     -
AOTVOKIR3DS7*01v1   : ----------------------------------------------------------------------------------------------- :     -
AOTVOKIR3DL8*01     : ----------------------------------------------------------------------------------------------- :     -
AOTVOKIR3DL8*01v1   : ----------------------------------------------------------------------------------------------- :     -
AOTVOKIR3DS8*01v2   : ----------------------------------------------------------------------------------------------- :     -
AOTVOKIR3DL8*02     : ----------------------------------------------------------------------------------------------- :     -
AOTVOKIR4DL9*01     : ----------------------------------------------------------------------------------------------- :     -
AOTVOKIR4DS9*01v1   : ----------------------------------------------------------------------------------------------- :     -
AOTVOKIR3DL9*01v2   : ----------------------------------------------------------------------------------------------- :     -
AOTVOKIR4DL9*02     : ----------------------------------------------------------------------------------------------- :     -
AOTVOKIR4DL10*01    : ----------------------------------------------------------------------------------------------- :     -
AOTVOKIR3DL10*01v1  : ----------------------------------------------------------------------------------------------- :     -
AOTVOKIR4DS10*01v2  : ----------------------------------------------------------------------------------------------- :     -
AOTVOKIR3DS10*01v3  : ----------------------------------------------------------------------------------------------- :     -
AOTVOKIR3DS10*01v4  : ----------------------------------------------------------------------------------------------- :     -

                               9320         *       9340         *       9360         *       9380         *       9400
BAC_clone_Om        : TGCCTCCTGGGTTCACAGGATTCTCCTACCTCAGCCTCCCTAGTAGGAATTTTCCCTCTTTGACACAAGGGCCCTCCTTGTCATTGACCTCAGCT :  9405
AOTVOKIR3DL4*01     : ----------------------------------------------------------------------------------------------- :     -
AOTVOKIR3DL4*02     : ----------------------------------------------------------------------------------------------- :     -
AOTVOKIR3DL4*02v1   : ----------------------------------------------------------------------------------------------- :     -
AOTVOKIR3DL4*02v2   : ----------------------------------------------------------------------------------------------- :     -
AOTVOKIR3DL4*03     : ----------------------------------------------------------------------------------------------- :     -
AOTVOKIR3DS4*04     : ----------------------------------------------------------------------------------------------- :     -
AOTVOKIR3DS4*04v1   : ----------------------------------------------------------------------------------------------- :     -
```

```
AOTVOKIR3DS5*01     : ----------------------------------------------------------------------------------------------- :     -
AOTVOKIR2DS5*01v1   : ----------------------------------------------------------------------------------------------- :     -
AOTVOKIR2DS5*01v2   : ----------------------------------------------------------------------------------------------- :     -
AOTVOKIR3DS7*01     : ----------------------------------------------------------------------------------------------- :     -
AOTVOKIR3DS7*01v1   : ----------------------------------------------------------------------------------------------- :     -
AOTVOKIR3DL8*01     : ----------------------------------------------------------------------------------------------- :     -
AOTVOKIR3DL8*01v1   : ----------------------------------------------------------------------------------------------- :     -
AOTVOKIR3DS8*01v2   : ----------------------------------------------------------------------------------------------- :     -
AOTVOKIR3DL8*02     : ----------------------------------------------------------------------------------------------- :     -
AOTVOKIR4DL9*01     : ----------------------------------------------------------------------------------------------- :     -
AOTVOKIR4DS9*01v1   : ----------------------------------------------------------------------------------------------- :     -
AOTVOKIR3DL9*01v2   : ----------------------------------------------------------------------------------------------- :     -
AOTVOKIR4DL9*02     : ----------------------------------------------------------------------------------------------- :     -
AOTVOKIR4DL10*01    : ----------------------------------------------------------------------------------------------- :     -
AOTVOKIR3DL10*01v1  : ----------------------------------------------------------------------------------------------- :     -
AOTVOKIR4DS10*01v2  : ----------------------------------------------------------------------------------------------- :     -
AOTVOKIR3DS10*01v3  : ----------------------------------------------------------------------------------------------- :     -
AOTVOKIR3DS10*01v4  : ----------------------------------------------------------------------------------------------- :     -

                          *      9420         *     9440         *       9460         *       9480         *      9500
BAC_clone_Om        : AAACTATGGCCATTTATAAATAGAGGAAACATTAATGAATTAAAAGCTTTCCTGATTTTTGTAACTAATAGTTGCACGTTTTCTTAGTCTCTTTC :  9500
AOTVOKIR3DL4*01     : ----------------------------------------------------------------------------------------------- :     -
AOTVOKIR3DL4*02     : ----------------------------------------------------------------------------------------------- :     -
AOTVOKIR3DL4*02v1   : ----------------------------------------------------------------------------------------------- :     -
AOTVOKIR3DL4*02v2   : ----------------------------------------------------------------------------------------------- :     -
AOTVOKIR3DL4*03     : ----------------------------------------------------------------------------------------------- :     -
AOTVOKIR3DS4*04     : ----------------------------------------------------------------------------------------------- :     -
AOTVOKIR3DS4*04v1   : ----------------------------------------------------------------------------------------------- :     -
AOTVOKIR3DS5*01     : ----------------------------------------------------------------------------------------------- :     -
AOTVOKIR2DS5*01v1   : ----------------------------------------------------------------------------------------------- :     -
AOTVOKIR2DS5*01v2   : ----------------------------------------------------------------------------------------------- :     -
AOTVOKIR3DS7*01     : ----------------------------------------------------------------------------------------------- :     -
AOTVOKIR3DS7*01v1   : ----------------------------------------------------------------------------------------------- :     -
AOTVOKIR3DL8*01     : ----------------------------------------------------------------------------------------------- :     -
AOTVOKIR3DL8*01v1   : ----------------------------------------------------------------------------------------------- :     -
AOTVOKIR3DS8*01v2   : ----------------------------------------------------------------------------------------------- :     -
AOTVOKIR3DL8*02     : ----------------------------------------------------------------------------------------------- :     -
AOTVOKIR4DL9*01     : ----------------------------------------------------------------------------------------------- :     -
AOTVOKIR4DS9*01v1   : ----------------------------------------------------------------------------------------------- :     -
AOTVOKIR3DL9*01v2   : ----------------------------------------------------------------------------------------------- :     -
AOTVOKIR4DL9*02     : ----------------------------------------------------------------------------------------------- :     -
AOTVOKIR4DL10*01    : ----------------------------------------------------------------------------------------------- :     -
AOTVOKIR3DL10*01v1  : ----------------------------------------------------------------------------------------------- :     -
AOTVOKIR4DS10*01v2  : ----------------------------------------------------------------------------------------------- :     -
AOTVOKIR3DS10*01v3  : ----------------------------------------------------------------------------------------------- :     -
```

```
AOTVOKIR3DS10*01v4 : ----------------------------------------------------------------------------------------------- :     -

                               *      9520         *      9540         *      9560         *      9580         *
BAC_clone_Om       : CAAGTCCTTGGAAATTACAGGTACACGCTACCACGCCTGGCTAATTTTTGTATTTTTTAGTACAGACAGGGTTTCACCAGGTTGGCTAGCCTGCT :  9595
AOTVOKIR3DL4*01    : ----------------------------------------------------------------------------------------------- :     -
AOTVOKIR3DL4*02    : ----------------------------------------------------------------------------------------------- :     -
AOTVOKIR3DL4*02v1  : ----------------------------------------------------------------------------------------------- :     -
AOTVOKIR3DL4*02v2  : ----------------------------------------------------------------------------------------------- :     -
AOTVOKIR3DL4*03    : ----------------------------------------------------------------------------------------------- :     -
AOTVOKIR3DS4*04    : ----------------------------------------------------------------------------------------------- :     -
AOTVOKIR3DS4*04v1  : ----------------------------------------------------------------------------------------------- :     -
AOTVOKIR3DS5*01    : ----------------------------------------------------------------------------------------------- :     -
AOTVOKIR2DS5*01v1  : ----------------------------------------------------------------------------------------------- :     -
AOTVOKIR2DS5*01v2  : ----------------------------------------------------------------------------------------------- :     -
AOTVOKIR3DS7*01    : ----------------------------------------------------------------------------------------------- :     -
AOTVOKIR3DS7*01v1  : ----------------------------------------------------------------------------------------------- :     -
AOTVOKIR3DL8*01    : ----------------------------------------------------------------------------------------------- :     -
AOTVOKIR3DL8*01v1  : ----------------------------------------------------------------------------------------------- :     -
AOTVOKIR3DS8*01v2  : ----------------------------------------------------------------------------------------------- :     -
AOTVOKIR3DL8*02    : ----------------------------------------------------------------------------------------------- :     -
AOTVOKIR4DL9*01    : ----------------------------------------------------------------------------------------------- :     -
AOTVOKIR4DS9*01v1  : ----------------------------------------------------------------------------------------------- :     -
AOTVOKIR3DL9*01v2  : ----------------------------------------------------------------------------------------------- :     -
AOTVOKIR4DL9*02    : ----------------------------------------------------------------------------------------------- :     -
AOTVOKIR4DL10*01   : ----------------------------------------------------------------------------------------------- :     -
AOTVOKIR3DL10*01v1 : ----------------------------------------------------------------------------------------------- :     -
AOTVOKIR4DS10*01v2 : ----------------------------------------------------------------------------------------------- :     -
AOTVOKIR3DS10*01v3 : ----------------------------------------------------------------------------------------------- :     -
AOTVOKIR3DS10*01v4 : ----------------------------------------------------------------------------------------------- :     -

                      9600         *      9620         *      9640         *      9660         *      9680         *
BAC_clone_Om       : CGCAAATTCCTGACCACAAGTGAGGTGCCCACCTCGGTTTCCCAAAGTGCTGGGATTACAGACGTGAGCCAACACGCCTGTCCGATTTCTAGTTA :  9690
AOTVOKIR3DL4*01    : ----------------------------------------------------------------------------------------------- :     -
AOTVOKIR3DL4*02    : ----------------------------------------------------------------------------------------------- :     -
AOTVOKIR3DL4*02v1  : ----------------------------------------------------------------------------------------------- :     -
AOTVOKIR3DL4*02v2  : ----------------------------------------------------------------------------------------------- :     -
AOTVOKIR3DL4*03    : ----------------------------------------------------------------------------------------------- :     -
AOTVOKIR3DS4*04    : ----------------------------------------------------------------------------------------------- :     -
AOTVOKIR3DS4*04v1  : ----------------------------------------------------------------------------------------------- :     -
AOTVOKIR3DS5*01    : ----------------------------------------------------------------------------------------------- :     -
AOTVOKIR2DS5*01v1  : ----------------------------------------------------------------------------------------------- :     -
AOTVOKIR2DS5*01v2  : ----------------------------------------------------------------------------------------------- :     -
AOTVOKIR3DS7*01    : ----------------------------------------------------------------------------------------------- :     -
AOTVOKIR3DS7*01v1  : ----------------------------------------------------------------------------------------------- :     -
```

```
AOTVOKIR3DL8*01     : ----------------------------------------------------------------------------------------------- :     -
AOTVOKIR3DL8*01v1   : ----------------------------------------------------------------------------------------------- :     -
AOTVOKIR3DS8*01v2   : ----------------------------------------------------------------------------------------------- :     -
AOTVOKIR3DL8*02     : ----------------------------------------------------------------------------------------------- :     -
AOTVOKIR4DL9*01     : ----------------------------------------------------------------------------------------------- :     -
AOTVOKIR4DS9*01v1   : ----------------------------------------------------------------------------------------------- :     -
AOTVOKIR3DL9*01v2   : ----------------------------------------------------------------------------------------------- :     -
AOTVOKIR4DL9*02     : ----------------------------------------------------------------------------------------------- :     -
AOTVOKIR4DL10*01    : ----------------------------------------------------------------------------------------------- :     -
AOTVOKIR3DL10*01v1  : ----------------------------------------------------------------------------------------------- :     -
AOTVOKIR4DS10*01v2  : ----------------------------------------------------------------------------------------------- :     -
AOTVOKIR3DS10*01v3  : ----------------------------------------------------------------------------------------------- :     -
AOTVOKIR3DS10*01v4  : ----------------------------------------------------------------------------------------------- :     -

                              9700         *        9720         *        9740         *        9760         *        9780
BAC_clone_Om        : TTAATCCCATCTCAGAATCATCGTTTGCGAATAGTTGCTCTCATTCTGTGGGTTGTCTCATCACTTCTTTGGTTTATCTTTCGTGGTGCAGAAGT :  9785
AOTVOKIR3DL4*01     : ----------------------------------------------------------------------------------------------- :     -
AOTVOKIR3DL4*02     : ----------------------------------------------------------------------------------------------- :     -
AOTVOKIR3DL4*02v1   : ----------------------------------------------------------------------------------------------- :     -
AOTVOKIR3DL4*02v2   : ----------------------------------------------------------------------------------------------- :     -
AOTVOKIR3DL4*03     : ----------------------------------------------------------------------------------------------- :     -
AOTVOKIR3DS4*04     : ----------------------------------------------------------------------------------------------- :     -
AOTVOKIR3DS4*04v1   : ----------------------------------------------------------------------------------------------- :     -
AOTVOKIR3DS5*01     : ----------------------------------------------------------------------------------------------- :     -
AOTVOKIR2DS5*01v1   : ----------------------------------------------------------------------------------------------- :     -
AOTVOKIR2DS5*01v2   : ----------------------------------------------------------------------------------------------- :     -
AOTVOKIR3DS7*01     : ----------------------------------------------------------------------------------------------- :     -
AOTVOKIR3DS7*01v1   : ----------------------------------------------------------------------------------------------- :     -
AOTVOKIR3DL8*01     : ----------------------------------------------------------------------------------------------- :     -
AOTVOKIR3DL8*01v1   : ----------------------------------------------------------------------------------------------- :     -
AOTVOKIR3DS8*01v2   : ----------------------------------------------------------------------------------------------- :     -
AOTVOKIR3DL8*02     : ----------------------------------------------------------------------------------------------- :     -
AOTVOKIR4DL9*01     : ----------------------------------------------------------------------------------------------- :     -
AOTVOKIR4DS9*01v1   : ----------------------------------------------------------------------------------------------- :     -
AOTVOKIR3DL9*01v2   : ----------------------------------------------------------------------------------------------- :     -
AOTVOKIR4DL9*02     : ----------------------------------------------------------------------------------------------- :     -
AOTVOKIR4DL10*01    : ----------------------------------------------------------------------------------------------- :     -
AOTVOKIR3DL10*01v1  : ----------------------------------------------------------------------------------------------- :     -
AOTVOKIR4DS10*01v2  : ----------------------------------------------------------------------------------------------- :     -
AOTVOKIR3DS10*01v3  : ----------------------------------------------------------------------------------------------- :     -
AOTVOKIR3DS10*01v4  : ----------------------------------------------------------------------------------------------- :     -

                          *        9800         *        9820         *        9840         *        9860         *        9880
BAC_clone_Om        : TGCTTCGTTTGATGCAATCCCAATGGTCTATTTTTTGCTTTGATTACTTGTGTTTTTAAGGTTTTAAACAAAATGTCTTTTCTCATGCAAATGTC :  9880
```

```
AOTVOKIR3DL4*01    : ----------------------------------------------------------------------------------------------- :     -
AOTVOKIR3DL4*02    : ----------------------------------------------------------------------------------------------- :     -
AOTVOKIR3DL4*02v1  : ----------------------------------------------------------------------------------------------- :     -
AOTVOKIR3DL4*02v2  : ----------------------------------------------------------------------------------------------- :     -
AOTVOKIR3DL4*03    : ----------------------------------------------------------------------------------------------- :     -
AOTVOKIR3DS4*04    : ----------------------------------------------------------------------------------------------- :     -
AOTVOKIR3DS4*04v1  : ----------------------------------------------------------------------------------------------- :     -
AOTVOKIR3DS5*01    : ----------------------------------------------------------------------------------------------- :     -
AOTVOKIR2DS5*01v1  : ----------------------------------------------------------------------------------------------- :     -
AOTVOKIR2DS5*01v2  : ----------------------------------------------------------------------------------------------- :     -
AOTVOKIR3DS7*01    : ----------------------------------------------------------------------------------------------- :     -
AOTVOKIR3DS7*01v1  : ----------------------------------------------------------------------------------------------- :     -
AOTVOKIR3DL8*01    : ----------------------------------------------------------------------------------------------- :     -
AOTVOKIR3DL8*01v1  : ----------------------------------------------------------------------------------------------- :     -
AOTVOKIR3DS8*01v2  : ----------------------------------------------------------------------------------------------- :     -
AOTVOKIR3DL8*02    : ----------------------------------------------------------------------------------------------- :     -
AOTVOKIR4DL9*01    : ----------------------------------------------------------------------------------------------- :     -
AOTVOKIR4DS9*01v1  : ----------------------------------------------------------------------------------------------- :     -
AOTVOKIR3DL9*01v2  : ----------------------------------------------------------------------------------------------- :     -
AOTVOKIR4DL9*02    : ----------------------------------------------------------------------------------------------- :     -
AOTVOKIR4DL10*01   : ----------------------------------------------------------------------------------------------- :     -
AOTVOKIR3DL10*01v1 : ----------------------------------------------------------------------------------------------- :     -
AOTVOKIR4DS10*01v2 : ----------------------------------------------------------------------------------------------- :     -
AOTVOKIR3DS10*01v3 : ----------------------------------------------------------------------------------------------- :     -
AOTVOKIR3DS10*01v4 : ----------------------------------------------------------------------------------------------- :     -

                              *      9900         *      9920         *      9940         *      9960         *
BAC_clone_Om       : CTGGAGCATTTCCCCAGTGTTTTCTTCTACACATTTCATAGGTTCAGGCCTTAGATGCACGTCTTTCATCCATTTTCATTTGATTTTTGTGTATG :  9975
AOTVOKIR3DL4*01    : ----------------------------------------------------------------------------------------------- :     -
AOTVOKIR3DL4*02    : ----------------------------------------------------------------------------------------------- :     -
AOTVOKIR3DL4*02v1  : ----------------------------------------------------------------------------------------------- :     -
AOTVOKIR3DL4*02v2  : ----------------------------------------------------------------------------------------------- :     -
AOTVOKIR3DL4*03    : ----------------------------------------------------------------------------------------------- :     -
AOTVOKIR3DS4*04    : ----------------------------------------------------------------------------------------------- :     -
AOTVOKIR3DS4*04v1  : ----------------------------------------------------------------------------------------------- :     -
AOTVOKIR3DS5*01    : ----------------------------------------------------------------------------------------------- :     -
AOTVOKIR2DS5*01v1  : ----------------------------------------------------------------------------------------------- :     -
AOTVOKIR2DS5*01v2  : ----------------------------------------------------------------------------------------------- :     -
AOTVOKIR3DS7*01    : ----------------------------------------------------------------------------------------------- :     -
AOTVOKIR3DS7*01v1  : ----------------------------------------------------------------------------------------------- :     -
AOTVOKIR3DL8*01    : ----------------------------------------------------------------------------------------------- :     -
AOTVOKIR3DL8*01v1  : ----------------------------------------------------------------------------------------------- :     -
AOTVOKIR3DS8*01v2  : ----------------------------------------------------------------------------------------------- :     -
AOTVOKIR3DL8*02    : ----------------------------------------------------------------------------------------------- :     -
AOTVOKIR4DL9*01    : ----------------------------------------------------------------------------------------------- :     -
```

```
AOTVOKIR4DS9*01v1   : ----------------------------------------------------------------------------------------------- :     -
AOTVOKIR3DL9*01v2   : ----------------------------------------------------------------------------------------------- :     -
AOTVOKIR4DL9*02     : ----------------------------------------------------------------------------------------------- :     -
AOTVOKIR4DL10*01    : ----------------------------------------------------------------------------------------------- :     -
AOTVOKIR3DL10*01v1  : ----------------------------------------------------------------------------------------------- :     -
AOTVOKIR4DS10*01v2  : ----------------------------------------------------------------------------------------------- :     -
AOTVOKIR3DS10*01v3  : ----------------------------------------------------------------------------------------------- :     -
AOTVOKIR3DS10*01v4  : ----------------------------------------------------------------------------------------------- :     -

                         9980         *        10000         *        10020         *        10040         *        10060         *
BAC_clone_Om        : CTGAGAGGTAGAGGTGCAGTTTCATCCCTGCACTGTTTATTGAAAAGACTGTCCTTTCCTGAGTGTAGGTTCTTAGCACCCTTGTCAAAGTCCAT : 10070
AOTVOKIR3DL4*01     : ----------------------------------------------------------------------------------------------- :     -
AOTVOKIR3DL4*02     : ----------------------------------------------------------------------------------------------- :     -
AOTVOKIR3DL4*02v1   : ----------------------------------------------------------------------------------------------- :     -
AOTVOKIR3DL4*02v2   : --------------------------------------------------------------------GTTCCTGGCACCCTTGTCAAAGTCCAT :   973
AOTVOKIR3DL4*03     : ----------------------------------------------------------------------------------------------- :     -
AOTVOKIR3DS4*04     : ----------------------------------------------------------------------------------------------- :     -
AOTVOKIR3DS4*04v1   : ----------------------------------------------------------------------------------------------- :     -
AOTVOKIR3DS5*01     : ----------------------------------------------------------------------------------------------- :     -
AOTVOKIR2DS5*01v1   : ----------------------------------------------------------------------------------------------- :     -
AOTVOKIR2DS5*01v2   : ----------------------------------------------------------------------------------------------- :     -
AOTVOKIR3DS7*01     : ----------------------------------------------------------------------------------------------- :     -
AOTVOKIR3DS7*01v1   : ----------------------------------------------------------------------------------------------- :     -
AOTVOKIR3DL8*01     : ----------------------------------------------------------------------------------------------- :     -
AOTVOKIR3DL8*01v1   : ----------------------------------------------------------------------------------------------- :     -
AOTVOKIR3DS8*01v2   : ----------------------------------------------------------------------------------------------- :     -
AOTVOKIR3DL8*02     : ----------------------------------------------------------------------------------------------- :     -
AOTVOKIR4DL9*01     : ----------------------------------------------------------------------------------------------- :     -
AOTVOKIR4DS9*01v1   : ----------------------------------------------------------------------------------------------- :     -
AOTVOKIR3DL9*01v2   : ----------------------------------------------------------------------------------------------- :     -
AOTVOKIR4DL9*02     : ----------------------------------------------------------------------------------------------- :     -
AOTVOKIR4DL10*01    : ----------------------------------------------------------------------------------------------- :     -
AOTVOKIR3DL10*01v1  : ----------------------------------------------------------------------------------------------- :     -
AOTVOKIR4DS10*01v2  : ----------------------------------------------------------------------------------------------- :     -
AOTVOKIR3DS10*01v3  : ----------------------------------------------------------------------------------------------- :     -
AOTVOKIR3DS10*01v4  : ----------------------------------------------------------------------------------------------- :     -

                             10080         *        10100         *        10120         *        10140         *        10160
BAC_clone_Om        : TGGATGGGCTGTGCATGGTGGCTCACACCTGCAATCCCAGCACTTTGGGAGACCAAGGTAGGTGGATCACCTGTGGCCAGGAGTTCGAGACCAGC : 10165
AOTVOKIR3DL4*01     : ----------------------------------------------------------------------------------------------- :     -
AOTVOKIR3DL4*02     : ----------------------------------------------------------------------------------------------- :     -
AOTVOKIR3DL4*02v1   : ----------------------------------------------------------------------------------------------- :     -
AOTVOKIR3DL4*02v2   : TGGATGGGCTGTGCATGGTGGCTCACGCCTGCAATCCCAGCACTTTGGGAGACCAAG-------------------------------------- :  1030
AOTVOKIR3DL4*03     : ----------------------------------------------------------------------------------------------- :     -
```

```
AOTVOKIR3DS4*04     : ----------------------------------------------------------------------------------------------- :      -
AOTVOKIR3DS4*04v1   : ----------------------------------------------------------------------------------------------- :      -
AOTVOKIR3DS5*01     : ----------------------------------------------------------------------------------------------- :      -
AOTVOKIR2DS5*01v1   : ----------------------------------------------------------------------------------------------- :      -
AOTVOKIR2DS5*01v2   : ----------------------------------------------------------------------------------------------- :      -
AOTVOKIR3DS7*01     : ----------------------------------------------------------------------------------------------- :      -
AOTVOKIR3DS7*01v1   : ----------------------------------------------------------------------------------------------- :      -
AOTVOKIR3DL8*01     : ----------------------------------------------------------------------------------------------- :      -
AOTVOKIR3DL8*01v1   : ----------------------------------------------------------------------------------------------- :      -
AOTVOKIR3DS8*01v2   : ----------------------------------------------------------------------------------------------- :      -
AOTVOKIR3DL8*02     : ----------------------------------------------------------------------------------------------- :      -
AOTVOKIR4DL9*01     : ----------------------------------------------------------------------------------------------- :      -
AOTVOKIR4DS9*01v1   : ----------------------------------------------------------------------------------------------- :      -
AOTVOKIR3DL9*01v2   : ----------------------------------------------------------------------------------------------- :      -
AOTVOKIR4DL9*02     : ----------------------------------------------------------------------------------------------- :      -
AOTVOKIR4DL10*01    : ----------------------------------------------------------------------------------------------- :      -
AOTVOKIR3DL10*01v1  : ----------------------------------------------------------------------------------------------- :      -
AOTVOKIR4DS10*01v2  : ----------------------------------------------------------------------------------------------- :      -
AOTVOKIR3DS10*01v3  : ----------------------------------------------------------------------------------------------- :      -
AOTVOKIR3DS10*01v4  : ----------------------------------------------------------------------------------------------- :      -

                          *      10180         *      10200         *      10220         *      10240         *      10260
BAC_clone_Om        : CTGGCCGACATGGTGAAACCCCTTCTCCACTAAAAGTACAAAACTCAGCTGAGCATAGTGGTTGGTGCCTGTTATACCACTAGTCAGGTGTCTGA : 10260
AOTVOKIR3DL4*01     : ----------------------------------------------------------------------------------------------- :      -
AOTVOKIR3DL4*02     : ----------------------------------------------------------------------------------------------- :      -
AOTVOKIR3DL4*02v1   : ----------------------------------------------------------------------------------------------- :      -
AOTVOKIR3DL4*02v2   : ----------------------------------------------------------------------------------------------- :      -
AOTVOKIR3DL4*03     : ----------------------------------------------------------------------------------------------- :      -
AOTVOKIR3DS4*04     : ----------------------------------------------------------------------------------------------- :      -
AOTVOKIR3DS4*04v1   : ----------------------------------------------------------------------------------------------- :      -
AOTVOKIR3DS5*01     : ----------------------------------------------------------------------------------------------- :      -
AOTVOKIR2DS5*01v1   : ----------------------------------------------------------------------------------------------- :      -
AOTVOKIR2DS5*01v2   : ----------------------------------------------------------------------------------------------- :      -
AOTVOKIR3DS7*01     : ----------------------------------------------------------------------------------------------- :      -
AOTVOKIR3DS7*01v1   : ----------------------------------------------------------------------------------------------- :      -
AOTVOKIR3DL8*01     : ----------------------------------------------------------------------------------------------- :      -
AOTVOKIR3DL8*01v1   : ----------------------------------------------------------------------------------------------- :      -
AOTVOKIR3DS8*01v2   : ----------------------------------------------------------------------------------------------- :      -
AOTVOKIR3DL8*02     : ----------------------------------------------------------------------------------------------- :      -
AOTVOKIR4DL9*01     : ----------------------------------------------------------------------------------------------- :      -
AOTVOKIR4DS9*01v1   : ----------------------------------------------------------------------------------------------- :      -
AOTVOKIR3DL9*01v2   : ----------------------------------------------------------------------------------------------- :      -
AOTVOKIR4DL9*02     : ----------------------------------------------------------------------------------------------- :      -
AOTVOKIR4DL10*01    : ----------------------------------------------------------------------------------------------- :      -
AOTVOKIR3DL10*01v1  : ----------------------------------------------------------------------------------------------- :      -
```

```
AOTVOKIR4DS10*01v2 : ----------------------------------------------------------------------------------------------- :     -
AOTVOKIR3DS10*01v3 : ----------------------------------------------------------------------------------------------- :     -
AOTVOKIR3DS10*01v4 : ----------------------------------------------------------------------------------------------- :     -

                              *       10280          *       10300          *       10320          *       10340          *
BAC_clone_Om       : GGCAAGAGACTTGCTTGAAACCAAGAGATGGAGGTTGCAGTGACCTGAAATTGCACCTCTGCACTACAGCCTGGGTGACAGAGCGAGATTCCATC : 10355
AOTVOKIR3DL4*01    : ----------------------------------------------------------------------------------------------- :     -
AOTVOKIR3DL4*02    : ----------------------------------------------------------------------------------------------- :     -
AOTVOKIR3DL4*02v1  : ----------------------------------------------------------------------------------------------- :     -
AOTVOKIR3DL4*02v2  : ----------------------------------------------------------------------------------------------- :     -
AOTVOKIR3DL4*03    : ----------------------------------------------------------------------------------------------- :     -
AOTVOKIR3DS4*04    : ----------------------------------------------------------------------------------------------- :     -
AOTVOKIR3DS4*04v1  : ----------------------------------------------------------------------------------------------- :     -
AOTVOKIR3DS5*01    : ----------------------------------------------------------------------------------------------- :     -
AOTVOKIR2DS5*01v1  : ----------------------------------------------------------------------------------------------- :     -
AOTVOKIR2DS5*01v2  : ----------------------------------------------------------------------------------------------- :     -
AOTVOKIR3DS7*01    : ----------------------------------------------------------------------------------------------- :     -
AOTVOKIR3DS7*01v1  : ----------------------------------------------------------------------------------------------- :     -
AOTVOKIR3DL8*01    : ----------------------------------------------------------------------------------------------- :     -
AOTVOKIR3DL8*01v1  : ----------------------------------------------------------------------------------------------- :     -
AOTVOKIR3DS8*01v2  : ----------------------------------------------------------------------------------------------- :     -
AOTVOKIR3DL8*02    : ----------------------------------------------------------------------------------------------- :     -
AOTVOKIR4DL9*01    : ----------------------------------------------------------------------------------------------- :     -
AOTVOKIR4DS9*01v1  : ----------------------------------------------------------------------------------------------- :     -
AOTVOKIR3DL9*01v2  : ----------------------------------------------------------------------------------------------- :     -
AOTVOKIR4DL9*02    : ----------------------------------------------------------------------------------------------- :     -
AOTVOKIR4DL10*01   : ----------------------------------------------------------------------------------------------- :     -
AOTVOKIR3DL10*01v1 : ----------------------------------------------------------------------------------------------- :     -
AOTVOKIR4DS10*01v2 : ----------------------------------------------------------------------------------------------- :     -
AOTVOKIR3DS10*01v3 : ----------------------------------------------------------------------------------------------- :     -
AOTVOKIR3DS10*01v4 : ----------------------------------------------------------------------------------------------- :     -

                     10360          *       10380          *       10400          *       10420          *       10440          *
BAC_clone_Om       : TCAAAAAAAAAAAAAAAAAAAGCCATTGGATGTAAATGCATGGGTTATGTCTGTGTTCTTCATTCTCCTCCATTGTTCTATGAGCCTTTCTTTAT : 10450
AOTVOKIR3DL4*01    : ----------------------------------------------------------------------------------------------- :     -
AOTVOKIR3DL4*02    : ----------------------------------------------------------------------------------------------- :     -
AOTVOKIR3DL4*02v1  : ----------------------------------------------------------------------------------------------- :     -
AOTVOKIR3DL4*02v2  : ----------------------------------------------------------------------------------------------- :     -
AOTVOKIR3DL4*03    : ----------------------------------------------------------------------------------------------- :     -
AOTVOKIR3DS4*04    : ----------------------------------------------------------------------------------------------- :     -
AOTVOKIR3DS4*04v1  : ----------------------------------------------------------------------------------------------- :     -
AOTVOKIR3DS5*01    : ----------------------------------------------------------------------------------------------- :     -
AOTVOKIR2DS5*01v1  : ----------------------------------------------------------------------------------------------- :     -
AOTVOKIR2DS5*01v2  : ----------------------------------------------------------------------------------------------- :     -
```

```
AOTVOKIR3DS7*01     : ----------------------------------------------------------------------------------------------- :     -
AOTVOKIR3DS7*01v1   : ----------------------------------------------------------------------------------------------- :     -
AOTVOKIR3DL8*01     : ----------------------------------------------------------------------------------------------- :     -
AOTVOKIR3DL8*01v1   : ----------------------------------------------------------------------------------------------- :     -
AOTVOKIR3DS8*01v2   : ----------------------------------------------------------------------------------------------- :     -
AOTVOKIR3DL8*02     : ----------------------------------------------------------------------------------------------- :     -
AOTVOKIR4DL9*01     : ----------------------------------------------------------------------------------------------- :     -
AOTVOKIR4DS9*01v1   : ----------------------------------------------------------------------------------------------- :     -
AOTVOKIR3DL9*01v2   : ----------------------------------------------------------------------------------------------- :     -
AOTVOKIR4DL9*02     : ----------------------------------------------------------------------------------------------- :     -
AOTVOKIR4DL10*01    : ----------------------------------------------------------------------------------------------- :     -
AOTVOKIR3DL10*01v1  : ----------------------------------------------------------------------------------------------- :     -
AOTVOKIR4DS10*01v2  : ----------------------------------------------------------------------------------------------- :     -
AOTVOKIR3DS10*01v3  : ----------------------------------------------------------------------------------------------- :     -
AOTVOKIR3DS10*01v4  : ----------------------------------------------------------------------------------------------- :     -

                           10460         *     10480         *     10500         *     10520         *     10540
BAC_clone_Om        : GCCAGCATCATGCTGTTTTGCTTACTACAGCTCTGTAACATATTTTTAAGTCAGGTAGTGTGATGGCCTGTTTTCTCATTATACCTCAAAGTCTC : 10545
AOTVOKIR3DL4*01     : ----------------------------------------------------------------------------------------------- :     -
AOTVOKIR3DL4*02     : ----------------------------------------------------------------------------------------------- :     -
AOTVOKIR3DL4*02v1   : ----------------------------------------------------------------------------------------------- :     -
AOTVOKIR3DL4*02v2   : ----------------------------------------------------------------------------------------------- :     -
AOTVOKIR3DL4*03     : ----------------------------------------------------------------------------------------------- :     -
AOTVOKIR3DS4*04     : ----------------------------------------------------------------------------------------------- :     -
AOTVOKIR3DS4*04v1   : ----------------------------------------------------------------------------------------------- :     -
AOTVOKIR3DS5*01     : ----------------------------------------------------------------------------------------------- :     -
AOTVOKIR2DS5*01v1   : ----------------------------------------------------------------------------------------------- :     -
AOTVOKIR2DS5*01v2   : ----------------------------------------------------------------------------------------------- :     -
AOTVOKIR3DS7*01     : ----------------------------------------------------------------------------------------------- :     -
AOTVOKIR3DS7*01v1   : -------------------------------------------------------------------------------------------TCTC :   950
AOTVOKIR3DL8*01     : ----------------------------------------------------------------------------------------------- :     -
AOTVOKIR3DL8*01v1   : ----------------------------------------------------------------------------------------------- :     -
AOTVOKIR3DS8*01v2   : ----------------------------------------------------------------------------------------------- :     -
AOTVOKIR3DL8*02     : ----------------------------------------------------------------------------------------------- :     -
AOTVOKIR4DL9*01     : ----------------------------------------------------------------------------------------------- :     -
AOTVOKIR4DS9*01v1   : ----------------------------------------------------------------------------------------------- :     -
AOTVOKIR3DL9*01v2   : ----------------------------------------------------------------------------------------------- :     -
AOTVOKIR4DL9*02     : ----------------------------------------------------------------------------------------------- :     -
AOTVOKIR4DL10*01    : ----------------------------------------------------------------------------------------------- :     -
AOTVOKIR3DL10*01v1  : ----------------------------------------------------------------------------------------------- :     -
AOTVOKIR4DS10*01v2  : ----------------------------------------------------------------------------------------------- :     -
AOTVOKIR3DS10*01v3  : ----------------------------------------------------------------------------------------------- :     -
AOTVOKIR3DS10*01v4  : ----------------------------------------------------------------------------------------------- :     -
```

```
                                 *       10560          *       10580          *       10600          *       10620          *       10640
BAC_clone_Om          : AGGACAGTGGGCATCAGGTACAATGATTATGGAGAAGGGGATGCCAGGACTTCCAGGGCCCAACATTAGATAACAGAATGTTGGCCATGAACCAA : 10640
AOTVOKIR3DL4*01       : ----------------------------------------------------------------------------------------------- :     -
AOTVOKIR3DL4*02       : ----------------------------------------------------------------------------------------------- :     -
AOTVOKIR3DL4*02v1     : ----------------------------------------------------------------------------------------------- :     -
AOTVOKIR3DL4*02v2     : ----------------------------------------------------------------------------------------------- :     -
AOTVOKIR3DL4*03       : ----------------------------------------------------------------------------------------------- :     -
AOTVOKIR3DS4*04       : ----------------------------------------------------------------------------------------------- :     -
AOTVOKIR3DS4*04v1     : ----------------------------------------------------------------------------------------------- :     -
AOTVOKIR3DS5*01       : ----------------------------------------------------------------------------------------------- :     -
AOTVOKIR2DS5*01v1     : ----------------------------------------------------------------------------------------------- :     -
AOTVOKIR2DS5*01v2     : ----------------------------------------------------------------------------------------------- :     -
AOTVOKIR3DS7*01       : ----------------------------------------------------------------------------------------------- :     -
AOTVOKIR3DS7*01v1     : AGGACAGTGGGCATCAGGTACAATGATTATGGAGAAGGGGATGCCAGGACTCCCAGG-CCCAACATTAGATAACAGAGTGTTGGCCATGAACAAA :  1044
AOTVOKIR3DL8*01       : ----------------------------------------------------------------------------------------------- :     -
AOTVOKIR3DL8*01v1     : ----------------------------------------------------------------------------------------------- :     -
AOTVOKIR3DS8*01v2     : ----------------------------------------------------------------------------------------------- :     -
AOTVOKIR3DL8*02       : ----------------------------------------------------------------------------------------------- :     -
AOTVOKIR4DL9*01       : ----------------------------------------------------------------------------------------------- :     -
AOTVOKIR4DS9*01v1     : ----------------------------------------------------------------------------------------------- :     -
AOTVOKIR3DL9*01v2     : ----------------------------------------------------------------------------------------------- :     -
AOTVOKIR4DL9*02       : ----------------------------------------------------------------------------------------------- :     -
AOTVOKIR4DL10*01      : ----------------------------------------------------------------------------------------------- :     -
AOTVOKIR3DL10*01v1    : ----------------------------------------------------------------------------------------------- :     -
AOTVOKIR4DS10*01v2    : ----------------------------------------------------------------------------------------------- :     -
AOTVOKIR3DS10*01v3    : ----------------------------------------------------------------------------------------------- :     -
AOTVOKIR3DS10*01v4    : ----------------------------------------------------------------------------------------------- :     -

                                                                                                             EXON 6
                                      *       10660          *       10680          *       10700          *       10720          *
BAC_clone_Om          : CCTCAGAGATTTCCACTGAGTAGAAGACAGGCATCCTCATTGCCACACCTCTCTCCTGTCCCGTGTTCTAGGAAACCCTTCAAGCGGTTGGCCAT : 10735
AOTVOKIR3DL4*01       : -----------------------------------------------------------------------GAAACCCTTCAAGCGGTTGGCTAT :   970
AOTVOKIR3DL4*02       : -----------------------------------------------------------------------GAAACCCTTCAAGCGGTTGGCTAT :   970
AOTVOKIR3DL4*02v1     : -----------------------------------------------------------------------GAAACCCTTCAAGCGGTTGGCTAT :   934
AOTVOKIR3DL4*02v2     : -----------------------------------------------------------------------GAAACCCTTCAAGCGGTTGGCTAT :  1054
AOTVOKIR3DL4*03       : -----------------------------------------------------------------------GAAACCCTTCAAGCGGTTGGCTAT :   970
AOTVOKIR3DS4*04       : -----------------------------------------------------------------------GAAACCCTTCAAGCGGTTGGCTAT :   970
AOTVOKIR3DS4*04v1     : -----------------------------------------------------------------------GAAACCCTTCAAGCGGTTGGCTAT :   970
AOTVOKIR3DS5*01       : -----------------------------------------------------------------------GAAACCCCTCAAGCAGTTGGCCAT :   970
AOTVOKIR2DS5*01v1     : -----------------------------------------------------------------------GAAACCCCTCAAGCAGTTGGCCAT :   772
AOTVOKIR2DS5*01v2     : -----------------------------------------------------------------------GAAACCCCTCAAGCAGTTGGCCAT :   685
AOTVOKIR3DS7*01       : -----------------------------------------------------------------------GACACCCTTCAAGCAGTTGGCCAT :   970
AOTVOKIR3DS7*01v1     : CCTCAGAGATTTCCACTGAGTAGAAGACAGGCATCCTCATTGCCACACCTCTCTCCTGTCCCGTGTTCTAGGACACCCTTCAAGCAGTTGGCCAT :  1139
AOTVOKIR3DL8*01       : -----------------------------------------------------------------------GAAACCCTTCAAGCAGTTGGCCAT :   970
AOTVOKIR3DL8*01v1     : ----------------------------------------------------------------------------------------------- :     -
AOTVOKIR3DS8*01v2     : -----------------------------------------------------------------------GAAACCCTTCAAGCAGTTGGCCAT :   970
```

```
AOTVOKIR3DL8*02      : -----------------------------------------------------------------------GAAACCCTTCAAGCAGTTGGCCAT :   970
AOTVOKIR4DL9*01      : -----------------------------------------------------------------------GAAACCCTTCAAGCAGTTGGCCGT :  1255
AOTVOKIR4DS9*01v1    : ----------------------------------------------------------------------------------------------- :     -
AOTVOKIR3DL9*01v2    : -----------------------------------------------------------------------GAAACCCTTCAAGCAGTTGGCCGT :  1057
AOTVOKIR4DL9*02      : -----------------------------------------------------------------------GAAACCCTTCAAGCAGTTGGCCGT :  1255
AOTVOKIR4DL10*01     : -----------------------------------------------------------------------GAAACCCTTCAAGCAGTTGGCCAT :  1255
AOTVOKIR3DL10*01v1   : -----------------------------------------------------------------------GAAACCCTTCAAGCAGTTGGCCAT :  1054
AOTVOKIR4DS10*01v2   : -----------------------------------------------------------------------GAAACCCTTCAAGCAGTTGGCCAT :  1255
AOTVOKIR3DS10*01v3   : -----------------------------------------------------------------------GAAACCCTTCAAGCAGTTGGCCAT :   970
AOTVOKIR3DS10*01v4   : ----------------------------------------------------------------------------------------------- :     -
                                                                                              gaaaccc tcaagc gttggc  t

                          10740         *       10760          *       10780          *       10800         *       10820         *
BAC_clone_Om         : CACCCACTGAACCAAGCTCCAAAACTGGTGAGTAAAGGACCCCTCTTCTCTCTGCTTTTGGAAACCTGGGGAGGTGGAAGCCTTGGATTCCAGTG : 10830
AOTVOKIR3DL4*01      : CACCCACTGAACCAAGCTCCAAAACTG-------------------------------------------------------------------- :   997
AOTVOKIR3DL4*02      : CACCCACTGAACCAAGCTCCAAAACTG-------------------------------------------------------------------- :   997
AOTVOKIR3DL4*02v1    : CACCCACTGAACCAAGCTCCAAAACTG-------------------------------------------------------------------- :   961
AOTVOKIR3DL4*02v2    : CACCCACTGAACCAAGCTCCAAAACTG-------------------------------------------------------------------- :  1081
AOTVOKIR3DL4*03      : CACCCACTGAACCAAGCTCCAAAACTG-------------------------------------------------------------------- :   997
AOTVOKIR3DS4*04      : CACCCACTGAACCAAGCTCCAAAACTG-------------------------------------------------------------------- :   997
AOTVOKIR3DS4*04v1    : CACCCACTGAACCAAGCTCCAAAACTG-------------------------------------------------------------------- :   997
AOTVOKIR3DS5*01      : CACCCACTGAACCAGGCTCCAAAACTG-------------------------------------------------------------------- :   997
AOTVOKIR2DS5*01v1    : CACCCACTGAACCAGGCTCCAAAACTG-------------------------------------------------------------------- :   799
AOTVOKIR2DS5*01v2    : CACCCACTGAACCAGGCTCCAAAACTG-------------------------------------------------------------------- :   712
AOTVOKIR3DS7*01      : CACCCACAGAACCAAGCTCCAAAACTG-------------------------------------------------------------------- :   997
AOTVOKIR3DS7*01v1    : CACCCACAGAACCAAGCTCCAAAACTG-------------------------------------------------------------------- :  1166
AOTVOKIR3DL8*01      : CACCCACTGAACCAGGCTCCAAAACTG-------------------------------------------------------------------- :   997
AOTVOKIR3DL8*01v1    : ----------------------------------------------------------------------------------------------- :     -
AOTVOKIR3DS8*01v2    : CACCCACTGAACCAGGCTCCAAAACTG-------------------------------------------------------------------- :   997
AOTVOKIR3DL8*02      : CACCCACTGAACCAGGCTCCAAAACTC-------------------------------------------------------------------- :   997
AOTVOKIR4DL9*01      : CACCCACGGAACCAAGCTCCAAAACTG-------------------------------------------------------------------- :  1282
AOTVOKIR4DS9*01v1    : ----------------------------------------------------------------------------------------------- :     -
AOTVOKIR3DL9*01v2    : CACCCACGGAACCAAGCTCCAAAACTG-------------------------------------------------------------------- :  1084
AOTVOKIR4DL9*02      : CACCCACGGAACCAAGCTCCAAAACTG-------------------------------------------------------------------- :  1282
AOTVOKIR4DL10*01     : CACCCACGGAACCAAGCTCCAAAACTG-------------------------------------------------------------------- :  1282
AOTVOKIR3DL10*01v1   : CACCCACGGAACCAAGCTCCAAAACTG-------------------------------------------------------------------- :  1081
AOTVOKIR4DS10*01v2   : CACCCACGGAACCAAGCTCCAAAACTG-------------------------------------------------------------------- :  1282
AOTVOKIR3DS10*01v3   : CACCCACGGAACCAAGCTCCAAAACTG-------------------------------------------------------------------- :   997
AOTVOKIR3DS10*01v4   : ----------------------------------------------------------------------------------------------- :     -
                       cacccac gaacca gctccaaaactg

                               10840         *       10860         *       10880         *       10900         *       10920
BAC_clone_Om         : TTGGCTCAGCACCTCCCAGCTCTGTGATTGAGGGCCTGTCTTCTACCATCTCTGAACCCCAGACACTCCAACAGTGCAAGGATCTGGGCCCAAC : 10925
AOTVOKIR3DL4*01      : ----------------------------------------------------------------------------------------------- :     -
AOTVOKIR3DL4*02      : ----------------------------------------------------------------------------------------------- :     -
AOTVOKIR3DL4*02v1    : ----------------------------------------------------------------------------------------------- :     -
```

```
AOTVOKIR3DL4*02v2   : ----------------------------------------------------------------------------------------------- :     -
AOTVOKIR3DL4*03     : ----------------------------------------------------------------------------------------------- :     -
AOTVOKIR3DS4*04     : ----------------------------------------------------------------------------------------------- :     -
AOTVOKIR3DS4*04v1   : ----------------------------------------------------------------------------------------------- :     -
AOTVOKIR3DS5*01     : ----------------------------------------------------------------------------------------------- :     -
AOTVOKIR2DS5*01v1   : ----------------------------------------------------------------------------------------------- :     -
AOTVOKIR2DS5*01v2   : ----------------------------------------------------------------------------------------------- :     -
AOTVOKIR3DS7*01     : ----------------------------------------------------------------------------------------------- :     -
AOTVOKIR3DS7*01v1   : ----------------------------------------------------------------------------------------------- :     -
AOTVOKIR3DL8*01     : ----------------------------------------------------------------------------------------------- :     -
AOTVOKIR3DL8*01v1   : ----------------------------------------------------------------------------------------------- :     -
AOTVOKIR3DS8*01v2   : ----------------------------------------------------------------------------------------------- :     -
AOTVOKIR3DL8*02     : ----------------------------------------------------------------------------------------------- :     -
AOTVOKIR4DL9*01     : ----------------------------------------------------------------------------------------------- :     -
AOTVOKIR4DS9*01v1   : ----------------------------------------------------------------------------------------------- :     -
AOTVOKIR3DL9*01v2   : ----------------------------------------------------------------------------------------------- :     -
AOTVOKIR4DL9*02     : ----------------------------------------------------------------------------------------------- :     -
AOTVOKIR4DL10*01    : ----------------------------------------------------------------------------------------------- :     -
AOTVOKIR3DL10*01v1  : ----------------------------------------------------------------------------------------------- :     -
AOTVOKIR4DS10*01v2  : ----------------------------------------------------------------------------------------------- :     -
AOTVOKIR3DS10*01v3  : ----------------------------------------------------------------------------------------------- :     -
AOTVOKIR3DS10*01v4  : ----------------------------------------------------------------------------------------------- :     -

                          *      10940        *      10960        *      10980        *      11000        *      11020
BAC_clone_Om        : ACGAGGCTCAGTGACGTCTCTTAATCTCTAATTTTCTGCAGCTGAGACCTCCTTGAGGCTAGAAGAATGATTGCAAATCTGACATCCTTCTCAGG : 11020
AOTVOKIR3DL4*01     : ----------------------------------------------------------------------------------------------- :     -
AOTVOKIR3DL4*02     : ----------------------------------------------------------------------------------------------- :     -
AOTVOKIR3DL4*02v1   : ----------------------------------------------------------------------------------------------- :     -
AOTVOKIR3DL4*02v2   : ----------------------------------------------------------------------------------------------- :     -
AOTVOKIR3DL4*03     : ----------------------------------------------------------------------------------------------- :     -
AOTVOKIR3DS4*04     : ----------------------------------------------------------------------------------------------- :     -
AOTVOKIR3DS4*04v1   : ----------------------------------------------------------------------------------------------- :     -
AOTVOKIR3DS5*01     : ----------------------------------------------------------------------------------------------- :     -
AOTVOKIR2DS5*01v1   : ----------------------------------------------------------------------------------------------- :     -
AOTVOKIR2DS5*01v2   : ----------------------------------------------------------------------------------------------- :     -
AOTVOKIR3DS7*01     : ----------------------------------------------------------------------------------------------- :     -
AOTVOKIR3DS7*01v1   : ----------------------------------------------------------------------------------------------- :     -
AOTVOKIR3DL8*01     : ----------------------------------------------------------------------------------------------- :     -
AOTVOKIR3DL8*01v1   : ----------------------------------------------------------------------------------------------- :     -
AOTVOKIR3DS8*01v2   : ----------------------------------------------------------------------------------------------- :     -
AOTVOKIR3DL8*02     : ----------------------------------------------------------------------------------------------- :     -
AOTVOKIR4DL9*01     : ----------------------------------------------------------------------------------------------- :     -
AOTVOKIR4DS9*01v1   : ----------------------------------------------------------------------------------------------- :     -
AOTVOKIR3DL9*01v2   : ----------------------------------------------------------------------------------------------- :     -
AOTVOKIR4DL9*02     : ----------------------------------------------------------------------------------------------- :     -
```

```
AOTVOKIR4DL10*01   : ----------------------------------------------------------------------------------------------- :     -
AOTVOKIR3DL10*01v1 : ----------------------------------------------------------------------------------------------- :     -
AOTVOKIR4DS10*01v2 : ----------------------------------------------------------------------------------------------- :     -
AOTVOKIR3DS10*01v3 : ----------------------------------------------------------------------------------------------- :     -
AOTVOKIR3DS10*01v4 : ----------------------------------------------------------------------------------------------- :     -

                              *     11040         *     11060         *     11080         *     11100         *
BAC_clone_Om       : AAAAAATGCAGTGTTTGTTCTGCCTGCATTCCTAACTGGAGGATAAATGCCTGGGGGGCTTGGGGGAGGGAAAGGAAGGGAACATCTGATGAGGG : 11115
AOTVOKIR3DL4*01    : ----------------------------------------------------------------------------------------------- :     -
AOTVOKIR3DL4*02    : ----------------------------------------------------------------------------------------------- :     -
AOTVOKIR3DL4*02v1  : ----------------------------------------------------------------------------------------------- :     -
AOTVOKIR3DL4*02v2  : ----------------------------------------------------------------------------------------------- :     -
AOTVOKIR3DL4*03    : ----------------------------------------------------------------------------------------------- :     -
AOTVOKIR3DS4*04    : ----------------------------------------------------------------------------------------------- :     -
AOTVOKIR3DS4*04v1  : ----------------------------------------------------------------------------------------------- :     -
AOTVOKIR3DS5*01    : ----------------------------------------------------------------------------------------------- :     -
AOTVOKIR2DS5*01v1  : ----------------------------------------------------------------------------------------------- :     -
AOTVOKIR2DS5*01v2  : ----------------------------------------------------------------------------------------------- :     -
AOTVOKIR3DS7*01    : ----------------------------------------------------------------------------------------------- :     -
AOTVOKIR3DS7*01v1  : ----------------------------------------------------------------------------------------------- :     -
AOTVOKIR3DL8*01    : ----------------------------------------------------------------------------------------------- :     -
AOTVOKIR3DL8*01v1  : ----------------------------------------------------------------------------------------------- :     -
AOTVOKIR3DS8*01v2  : ----------------------------------------------------------------------------------------------- :     -
AOTVOKIR3DL8*02    : ----------------------------------------------------------------------------------------------- :     -
AOTVOKIR4DL9*01    : ----------------------------------------------------------------------------------------------- :     -
AOTVOKIR4DS9*01v1  : ----------------------------------------------------------------------------------------------- :     -
AOTVOKIR3DL9*01v2  : ----------------------------------------------------------------------------------------------- :     -
AOTVOKIR4DL9*02    : ----------------------------------------------------------------------------------------------- :     -
AOTVOKIR4DL10*01   : ----------------------------------------------------------------------------------------------- :     -
AOTVOKIR3DL10*01v1 : ----------------------------------------------------------------------------------------------- :     -
AOTVOKIR4DS10*01v2 : ----------------------------------------------------------------------------------------------- :     -
AOTVOKIR3DS10*01v3 : ----------------------------------------------------------------------------------------------- :     -
AOTVOKIR3DS10*01v4 : ----------------------------------------------------------------------------------------------- :     -

                     11120         *     11140         *     11160         *     11180         *     11200         *
BAC_clone_Om       : TGGGGCATTTTAGAGAAGTTCCACTTGCCAAGGAATTAGCTCCTGTCCCTCATGATGCAACACTGGCTGACTCAGCAGAGCAAGAGCTTTGCAGT : 11210
AOTVOKIR3DL4*01    : ----------------------------------------------------------------------------------------------- :     -
AOTVOKIR3DL4*02    : ----------------------------------------------------------------------------------------------- :     -
AOTVOKIR3DL4*02v1  : ----------------------------------------------------------------------------------------------- :     -
AOTVOKIR3DL4*02v2  : ----------------------------------------------------------------------------------------------- :     -
AOTVOKIR3DL4*03    : ----------------------------------------------------------------------------------------------- :     -
AOTVOKIR3DS4*04    : ----------------------------------------------------------------------------------------------- :     -
AOTVOKIR3DS4*04v1  : ----------------------------------------------------------------------------------------------- :     -
AOTVOKIR3DS5*01    : ----------------------------------------------------------------------------------------------- :     -
```

```
AOTVOKIR2DS5*01v1  : ----------------------------------------------------------------------------------------------- :     -
AOTVOKIR2DS5*01v2  : ----------------------------------------------------------------------------------------------- :     -
AOTVOKIR3DS7*01    : ----------------------------------------------------------------------------------------------- :     -
AOTVOKIR3DS7*01v1  : ----------------------------------------------------------------------------------------------- :     -
AOTVOKIR3DL8*01    : ----------------------------------------------------------------------------------------------- :     -
AOTVOKIR3DL8*01v1  : ----------------------------------------------------------------------------------------------- :     -
AOTVOKIR3DS8*01v2  : ----------------------------------------------------------------------------------------------- :     -
AOTVOKIR3DL8*02    : ----------------------------------------------------------------------------------------------- :     -
AOTVOKIR4DL9*01    : ----------------------------------------------------------------------------------------------- :     -
AOTVOKIR4DS9*01v1  : ----------------------------------------------------------------------------------------------- :     -
AOTVOKIR3DL9*01v2  : ----------------------------------------------------------------------------------------------- :     -
AOTVOKIR4DL9*02    : ----------------------------------------------------------------------------------------------- :     -
AOTVOKIR4DL10*01   : ----------------------------------------------------------------------------------------------- :     -
AOTVOKIR3DL10*01v1 : ----------------------------------------------------------------------------------------------- :     -
AOTVOKIR4DS10*01v2 : ----------------------------------------------------------------------------------------------- :     -
AOTVOKIR3DS10*01v3 : ----------------------------------------------------------------------------------------------- :     -
AOTVOKIR3DS10*01v4 : ----------------------------------------------------------------------------------------------- :     -

                          11220         *     11240         *     11260         *     11280         *     11300
BAC_clone_Om       : AAGAGAGAACCGAGCTCACGCATGCGCACTTTGACTGGATCACTCATTCGACCCAACCACTCACTGCAGCCTCCGCCTCCTCAGTTGAAGCAGGA : 11305
AOTVOKIR3DL4*01    : ----------------------------------------------------------------------------------------------- :     -
AOTVOKIR3DL4*02    : ----------------------------------------------------------------------------------------------- :     -
AOTVOKIR3DL4*02v1  : ----------------------------------------------------------------------------------------------- :     -
AOTVOKIR3DL4*02v2  : ----------------------------------------------------------------------------------------------- :     -
AOTVOKIR3DL4*03    : ----------------------------------------------------------------------------------------------- :     -
AOTVOKIR3DS4*04    : ----------------------------------------------------------------------------------------------- :     -
AOTVOKIR3DS4*04v1  : ----------------------------------------------------------------------------------------------- :     -
AOTVOKIR3DS5*01    : ----------------------------------------------------------------------------------------------- :     -
AOTVOKIR2DS5*01v1  : ----------------------------------------------------------------------------------------------- :     -
AOTVOKIR2DS5*01v2  : ----------------------------------------------------------------------------------------------- :     -
AOTVOKIR3DS7*01    : ----------------------------------------------------------------------------------------------- :     -
AOTVOKIR3DS7*01v1  : ----------------------------------------------------------------------------------------------- :     -
AOTVOKIR3DL8*01    : ----------------------------------------------------------------------------------------------- :     -
AOTVOKIR3DL8*01v1  : ----------------------------------------------------------------------------------------------- :     -
AOTVOKIR3DS8*01v2  : ----------------------------------------------------------------------------------------------- :     -
AOTVOKIR3DL8*02    : ----------------------------------------------------------------------------------------------- :     -
AOTVOKIR4DL9*01    : ----------------------------------------------------------------------------------------------- :     -
AOTVOKIR4DS9*01v1  : ----------------------------------------------------------------------------------------------- :     -
AOTVOKIR3DL9*01v2  : ----------------------------------------------------------------------------------------------- :     -
AOTVOKIR4DL9*02    : ----------------------------------------------------------------------------------------------- :     -
AOTVOKIR4DL10*01   : ----------------------------------------------------------------------------------------------- :     -
AOTVOKIR3DL10*01v1 : ----------------------------------------------------------------------------------------------- :     -
AOTVOKIR4DS10*01v2 : ----------------------------------------------------------------------------------------------- :     -
AOTVOKIR3DS10*01v3 : ----------------------------------------------------------------------------------------------- :     -
AOTVOKIR3DS10*01v4 : ----------------------------------------------------------------------------------------------- :     -
```

```
                             *      11320         *      11340         *      11360         *      11380         *      11400
BAC_clone_Om         : GAATGACTTCAACCCAGGAGGCGGAGGTTGCAGTGAGCCGAGATCACACCACTGCACTCCAGCCTGGGCGACACAGGGAGGCTCCATTTCCAAAA : 11400
AOTVOKIR3DL4*01      : ----------------------------------------------------------------------------------------------- :     -
AOTVOKIR3DL4*02      : ----------------------------------------------------------------------------------------------- :     -
AOTVOKIR3DL4*02v1    : ----------------------------------------------------------------------------------------------- :     -
AOTVOKIR3DL4*02v2    : ----------------------------------------------------------------------------------------------- :     -
AOTVOKIR3DL4*03      : ----------------------------------------------------------------------------------------------- :     -
AOTVOKIR3DS4*04      : ----------------------------------------------------------------------------------------------- :     -
AOTVOKIR3DS4*04v1    : ----------------------------------------------------------------------------------------------- :     -
AOTVOKIR3DS5*01      : ----------------------------------------------------------------------------------------------- :     -
AOTVOKIR2DS5*01v1    : ----------------------------------------------------------------------------------------------- :     -
AOTVOKIR2DS5*01v2    : ----------------------------------------------------------------------------------------------- :     -
AOTVOKIR3DS7*01      : ----------------------------------------------------------------------------------------------- :     -
AOTVOKIR3DS7*01v1    : ----------------------------------------------------------------------------------------------- :     -
AOTVOKIR3DL8*01      : ----------------------------------------------------------------------------------------------- :     -
AOTVOKIR3DL8*01v1    : ----------------------------------------------------------------------------------------------- :     -
AOTVOKIR3DS8*01v2    : ----------------------------------------------------------------------------------------------- :     -
AOTVOKIR3DL8*02      : ----------------------------------------------------------------------------------------------- :     -
AOTVOKIR4DL9*01      : ----------------------------------------------------------------------------------------------- :     -
AOTVOKIR4DS9*01v1    : ----------------------------------------------------------------------------------------------- :     -
AOTVOKIR3DL9*01v2    : ----------------------------------------------------------------------------------------------- :     -
AOTVOKIR4DL9*02      : ----------------------------------------------------------------------------------------------- :     -
AOTVOKIR4DL10*01     : ----------------------------------------------------------------------------------------------- :     -
AOTVOKIR3DL10*01v1   : ----------------------------------------------------------------------------------------------- :     -
AOTVOKIR4DS10*01v2   : ----------------------------------------------------------------------------------------------- :     -
AOTVOKIR3DS10*01v3   : ----------------------------------------------------------------------------------------------- :     -
AOTVOKIR3DS10*01v4   : ----------------------------------------------------------------------------------------------- :     -

                                   *      11420         *      11440         *      11460         *      11480         *
BAC_clone_Om         : TTAAAATTAAAAACTACGTAAATGTTCTATAACACACACGAATGACAGAGGCACCTGATTCCAATCATCATTTTTCTACTTCTCCATAATTACTT : 11495
AOTVOKIR3DL4*01      : ----------------------------------------------------------------------------------------------- :     -
AOTVOKIR3DL4*02      : ----------------------------------------------------------------------------------------------- :     -
AOTVOKIR3DL4*02v1    : ----------------------------------------------------------------------------------------------- :     -
AOTVOKIR3DL4*02v2    : ----------------------------------------------------------------------------------------------- :     -
AOTVOKIR3DL4*03      : ----------------------------------------------------------------------------------------------- :     -
AOTVOKIR3DS4*04      : ----------------------------------------------------------------------------------------------- :     -
AOTVOKIR3DS4*04v1    : ----------------------------------------------------------------------------------------------- :     -
AOTVOKIR3DS5*01      : ----------------------------------------------------------------------------------------------- :     -
AOTVOKIR2DS5*01v1    : ----------------------------------------------------------------------------------------------- :     -
AOTVOKIR2DS5*01v2    : ----------------------------------------------------------------------------------------------- :     -
AOTVOKIR3DS7*01      : ----------------------------------------------------------------------------------------------- :     -
AOTVOKIR3DS7*01v1    : ----------------------------------------------------------------------------------------------- :     -
AOTVOKIR3DL8*01      : ----------------------------------------------------------------------------------------------- :     -
```

```
AOTVOKIR3DL8*01v1  : ----------------------------------------------------------------------------------------------- :     -
AOTVOKIR3DS8*01v2  : ----------------------------------------------------------------------------------------------- :     -
AOTVOKIR3DL8*02    : ----------------------------------------------------------------------------------------------- :     -
AOTVOKIR4DL9*01    : ----------------------------------------------------------------------------------------------- :     -
AOTVOKIR4DS9*01v1  : ----------------------------------------------------------------------------------------------- :     -
AOTVOKIR3DL9*01v2  : ----------------------------------------------------------------------------------------------- :     -
AOTVOKIR4DL9*02    : ----------------------------------------------------------------------------------------------- :     -
AOTVOKIR4DL10*01   : ----------------------------------------------------------------------------------------------- :     -
AOTVOKIR3DL10*01v1 : ----------------------------------------------------------------------------------------------- :     -
AOTVOKIR4DS10*01v2 : ----------------------------------------------------------------------------------------------- :     -
AOTVOKIR3DS10*01v3 : ----------------------------------------------------------------------------------------------- :     -
AOTVOKIR3DS10*01v4 : ----------------------------------------------------------------------------------------------- :     -

                     11500         *     11520         *     11540         *     11560         *     11580         *
BAC_clone_Om       : TTTTGATCCTTACTCTTATCGATTAGAAAATCAGCCTAGAACCTCTTCCCTATTTGGCTTTCTGTCACCATGAGAGCATATGGAAAATGGGAAAG : 11590
AOTVOKIR3DL4*01    : ----------------------------------------------------------------------------------------------- :     -
AOTVOKIR3DL4*02    : ----------------------------------------------------------------------------------------------- :     -
AOTVOKIR3DL4*02v1  : ----------------------------------------------------------------------------------------------- :     -
AOTVOKIR3DL4*02v2  : ----------------------------------------------------------------------------------------------- :     -
AOTVOKIR3DL4*03    : ----------------------------------------------------------------------------------------------- :     -
AOTVOKIR3DS4*04    : ----------------------------------------------------------------------------------------------- :     -
AOTVOKIR3DS4*04v1  : ----------------------------------------------------------------------------------------------- :     -
AOTVOKIR3DS5*01    : ----------------------------------------------------------------------------------------------- :     -
AOTVOKIR2DS5*01v1  : ----------------------------------------------------------------------------------------------- :     -
AOTVOKIR2DS5*01v2  : ----------------------------------------------------------------------------------------------- :     -
AOTVOKIR3DS7*01    : ----------------------------------------------------------------------------------------------- :     -
AOTVOKIR3DS7*01v1  : ----------------------------------------------------------------------------------------------- :     -
AOTVOKIR3DL8*01    : ----------------------------------------------------------------------------------------------- :     -
AOTVOKIR3DL8*01v1  : ----------------------------------------------------------------------------------------------- :     -
AOTVOKIR3DS8*01v2  : ----------------------------------------------------------------------------------------------- :     -
AOTVOKIR3DL8*02    : ----------------------------------------------------------------------------------------------- :     -
AOTVOKIR4DL9*01    : ----------------------------------------------------------------------------------------------- :     -
AOTVOKIR4DS9*01v1  : ----------------------------------------------------------------------------------------------- :     -
AOTVOKIR3DL9*01v2  : ----------------------------------------------------------------------------------------------- :     -
AOTVOKIR4DL9*02    : ----------------------------------------------------------------------------------------------- :     -
AOTVOKIR4DL10*01   : ----------------------------------------------------------------------------------------------- :     -
AOTVOKIR3DL10*01v1 : ----------------------------------------------------------------------------------------------- :     -
AOTVOKIR4DS10*01v2 : ----------------------------------------------------------------------------------------------- :     -
AOTVOKIR3DS10*01v3 : ----------------------------------------------------------------------------------------------- :     -
AOTVOKIR3DS10*01v4 : ----------------------------------------------------------------------------------------------- :     -

                          11600         *     11620         *     11640         *     11660         *     11680
BAC_clone_Om       : CCCTCAACCCAGCAGCACAGGTCCTGAAATAGAGGAAGTGCTCTCTTCATGGCATAAAAGTTGCCCCCTCACCCAGATCCCCCACCTCACCCCTA : 11685
AOTVOKIR3DL4*01    : ----------------------------------------------------------------------------------------------- :     -
```

```
AOTVOKIR3DL4*02      : ----------------------------------------------------------------------------------------------- :     -
AOTVOKIR3DL4*02v1    : ----------------------------------------------------------------------------------------------- :     -
AOTVOKIR3DL4*02v2    : ----------------------------------------------------------------------------------------------- :     -
AOTVOKIR3DL4*03      : ----------------------------------------------------------------------------------------------- :     -
AOTVOKIR3DS4*04      : ----------------------------------------------------------------------------------------------- :     -
AOTVOKIR3DS4*04v1    : ----------------------------------------------------------------------------------------------- :     -
AOTVOKIR3DS5*01      : ----------------------------------------------------------------------------------------------- :     -
AOTVOKIR2DS5*01v1    : ----------------------------------------------------------------------------------------------- :     -
AOTVOKIR2DS5*01v2    : ----------------------------------------------------------------------------------------------- :     -
AOTVOKIR3DS7*01      : ----------------------------------------------------------------------------------------------- :     -
AOTVOKIR3DS7*01v1    : ----------------------------------------------------------------------------------------------- :     -
AOTVOKIR3DL8*01      : ----------------------------------------------------------------------------------------------- :     -
AOTVOKIR3DL8*01v1    : ----------------------------------------------------------------------------------------------- :     -
AOTVOKIR3DS8*01v2    : ----------------------------------------------------------------------------------------------- :     -
AOTVOKIR3DL8*02      : ----------------------------------------------------------------------------------------------- :     -
AOTVOKIR4DL9*01      : ----------------------------------------------------------------------------------------------- :     -
AOTVOKIR4DS9*01v1    : ----------------------------------------------------------------------------------------------- :     -
AOTVOKIR3DL9*01v2    : ----------------------------------------------------------------------------------------------- :     -
AOTVOKIR4DL9*02      : ----------------------------------------------------------------------------------------------- :     -
AOTVOKIR4DL10*01     : ----------------------------------------------------------------------------------------------- :     -
AOTVOKIR3DL10*01v1   : ----------------------------------------------------------------------------------------------- :     -
AOTVOKIR4DS10*01v2   : ----------------------------------------------------------------------------------------------- :     -
AOTVOKIR3DS10*01v3   : ----------------------------------------------------------------------------------------------- :     -
AOTVOKIR3DS10*01v4   : ----------------------------------------------------------------------------------------------- :     -


                           *      11700           *      11720           *      11740           *      11760           *      11780
BAC_clone_Om         : CTTCCAATCACGTGTGGAGATACAGATAGATTATGGGGAGGTCAAAACTAATATTCTTTGGAGTGACCTCAGTTCTTGGACTCAGAGACCAATGC : 11780
AOTVOKIR3DL4*01      : ----------------------------------------------------------------------------------------------- :     -
AOTVOKIR3DL4*02      : ----------------------------------------------------------------------------------------------- :     -
AOTVOKIR3DL4*02v1    : ----------------------------------------------------------------------------------------------- :     -
AOTVOKIR3DL4*02v2    : ----------------------------------------------------------------------------------------------- :     -
AOTVOKIR3DL4*03      : ----------------------------------------------------------------------------------------------- :     -
AOTVOKIR3DS4*04      : ----------------------------------------------------------------------------------------------- :     -
AOTVOKIR3DS4*04v1    : ----------------------------------------------------------------------------------------------- :     -
AOTVOKIR3DS5*01      : ----------------------------------------------------------------------------------------------- :     -
AOTVOKIR2DS5*01v1    : ----------------------------------------------------------------------------------------------- :     -
AOTVOKIR2DS5*01v2    : ----------------------------------------------------------------------------------------------- :     -
AOTVOKIR3DS7*01      : ----------------------------------------------------------------------------------------------- :     -
AOTVOKIR3DS7*01v1    : ----------------------------------------------------------------------------------------------- :     -
AOTVOKIR3DL8*01      : ----------------------------------------------------------------------------------------------- :     -
AOTVOKIR3DL8*01v1    : ----------------------------------------------------------------------------------------------- :     -
AOTVOKIR3DS8*01v2    : ----------------------------------------------------------------------------------------------- :     -
AOTVOKIR3DL8*02      : ----------------------------------------------------------------------------------------------- :     -
AOTVOKIR4DL9*01      : ----------------------------------------------------------------------------------------------- :     -
AOTVOKIR4DS9*01v1    : ----------------------------------------------------------------------------------------------- :     -
```

```
AOTVOKIR3DL9*01v2  : ----------------------------------------------------------------------------------------------- :     -
AOTVOKIR4DL9*02    : ----------------------------------------------------------------------------------------------- :     -
AOTVOKIR4DL10*01   : ----------------------------------------------------------------------------------------------- :     -
AOTVOKIR3DL10*01v1 : ----------------------------------------------------------------------------------------------- :     -
AOTVOKIR4DS10*01v2 : ----------------------------------------------------------------------------------------------- :     -
AOTVOKIR3DS10*01v3 : ----------------------------------------------------------------------------------------------- :     -
AOTVOKIR3DS10*01v4 : ----------------------------------------------------------------------------------------------- :     -

                              *     11800         *     11820         *     11840         *     11860         *
BAC_clone_Om       : CAGCACTATCTCCTGGTCCCCTTTCCTACTAATTCACAGAAGGACAGGCTGTATTTTGAAGCAATAGATGATGGAGGGCGGGTAGAACAGCAGCC : 11875
AOTVOKIR3DL4*01    : ----------------------------------------------------------------------------------------------- :     -
AOTVOKIR3DL4*02    : ----------------------------------------------------------------------------------------------- :     -
AOTVOKIR3DL4*02v1  : ----------------------------------------------------------------------------------------------- :     -
AOTVOKIR3DL4*02v2  : ----------------------------------------------------------------------------------------------- :     -
AOTVOKIR3DL4*03    : ----------------------------------------------------------------------------------------------- :     -
AOTVOKIR3DS4*04    : ----------------------------------------------------------------------------------------------- :     -
AOTVOKIR3DS4*04v1  : ----------------------------------------------------------------------------------------------- :     -
AOTVOKIR3DS5*01    : ----------------------------------------------------------------------------------------------- :     -
AOTVOKIR2DS5*01v1  : ----------------------------------------------------------------------------------------------- :     -
AOTVOKIR2DS5*01v2  : ----------------------------------------------------------------------------------------------- :     -
AOTVOKIR3DS7*01    : ----------------------------------------------------------------------------------------------- :     -
AOTVOKIR3DS7*01v1  : ----------------------------------------------------------------------------------------------- :     -
AOTVOKIR3DL8*01    : ----------------------------------------------------------------------------------------------- :     -
AOTVOKIR3DL8*01v1  : ----------------------------------------------------------------------------------------------- :     -
AOTVOKIR3DS8*01v2  : ----------------------------------------------------------------------------------------------- :     -
AOTVOKIR3DL8*02    : ----------------------------------------------------------------------------------------------- :     -
AOTVOKIR4DL9*01    : ----------------------------------------------------------------------------------------------- :     -
AOTVOKIR4DS9*01v1  : ----------------------------------------------------------------------------------------------- :     -
AOTVOKIR3DL9*01v2  : ----------------------------------------------------------------------------------------------- :     -
AOTVOKIR4DL9*02    : ----------------------------------------------------------------------------------------------- :     -
AOTVOKIR4DL10*01   : ----------------------------------------------------------------------------------------------- :     -
AOTVOKIR3DL10*01v1 : ----------------------------------------------------------------------------------------------- :     -
AOTVOKIR4DS10*01v2 : ----------------------------------------------------------------------------------------------- :     -
AOTVOKIR3DS10*01v3 : ----------------------------------------------------------------------------------------------- :     -
AOTVOKIR3DS10*01v4 : ----------------------------------------------------------------------------------------------- :     -

                     11880         *     11900         *     11920         *     11940         *     11960         *
BAC_clone_Om       : TATTACACGTCTCACGAGATCACAAAAAGTAACACGTTGCCCACGGGCTTCTGCACTATTTCCTGGCTGTTTGATGTAAGACATTCTACTTCACT : 11970
AOTVOKIR3DL4*01    : ----------------------------------------------------------------------------------------------- :     -
AOTVOKIR3DL4*02    : ----------------------------------------------------------------------------------------------- :     -
AOTVOKIR3DL4*02v1  : ----------------------------------------------------------------------------------------------- :     -
AOTVOKIR3DL4*02v2  : ----------------------------------------------------------------------------------------------- :     -
AOTVOKIR3DL4*03    : ----------------------------------------------------------------------------------------------- :     -
AOTVOKIR3DS4*04    : ----------------------------------------------------------------------------------------------- :     -
```

```
AOTVOKIR3DS4*04v1  : ----------------------------------------------------------------------------------------------- :     -
AOTVOKIR3DS5*01    : ----------------------------------------------------------------------------------------------- :     -
AOTVOKIR2DS5*01v1  : ----------------------------------------------------------------------------------------------- :     -
AOTVOKIR2DS5*01v2  : ----------------------------------------------------------------------------------------------- :     -
AOTVOKIR3DS7*01    : ----------------------------------------------------------------------------------------------- :     -
AOTVOKIR3DS7*01v1  : ----------------------------------------------------------------------------------------------- :     -
AOTVOKIR3DL8*01    : ----------------------------------------------------------------------------------------------- :     -
AOTVOKIR3DL8*01v1  : ----------------------------------------------------------------------------------------------- :     -
AOTVOKIR3DS8*01v2  : ----------------------------------------------------------------------------------------------- :     -
AOTVOKIR3DL8*02    : ----------------------------------------------------------------------------------------------- :     -
AOTVOKIR4DL9*01    : ----------------------------------------------------------------------------------------------- :     -
AOTVOKIR4DS9*01v1  : ----------------------------------------------------------------------------------------------- :     -
AOTVOKIR3DL9*01v2  : ----------------------------------------------------------------------------------------------- :     -
AOTVOKIR4DL9*02    : ----------------------------------------------------------------------------------------------- :     -
AOTVOKIR4DL10*01   : ----------------------------------------------------------------------------------------------- :     -
AOTVOKIR3DL10*01v1 : ----------------------------------------------------------------------------------------------- :     -
AOTVOKIR4DS10*01v2 : ----------------------------------------------------------------------------------------------- :     -
AOTVOKIR3DS10*01v3 : ----------------------------------------------------------------------------------------------- :     -
AOTVOKIR3DS10*01v4 : ----------------------------------------------------------------------------------------------- :     -

                          11980         *     12000         *     12020         *     12040         *     12060
BAC_clone_Om       : TCTTTCGATTTCGATTCCACTTTTTTTTCCTTTTCTTGGAGAACGTAATTTGTTTGAGTCAAGAGTGTTGTGGATGCAGACGTTGTAAAGCACAT : 12065
AOTVOKIR3DL4*01    : ----------------------------------------------------------------------------------------------- :     -
AOTVOKIR3DL4*02    : ----------------------------------------------------------------------------------------------- :     -
AOTVOKIR3DL4*02v1  : ----------------------------------------------------------------------------------------------- :     -
AOTVOKIR3DL4*02v2  : ----------------------------------------------------------------------------------------------- :     -
AOTVOKIR3DL4*03    : ----------------------------------------------------------------------------------------------- :     -
AOTVOKIR3DS4*04    : ----------------------------------------------------------------------------------------------- :     -
AOTVOKIR3DS4*04v1  : ----------------------------------------------------------------------------------------------- :     -
AOTVOKIR3DS5*01    : ----------------------------------------------------------------------------------------------- :     -
AOTVOKIR2DS5*01v1  : ----------------------------------------------------------------------------------------------- :     -
AOTVOKIR2DS5*01v2  : ----------------------------------------------------------------------------------------------- :     -
AOTVOKIR3DS7*01    : ----------------------------------------------------------------------------------------------- :     -
AOTVOKIR3DS7*01v1  : ----------------------------------------------------------------------------------------------- :     -
AOTVOKIR3DL8*01    : ----------------------------------------------------------------------------------------------- :     -
AOTVOKIR3DL8*01v1  : ----------------------------------------------------------------------------------------------- :     -
AOTVOKIR3DS8*01v2  : ----------------------------------------------------------------------------------------------- :     -
AOTVOKIR3DL8*02    : ----------------------------------------------------------------------------------------------- :     -
AOTVOKIR4DL9*01    : ----------------------------------------------------------------------------------------------- :     -
AOTVOKIR4DS9*01v1  : ----------------------------------------------------------------------------------------------- :     -
AOTVOKIR3DL9*01v2  : ----------------------------------------------------------------------------------------------- :     -
AOTVOKIR4DL9*02    : ----------------------------------------------------------------------------------------------- :     -
AOTVOKIR4DL10*01   : ----------------------------------------------------------------------------------------------- :     -
AOTVOKIR3DL10*01v1 : ----------------------------------------------------------------------------------------------- :     -
AOTVOKIR4DS10*01v2 : ----------------------------------------------------------------------------------------------- :     -
```

```
AOTVOKIR3DS10*01v3 : ----------------------------------------------------------------------------------------------- :     -
AOTVOKIR3DS10*01v4 : ----------------------------------------------------------------------------------------------- :     -

                         *     12080         *     12100         *     12120         *     12140         *     12160
BAC_clone_Om       : TCGCTGTGTATCAATCCCAGTCCGGTCTTCCTAGAGAAGACTCTGAACTCCTCCTGACTGCACCTGGGGCTATGCCAGTTTCTATCACTGACAGT : 12160
AOTVOKIR3DL4*01    : ----------------------------------------------------------------------------------------------- :     -
AOTVOKIR3DL4*02    : ----------------------------------------------------------------------------------------------- :     -
AOTVOKIR3DL4*02v1  : ----------------------------------------------------------------------------------------------- :     -
AOTVOKIR3DL4*02v2  : ----------------------------------------------------------------------------------------------- :     -
AOTVOKIR3DL4*03    : ----------------------------------------------------------------------------------------------- :     -
AOTVOKIR3DS4*04    : ----------------------------------------------------------------------------------------------- :     -
AOTVOKIR3DS4*04v1  : ----------------------------------------------------------------------------------------------- :     -
AOTVOKIR3DS5*01    : ----------------------------------------------------------------------------------------------- :     -
AOTVOKIR2DS5*01v1  : ----------------------------------------------------------------------------------------------- :     -
AOTVOKIR2DS5*01v2  : ----------------------------------------------------------------------------------------------- :     -
AOTVOKIR3DS7*01    : ----------------------------------------------------------------------------------------------- :     -
AOTVOKIR3DS7*01v1  : ----------------------------------------------------------------------------------------------- :     -
AOTVOKIR3DL8*01    : ----------------------------------------------------------------------------------------------- :     -
AOTVOKIR3DL8*01v1  : ----------------------------------------------------------------------------------------------- :     -
AOTVOKIR3DS8*01v2  : ----------------------------------------------------------------------------------------------- :     -
AOTVOKIR3DL8*02    : ----------------------------------------------------------------------------------------------- :     -
AOTVOKIR4DL9*01    : ----------------------------------------------------------------------------------------------- :     -
AOTVOKIR4DS9*01v1  : ----------------------------------------------------------------------------------------------- :     -
AOTVOKIR3DL9*01v2  : ----------------------------------------------------------------------------------------------- :     -
AOTVOKIR4DL9*02    : ----------------------------------------------------------------------------------------------- :     -
AOTVOKIR4DL10*01   : ----------------------------------------------------------------------------------------------- :     -
AOTVOKIR3DL10*01v1 : ----------------------------------------------------------------------------------------------- :     -
AOTVOKIR4DS10*01v2 : ----------------------------------------------------------------------------------------------- :     -
AOTVOKIR3DS10*01v3 : ----------------------------------------------------------------------------------------------- :     -
AOTVOKIR3DS10*01v4 : ----------------------------------------------------------------------------------------------- :     -

                              *     12180         *     12200         *     12220         *     12240         *
BAC_clone_Om       : CACTCCAGGGAGACAGAACACAGACAGAATAAATTACATAAAGCAGGTTCATGGGGCCGGGTGCGGTGGCTCACGCCTGTAATCCCAGCACTTTG : 12255
AOTVOKIR3DL4*01    : ----------------------------------------------------------------------------------------------- :     -
AOTVOKIR3DL4*02    : ----------------------------------------------------------------------------------------------- :     -
AOTVOKIR3DL4*02v1  : ----------------------------------------------------------------------------------------------- :     -
AOTVOKIR3DL4*02v2  : ----------------------------------------------------------------------------------------------- :     -
AOTVOKIR3DL4*03    : ----------------------------------------------------------------------------------------------- :     -
AOTVOKIR3DS4*04    : ----------------------------------------------------------------------------------------------- :     -
AOTVOKIR3DS4*04v1  : ----------------------------------------------------------------------------------------------- :     -
AOTVOKIR3DS5*01    : ----------------------------------------------------------------------------------------------- :     -
AOTVOKIR2DS5*01v1  : ----------------------------------------------------------------------------------------------- :     -
AOTVOKIR2DS5*01v2  : ----------------------------------------------------------------------------------------------- :     -
AOTVOKIR3DS7*01    : ----------------------------------------------------------------------------------------------- :     -
```

```
AOTVOKIR3DS7*01v1  : ----------------------------------------------------------------------------------------------- :     -
AOTVOKIR3DL8*01    : ----------------------------------------------------------------------------------------------- :     -
AOTVOKIR3DL8*01v1  : ----------------------------------------------------------------------------------------------- :     -
AOTVOKIR3DS8*01v2  : ----------------------------------------------------------------------------------------------- :     -
AOTVOKIR3DL8*02    : ----------------------------------------------------------------------------------------------- :     -
AOTVOKIR4DL9*01    : ----------------------------------------------------------------------------------------------- :     -
AOTVOKIR4DS9*01v1  : ----------------------------------------------------------------------------------------------- :     -
AOTVOKIR3DL9*01v2  : ----------------------------------------------------------------------------------------------- :     -
AOTVOKIR4DL9*02    : ----------------------------------------------------------------------------------------------- :     -
AOTVOKIR4DL10*01   : ----------------------------------------------------------------------------------------------- :     -
AOTVOKIR3DL10*01v1 : ----------------------------------------------------------------------------------------------- :     -
AOTVOKIR4DS10*01v2 : ----------------------------------------------------------------------------------------------- :     -
AOTVOKIR3DS10*01v3 : ----------------------------------------------------------------------------------------------- :     -
AOTVOKIR3DS10*01v4 : ----------------------------------------------------------------------------------------------- :     -


                     12260         *     12280         *     12300         *     12320         *     12340         *
BAC_clone_Om       : GGAAGCCGAGGCGGGTGGATCATGAGGTCAAGAGATCGAGACCATCCTGGTCAACATGGTGAAACCCCATCTCTACTAAAAATACAAAATATTAG : 12350
AOTVOKIR3DL4*01    : ----------------------------------------------------------------------------------------------- :     -
AOTVOKIR3DL4*02    : ----------------------------------------------------------------------------------------------- :     -
AOTVOKIR3DL4*02v1  : ----------------------------------------------------------------------------------------------- :     -
AOTVOKIR3DL4*02v2  : ----------------------------------------------------------------------------------------------- :     -
AOTVOKIR3DL4*03    : ----------------------------------------------------------------------------------------------- :     -
AOTVOKIR3DS4*04    : ----------------------------------------------------------------------------------------------- :     -
AOTVOKIR3DS4*04v1  : ----------------------------------------------------------------------------------------------- :     -
AOTVOKIR3DS5*01    : ----------------------------------------------------------------------------------------------- :     -
AOTVOKIR2DS5*01v1  : ----------------------------------------------------------------------------------------------- :     -
AOTVOKIR2DS5*01v2  : ----------------------------------------------------------------------------------------------- :     -
AOTVOKIR3DS7*01    : ----------------------------------------------------------------------------------------------- :     -
AOTVOKIR3DS7*01v1  : ----------------------------------------------------------------------------------------------- :     -
AOTVOKIR3DL8*01    : ----------------------------------------------------------------------------------------------- :     -
AOTVOKIR3DL8*01v1  : ----------------------------------------------------------------------------------------------- :     -
AOTVOKIR3DS8*01v2  : ----------------------------------------------------------------------------------------------- :     -
AOTVOKIR3DL8*02    : ----------------------------------------------------------------------------------------------- :     -
AOTVOKIR4DL9*01    : ----------------------------------------------------------------------------------------------- :     -
AOTVOKIR4DS9*01v1  : ----------------------------------------------------------------------------------------------- :     -
AOTVOKIR3DL9*01v2  : ----------------------------------------------------------------------------------------------- :     -
AOTVOKIR4DL9*02    : ----------------------------------------------------------------------------------------------- :     -
AOTVOKIR4DL10*01   : ----------------------------------------------------------------------------------------------- :     -
AOTVOKIR3DL10*01v1 : ----------------------------------------------------------------------------------------------- :     -
AOTVOKIR4DS10*01v2 : ----------------------------------------------------------------------------------------------- :     -
AOTVOKIR3DS10*01v3 : ----------------------------------------------------------------------------------------------- :     -
AOTVOKIR3DS10*01v4 : ----------------------------------------------------------------------------------------------- :     -


                          12360         *     12380         *     12400         *     12420         *     12440
```

```
BAC_clone_Om        : CTGGGCACGGTGGCACGTGCCTGTAATCCCAGCTACTCTGGAGGCTGAGGCAGGAGAATTGCCTGAACCCAGGAGGCGGAGGTTGCGGTGAGCCG : 12445
AOTVOKIR3DL4*01     : ----------------------------------------------------------------------------------------------- :     -
AOTVOKIR3DL4*02     : ----------------------------------------------------------------------------------------------- :     -
AOTVOKIR3DL4*02v1   : ----------------------------------------------------------------------------------------------- :     -
AOTVOKIR3DL4*02v2   : ----------------------------------------------------------------------------------------------- :     -
AOTVOKIR3DL4*03     : ----------------------------------------------------------------------------------------------- :     -
AOTVOKIR3DS4*04     : ----------------------------------------------------------------------------------------------- :     -
AOTVOKIR3DS4*04v1   : ----------------------------------------------------------------------------------------------- :     -
AOTVOKIR3DS5*01     : ----------------------------------------------------------------------------------------------- :     -
AOTVOKIR2DS5*01v1   : ----------------------------------------------------------------------------------------------- :     -
AOTVOKIR2DS5*01v2   : ----------------------------------------------------------------------------------------------- :     -
AOTVOKIR3DS7*01     : ----------------------------------------------------------------------------------------------- :     -
AOTVOKIR3DS7*01v1   : ----------------------------------------------------------------------------------------------- :     -
AOTVOKIR3DL8*01     : ----------------------------------------------------------------------------------------------- :     -
AOTVOKIR3DL8*01v1   : ----------------------------------------------------------------------------------------------- :     -
AOTVOKIR3DS8*01v2   : ----------------------------------------------------------------------------------------------- :     -
AOTVOKIR3DL8*02     : ----------------------------------------------------------------------------------------------- :     -
AOTVOKIR4DL9*01     : ----------------------------------------------------------------------------------------------- :     -
AOTVOKIR4DS9*01v1   : ----------------------------------------------------------------------------------------------- :     -
AOTVOKIR3DL9*01v2   : ----------------------------------------------------------------------------------------------- :     -
AOTVOKIR4DL9*02     : ----------------------------------------------------------------------------------------------- :     -
AOTVOKIR4DL10*01    : ----------------------------------------------------------------------------------------------- :     -
AOTVOKIR3DL10*01v1  : ----------------------------------------------------------------------------------------------- :     -
AOTVOKIR4DS10*01v2  : ----------------------------------------------------------------------------------------------- :     -
AOTVOKIR3DS10*01v3  : ----------------------------------------------------------------------------------------------- :     -
AOTVOKIR3DS10*01v4  : ----------------------------------------------------------------------------------------------- :     -

                          *     12460         *     12480         *     12500         *     12520         *     12540
BAC_clone_Om        : AGATCGTGCCATTGCTCTCCAGCCTGGGTAACAACAGCGAAACTCCGTCTCAAAAAAAAAAAAAAGAAAGAAAGAAAAAGCAGGTTCATTATTCA : 12540
AOTVOKIR3DL4*01     : ----------------------------------------------------------------------------------------------- :     -
AOTVOKIR3DL4*02     : ----------------------------------------------------------------------------------------------- :     -
AOTVOKIR3DL4*02v1   : ----------------------------------------------------------------------------------------------- :     -
AOTVOKIR3DL4*02v2   : ----------------------------------------------------------------------------------------------- :     -
AOTVOKIR3DL4*03     : ----------------------------------------------------------------------------------------------- :     -
AOTVOKIR3DS4*04     : ----------------------------------------------------------------------------------------------- :     -
AOTVOKIR3DS4*04v1   : ----------------------------------------------------------------------------------------------- :     -
AOTVOKIR3DS5*01     : ----------------------------------------------------------------------------------------------- :     -
AOTVOKIR2DS5*01v1   : ----------------------------------------------------------------------------------------------- :     -
AOTVOKIR2DS5*01v2   : ----------------------------------------------------------------------------------------------- :     -
AOTVOKIR3DS7*01     : ----------------------------------------------------------------------------------------------- :     -
AOTVOKIR3DS7*01v1   : ----------------------------------------------------------------------------------------------- :     -
AOTVOKIR3DL8*01     : ----------------------------------------------------------------------------------------------- :     -
AOTVOKIR3DL8*01v1   : ----------------------------------------------------------------------------------------------- :     -
AOTVOKIR3DS8*01v2   : ----------------------------------------------------------------------------------------------- :     -
AOTVOKIR3DL8*02     : ----------------------------------------------------------------------------------------------- :     -
```

```
AOTVOKIR4DL9*01    : ----------------------------------------------------------------------------------------------- :     -
AOTVOKIR4DS9*01v1  : ----------------------------------------------------------------------------------------------- :     -
AOTVOKIR3DL9*01v2  : ----------------------------------------------------------------------------------------------- :     -
AOTVOKIR4DL9*02    : ----------------------------------------------------------------------------------------------- :     -
AOTVOKIR4DL10*01   : ----------------------------------------------------------------------------------------------- :     -
AOTVOKIR3DL10*01v1 : ----------------------------------------------------------------------------------------------- :     -
AOTVOKIR4DS10*01v2 : ----------------------------------------------------------------------------------------------- :     -
AOTVOKIR3DS10*01v3 : ----------------------------------------------------------------------------------------------- :     -
AOTVOKIR3DS10*01v4 : ----------------------------------------------------------------------------------------------- :     -

                              *     12560         *     12580         *     12600         *     12620         *
BAC_clone_Om       : CAGATCAGTAGCAAGTGATGGCAGAAGCCTACCTTTCAGGGTGAGCCAGTCCCCCGAGGCTCAGAAAAGCGGCCCACGAAACACAGAGCCACCCC : 12635
AOTVOKIR3DL4*01    : ----------------------------------------------------------------------------------------------- :     -
AOTVOKIR3DL4*02    : ----------------------------------------------------------------------------------------------- :     -
AOTVOKIR3DL4*02v1  : ----------------------------------------------------------------------------------------------- :     -
AOTVOKIR3DL4*02v2  : ----------------------------------------------------------------------------------------------- :     -
AOTVOKIR3DL4*03    : ----------------------------------------------------------------------------------------------- :     -
AOTVOKIR3DS4*04    : ----------------------------------------------------------------------------------------------- :     -
AOTVOKIR3DS4*04v1  : ----------------------------------------------------------------------------------------------- :     -
AOTVOKIR3DS5*01    : ----------------------------------------------------------------------------------------------- :     -
AOTVOKIR2DS5*01v1  : ----------------------------------------------------------------------------------------------- :     -
AOTVOKIR2DS5*01v2  : ----------------------------------------------------------------------------------------------- :     -
AOTVOKIR3DS7*01    : ----------------------------------------------------------------------------------------------- :     -
AOTVOKIR3DS7*01v1  : ----------------------------------------------------------------------------------------------- :     -
AOTVOKIR3DL8*01    : ----------------------------------------------------------------------------------------------- :     -
AOTVOKIR3DL8*01v1  : ----------------------------------------------------------------------------------------------- :     -
AOTVOKIR3DS8*01v2  : ----------------------------------------------------------------------------------------------- :     -
AOTVOKIR3DL8*02    : ----------------------------------------------------------------------------------------------- :     -
AOTVOKIR4DL9*01    : ----------------------------------------------------------------------------------------------- :     -
AOTVOKIR4DS9*01v1  : ----------------------------------------------------------------------------------------------- :     -
AOTVOKIR3DL9*01v2  : ----------------------------------------------------------------------------------------------- :     -
AOTVOKIR4DL9*02    : ----------------------------------------------------------------------------------------------- :     -
AOTVOKIR4DL10*01   : ----------------------------------------------------------------------------------------------- :     -
AOTVOKIR3DL10*01v1 : ----------------------------------------------------------------------------------------------- :     -
AOTVOKIR4DS10*01v2 : ----------------------------------------------------------------------------------------------- :     -
AOTVOKIR3DS10*01v3 : ----------------------------------------------------------------------------------------------- :     -
AOTVOKIR3DS10*01v4 : ----------------------------------------------------------------------------------------------- :     -

                     12640         *     12660         *     12680         *     12700         *     12720         *
BAC_clone_Om       : ATATGCAGTGTAGCTGGGGGAACTGAAAAGCAGCCCTGCCTGAGCTTTGCACCCTGGAGCCACAGGAAGCGCTGAGTTAAAGCCCTGCCTCACAC : 12730
AOTVOKIR3DL4*01    : ----------------------------------------------------------------------------------------------- :     -
AOTVOKIR3DL4*02    : ----------------------------------------------------------------------------------------------- :     -
AOTVOKIR3DL4*02v1  : ----------------------------------------------------------------------------------------------- :     -
AOTVOKIR3DL4*02v2  : ----------------------------------------------------------------------------------------------- :     -
```

```
AOTVOKIR3DL4*03    : ----------------------------------------------------------------------------------------------- :     -
AOTVOKIR3DS4*04    : ----------------------------------------------------------------------------------------------- :     -
AOTVOKIR3DS4*04v1  : ----------------------------------------------------------------------------------------------- :     -
AOTVOKIR3DS5*01    : ----------------------------------------------------------------------------------------------- :     -
AOTVOKIR2DS5*01v1  : ----------------------------------------------------------------------------------------------- :     -
AOTVOKIR2DS5*01v2  : ----------------------------------------------------------------------------------------------- :     -
AOTVOKIR3DS7*01    : ----------------------------------------------------------------------------------------------- :     -
AOTVOKIR3DS7*01v1  : ----------------------------------------------------------------------------------------------- :     -
AOTVOKIR3DL8*01    : ----------------------------------------------------------------------------------------------- :     -
AOTVOKIR3DL8*01v1  : ----------------------------------------------------------------------------------------------- :     -
AOTVOKIR3DS8*01v2  : ----------------------------------------------------------------------------------------------- :     -
AOTVOKIR3DL8*02    : ----------------------------------------------------------------------------------------------- :     -
AOTVOKIR4DL9*01    : ----------------------------------------------------------------------------------------------- :     -
AOTVOKIR4DS9*01v1  : ----------------------------------------------------------------------------------------------- :     -
AOTVOKIR3DL9*01v2  : ----------------------------------------------------------------------------------------------- :     -
AOTVOKIR4DL9*02    : ----------------------------------------------------------------------------------------------- :     -
AOTVOKIR4DL10*01   : ----------------------------------------------------------------------------------------------- :     -
AOTVOKIR3DL10*01v1 : ----------------------------------------------------------------------------------------------- :     -
AOTVOKIR4DS10*01v2 : ----------------------------------------------------------------------------------------------- :     -
AOTVOKIR3DS10*01v3 : ----------------------------------------------------------------------------------------------- :     -
AOTVOKIR3DS10*01v4 : ----------------------------------------------------------------------------------------------- :     -


                          12740         *       12760         *       12780         *       12800         *       12820
BAC_clone_Om       : CCTCCTCTAGGAAGAGCAGGAGAGAGCCCAGGCTGTTCTGGGACGTTCCTCCTGATCTCAGGACGTTGCTGTCTTAGTCCGATTTTGTTGCTATC : 12825
AOTVOKIR3DL4*01    : ----------------------------------------------------------------------------------------------- :     -
AOTVOKIR3DL4*02    : ----------------------------------------------------------------------------------------------- :     -
AOTVOKIR3DL4*02v1  : ----------------------------------------------------------------------------------------------- :     -
AOTVOKIR3DL4*02v2  : ----------------------------------------------------------------------------------------------- :     -
AOTVOKIR3DL4*03    : ----------------------------------------------------------------------------------------------- :     -
AOTVOKIR3DS4*04    : ----------------------------------------------------------------------------------------------- :     -
AOTVOKIR3DS4*04v1  : ----------------------------------------------------------------------------------------------- :     -
AOTVOKIR3DS5*01    : ----------------------------------------------------------------------------------------------- :     -
AOTVOKIR2DS5*01v1  : ----------------------------------------------------------------------------------------------- :     -
AOTVOKIR2DS5*01v2  : ----------------------------------------------------------------------------------------------- :     -
AOTVOKIR3DS7*01    : ----------------------------------------------------------------------------------------------- :     -
AOTVOKIR3DS7*01v1  : ----------------------------------------------------------------------------------------------- :     -
AOTVOKIR3DL8*01    : ----------------------------------------------------------------------------------------------- :     -
AOTVOKIR3DL8*01v1  : ----------------------------------------------------------------------------------------------- :     -
AOTVOKIR3DS8*01v2  : ----------------------------------------------------------------------------------------------- :     -
AOTVOKIR3DL8*02    : ----------------------------------------------------------------------------------------------- :     -
AOTVOKIR4DL9*01    : ----------------------------------------------------------------------------------------------- :     -
AOTVOKIR4DS9*01v1  : ----------------------------------------------------------------------------------------------- :     -
AOTVOKIR3DL9*01v2  : ----------------------------------------------------------------------------------------------- :     -
AOTVOKIR4DL9*02    : ----------------------------------------------------------------------------------------------- :     -
AOTVOKIR4DL10*01   : ----------------------------------------------------------------------------------------------- :     -
```

```
AOTVOKIR3DL10*01v1 : ----------------------------------------------------------------------------------------------- :     -
AOTVOKIR4DS10*01v2 : ----------------------------------------------------------------------------------------------- :     -
AOTVOKIR3DS10*01v3 : ----------------------------------------------------------------------------------------------- :     -
AOTVOKIR3DS10*01v4 : ----------------------------------------------------------------------------------------------- :     -

                         *     12840         *     12860         *     12880         *     12900         *     12920
BAC_clone_Om       : AGGGAACACCTGAGCCTGGGTAACTTCTAAAGAAAACAGATATGTTTGGCTCACGGTTCCACAGGCTGTACTAGAAGCATGGCACCAGCATCTAT : 12920
AOTVOKIR3DL4*01    : ----------------------------------------------------------------------------------------------- :     -
AOTVOKIR3DL4*02    : ----------------------------------------------------------------------------------------------- :     -
AOTVOKIR3DL4*02v1  : ----------------------------------------------------------------------------------------------- :     -
AOTVOKIR3DL4*02v2  : ----------------------------------------------------------------------------------------------- :     -
AOTVOKIR3DL4*03    : ----------------------------------------------------------------------------------------------- :     -
AOTVOKIR3DS4*04    : ----------------------------------------------------------------------------------------------- :     -
AOTVOKIR3DS4*04v1  : ----------------------------------------------------------------------------------------------- :     -
AOTVOKIR3DS5*01    : ----------------------------------------------------------------------------------------------- :     -
AOTVOKIR2DS5*01v1  : ----------------------------------------------------------------------------------------------- :     -
AOTVOKIR2DS5*01v2  : ----------------------------------------------------------------------------------------------- :     -
AOTVOKIR3DS7*01    : ----------------------------------------------------------------------------------------------- :     -
AOTVOKIR3DS7*01v1  : ----------------------------------------------------------------------------------------------- :     -
AOTVOKIR3DL8*01    : ----------------------------------------------------------------------------------------------- :     -
AOTVOKIR3DL8*01v1  : ----------------------------------------------------------------------------------------------- :     -
AOTVOKIR3DS8*01v2  : ----------------------------------------------------------------------------------------------- :     -
AOTVOKIR3DL8*02    : ----------------------------------------------------------------------------------------------- :     -
AOTVOKIR4DL9*01    : ----------------------------------------------------------------------------------------------- :     -
AOTVOKIR4DS9*01v1  : ----------------------------------------------------------------------------------------------- :     -
AOTVOKIR3DL9*01v2  : ----------------------------------------------------------------------------------------------- :     -
AOTVOKIR4DL9*02    : ----------------------------------------------------------------------------------------------- :     -
AOTVOKIR4DL10*01   : ----------------------------------------------------------------------------------------------- :     -
AOTVOKIR3DL10*01v1 : ----------------------------------------------------------------------------------------------- :     -
AOTVOKIR4DS10*01v2 : ----------------------------------------------------------------------------------------------- :     -
AOTVOKIR3DS10*01v3 : ----------------------------------------------------------------------------------------------- :     -
AOTVOKIR3DS10*01v4 : ----------------------------------------------------------------------------------------------- :     -

                              *     12940         *     12960         *     12980         *     13000         *
BAC_clone_Om       : TTCTTGTGATGACCTGAGGCCGCTTTCACTCTGGCAGAAGGAAGGGGGCCTGTGTGTGCAGAGACCACAGAGATCACACA--GCAAGAGACGGAG : 13013
AOTVOKIR3DL4*01    : ----------------------------------------------------------------------------------------------- :     -
AOTVOKIR3DL4*02    : ----------------------------------------------------------------------------------------------- :     -
AOTVOKIR3DL4*02v1  : ----------------------------------------------------------------------------------------------- :     -
AOTVOKIR3DL4*02v2  : ----------------------------------------------------------------------------------------------- :     -
AOTVOKIR3DL4*03    : ----------------------------------------------------------------------------------------------- :     -
AOTVOKIR3DS4*04    : ----------------------------------------------------------------------------------------------- :     -
AOTVOKIR3DS4*04v1  : ------------------GCTGCTCCCACTCTGGCAGAAGAGAAGTGGAGTGTGTGTGTGCAGAGACCACAGAGATCACACAGCAAGAGAGGGAG :  1074
AOTVOKIR3DS5*01    : ----------------------------------------------------------------------------------------------- :     -
AOTVOKIR2DS5*01v1  : ----------------------------------------------------------------------------------------------- :     -
```

```
AOTVOKIR2DS5*01v2  : ----------------------------------------------------------------------------------------------- :     -
AOTVOKIR3DS7*01    : ----------------------------------------------------------------------------------------------- :     -
AOTVOKIR3DS7*01v1  : ----------------------------------------------------------------------------------------------- :     -
AOTVOKIR3DL8*01    : ----------------------------------------------------------------------------------------------- :     -
AOTVOKIR3DL8*01v1  : ----------------------------------------------------------------------------------------------- :     -
AOTVOKIR3DS8*01v2  : ----------------------------------------------------------------------------------------------- :     -
AOTVOKIR3DL8*02    : ----------------------------------------------------------------------------------------------- :     -
AOTVOKIR4DL9*01    : ----------------------------------------------------------------------------------------------- :     -
AOTVOKIR4DS9*01v1  : ----------------------------------------------------------------------------------------------- :     -
AOTVOKIR3DL9*01v2  : ----------------------------------------------------------------------------------------------- :     -
AOTVOKIR4DL9*02    : ----------------------------------------------------------------------------------------------- :     -
AOTVOKIR4DL10*01   : ----------------------------------------------------------------------------------------------- :     -
AOTVOKIR3DL10*01v1 : ----------------------------------------------------------------------------------------------- :     -
AOTVOKIR4DS10*01v2 : ----------------------------------------------------------------------------------------------- :     -
AOTVOKIR3DS10*01v3 : ----------------------------------------------------------------------------------------------- :     -
AOTVOKIR3DS10*01v4 : ----------------------------------------------------------------------------------------------- :     -

                     13020         *     13040         *     13060         *     13080         *     13100         *
BAC_clone_Om       : CAGGAGGCGGAGGTGCGATGGAGCTTCCAAGTTCTCCTTCACAACCAACTCTCCAGGAAGTAATAGAGGGAAAACTTGCTAACCCCGTCTCATGG : 13108
AOTVOKIR3DL4*01    : ----------------------------------------------------------------------------------------------- :     -
AOTVOKIR3DL4*02    : ----------------------------------------------------------------------------------------------- :     -
AOTVOKIR3DL4*02v1  : ----------------------------------------------------------------------------------------------- :     -
AOTVOKIR3DL4*02v2  : ----------------------------------------------------------------------------------------------- :     -
AOTVOKIR3DL4*03    : ----------------------------------------------------------------------------------------------- :     -
AOTVOKIR3DS4*04    : ----------------------------------------------------------------------------------------------- :     -
AOTVOKIR3DS4*04v1  : CGGGAGGCGGAG----------------------------------------------------------------------------------- :  1086
AOTVOKIR3DS5*01    : ----------------------------------------------------------------------------------------------- :     -
AOTVOKIR2DS5*01v1  : ----------------------------------------------------------------------------------------------- :     -
AOTVOKIR2DS5*01v2  : ----------------------------------------------------------------------------------------------- :     -
AOTVOKIR3DS7*01    : ----------------------------------------------------------------------------------------------- :     -
AOTVOKIR3DS7*01v1  : ----------------------------------------------------------------------------------------------- :     -
AOTVOKIR3DL8*01    : ----------------------------------------------------------------------------------------------- :     -
AOTVOKIR3DL8*01v1  : ----------------------------------------------------------------------------------------------- :     -
AOTVOKIR3DS8*01v2  : ----------------------------------------------------------------------------------------------- :     -
AOTVOKIR3DL8*02    : ----------------------------------------------------------------------------------------------- :     -
AOTVOKIR4DL9*01    : ----------------------------------------------------------------------------------------------- :     -
AOTVOKIR4DS9*01v1  : ----------------------------------------------------------------------------------------------- :     -
AOTVOKIR3DL9*01v2  : ----------------------------------------------------------------------------------------------- :     -
AOTVOKIR4DL9*02    : ----------------------------------------------------------------------------------------------- :     -
AOTVOKIR4DL10*01   : ----------------------------------------------------------------------------------------------- :     -
AOTVOKIR3DL10*01v1 : ----------------------------------------------------------------------------------------------- :     -
AOTVOKIR4DS10*01v2 : ----------------------------------------------------------------------------------------------- :     -
AOTVOKIR3DS10*01v3 : ----------------------------------------------------------------------------------------------- :     -
AOTVOKIR3DS10*01v4 : ----------------------------------------------------------------------------------------------- :     -
```

```
                            13120         *     13140         *     13160         *     13180         *     13200
BAC_clone_Om       : GGACAGTATTAATCTATTCATGATGGATCCATCCCCATGAACCAACCACCTCCCGCTAAGCCCAACCTCCCACACTGGGGGTTAAACTTCAATGT : 13203
AOTVOKIR3DL4*01    : ----------------------------------------------------------------------------------------------- :     -
AOTVOKIR3DL4*02    : ----------------------------------------------------------------------------------------------- :     -
AOTVOKIR3DL4*02v1  : ----------------------------------------------------------------------------------------------- :     -
AOTVOKIR3DL4*02v2  : ----------------------------------------------------------------------------------------------- :     -
AOTVOKIR3DL4*03    : ----------------------------------------------------------------------------------------------- :     -
AOTVOKIR3DS4*04    : ----------------------------------------------------------------------------------------------- :     -
AOTVOKIR3DS4*04v1  : ----------------------------------------------------------------------------------------------- :     -
AOTVOKIR3DS5*01    : ----------------------------------------------------------------------------------------------- :     -
AOTVOKIR2DS5*01v1  : ----------------------------------------------------------------------------------------------- :     -
AOTVOKIR2DS5*01v2  : ----------------------------------------------------------------------------------------------- :     -
AOTVOKIR3DS7*01    : ----------------------------------------------------------------------------------------------- :     -
AOTVOKIR3DS7*01v1  : ----------------------------------------------------------------------------------------------- :     -
AOTVOKIR3DL8*01    : ----------------------------------------------------------------------------------------------- :     -
AOTVOKIR3DL8*01v1  : ----------------------------------------------------------------------------------------------- :     -
AOTVOKIR3DS8*01v2  : ----------------------------------------------------------------------------------------------- :     -
AOTVOKIR3DL8*02    : ----------------------------------------------------------------------------------------------- :     -
AOTVOKIR4DL9*01    : ----------------------------------------------------------------------------------------------- :     -
AOTVOKIR4DS9*01v1  : ----------------------------------------------------------------------------------------------- :     -
AOTVOKIR3DL9*01v2  : ----------------------------------------------------------------------------------------------- :     -
AOTVOKIR4DL9*02    : ----------------------------------------------------------------------------------------------- :     -
AOTVOKIR4DL10*01   : ----------------------------------------------------------------------------------------------- :     -
AOTVOKIR3DL10*01v1 : ----------------------------------------------------------------------------------------------- :     -
AOTVOKIR4DS10*01v2 : ----------------------------------------------------------------------------------------------- :     -
AOTVOKIR3DS10*01v3 : ----------------------------------------------------------------------------------------------- :     -
AOTVOKIR3DS10*01v4 : ----------------------------------------------------------------------------------------------- :     -

                           *     13220         *     13240         *     13260         *     13280         *     13300
BAC_clone_Om       : GAGGTTTGAAGAGGATCAAACATCTAAACCAAAGCAGTGGTACCATCAGCTCTTTCTGTGGTTACTATGAGAACCGTAACTGAGCCAGCAGGGGA : 13298
AOTVOKIR3DL4*01    : ----------------------------------------------------------------------------------------------- :     -
AOTVOKIR3DL4*02    : ----------------------------------------------------------------------------------------------- :     -
AOTVOKIR3DL4*02v1  : ----------------------------------------------------------------------------------------------- :     -
AOTVOKIR3DL4*02v2  : ----------------------------------------------------------------------------------------------- :     -
AOTVOKIR3DL4*03    : ----------------------------------------------------------------------------------------------- :     -
AOTVOKIR3DS4*04    : ----------------------------------------------------------------------------------------------- :     -
AOTVOKIR3DS4*04v1  : ----------------------------------------------------------------------------------------------- :     -
AOTVOKIR3DS5*01    : ----------------------------------------------------------------------------------------------- :     -
AOTVOKIR2DS5*01v1  : ----------------------------------------------------------------------------------------------- :     -
AOTVOKIR2DS5*01v2  : ----------------------------------------------------------------------------------------------- :     -
AOTVOKIR3DS7*01    : ----------------------------------------------------------------------------------------------- :     -
AOTVOKIR3DS7*01v1  : ----------------------------------------------------------------------------------------------- :     -
AOTVOKIR3DL8*01    : ----------------------------------------------------------------------------------------------- :     -
AOTVOKIR3DL8*01v1  : ----------------------------------------------------------------------------------------------- :     -
```

```
AOTVOKIR3DS8*01v2   : ----------------------------------------------------------------------------------------------- :     -
AOTVOKIR3DL8*02     : ----------------------------------------------------------------------------------------------- :     -
AOTVOKIR4DL9*01     : ----------------------------------------------------------------------------------------------- :     -
AOTVOKIR4DS9*01v1   : ----------------------------------------------------------------------------------------------- :     -
AOTVOKIR3DL9*01v2   : ----------------------------------------------------------------------------------------------- :     -
AOTVOKIR4DL9*02     : ----------------------------------------------------------------------------------------------- :     -
AOTVOKIR4DL10*01    : ----------------------------------------------------------------------------------------------- :     -
AOTVOKIR3DL10*01v1  : ----------------------------------------------------------------------------------------------- :     -
AOTVOKIR4DS10*01v2  : ----------------------------------------------------------------------------------------------- :     -
AOTVOKIR3DS10*01v3  : ----------------------------------------------------------------------------------------------- :     -
AOTVOKIR3DS10*01v4  : ----------------------------------------------------------------------------------------------- :     -

                               *     13320           *     13340           *     13360           *     13380           *
BAC_clone_Om        : TAGCTGGGTCTCCCACCATCTGGGTGCTTGTCCTAATGAGACGCTGTGTGGTTACCTGGCAATGAAGAGATGAGAGACGATCCCTGAAGAGGAAC : 13393
AOTVOKIR3DL4*01     : ----------------------------------------------------------------------------------------------- :     -
AOTVOKIR3DL4*02     : ----------------------------------------------------------------------------------------------- :     -
AOTVOKIR3DL4*02v1   : ----------------------------------------------------------------------------------------------- :     -
AOTVOKIR3DL4*02v2   : ----------------------------------------------------------------------------------------------- :     -
AOTVOKIR3DL4*03     : ----------------------------------------------------------------------------------------------- :     -
AOTVOKIR3DS4*04     : ----------------------------------------------------------------------------------------------- :     -
AOTVOKIR3DS4*04v1   : ----------------------------------------------------------------------------------------------- :     -
AOTVOKIR3DS5*01     : ----------------------------------------------------------------------------------------------- :     -
AOTVOKIR2DS5*01v1   : ----------------------------------------------------------------------------------------------- :     -
AOTVOKIR2DS5*01v2   : ----------------------------------------------------------------------------------------------- :     -
AOTVOKIR3DS7*01     : ----------------------------------------------------------------------------------------------- :     -
AOTVOKIR3DS7*01v1   : ----------------------------------------------------------------------------------------------- :     -
AOTVOKIR3DL8*01     : ----------------------------------------------------------------------------------------------- :     -
AOTVOKIR3DL8*01v1   : ----------------------------------------------------------------------------------------------- :     -
AOTVOKIR3DS8*01v2   : ----------------------------------------------------------------------------------------------- :     -
AOTVOKIR3DL8*02     : ----------------------------------------------------------------------------------------------- :     -
AOTVOKIR4DL9*01     : ----------------------------------------------------------------------------------------------- :     -
AOTVOKIR4DS9*01v1   : ----------------------------------------------------------------------------------------------- :     -
AOTVOKIR3DL9*01v2   : ----------------------------------------------------------------------------------------------- :     -
AOTVOKIR4DL9*02     : ----------------------------------------------------------------------------------------------- :     -
AOTVOKIR4DL10*01    : ----------------------------------------------------------------------------------------------- :     -
AOTVOKIR3DL10*01v1  : ----------------------------------------------------------------------------------------------- :     -
AOTVOKIR4DS10*01v2  : ----------------------------------------------------------------------------------------------- :     -
AOTVOKIR3DS10*01v3  : ----------------------------------------------------------------------------------------------- :     -
AOTVOKIR3DS10*01v4  : ----------------------------------------------------------------------------------------------- :     -

                                                                                                   EXON 7
                      13400           *     13420           *     13440           *     13460           *     13480           *
BAC_clone_Om        : TGCCGTGGTCAGCTTCTTACTGTGTTCCCATCTTCCTCCAGGTAACCCCAGACACCTGCATGTTCTGATTGGGTCCTCAGTGGTCATCATCCTCT : 13488
AOTVOKIR3DL4*01     : -----------------------------------------GTAACCCCAGACACCTGCACATTCTGATTGGGTCCTCAGTGGTCATCATCCTTT :  1051
AOTVOKIR3DL4*02     : -----------------------------------------GTAACCCCAGACACCTGCACGTTCTGATTGGGTCCTCAGTGGTCATCATCCTTT :  1051
```

```
AOTVOKIR3DL4*02v1  : -----------------------------------------GTAACCCCAGACACCTGCACGTTCTGATTGGGTCCTCAGTGGTCATCATCCTTT : 1015
AOTVOKIR3DL4*02v2  : -----------------------------------------GTAACCCCAGACACCTGCACGTTCTGATTGGGTCCTCAGTGGTCATCATCCTTT : 1135
AOTVOKIR3DL4*03    : -----------------------------------------GTAACCCCAGACACCTGCACATTCTGATTGGGTCCTCAGTGGTCATCATCCTTT : 1051
AOTVOKIR3DS4*04    : -----------------------------------------GTAACCCCAGACACCTGCACGTTCTGATTGGGTCCTCAGTGGTCATCATCCTTT : 1051
AOTVOKIR3DS4*04v1  : -----------------------------------------GTAACCCCAGACACCTGCACGTTCTGA--------------------------- : 1110
AOTVOKIR3DS5*01    : -----------------------------------------GTTTCCCCAGACACCTGCACATTCTCATTCAGTGCTCAGTGGTCATCATCCCCT : 1051
AOTVOKIR2DS5*01v1  : -----------------------------------------GTTTCCCCAGACACCTGCACATTCTCATTCAGTGCTCAGTGGTCATCATCCCCT :  853
AOTVOKIR2DS5*01v2  : -----------------------------------------GTTTCCCCAGACACCTGCACATTCTCATTCAGTGCTCAGTGGTCATCATCCCCT :  766
AOTVOKIR3DS7*01    : -----------------------------------------GTTTGCCCAGGCACCTGCACGTTCTCATTCGGTGCTCAGTGGTCATCATCCCCT : 1051
AOTVOKIR3DS7*01v1  : -----------------------------------------GTTTGCCCAGGCACCTGCACGTTCTCATTCGGTGCTCAGTGGTCATCATCCCCT : 1220
AOTVOKIR3DL8*01    : -----------------------------------------GTAACCTCAGACACCTGCACGTTCTGATTGGGTCCTCAGTGGTCATCATCCTCT : 1051
AOTVOKIR3DL8*01v1  : -----------------------------------------GTAACCTCAGACACCTGCACGTTCTGATTGGGTCCTCAGTGGTCATCATCCTCT : 1000
AOTVOKIR3DS8*01v2  : -----------------------------------------GTAACCTCAGACACCTGCACGTTCTGATTGGGTCCTCAGTGGTCATCATCCTCT : 1051
AOTVOKIR3DL8*02    : -----------------------------------------GTAACCTCAGACACCTGCACGTTCTGATTGGGTCCTCAGTGGTCATCATCCTCT : 1051
AOTVOKIR4DL9*01    : -----------------------------------------GTAACCCCAGACACCTGCACGTTCTGATTGGGTCCTCAGTAGTCATCATCCTCT : 1336
AOTVOKIR4DS9*01v1  : ----------------------------------------------------------------------------------------------- :    -
AOTVOKIR3DL9*01v2  : -----------------------------------------GTAACCCCAGACACCTGCACGTTCTGATTGGGTCCTCAGTAGTCATCATCCTCT : 1138
AOTVOKIR4DL9*02    : -----------------------------------------GTAACCCCAGACACCTGCACGTTCTGATTGGGTCCTCAGTAGTCATCATCCTCT : 1336
AOTVOKIR4DL10*01   : -----------------------------------------GTAACCCCAGACACCTGCACGTTCTGATTGGGTCCTCAGTGGTCATCATCCTCT : 1336
AOTVOKIR3DL10*01v1 : -----------------------------------------GTAACCCCAGACACCTGCACGTTCTGATTGGGTCCTCAGTGGTCATCATCCTCT : 1135
AOTVOKIR4DS10*01v2 : -----------------------------------------GTAACCCCAGACACCTGCACGTTCTGATTGGGTCCTCAGTGGTCATCATCCTCT : 1336
AOTVOKIR3DS10*01v3 : -----------------------------------------GTAACCCCAGACACCTGCACGTTCTGATTGGGTCCTCAGTGGTCATCATCCTCT : 1051
AOTVOKIR3DS10*01v4 : ----------------------------------------------------------------------------------------------- :    -
                                                              gt  cc cagacacctgcac ttct att  gt ctcagt gtcatcatcc  t

                          13500         *       13520         *       13540         *       13560         *       13580
BAC_clone_Om       : TCATCATCCTCCTCTTCCTTCTCATTCATCGCTGGTGGTCCAACAAAAAGAGTAAGTCTCATGAAGCAGAGGCCGGAGACCTCAGGGCCGTGTGC : 13583
AOTVOKIR3DL4*01    : TCATCATCCTCCTCTTCTTTCTCATTTGTCGCTGGTGGTCCAACAAAAAGA-------------------------------------------- : 1102
AOTVOKIR3DL4*02    : TCATCATCCTCCTCTTCTTTCTCATTTGTCGCTGGTGGTCCAACAAAAAGA-------------------------------------------- : 1102
AOTVOKIR3DL4*02v1  : TCATCATCCTCCTCTTCTTTCTCATTTGTCGCTGGTGGTCCAACAAAAAGA-------------------------------------------- : 1066
AOTVOKIR3DL4*02v2  : TCATCATCCTCCTCTTCTTTCTCATTTGTCGCTGGTGGTCCAACAAAAAGA-------------------------------------------- : 1186
AOTVOKIR3DL4*03    : TCATCATCCTCCTCTTCTTTCTCATTTGTCGCTGGTGGTCCAACAAAAAGA-------------------------------------------- : 1102
AOTVOKIR3DS4*04    : TCATCATCCTCCTCTTCTTTCTCATTTGTCGCTGGTGGTCCAACAAAAAGA-------------------------------------------- : 1102
AOTVOKIR3DS4*04v1  : ----------------------------------------------------------------------------------------------- :    -
AOTVOKIR3DS5*01    : TCACCATCCTCCTCTTCTTTCTCCTTCATCTCTGGTGCTCTGACAAAAGAAG-ACGTCTCACAAAGCAGAGTCCAGAGACCCCAGGGCCATGTGT : 1145
AOTVOKIR2DS5*01v1  : TCACCATCCTCCTCTTCTTTCTCCTTCATCTCTGGTGCTCTGACAAAAGAAG-ACGTCTCACAAAGCAGAGTCCAGAGACCCCAGGGCCATGTGT :  947
AOTVOKIR2DS5*01v2  : TCACCATCCTCCTCTTCTTTCTCCTTCATCTCTGGTGCTCTGACAAAAGAAG-ACGTCTCACAAAGCAGAGTCCAGAGACCCCAGGGCCATGTGT :  860
AOTVOKIR3DS7*01    : TCACCATCCTCCTCTTCTTTCTCCTTCATCTCTGGTGCTCTGACAAAAGAA-TACGTCTCACAAAGCAGAGTCCAGAGACCCCAGGGCCATGTGG : 1145
AOTVOKIR3DS7*01v1  : TCACCATCCTCCTCTTCTTTCTCCTTCATCTCTGGTGCTC------------------------------------------------------- : 1260
AOTVOKIR3DL8*01    : TCACCATCCTCCTCTTCTTTCTCATTCGTCGCTGGTGGTCCAACAAAAAGA-------------------------------------------- : 1102
AOTVOKIR3DL8*01v1  : TCACCATCCTCCTCTTCTTTCTCATTCGTCGCTGGTGGTCCAACAAAAAGA-------------------------------------------- : 1051
AOTVOKIR3DS8*01v2  : TCACCATCCTCCTCTTCTTTCTCATTCGTCGCTGGTGGTCCAACAAAAAGA-------------------------------------------- : 1102
AOTVOKIR3DL8*02    : TCACCATCCTCCTCTTCTTTCTCATTCGTCGTTGGTGGTCCAACAAAAAGA-------------------------------------------- : 1102
AOTVOKIR4DL9*01    : TCATCATCCTCCTCTTCTTTCTCATTCGTCGCTGGTGGTCCAACAAAAAGA-------------------------------------------- : 1387
AOTVOKIR4DS9*01v1  : ----------------------------------------------------------------------------------------------- :    -
AOTVOKIR3DL9*01v2  : TCATCATCCTCCTCTTCTTTCTCATTCGTCGCTGGTGGTCCAACAAAAAGA-------------------------------------------- : 1189
```

```
AOTVOKIR4DL9*02      : TCATCATCCTCCTCTTCTTTCTCATTCGTCGCTGGTGGTCCAACAAAAAGA-------------------------------------------- :  1387
AOTVOKIR4DL10*01     : TCATCATCCTCCTCTTCTTTCTCATTCGTCGCTGGTGGTCCAACAAAAAGA-------------------------------------------- :  1387
AOTVOKIR3DL10*01v1   : TCATCATCCTCCTCTTCTTTCTCATTCGTCGCTGGTGGTCCAACAAAAAGA-------------------------------------------- :  1186
AOTVOKIR4DS10*01v2   : TCATCATCCTCCTCTTCTTTCTCATTCGTCGCTGGTGGTCCAACAAAAAGA-------------------------------------------- :  1387
AOTVOKIR3DS10*01v3   : TCATCATCCTCCTCTTCTTTCTCATTCGTCGCTGGTGGTCCAACAAAAAGA-------------------------------------------- :  1102
AOTVOKIR3DS10*01v4   : ----------------------------------------------------------------------------------------------- :     -
                       tca catcctcctcttctttctc tt  tc ctggtg tc  acaaaa  a

                             *     13600         *     13620         *     13640         *     13660         *     13680
BAC_clone_Om         : GGAAGCGGGATGGGAGCACGCGGGTGTGTGTTCCTCATTGGCGGGGTGGTCTCTGGCCCAAGGCAGGAGCTGCAGAGGCAGGGCTTTCTAGAGAG : 13678
AOTVOKIR3DL4*01      : ----------------------------------------------------------------------------------------------- :     -
AOTVOKIR3DL4*02      : ----------------------------------------------------------------------------------------------- :     -
AOTVOKIR3DL4*02v1    : ----------------------------------------------------------------------------------------------- :     -
AOTVOKIR3DL4*02v2    : ----------------------------------------------------------------------------------------------- :     -
AOTVOKIR3DL4*03      : ----------------------------------------------------------------------------------------------- :     -
AOTVOKIR3DS4*04      : ----------------------------------------------------------------------------------------------- :     -
AOTVOKIR3DS4*04v1    : ----------------------------------------------------------------------------------------------- :     -
AOTVOKIR3DS5*01      : GGAAGCAGGATGG-AGCATGCAG------------------------------------------------------------------------ :  1167
AOTVOKIR2DS5*01v1    : GGAAGCAGGATGG-AGCATGCAG------------------------------------------------------------------------ :   969
AOTVOKIR2DS5*01v2    : GGAAGCAGGATGG-AGCATGCAG------------------------------------------------------------------------ :   882
AOTVOKIR3DS7*01      : GGAAGCAGGATGG-AGCATGCAG------------------------------------------------------------------------ :  1167
AOTVOKIR3DS7*01v1    : ----------------------------------------------------------------------------------------------- :     -
AOTVOKIR3DL8*01      : ----------------------------------------------------------------------------------------------- :     -
AOTVOKIR3DL8*01v1    : ----------------------------------------------------------------------------------------------- :     -
AOTVOKIR3DS8*01v2    : ----------------------------------------------------------------------------------------------- :     -
AOTVOKIR3DL8*02      : ----------------------------------------------------------------------------------------------- :     -
AOTVOKIR4DL9*01      : ----------------------------------------------------------------------------------------------- :     -
AOTVOKIR4DS9*01v1    : ----------------------------------------------------------------------------------------------- :     -
AOTVOKIR3DL9*01v2    : ----------------------------------------------------------------------------------------------- :     -
AOTVOKIR4DL9*02      : ----------------------------------------------------------------------------------------------- :     -
AOTVOKIR4DL10*01     : ----------------------------------------------------------------------------------------------- :     -
AOTVOKIR3DL10*01v1   : ----------------------------------------------------------------------------------------------- :     -
AOTVOKIR4DS10*01v2   : ----------------------------------------------------------------------------------------------- :     -
AOTVOKIR3DS10*01v3   : ----------------------------------------------------------------------------------------------- :     -
AOTVOKIR3DS10*01v4   : ----------------------------------------------------------------------------------------------- :     -


                                *     13700         *     13720         *     13740         *     13760         *
BAC_clone_Om         : AGCACCAGACACCCTGCCCCTGCCCTCAGCTCAGAATCAATGGGATGGAAATTGAGAGCTCTTCATGGGAGGGGTCCTTGCACTCAGAGAGACAG : 13773
AOTVOKIR3DL4*01      : ----------------------------------------------------------------------------------------------- :     -
AOTVOKIR3DL4*02      : ----------------------------------------------------------------------------------------------- :     -
AOTVOKIR3DL4*02v1    : ----------------------------------------------------------------------------------------------- :     -
AOTVOKIR3DL4*02v2    : ----------------------------------------------------------------------------------------------- :     -
AOTVOKIR3DL4*03      : ----------------------------------------------------------------------------------------------- :     -
AOTVOKIR3DS4*04      : ----------------------------------------------------------------------------------------------- :     -
AOTVOKIR3DS4*04v1    : ----------------------------------------------------------------------------------------------- :     -
```

```
AOTVOKIR3DS5*01     : ----------------------------------------------------------------------------------------------- :     -
AOTVOKIR2DS5*01v1   : ----------------------------------------------------------------------------------------------- :     -
AOTVOKIR2DS5*01v2   : ----------------------------------------------------------------------------------------------- :     -
AOTVOKIR3DS7*01     : ----------------------------------------------------------------------------------------------- :     -
AOTVOKIR3DS7*01v1   : ----------------------------------------------------------------------------------------------- :     -
AOTVOKIR3DL8*01     : ----------------------------------------------------------------------------------------------- :     -
AOTVOKIR3DL8*01v1   : ----------------------------------------------------------------------------------------------- :     -
AOTVOKIR3DS8*01v2   : ----------------------------------------------------------------------------------------------- :     -
AOTVOKIR3DL8*02     : ----------------------------------------------------------------------------------------------- :     -
AOTVOKIR4DL9*01     : ----------------------------------------------------------------------------------------------- :     -
AOTVOKIR4DS9*01v1   : ----------------------------------------------------------------------------------------------- :     -
AOTVOKIR3DL9*01v2   : ----------------------------------------------------------------------------------------------- :     -
AOTVOKIR4DL9*02     : ----------------------------------------------------------------------------------------------- :     -
AOTVOKIR4DL10*01    : ----------------------------------------------------------------------------------------------- :     -
AOTVOKIR3DL10*01v1  : ----------------------------------------------------------------------------------------------- :     -
AOTVOKIR4DS10*01v2  : ----------------------------------------------------------------------------------------------- :     -
AOTVOKIR3DS10*01v3  : ----------------------------------------------------------------------------------------------- :     -
AOTVOKIR3DS10*01v4  : ----------------------------------------------------------------------------------------------- :     -

                      13780         *      13800         *      13820         *      13840         *      13860         *
BAC_clone_Om        : AATGTGTGAGTCAGGCTGTTGGCAGCTGAGGGACCTCAGGCACTCATGGCCTCCCCCTGTGTGTTGGTGTCTGCACATGAAATGAGGACACAGAA : 13868
AOTVOKIR3DL4*01     : ----------------------------------------------------------------------------------------------- :     -
AOTVOKIR3DL4*02     : ----------------------------------------------------------------------------------------------- :     -
AOTVOKIR3DL4*02v1   : ----------------------------------------------------------------------------------------------- :     -
AOTVOKIR3DL4*02v2   : ----------------------------------------------------------------------------------------------- :     -
AOTVOKIR3DL4*03     : ----------------------------------------------------------------------------------------------- :     -
AOTVOKIR3DS4*04     : ----------------------------------------------------------------------------------------------- :     -
AOTVOKIR3DS4*04v1   : ----------------------------------------------------------------------------------------------- :     -
AOTVOKIR3DS5*01     : ----------------------------------------------------------------------------------------------- :     -
AOTVOKIR2DS5*01v1   : ----------------------------------------------------------------------------------------------- :     -
AOTVOKIR2DS5*01v2   : ----------------------------------------------------------------------------------------------- :     -
AOTVOKIR3DS7*01     : ----------------------------------------------------------------------------------------------- :     -
AOTVOKIR3DS7*01v1   : ----------------------------------------------------------------------------------------------- :     -
AOTVOKIR3DL8*01     : ----------------------------------------------------------------------------------------------- :     -
AOTVOKIR3DL8*01v1   : ----------------------------------------------------------------------------------------------- :     -
AOTVOKIR3DS8*01v2   : ----------------------------------------------------------------------------------------------- :     -
AOTVOKIR3DL8*02     : ----------------------------------------------------------------------------------------------- :     -
AOTVOKIR4DL9*01     : ----------------------------------------------------------------------------------------------- :     -
AOTVOKIR4DS9*01v1   : ----------------------------------------------------------------------------------------------- :     -
AOTVOKIR3DL9*01v2   : ----------------------------------------------------------------------------------------------- :     -
AOTVOKIR4DL9*02     : ----------------------------------------------------------------------------------------------- :     -
AOTVOKIR4DL10*01    : ----------------------------------------------------------------------------------------------- :     -
AOTVOKIR3DL10*01v1  : ----------------------------------------------------------------------------------------------- :     -
AOTVOKIR4DS10*01v2  : ----------------------------------------------------------------------------------------------- :     -
AOTVOKIR3DS10*01v3  : ----------------------------------------------------------------------------------------------- :     -
```

```
AOTVOKIR3DS10*01v4 : ----------------------------------------------------------------------------------------------- :     -

                                                                                      EXON 8
                            13880         *        13900         *        13920         *        13940         *        13960
BAC_clone_Om       : GGGCCCTCCCAGGTGCTTTGATGACTTCTGTCTCCTACAGATGCTGCTGTAATGAACCAAGAACCTGGGGAGGACAGAAGAGTGAACAGGGAGGT : 13963
AOTVOKIR3DL4*01    : ----------------------------------------ATGCTGCTGTAATGAACCAAGAGCCTGGGGAGGACAGAACAGTGAACAGGGAG-- :  1155
AOTVOKIR3DL4*02    : ----------------------------------------ATGCTGCTGTAATGAACCAAGAACCTGGGGAGGACAGAACAGTGAACAGGGAG-- :  1155
AOTVOKIR3DL4*02v1  : ----------------------------------------ATGCTGCTGTAATGAACCAAGAACCTGGGGAGGACAGAACAGTGAACAGGGAG-- :  1119
AOTVOKIR3DL4*02v2  : ----------------------------------------ATGCTGCTGTAATGAACCAAGAACCTGGGGAGGACAGAACAGTGAACAGGGAG-- :  1239
AOTVOKIR3DL4*03    : ----------------------------------------ATGCTGCTGTAATGAACCAAGAGCCTGGGGAGGACAGAACAGTGAACAGGGAG-- :  1155
AOTVOKIR3DS4*04    : ----------------------------------------------------------------------------------------------- :     -
AOTVOKIR3DS4*04v1  : ----------------------------------------------------------------------------------------------- :     -
AOTVOKIR3DS5*01    : ----------------------------------------ATGCTGCAGTCAAAGACCAAGAGCCTGGGGCAGACAGAACAGTGA---------- :  1209
AOTVOKIR2DS5*01v1  : ----------------------------------------ATGCTGCAGTCAAAGACCAAGAGCCTGGGGCAGACAGAACAGTGA---------- :  1011
AOTVOKIR2DS5*01v2  : ----------------------------------------ATGCTGCAGTCAAAGACCAAGAGCCTGGGGCAGACAGAACAGTGA---------- :   924
AOTVOKIR3DS7*01    : ----------------------------------------ATGCTGCAGTCAAAGACCAAGAGCCTGGGGTGGACAGGACAGTGA---------- :  1209
AOTVOKIR3DS7*01v1  : ----------------------------------------------------------------------------------------------- :     -
AOTVOKIR3DL8*01    : ----------------------------------------ATGCTGCTGTAATGAACCAAGAACCTGGGGAGGACAGAACAGTGAACAGGGAG-- :  1155
AOTVOKIR3DL8*01v1  : ----------------------------------------ATGCTGCTGTAATGAACCAAGAACCTGGGGAGGACAGAACAGTGAACAGGGAG-- :  1104
AOTVOKIR3DS8*01v2  : ----------------------------------------------------------------------------------------------- :     -
AOTVOKIR3DL8*02    : ----------------------------------------ATGCTGCTGTAATGAACCAAGAACCTGGGGAGGACAGAACAGTGAACAGGGAG-- :  1155
AOTVOKIR4DL9*01    : ----------------------------------------ATGCTGCTGTAATGAACCAAGAACCTGGGGAGGACAGAACAGTGAACAGGGAG-- :  1440
AOTVOKIR4DS9*01v1  : ----------------------------------------------------------------------------------------------- :     -
AOTVOKIR3DL9*01v2  : ----------------------------------------ATGCTGCTGTAATGAACCAAGAACCTGGGGAGGACAGAACAGTGAACAGGGAG-- :  1242
AOTVOKIR4DL9*02    : ----------------------------------------ATGCTGCTGTAATGAACCAAGAACCTGGGGAGGACAGAACAGTGAACAGGGAG-- :  1440
AOTVOKIR4DL10*01   : ----------------------------------------ATGCTGCTGTAATGAACCAAGAGCCTGGGGAGGACAGAACAGTGAACAGGGAG-- :  1440
AOTVOKIR3DL10*01v1 : ----------------------------------------ATGCTGCTGTAATGAACCAAGAGCCTGGGGAGGACAGAACAGTGAACAGGGAG-- :  1239
AOTVOKIR4DS10*01v2 : ----------------------------------------------------------------------------------------------- :     -
AOTVOKIR3DS10*01v3 : ----------------------------------------------------------------------------------------------- :     -
AOTVOKIR3DS10*01v4 : ----------------------------------------------------------------------------------------------- :     -

                         *        13980         *        14000         *        14020         *        14040         *        14060
BAC_clone_Om       : AGGTCCTCCTCAACCCAGGCTTGGGGTGGAGACTTATTCCCTAATAGTCCCAAAGAATGTGAACCCCTCCGTCACTCAACATTTCCTTCTCTCCA : 14058
AOTVOKIR3DL4*01    : ----------------------------------------------------------------------------------------------- :     -
AOTVOKIR3DL4*02    : ----------------------------------------------------------------------------------------------- :     -
AOTVOKIR3DL4*02v1  : ----------------------------------------------------------------------------------------------- :     -
AOTVOKIR3DL4*02v2  : ----------------------------------------------------------------------------------------------- :     -
AOTVOKIR3DL4*03    : ----------------------------------------------------------------------------------------------- :     -
AOTVOKIR3DS4*04    : ----------------------------------------------------------------------------------------------- :     -
AOTVOKIR3DS4*04v1  : ----------------------------------------------------------------------------------------------- :     -
AOTVOKIR3DS5*01    : ----------------------------------------------------------------------------------------------- :     -
AOTVOKIR2DS5*01v1  : ----------------------------------------------------------------------------------------------- :     -
AOTVOKIR2DS5*01v2  : ----------------------------------------------------------------------------------------------- :     -
AOTVOKIR3DS7*01    : ----------------------------------------------------------------------------------------------- :     -
AOTVOKIR3DS7*01v1  : ----------------------------------------------------------------------------------------------- :     -
```

```
AOTVOKIR3DL8*01     : ----------------------------------------------------------------------------------------------- :     -
AOTVOKIR3DL8*01v1   : ----------------------------------------------------------------------------------------------- :     -
AOTVOKIR3DS8*01v2   : ----------------------------------------------------------------------------------------------- :     -
AOTVOKIR3DL8*02     : ----------------------------------------------------------------------------------------------- :     -
AOTVOKIR4DL9*01     : ----------------------------------------------------------------------------------------------- :     -
AOTVOKIR4DS9*01v1   : ----------------------------------------------------------------------------------------------- :     -
AOTVOKIR3DL9*01v2   : ----------------------------------------------------------------------------------------------- :     -
AOTVOKIR4DL9*02     : ----------------------------------------------------------------------------------------------- :     -
AOTVOKIR4DL10*01    : ----------------------------------------------------------------------------------------------- :     -
AOTVOKIR3DL10*01v1  : ----------------------------------------------------------------------------------------------- :     -
AOTVOKIR4DS10*01v2  : ----------------------------------------------------------------------------------------------- :     -
AOTVOKIR3DS10*01v3  : ----------------------------------------------------------------------------------------------- :     -
AOTVOKIR3DS10*01v4  : ----------------------------------------------------------------------------------------------- :     -

                                                                              EXON 9
                               *       14080           *       14100           *       14120           *       14140           *
BAC_clone_Om        : GGACTCTGAAGAACAAGACCCTCAGGAGGTGACATATGCACAGCTCGATCACTGCATTTTCACGCAGAGAAAAATCACTCACCCCTCTCAGAGGC : 14153
AOTVOKIR3DL4*01     : -GACTCTGAAGAACAAGACTCTCAGGAGGTGACATACGCACAGCTGGATCACTGCGTTTTCACGCAGAGAAAAATCACTCATGCCTCTCAGAGGC :  1249
AOTVOKIR3DL4*02     : -GACTCTGAAGAACAAGACTCTCAGGAGGTGACATATGCACAGCTGGATCACTGCGTTTTCACGCAGAGAAAAATCACTCATGCCTCTCAGAGGC :  1249
AOTVOKIR3DL4*02v1   : -GACTCTGAAGAACAAGACTCTCAGGAGGTGACATATGCACAGCTGGATCACTGCGTTTTCACGCAGAGAAAAATCACTCATGCCTCTCAGAGGC :  1213
AOTVOKIR3DL4*02v2   : -GACTCTGAAGAACAAGACTCTCAGGAGGTGACATATGCACAGCTGGATCACTGCGTTTTCACGCAGAGAAAAATCACTCATGCCTCTCAGAGGC :  1333
AOTVOKIR3DL4*03     : -GACTCTGAAGAACAAGACTCTCAGGAGGTGACATACGCACAGCTGGATCACTGCGTTTTCACGCAGAGAAAAATCACTCATGCCTCTCAGAGGC :  1249
AOTVOKIR3DS4*04     : -GACTCTGAAGAACAAGACCCTCAGGAGG------------------------------------------------------------------ :  1130
AOTVOKIR3DS4*04v1   : ----------------------------------------------------------------------------------------------- :     -
AOTVOKIR3DS5*01     : ----------------------------------------------------------------------------------------------- :     -
AOTVOKIR2DS5*01v1   : ----------------------------------------------------------------------------------------------- :     -
AOTVOKIR2DS5*01v2   : ----------------------------------------------------------------------------------------------- :     -
AOTVOKIR3DS7*01     : ----------------------------------------------------------------------------------------------- :     -
AOTVOKIR3DS7*01v1   : ----------------------------------------------------------------------------------------------- :     -
AOTVOKIR3DL8*01     : -GACTCTGAAGAACAAGACTCTCAGGAGGTGACATACGCACAGCTGGATCACTGCGTTTTCACACAGAGAAAAATCACTCATGCCTCTCAGAGGC :  1249
AOTVOKIR3DL8*01v1   : -GACTCTGAAGAACAAGACTCTCAGGAGGTGACATACGCACAGCTGGATCACTGCGTTTTCACACAGAGAAAAATCACTCATGCCTCTCAGAGGC :  1198
AOTVOKIR3DS8*01v2   : -GACTC----------------------------------------------------------------------------------------- :  1107
AOTVOKIR3DL8*02     : -GACTCTGAAGAACAAGACTCTCAGGAGGTGACATACGCACAGCTGGATCACTGCGTTTTCACACAGAGAAAAATCACTCATGCCTCTCAGAGGC :  1249
AOTVOKIR4DL9*01     : -GACTCTGAAGAACAAGACCCTCAGGAGGTGACATATGCACAGCTGGATCACTGCGTTTTCACACAGAGAAAAATCACTTGCCCCTCTCAGAGGC :  1534
AOTVOKIR4DS9*01v1   : ----------------------------------------------------------------------------------------------- :     -
AOTVOKIR3DL9*01v2   : -GACTCTGAAGAACAAGACCCTCAGGAGGTGACATATGCACAGCTGGATCACTGCGTTTTCACACAGAGAAAAATCACTTGCCCCTCTCAGAGGC :  1336
AOTVOKIR4DL9*02     : -GACTCTGAAGAACAAGACCCTCAGGAGGTGACATATGCACAGCTGGATCACTGCGTTTTCACACAGAGAAAAATCACTTGCCCCTCTCAGAGGC :  1534
AOTVOKIR4DL10*01    : -GACTCTGAAGAACAAGACCCTCAGGAGGTGACATATGCACAGCTGGATCACTGCGTTTTCACACAGAGAAAAATCACTCGCCCTTCTCAGAGGC :  1534
AOTVOKIR3DL10*01v1  : -GACTCTGAAGAACAAGACCCTCAGGAGGTGACATATGCACAGCTGGATCACTGCGTTTTCACACAGAGAAAAATCACTCGCCCTTCTCAGAGGC :  1333
AOTVOKIR4DS10*01v2  : -GACTC----------------------------------------------------------------------------------------- :  1392
AOTVOKIR3DS10*01v3  : -GACTC----------------------------------------------------------------------------------------- :  1107
AOTVOKIR3DS10*01v4  : ----------------------------------------------------------------------------------------------- :     -


                      14160           *       14180           *       14200           *       14220           *
BAC_clone_Om        : CCAAGAGACCCCCAAAAGAAACCAGTGTGTACATGGAACTCCCAAATGCGGAGCCCAGATTGAAAGTTGTCTCCTGTCCATGA : 14236
```

```
AOTVOKIR3DL4*01      : CCAAGAGACCCCCAACAGAAACCAGCGTGTACATGGAACTCCCAAATGCGGAGCCCAGATTGAAAGTTGTCTCCTGTCCATGA :  1329
AOTVOKIR3DL4*02      : CCAAGAGACCCCCAACAGAAACCAGTGTGTACATGGAACTCCCAAATGCGGAGCCCAGATTGAAAGTTGTCTCCTGTCCATGA :  1329
AOTVOKIR3DL4*02v1    : CCAAGAGACCCCCAACAGAAACCAGTGTGTACATGGAACTCCCAAATGCGGAGCCCAGATTGAAAGTTGTCTCCTGTCCATGA :  1293
AOTVOKIR3DL4*02v2    : CCAAGAGACCCCCAACAGAAACCAGTGTGTACATGGAACTCCCAAATGCGGAGCCCAGATTGAAAGTTGTCTCCTGTCCATGA :  1413
AOTVOKIR3DL4*03      : CCAAGAGACCCCCAACAGAAACCAGCGTGTACATGGAACTCCCAAATGCGGAGCCCAGATTGAAAGTTGTCTCCTGTCCATGA :  1329
AOTVOKIR3DS4*04      : ----------------------------------------------------------------------------------- :     -
AOTVOKIR3DS4*04v1    : ----------------------------------------------------------------------------------- :     -
AOTVOKIR3DS5*01      : ----------------------------------------------------------------------------------- :     -
AOTVOKIR2DS5*01v1    : ----------------------------------------------------------------------------------- :     -
AOTVOKIR2DS5*01v2    : ----------------------------------------------------------------------------------- :     -
AOTVOKIR3DS7*01      : ----------------------------------------------------------------------------------- :     -
AOTVOKIR3DS7*01v1    : ----------------------------------------------------------------------------------- :     -
AOTVOKIR3DL8*01      : CCAAGAGACCCCCAACAGAAACCAGTGTGTACATGGAACTCCCAAATGCGGAGCCCAGATTGAAAGTTGTCTCCTGTCCATGA :  1329
AOTVOKIR3DL8*01v1    : CCAAGAGACCCCCAACAGAAACCAGTGTGTACATGGAACTCCCAAATGCGGAGCCCAGATTGAAAGTTGTCTCCTGTCCATG- :  1278
AOTVOKIR3DS8*01v2    : ----------------------------------------------------------------------------------- :     -
AOTVOKIR3DL8*02      : CCAAGAGACCCCCAACAGAAACCAGTGTGTACATGGAACTCCCAAATGCGGAGCCCAGATTGAAAGTTGTCTCCTGTCCATGA :  1329
AOTVOKIR4DL9*01      : CCATGAGAGCCCCAACAGAAACCAGTGTGTACATGGAACTCCCAAATGCAGAGCCCGGATTGAAAGTTGTCTCCTGTCCATGA :  1614
AOTVOKIR4DS9*01v1    : ----------------------------------------------------------------------------------- :     -
AOTVOKIR3DL9*01v2    : CCATGAGAGCCCCAACAGAAACCAGTGTGTACATGGAACTCCCAAATGCAGAGCCCGGATTGAAAGTTGTCTCCTGTCCATGA :  1416
AOTVOKIR4DL9*02      : CCATGAGAGCCCCAACAGAAACCAGTGTGTACATGGAACTCCCAAATGCAGAGCCCGGATTGAAAGTTGTCTCCTGTCCATGA :  1614
AOTVOKIR4DL10*01     : CCAAGAGACCCCCAACAGAAACCAGTGTGTACATGGAACTCCCAAATGCGGAGCCCAGATTGAAAGTTGTCTCCTGTCCATGA :  1614
AOTVOKIR3DL10*01v1   : CCAAGAGACCCCCAACAGAAACCAGTGTGTACATGGAACTCCCAAATGCGGAGCCCAGATTGAAAGTTGTCTCCTGTCCATG- :  1413
AOTVOKIR4DS10*01v2   : ----------------------------------------------------------------------------------- :     -
AOTVOKIR3DS10*01v3   : ----------------------------------------------------------------------------------- :     -
AOTVOKIR3DS10*01v4   : ----------------------------------------------------------------------------------- :     -
```
